# An adamantane‐based ligand as a novel chemical tool for thermosensory TRPM8 channel therapeutic modulation

**DOI:** 10.1111/febs.70065

**Published:** 2025-03-23

**Authors:** Angela Lamberti, Silvio Aprile, David Cabañero, Fabio Travagin, Laura Butron, Gregorio Fernández‐Ballester, Gian Cesare Tron, Asia Fernández‐Carvajal, Antonio Ferrer‐Montiel, Ubaldina Galli

**Affiliations:** ^1^ Instituto de Investigación, Desarrollo e Innovación en Biotecnología Sanitaria de Elche (IDiBE) Universidad Miguel Hernández de Elche Spain; ^2^ Department of Pharmaceutical Sciences Università degli Studi del Piemonte Orientale Novara Italy

**Keywords:** cold allodynia, drug discovery, ion channel, medicinal chemistry, neuropathy

## Abstract

Transient receptor potential cation channel subfamily M member 8 (TRPM8) is a nonselective thermosensory cation channel expressed in peripheral nociceptor terminals where it transduces cold temperatures and cooling agents such as menthol. TRPM8 dysfunction has been involved in disabling sensory symptoms, such as cold allodynia. In addition, its widespread expression has signaled this channel as a pivotal therapeutic target for a variety of diseases, from peripheral neuropathies to cancer. Thus, the design and therapeutic validation of TRPM8 antagonists is an important endeavor in biomedicine. To address this, we used the multicomponent Passerini and Ugi reactions to design a novel family of TRPM8 modulators using as a scaffold the adamantane ring that exhibits drug‐like qualities. These green chemistry transformations are ideal for the fast synthesis of libraries of medium complexity with minimal or no generation of waste by‐products. We report the identification of a family of TRPM8 agonists and antagonists. Among them, 2‐((3S,5S,7S)‐adamantan‐1‐ylamino)‐2‐oxoethyl [1,1′‐biphenyl]‐2‐carboxylate (referred to as compound **23**) is a potent and selective antagonist that reduces TRPM8‐induced neuronal firing in primary nociceptor cultures. Compound **23** exhibits 10‐fold higher potency for human TRPM8 (hTRPM8) than for hTRPV1 and hTRPA1 channels. Notably, local administration of compound **23** significantly attenuated oxaliplatin‐induced peripheral cold allodynia by modulating epidermal TRPM8 sensory endings. Thus, α‐acyloxy carboxamide **23** appears as a promising therapeutic candidate to topically intervene on TRPM8‐mediated peripheral neuropathies.

## Graphical abstract

Transient receptor potential cation channel subfamily M member 8 (TRPM8) is a cationic thermosensory channel crucial for cold sensation and menthol response. TRPM8 dysfunction leads to sensory symptoms such as cold allodynia. Targeting TRPM8 is important for treating diseases like peripheral neuropathies and cancer. Using green chemistry, we found compound **23**, as a potent TRPPM8 antagonist that, locally applied, attenuates oxaliplatin‐induced cold allodynia, showing promise treatment for chemotherapy‐induced neuropathies.

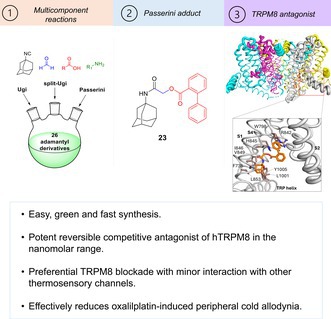

**Abbreviations**
Ac_2_O: acetic anhydride
AITC: allyl isothiocyanate
AMG333: (S)‐6‐(((3‐fluoro‐4‐(trifluoromethoxy)phenyl)(3‐fluoropyridin‐2‐yl)methyl)carbamoyl)nicotinic acid
AMTB: *N*‐(3‐aminopropyl)‐2‐{[(3‐methylphenyl)methyl]oxy}(20)‐*N*‐(2‐thienylmethyl)benzamide
AP: action potential
CDCl_3_
: deuterated chloroform
DMSO‐d_6_
: deuterated dimethyl sulfoxide
DRG: dorsal root ganglion
EC_50_
: half‐maximal effective concentration
EtOAc: ethyl acetate
HEK293: human embryonic kidney 293 cells
hTRP: human transient receptor potential
IC_50_
: half‐maximal inhibitory concentration
LC‐HRMS: liquid chromatography coupled to high‐resolution mass spectrometry
LC‐UV: liquid chromatography coupled to ultraviolet detector
MEA: multielectrode array
MeOH: methanol
MLM: mouse liver microsome
OIPN: oxaliplatin‐induced peripheral neuropathy
OXP: oxaliplatin
PE: petroleum ether
PF‐05105679: (R)‐3‐[(1‐(4‐fluorophenyl)ethyl)(quinolin‐3‐ylcarbonyl)amino]methylbenzoic acid
POCl_3_
: phosphorus oxychloride
SEM: standard error of the mean
TEA: triethylamine
TRP: transient receptor potential
TRPA1: transient receptor potential cation channel ankyrin 1
TRPM8: transient receptor potential cation channel melastatin 8
TRPV1: transient receptor potential cation channel vanilloid 1
WS12: (1R,2S,5R)‐*N*‐(4‐methoxyphenyl)‐5‐methyl‐2‐(propan‐2‐yl)cyclohexane‐1‐carboxamide

## Introduction

The transient receptor potential melastatin 8 (TRPM8) is a nonselective cation channel expressed in sensory neurons. As a member of the TRP family of ion channels, TRPM8 is involved in thermosensation playing a pivotal role in the perception of environmental cold, and the cooling sensation evoked by menthol [1, 2]. TRPM8 is active in the range of 8–28 °C and participates in the transduction of cold allodynia [3]. Structurally, the channel is a tetrameric protein assembled by four identical subunits arranged around a central aqueous pore. Each subunit contains six transmembrane segments (S1–S6) and intracellular N‐ and C‐terminals [4]. Upon activation, TRPM8 transits to an open conformation that allows the influx of cations, such as calcium (Ca^2+^) and sodium (Na^+^), favoring neuronal depolarization and action potential firing. Electrical impulses are transmitted through the spinal cord towards the sensory processing areas of the brain to elicit the cold sensation [5, 6].

TRPM8 has been involved in inflammation and immune responses in several tissues. For example, TRPM8 influences the release of inflammatory mediators from pulmonary epithelial cells leading to dysregulation of immune responses and chronic inflammation [7, 8]. The suppression of TRPM8 activity alleviated respiratory hypersensitivity in individuals with asthma [9]. Additionally, several studies have explored the role of TRPM8 in cancer cell proliferation, migration, and apoptosis [10, 11] positioning this channel as a potential target for cancer therapy in combination with other treatment modalities [12]. Furthermore, few studies have suggested that TRPM8 is involved in the perception of itch, modulating the transmission of itch‐related neural signals through peripheral pathways [13]. Moreover, the TRPM8 receptor has been also implicated in the etiology of dry eye syndrome, a condition characterized by insufficient tear production or poor tear quality, leading to discomfort and irritation [14, 15]. Notably, positive results of the phase 3 COMET trial of AR‐15512, a novel formulation of a TRPM8 drug candidate for dry eye syndrome, were announced [16].

TRPM8 has been also signaled as pivotal in the pathophysiology of chronic migraine [17, 18] and plays a major role in the pathogenesis of oxaliplatin‐induced cold allodynia (OIPN). Its pharmacological modulation has been explored as a potential strategy for pain alleviation [19, 20]. Thus, the design and validation of TRPM8 modulators are crucial for advancing our understanding on the contribution of TRPM8 to the etiology of neuropathic pain, inflammatory disorders, pruritus, and cancer and to select novel candidates for therapeutic development. Specifically, therapeutic inhibition of TRPM8 may offer antinociception for conditions characterized by cold‐associated pain (i.e., cold allodynia) [21] and provide alternatives to traditional analgesics with unwanted side effects such as opioid drugs [22].

In the past years, the development of TRPM8 modulators has faced challenges due to off‐target effects and species‐specific structural variations [23]. Compounds such as PF‐05105679 and AMG333, both orally active TRPM8 antagonists, progressed into clinical trials with the goal of investigating their potential in cold pain hypersensitivity and alleviating migraine symptoms, respectively [24, 25]. However, these promising compounds did not advance beyond phase I trials due to safety concerns. Thus, the discovery and development of therapeutically useful TRPM8 modulators remain a biomedical challenge.

In the pursuit of identifying new TRPM8 ligands with therapeutic potential, we combined the power of multicomponent reactions to readily achieve molecular diversity with adamantane that exhibits therapeutic properties. Its cyclic aliphatic hydrocarbon inspired us to consider it as a ‘potential surrogate’ for the terpene skeleton of TRPM8 ligands such as menthol, eucalyptol, and camphor. Furthermore, the choice of this caged compound as a potential modulator of the TRPM8 channel was motivated by the fact that the adamantane structure has already been reported capable of inhibiting different ion channels implicated in diseases such as viral infections and neurodegenerative disorders [26, 27]. In addition, the adamantyl group has been considered as a ‘lipophilic bullet’ capable of endowing favorable pharmacokinetic characteristics to molecules, such as a good propensity for crossing biological membranes [27, 28]. Beyond increasing partition coefficients (log*P* value) by about 3.1 log units, the adamantyl group has been also found to improve drug‐like qualities such as metabolic stability. Of note, the steric bulk of the adamantyl group can impede the access of hydrolytic enzymes (esterases, amidases), thereby increasing the plasma half‐life of lead compounds [27]. These unique properties of the adamantane scaffold make it an ideal candidate for modulating ion channels, showcasing its potential in the development of therapeutics for diseases resulting from TRPM8 channel dysregulation [29].

Among the dozens of multicomponent reactions discovered to date [30, 31, 32, 33] in terms of versatility, efficiency, exploratory power, and experimental simplicity, the Ugi [34, 35, 36, 37] and Passerini reactions [38, 39, 40, 41] remain very attractive. In these two isocyanide‐mediated reactions, four and three components are respectively reacted together to generate α‐aminoacyl amides and α‐acyloxy carboxamides. These multicomponent reactions produce focused libraries and have the advantage of minimizing the formation of waste products, thus fulfilling the criteria to be considered green chemistry [40]. In 2006, we discovered a new variant of the Ugi reaction, named split‐Ugi, whereby replacing a primary amine with a symmetric secondary diamine yielded a new drug‐like molecular scaffold [42].

Here, we report a family of adamantane‐based TRPM8 agonists and antagonists that increase the channel pharmacopeia. Among all ligands tested, the acyloxy carboxamide **23** (referred to as compound **23**) is a potent hTRPM8 antagonist showing a > 10‐fold blockade preference over the structurally related thermosensory channels TRPV1 and TRPA1. Furthermore, compound **23** reduced nociceptor excitability and locally attenuated oxaliplatin‐induced *in vivo* cold allodynia.

## Results

### Synthesis of compounds using the Passerini, Ugi, and split‐Ugi reactions

**Fig. 1:**
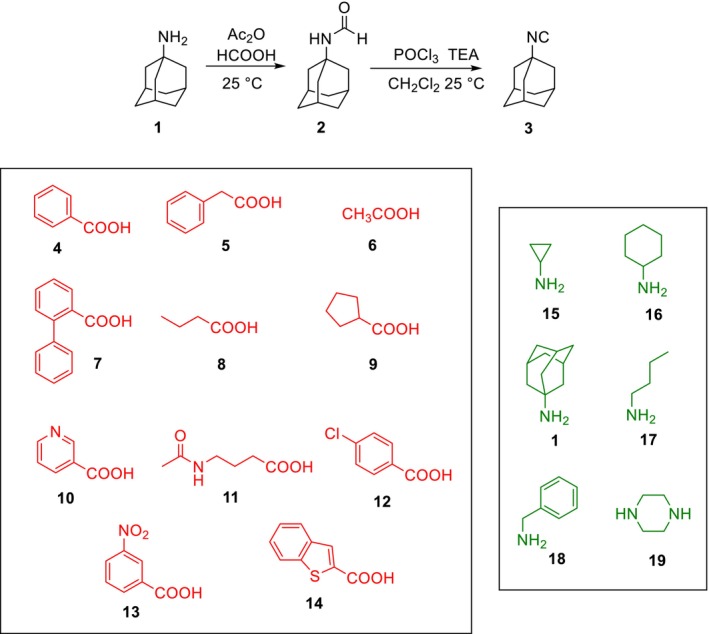
Preparation reaction of 1‐isocyanoadamantane 3 and chemical structure of carboxylic acids **4–14** (red color) and amines **15–19**, **1** (green color) used in this study.

To employ the three multicomponent reactions, we utilized 1‐isocyanoadamantane (**3**) as a lipophilic warhead. This reagent was prepared from 1‐aminoadamantane (**1**) through the intermediate 1‐adamantylformamide (**2**), as described previously [43] (Fig. 1). 1‐Isocyanoadamantane (**3**) was reacted with various commercially available carboxylic acids (**4–14**) and amines (**15–19**, **1**) (Fig. 1), always employing formaldehyde as the carbonyl component to avoid the introduction of a stereogenic center.

**Fig. 2:**
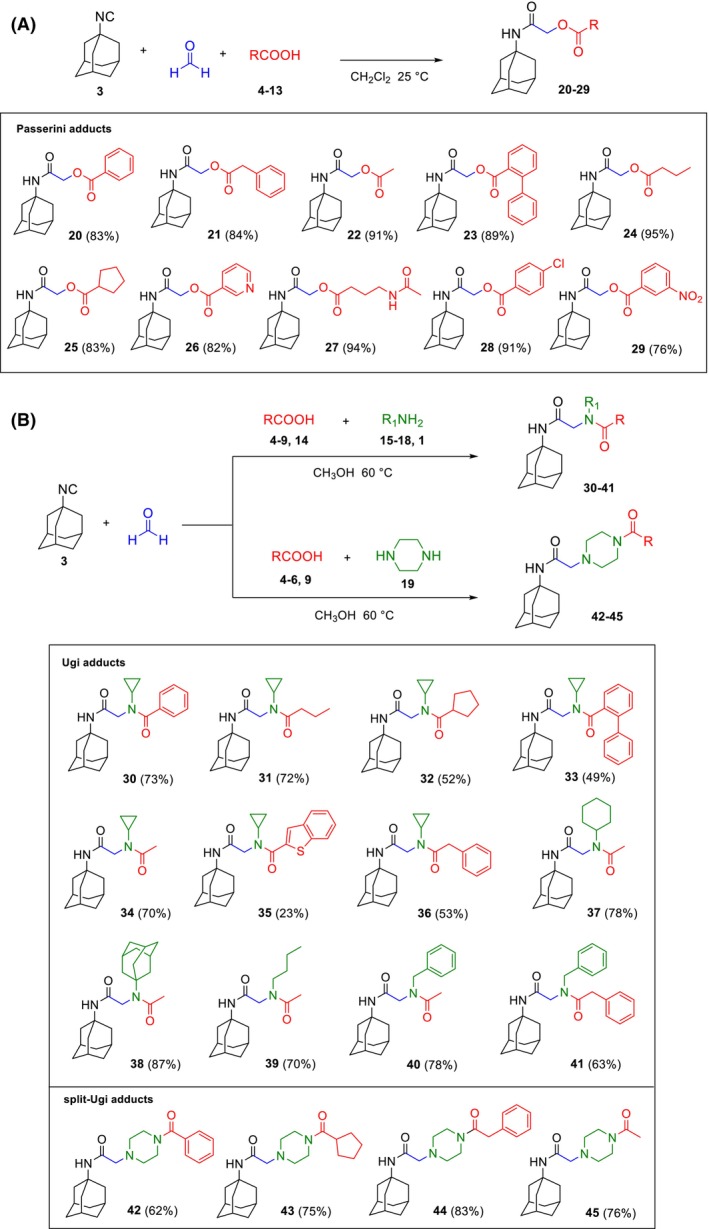
Passerini and Ugi reactions. (A) Preparation of α‐acyloxy carboxamides **20–29** via the Passerini reaction and compounds obtained. (B) Synthesis of α‐aminoacyl amides **30–41** and α‐acylpiperazino amides **42–45** exploiting the Ugi and split‐Ugi reactions, respectively, and compounds obtained (black color refers to isocyanide component, blue color refers to aldehyde component, red color refers to carboxylic acid component, and green color refers to amine component).

For the Passerini reaction, we mixed isocyanide **3**, formaldehyde, and a carboxylic acid (**4–13**) in dichloromethane and stirred the three‐component mixture at room temperature. We obtained 10 α‐acyloxy carboxamides (**20–29**) with excellent yields (Fig. 2A). Employing the Ugi reaction, we obtained 12 α‐aminoacyl amides (**30–41**) by mixing the 4 components (isocyanide **3**, formaldehyde, a carboxylic acid **4–9**, **14** and a primary amine **1**, **15–18**) in methanol and stirring them at 60 °C (Fig. 2B). Finally, we carried out the split‐Ugi reaction [42] to prepare 4 α‐acylpiperazino amides (**42–45**) using carboxylic acids **4–6** and **9** (Fig. 2B). In this variant of the Ugi reaction, piperazine **19** was added to the other 3 components, instead of a primary amine, maintaining the same experimental conditions. ^1^H‐NMR, ^13^C‐NMR spectra, and HRMS spectra of final compounds **20–45** are reported in Figs S1–S78.

### Ca^2+^‐microfluorometry to screen for agonist/antagonist activity of synthesized compounds

We first evaluated the series of compounds **20–29** synthesized using the Passerini‐Adamantane reaction. All compounds were screened for channel agonism and antagonism on recombinant human TRPM8 stably expressed in HEK293 cells (hTRPM8‐HEK293) with Ca^2+^ microfluorimetry. For agonistic activity, we directly assessed compound‐induced Ca^2+^ influx into hTRPM8‐HEK293 cells and compared it with the activity of WS‐12, a well‐known potent hTPRM8 agonist. Compound‐induced TRPM8‐mediated Ca^2+^ influx was confirmed using untransfected HEK293 cells and the channel blocker AMTB. For antagonistic activity, modulation of WS‐12‐induced Ca^2+^ influx was evaluated. The concentration of compounds to reach half of channel activation (EC_50_) or to block half‐channel activity evoked by WS‐12 (IC_50_) are reported in Table 1, along with the percentage of maximal activation or blockade (Efficacy).

**Table 1:** Screening for agonist/antagonist activity of synthesized compounds **20–45**. The experiments were conducted three times independently (*N* = 3), with each determination performed in triplicate (*n* = 3). The data are presented as the mean value accompanied by the standard deviation (SD).

Compound	hTRPM8					
	R	Activity	EC_50_ (μm)	IC_50_ (μm)	Hill coefficient	Efficacy (%)
**20**	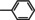	Antagonist		1.8 ± 1.1	0.4 ± 1.2	60
**21**	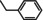	Antagonist		1.5 ± 1.1	0.5 ± 2.3	44
**22**	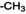	Agonist	1.1 ± 1.5		0.4 ± 1.3	64
**23**	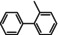	Antagonist		0.2 ± 1.3	0.5 ± 0.9	100
**24**	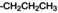	Agonist	19 ± 2.0		0.6 ± 0.2	19
**25**	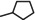	Antagonist		45 ± 2.0	0.8 ± 3.0	57
**26**	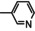	Agonist	1.9 ± 1.6		0.4 ± 6.4	72
**27**	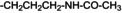	Agonist	0.6 ± 1.2		0.3 ± 1.6	60
**28**	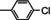	Agonist	1.2 ± 1.2		0.1 ± 0.2	87
**29**	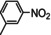	Antagonist		2.3 ± 1.1	0.5 ± 3.0	69
**30**	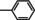	Agonist	1.5 ± 1.5		0.7 ± 1.6	92
**31**	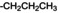	Agonist	0.3 ± 1.9		0.7 ± 2.5	72
**32**	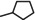	Agonist	0.4 ± 1.7		0.2 ± 1.5	56
**33**	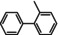	Antagonist		1.1 ± 1.2	2.0 ± 7.8	48
**34**	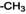	Agonist	0.2 ± 1.9		0.3 ± 14	54
**35**	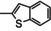	Antagonist		0.6 ± 1.2	2.0 ± 4.3	40
**36**	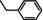	Agonist	4.9 ± 1.7		2.3 ± 2.6	47
**37**	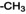	Agonist	0.2 ± 3.0		0.9 ± 9.2	48
**38**	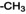	Agonist	2.0 ± 1.4		0.3 ± 3.6	65
**39**	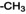	Agonist	0.5 ± 1.4		0.4 ± 9.1	64
**40**	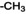	Agonist	1.4 ± 1.4		0.7 ± 2.4	86
**41**	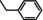	Agonist	0.9 ± 1.1		2.1 ± 1.0	49
**42**	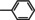	Agonist	5.4 ± 1.5		0.4 ± 4.3	73
**43**	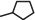	Agonist	9.3 ± 1.5		0.6 ± 1.8	82
**44**	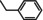	Agonist	9.6 ± 1.5		0.7 ± 4.0	69
**45**	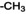	Agonist	2.3 ± 1.6		0.7 ± 2.1	54

We found that compounds **20**, **21**, **23**, **25**, and **29** behave as hTRPM8 antagonists, blocking WS‐12 responses with potencies ranging from 0.2 to 45 μm (Table 1). The most potent antagonist was compound **23** that exhibited an IC_50_ value of 0.2 ± 1.3 μm and 100% efficacy. The lowest activity was observed in compound **25**, suggesting that a bulky aromatic group at position R was important for potent antagonistic activity. Compounds **22**, **24**, and **27** exhibited an agonistic activity with potency ranging from 0.6 to 19 μm. Data show that increasing the hydrocarbon side chain (**22** and **24**) produced a decrease in potency, that can be compensated if the increase in size is accompanied by the addition of nucleophile atoms such as nitrogen and oxygen (**27**). Interestingly, compounds **26** and **28**, containing an aromatic group at the R position, displayed agonist activity contrasting with the antagonistic action of compounds **20** and **29**. This suggests that changes in the aromatic ring modulated the interaction of these ligands with molecular determinants of the channel binding site, differentially impacting the allosteric conformational changes needed for channel activation. These results provide a novel family of hTRPM8 agonists and antagonists and signal to α‐acyloxy carboxamide **23** as a potent TRPM8 antagonist with potential therapeutic value.

We next evaluated the α‐aminoacyl amides **30–41** exploiting the Ugi reaction (Table 1). We also found a mixture of agonists and antagonists within this family of compounds, although more enriched in agonists. Hence, compounds **33** and **35** displayed moderate antagonistic activity with IC_50_ in the low micromolar range. Compound **33** is closely related to compound **23**, indicating that the substitution of the ester by an amide did not drastically alter the activity of the compound, substantiating the importance for antagonistic activity of the bulky aromatic rings. Additional support is provided by compound **35**. All other α‐aminoacyl amides exhibited agonistic activity with potencies ranging from 0.2 to 5 μm. Again, the substitution of the ester by an amide modestly affected the potency of most compounds (see **22** with **34**, **37**, **39**, and **40**). A significant increase in potency was observed for compound **31** (EC_50_ = 0.3 ± 1.9 μm) as compared with the ester‐containing counterpart **24** (EC_50_ = 19 ± 2.0 μm). Most notably, at variance with the antagonistic activity of the α‐acyloxy carboxamide **25** (IC_50_ = 45 ± 2.0 μm), the α‐aminoacyl amide **32** acted as a potent hTRPM8 agonist (EC_50_ = 0.4 ± 1.7 μm).

Last, functional evaluation of the four α‐acylpiperazino amides (**42–45**) revealed an agonistic activity on hTRPM8 channels, with lower micromolar potency than the α‐acyloxy carboxamide and α‐aminoacyl amides (Table 1). To highlight specific comparisons: compound **42** can be directly compared to compound **30**; compound **44** to compound **36**; and compound **45** to compounds **22**, **34**, **37**, **39**, and **40**. Akin to α‐aminoacyl amide **32**, the α‐acylpiperazino amide **43** also behaves as a TRPM8 agonist although with > 10‐fold lower potency.

### α‐Acyloxy carboxamide 23 is a potent hTRPM8 competitive WS‐12 antagonist

**Fig. 3:**
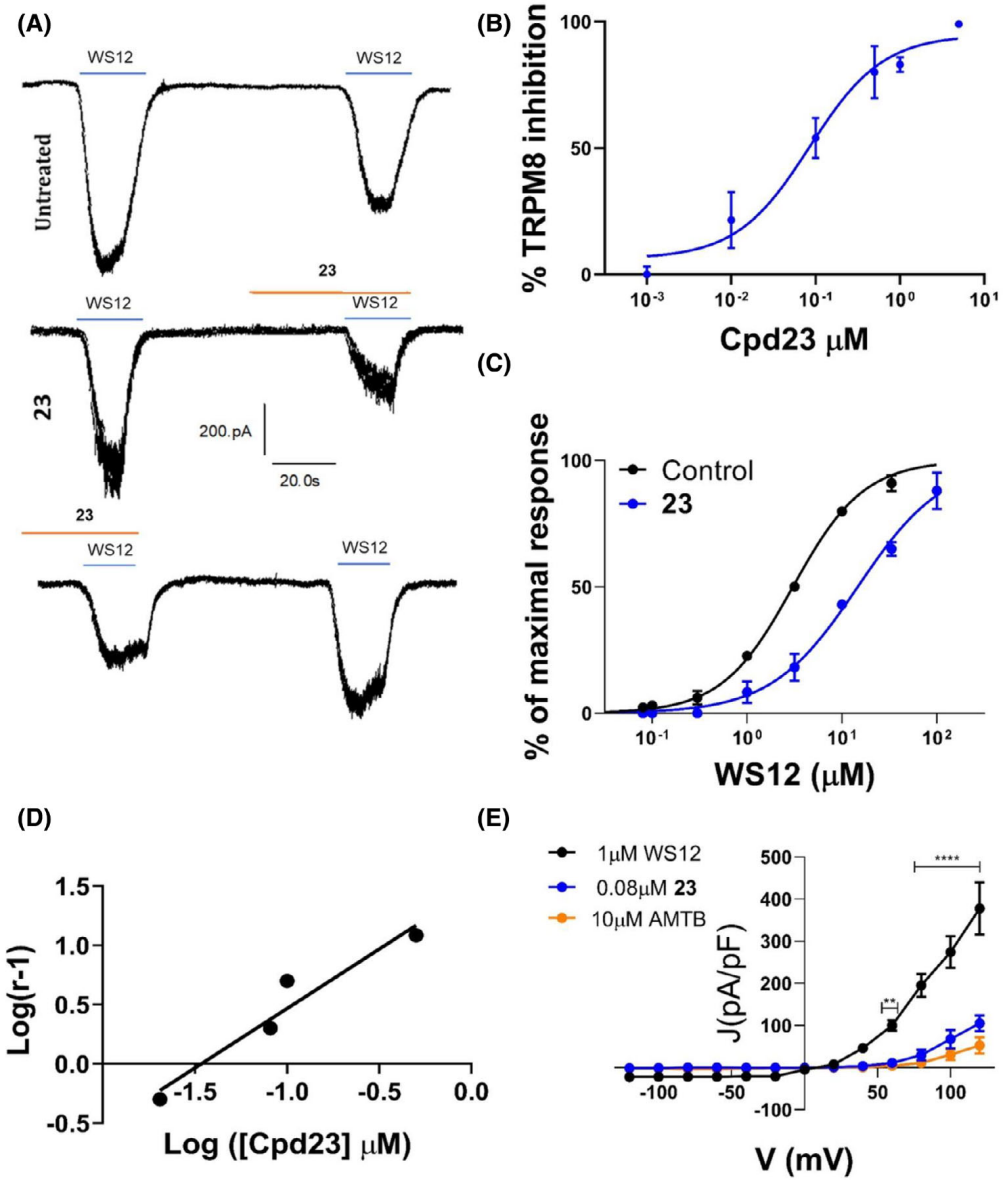
Compound **23** is a hTRPM8 competitive blocker with nanomolar potency. (A) Representative traces of WS‐12‐evoked ionic currents recorded at a holding potential of −60 mV in HEK293 cells heterologously expressing hTRPM8. Control cells (upper panel) were exposed to two pulses (1 μm) of WS‐12, interspaced by a washing period whereas compound **23**‐treated cells (middle panel) were exposed before to 1 min of compound **23** (1 μm) and then to 30s of compound **23** during the second pulse of WS‐12. In the lower panel, the recovery of WS‐12‐evoked currents after compound **23** treatment is shown, indicating a reversible effect of the compound on hTRPM8 activity. (B) Dose–response curve for hTRPM8 current blockade at a holding voltage of −60 mV. The line represents the fit of the experimental data to the Hill equation: $Y=\text{Bottom}+\left(\mathrm{Top}‐\text{Bottom}\right)/\left(1+10^{\left(X-{\text{LogIC}}_{50}\right)}\right)$ with a standard slope of 1.0 (Hill coefficient) and a restriction Top (= 100). The fitted value for IC_50_ was 0.08 ± 0.07 μm. Each point is the mean ± SEM of *N* = 3, *n* = 10. (C) WS‐12 dose–response curve in the absence (black) or presence of 0.08 μm compound **23** (blue). The best fitted values for WS‐12 EC_50_ value were 3.0 μm (95% CI 2.5–3.68 μm) (*n* = 18) in the absence of 0.1 μm compound **23** and 15 μm (95% CI 12.8–16.8 μm) in its presence (blue curve, *n* = 16, Top = 100). Hill coefficient in the absence of compound **23** was 1.17 (0.9–1.4) and in its presence was 0.95 (95% CI 0.8–1.0). All data are expressed as mean ± SEM. *N* = 3. (D) Schild plot derived from the interaction between agonist (WS12) and antagonist (compound **23**) at four different concentrations: 0.02, 0.08, 0.1, 0.5 μm. Equation: *Y* = 1003 × *X* + 1469; Slope: 1.003; 1/Slope: 0.9973; Std. error: 0.1935. *R* square: 0.9307; *P* value: 0.0353. *N* = 3, *n* = 10. (E) Representative current density (*J*)‐*V* curves elicited by a protocol of voltage steps from −120 to 120 mV in steps of 20 mV in the absence (control) or presence of 0.08 μm compound **23** (*n* = 10) or 10 μm AMTB (*n* = 8). In the control condition, an additional pulse of WS‐12 was used to consider channel desensitization. Data were analyzed with two‐way ANOVA, *N* = 3. Each point is the mean ± SEM. Data were analyzed using a two‐way ANOVA followed by a Sidak *post hoc* test when appropriate, **P value = 0.0069; ****P value < 0.0001.

We selected α‐acyloxy carboxamide **23** as the most potent antagonist (IC_50_ = 0.2 ± 1.3 μm) for further *in vitro* and *in vivo* characterization. We first used patch‐clamp to investigate the potency and blockade mechanism of compound **23** inhibiting WS‐12 evoked ionic currents in hTRPM8‐HEK293 cells. As shown in Fig. 3A, 1 μm of α‐acyloxy carboxamide **23** reduced ≥ 75% WS‐12 evoked inward current. This blockade activity was reversible as virtually all WS‐12‐evoked ionic current could be recovered upon washout (Fig. 3A, bottom panel), thus preventing a use‐dependent side effect. A dose–response curve revealed an IC_50_ of 0.08 ± 0.07 μm, and a Hill coefficient of 1 (Fig. 3B), suggesting that compound **23** is a potent hTRPM8 antagonist.

To unveil the underlying mechanism of channel blockade (competitive vs noncompetitive) we evaluated the effect of the α‐acyloxy carboxamide **23** on WS‐12 activating potency. For this purpose, we obtained the WS‐12 dose response in the absence and presence of 80 nm compound **23** (corresponding to its IC_50_ value). As depicted in Fig. 3C, the WS‐12 dose response was right‐shifted to higher concentrations in the presence of compound **23** increasing up to fivefold the WS‐12 EC_50_ value from 3 to 15 μm, without altering the agonist efficacy, suggesting a competitive blockade mechanism. In support of this tenet, a Schild plot to evaluate the effect of increasing concentrations of compound **23** in the blockade efficacy of WS‐12 revealed a linear relationship (slope of 1.0) consistent with a competitive inhibitory mechanism (Fig. 3D). Thus, these results suggest that α‐acyloxy carboxamide **23** and WS‐12 binding to the same or nearby site in the receptor.

To further substantiate the competitive mechanism of channel blockade, we examined the voltage dependency of compound **23** antagonistic activity (Fig. 3E). For this purpose, we run 100 ms voltage ramps from −120 to 120 mV activated by WS‐12 in the absence and presence of 80 nm α‐acyloxy carboxamide **23**. Figure 3E shows the current density (*J*)‐*V* relationships for compound **23** and the well‐known TRPM8 competitive antagonist **AMTB**. Both compounds inhibited WS‐12 in the entire range of depolarizing voltages, indicating channel blockade was not voltage dependent as it would be expected for an uncompetitive antagonist. Thus, these data further support a competitive, voltage‐independent blockade mechanism for compound **23**.

### Compound 23 marginally blocks TRPV1 and TRPA1 activity

**Fig. 4:**
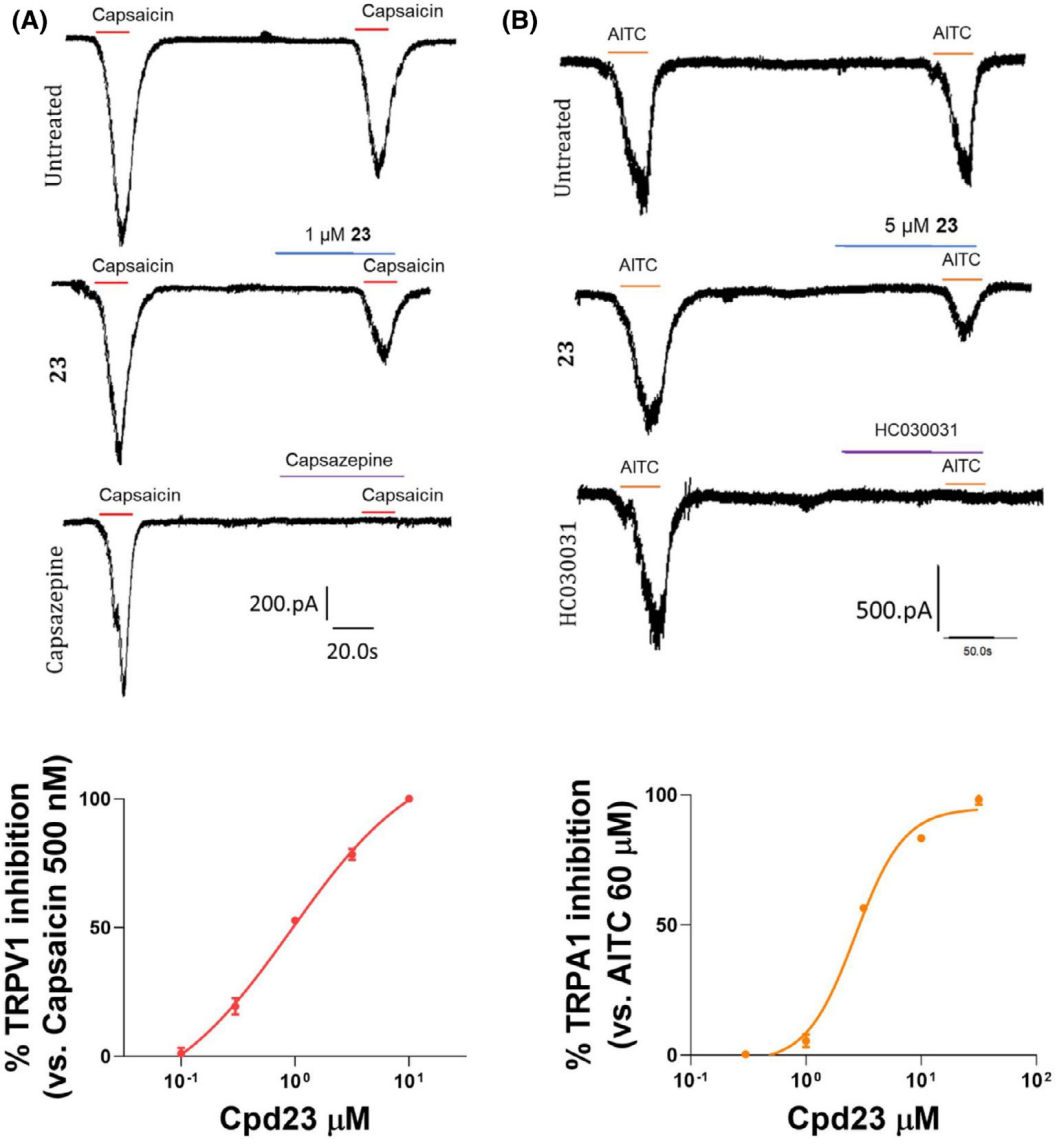
Compound **23** modestly inhibits currents of hTRPV1 and hTRPA1 expressed in HEK293 cells. (A) Representative capsaicin (0.5 μm)‐evoked hTRPV1 inward currents recorded at a holding potential of −60 mV, for control cells (untreated) and cells treated with 1 μm compound **23** or 10 μm of Capsazepine. Red lines represent the duration of the capsaicin pulse (top panel). Dose–response curve for hTRPV1 current blockade at a holding voltage of −60 mV. The line represents the fitted curve of the experimental data to the Hill equation. The fitted value for IC_50_ was 1 μm ± 1.02. Each point is the mean ± SEM of *n* = 6 (bottom panel). (B) Representative AITC (60 μm) evoked hTRPA1 inward current recorded at a holding potential of −60 mV, for control cells (untreated) and cells treated with 5 μm compound **23** or 5 μm of HC030031. Orange lines represent the duration of the AITC pulse (top panel). Dose–response curve for hTRPA1 current blockade at a holding voltage of −60 mV. The line represents fits of the experimental data to the Hill equation. The fitted value for IC_50_ was 3.04 μm ± 2.01. Each point is the mean ± SEM of *n* = 8 (bottom panel).

To evaluate receptor selectivity, we investigated the activity of compound **23** on recombinant hTRPV1 and hTRPA1 channels recombinantly expressed in HEK293 cells (Fig. 4). We choose these thermosensory channels because of their structural similarity and expression in peripheral nociceptive terminals. In addition, some ligand cross‐interaction within these channels has been reported [44]. At 1 μm, compound **23** blocked ≈ 50% of hTRPV1‐channel activity (Fig. 4A, top panel), showing an IC_50_ of 1.1 ± 1.02 μm (Fig. 4A, bottom panel). Similarly, at 5 μm compound **23** blocked ≈ 60% of hTRPA1‐evoked current (Fig. 4B, top panel), exhibiting an IC_50_ of 3.04 ± 2.01 μm (Fig. 4B, bottom panel). For comparison, 2‐(1,3‐Dimethyl‐2,6‐dioxo‐1,2,3,6‐tetrahydro‐7H‐purin‐7‐yl)‐*N*‐(4‐isopropylphenyl)acetamide (HC030031), a reference TRPA1 antagonist [45], completely abrogated AITC‐evoked ionic currents at 5 μm (Fig. 4B, top panel). Collectively, these data indicate that α‐acyloxy carboxamide **23** is ≥ 10‐fold more potent blocking hTRPM8 than hTRPV1 and hTRPA1 channels, denoting a preferential selectivity for hTRPM8.

### Modeling of α‐acyloxy carboxamide 23 docking in the menthol WS‐12 site of the hTRPM8 channel

**Fig. 5:**
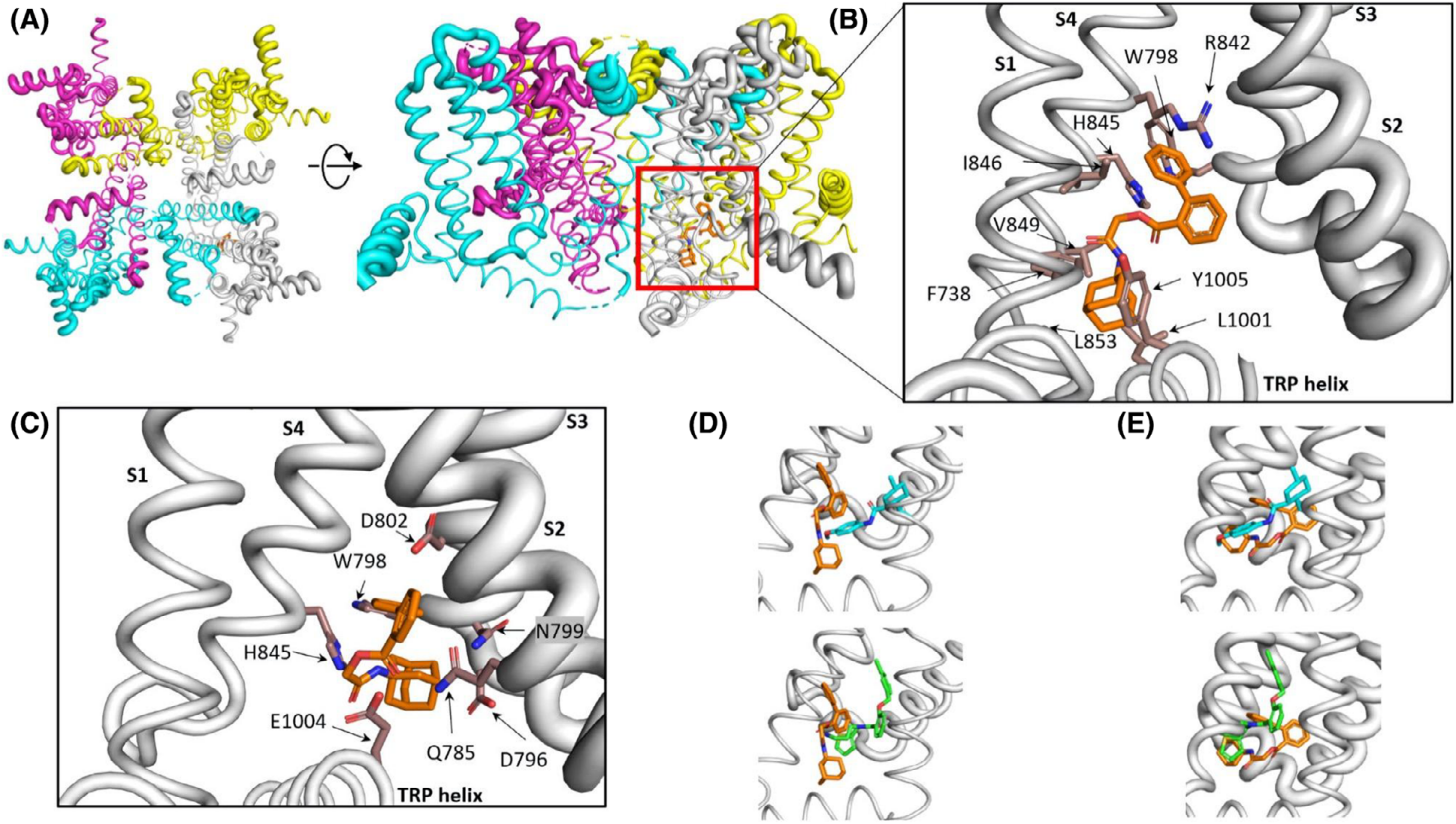
Molecular docking of compound **23** in TRPM8 channels. (A) Side and top view of human TRPM8 structure (PDB ID: 8BDC) used for docking studies. Subunits are colored differently. The tetrameric nature of the channel and the pore are clearly seen. The red square roughly indicates the simulation box built around the menthol binding site to accommodate compound **23**. (B) Detail of the human TRPM8 menthol binding pocket with bound compound **23** (orange color). Residues mainly involved in ligand interactions were F738 (S1), W798 (S3), R842, H845, I846, V849, L853 (S4), and L1001, Y1005 (TRP helix). Interactions are mainly hydrophobic, although a salt bridge and π‐cation interactions are also observed. (C) Detail of compound **23** bound to the mouse TRPM8 binding pocket (equivalent view to panel B). Residues interacting with compound **23** (orange) were Q785 (S2), D796, W798, N799, D802 (S3), H845 (S4), and E1004 (TRP helix). (D) Compound **23** (orange) and WS‐12 (cyan) or AMTB (green) superimposed in the human TRPM8 menthol binding site for comparison. (E) Compound **23** (orange) and WS‐12 (cyan) or AMTB (green) superimposed in the mouse TRPM8 menthol binding site for comparison. Figures were constructed using open‐source pymol v3.0 (https://pymol.org/).

To further support the blocking mechanism and selectivity, we performed molecular docking of compound **23** on the hTRPM8 channel (Fig. 5). Notably, compound **23** readily accommodates into the menthol binding pocket located between the S1, S2, S3, and S4 transmembrane domains in the closed conformation (Fig. 5A,B). The biphenyl group of compound **23** interacts with residues R842, H845, and I846 in S4, and W798 in S3 of hTRPM8. Similarly, the adamantane group contacts V849 and L853 in S4, F738 in S1, and L1001 and Y1005 in the TRP helix. Additional interactions are a salt bridge with H845 and two π‐cation involving R842 and H845 in S4 (Fig. 5C).

The superimposition of compound **23**, **WS‐12**, and **AMTB** bound to the receptor menthol binding pocket (Fig. 5D) shows that compound **23** partially occupies the site of **WS‐12**, consistent with the observed competitive mechanism of channel blockade. The estimated binding energies to the hTRPM8 receptor reveal that the largest value corresponds to compound **23** (−10.41 kcal·mol^−1^ vs. −9.93 kcal·mol^−1^ for AMTB and −7.72 kcal·mol^−1^ for WS‐12). The higher binding energy of compound **23** as compared to WS‐12 may be due to the polar and hydrophobic contacts with residues located mainly in S4 and the TRP helix that involve both the biphenyl and the adamantane groups of compound **23**.

For comparison, we also modeled the interaction of compound **23** with the menthol binding site of mouse TRPM8 (mTRPM8, Fig. 5E). Docking of compound **23** into this binding site appears to involve slightly different interactions than those observed in the human ortholog. At variance with hTRPM8, where compound **23** accommodates into a faintly polar environment with the biphenyl and the adamantane groups closer to S2 and S3 transmembrane domains, in the murine TRPM8 compound **23** interacts with Q785 (S2), D796, W798, N799, D802 in S3, H845 in S4, and E1004 in the TRP helix. The reduced hydrophobic interactions noticed in the mTRPM8 binding site may underlie the lower binding energy estimated (−9.96 kcal·mol^−1^) as compared to the human ortholog. This lower binding energy suggests a lower blocking potency of compound **23** blocking mTRPM8.

### Compound 23 blocks neuronal TRPM8 and modulates WS‐12‐induced neuronal firing

**Fig. 6:**
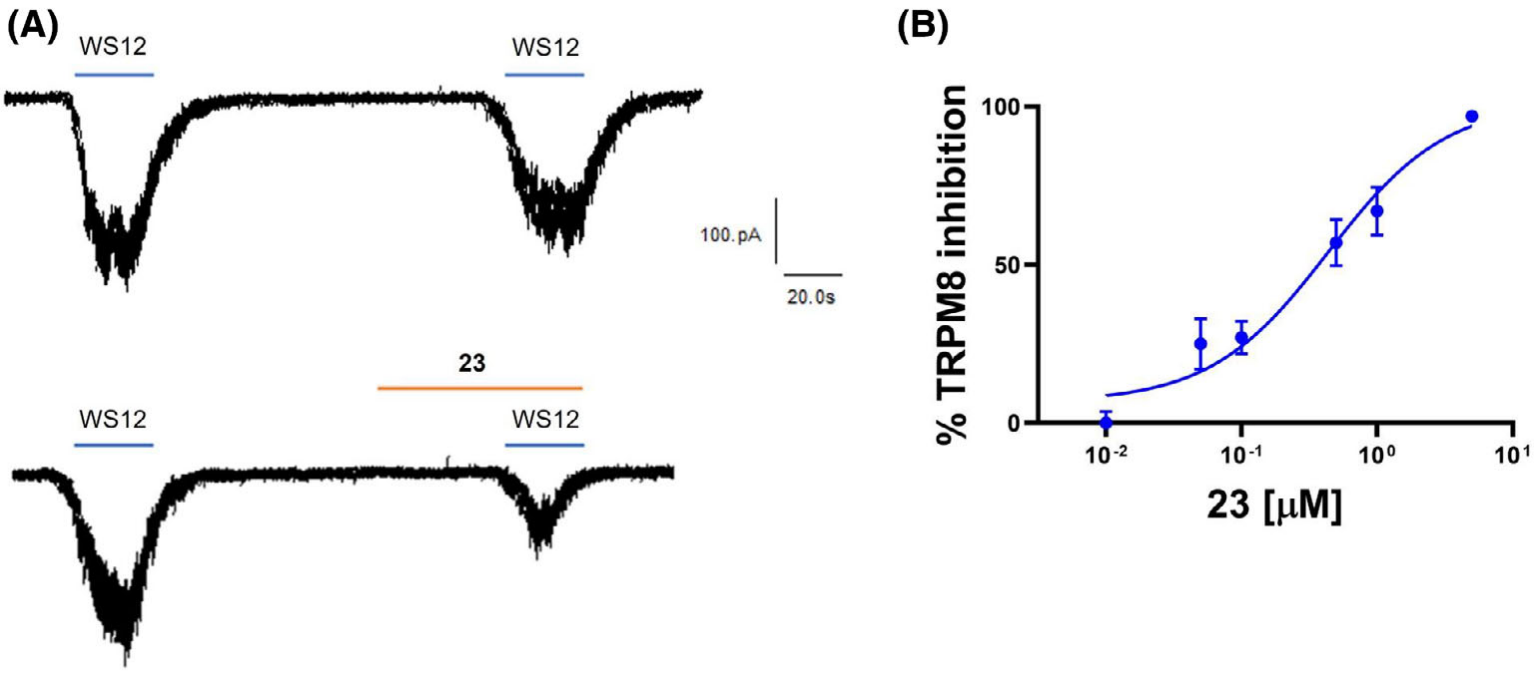
Compound **23** blocks WS‐12‐induced TRPM8 activation in murine nociceptors. (A) Representative WS‐12‐evoked currents in DRG neurons of neonatal rats recorded at a holding potential of −60 mV. For control conditions (untreated) cells were exposed to two pulses (1 μm) of WS‐12, interspaced by a washing period, whereas treated cells were exposed before to 1 min of compound **23** (1 μm) and then to 30 s of compound **23** during the second pulse of WS‐12. (B) Dose–response curve for mTRPM8 current blockade at a holding voltage of −60 mV. The line represents fits of the experimental data to the Hill equation. The fitted value for IC_50_ was 0.43 μm ± 0.75. Each point is the mean ± SEM of *N* = 3, *n* = 8.

To examine the *in vivo* activity of compound **23**, we first investigated its inhibitory potency on neuronal TRPM8 channels expressed in murine primary nociceptor cultures by patch‐clamp. As seen in Fig. 6A, 5 μm of compound **23** blocked 60% of WS‐12‐evoked ionic currents in primary cultures of dorsal root ganglion neurons. A dose–response curve revealed an IC_50_ of 0.43 ± 0.75 μm (Fig. 6B). This result indicates a lower potency of compound **23** inhibiting the murine TRPM8 as compared with the human ortholog consistent with the predicted lower binding energy to mTRPM8. Alternatively, it could also be due to distinct gating mechanisms of both orthologs or the influence of the neuronal environment.

**Fig. 7:**
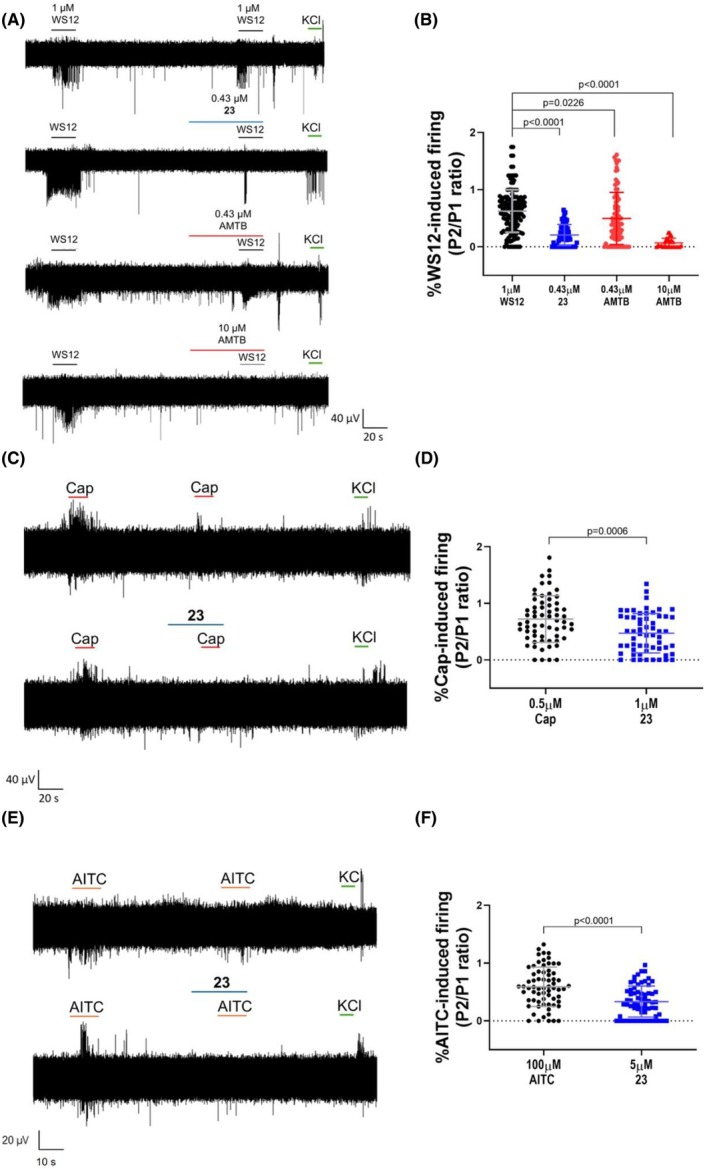
Compound **23** reduces WS‐12‐induced excitability of murine nociceptors. (A) Multielectrode array (MEA) recordings, with representative traces showing WS‐12‐evoked action potential (AP) firing under different conditions: control, 0.43 μm compound **23**, 0.43 μm AMTB, and 10 μm AMTB. WS‐12 (1 μm) was applied in two consecutive pulses, with compounds added 1 min before and during the second pulse. The protocol concluded with a 15 s pulse of 40 mm KCl to ensure neuronal viability. (B) Normalized WS12‐induced firing (P2/P1) in the absence (vehicle) and presence of antagonists (0.43 μm compound **23**, 0.43 μm AMTB and 10 μm AMTB) were compared. Data were analyzed with one‐way ANOVA followed by Dunnett's test; *P* values for statistical differences are indicated. The number of independent experiments (*N*) was 3, with 94 electrodes per condition. The data points are plotted with error bars representing the standard deviation (SD). (C) Multielectrode array (MEA) recordings, with representative traces showing Cap‐evoked action potential (AP) firing under different conditions: control and 1 μm compound **23**. Cap (0.5 μm) was applied in two consecutive pulses, with compounds added 1 min before and during the second pulse. The protocol concluded with a 15 s pulse of 40 mm KCl to ensure neuronal culture viability. (D) Normalized capsaicin‐induced firing (P2/P1) in the absence (vehicle) and presence of antagonist (1 μm compound **23**) were compared. The data points are plotted with error bars representing the standard deviation (SD). The number of independent experiments (*N*) was 3, with 63 electrodes per condition. Data were analyzed with Mann–Whitney test; *P* values for statistical differences are indicated. (E) Representative traces showing AITC‐evoked action potential (AP) firing under different conditions: control and 5 μm compound **23**. AITC (100 μm) was applied in two consecutive pulses, with compounds added 1 min before and during the second pulse. The protocol concluded with a 15 s pulse of 40 mm KCl to ensure neuronal culture viability. (F) Normalized AITC‐induced firing (P2/P1) in the absence (vehicle) and presence of antagonist (5 μm compound **23**) were compared. Data were analyzed with Mann–Whitney test; *P* values for statistical differences are indicated. The data points are plotted with error bars representing the standard deviation (SD). The number of independent experiments (*N*) was 3, with 67 electrodes per condition.

Complementarily, we examined the effect of compound **23** on WS‐12‐evoked action potentials in primary cultures of murine DRG sensory neurons. For this task, we recorded neuronal excitability using multielectrode arrays (MEAs). The IC_50_ value corresponding to 0.43 μm was used to examine compound **23** induced inhibition of neuronal excitability. A protocol of two sequential pulses of WS‐12 interspersed with a washing pulse was applied to correct for channel desensitization (Fig. 7A, upper recording). Compounds **23** and **AMTB** (0.43 and 10 μm, respectively) were applied 1 min before and during the second WS‐12 pulse (Fig. 7A, second, third, and fourth recordings). As depicted in Fig. 7B, compound **23** virtually abolished action potential firings induced by WS‐12 application in murine primary afferent neurons (≥ 75%). Thus, compound **23** appears to be a potent inhibitor of WS12‐evoked firing frequency, even surpassing the efficacy of AMTB at equimolar concentrations (Fig. 7B).

Additionally, we investigated the effect of compound **23** on capsaicin and AITC‐induced excitability in murine nociceptors. Employing an IC_50_ value of 1 μm for TRPV1 and 5 μm for TRPA1 of firing inhibition by compound **23** (Fig. 4), we interrogated its ability to modulate neuronal excitability. As for TRPM8, our experimental design involved a protocol of dual agonist pulses interspersed with a washing pulse to minimize agonist‐induced channel desensitization. Compound **23** was administered 1 min prior to and during the second agonist pulse. Figure 7C–F show that compound **23** attenuated capsaicin and AITC responses by 30% and 40%, respectively, consistent with a more potent blockade of TRPM8 channels and a mild cross‐reactivity with TRPV1 and TRPA1 akin to other modulators of this channel family [46]. Collectively, these results indicate that α‐acyloxy carboxamide **23** is a novel TRPM8 antagonist that potently and selectively blocks TRPM8 channel activity and reduces neuronal excitability induced by receptor agonists.

### Local application of compound 23 reduces oxaliplatin‐induced peripheral cold allodynia

**Fig. 8:**
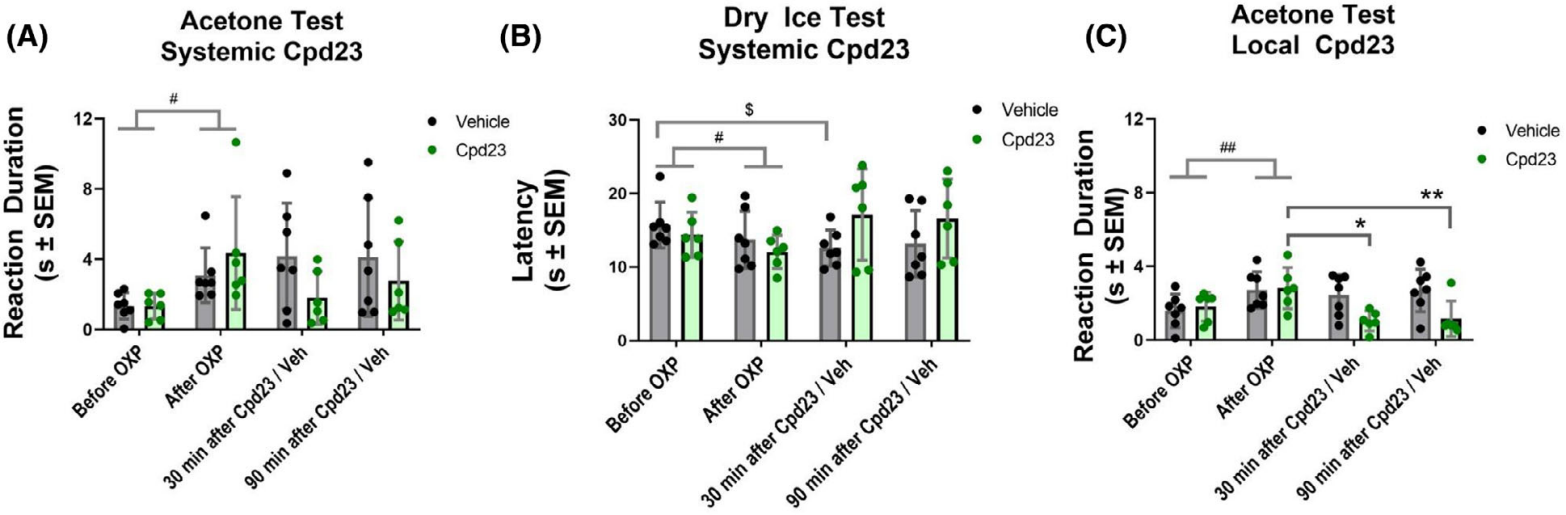
Local application of **23** alleviates oxaliplatin‐induced sensitivity to cold. (A) Repeated administration of oxaliplatin increased the duration of responses to cold induced by acetone application (A, ^#^ *P* < 0.05 Before vs After OXP, two‐way ANOVA). However, no significant effect of systemic **23** was found 30 or 90 min after its intraperitoneal (i.p.) administration. (B) Oxaliplatin induced a significant decrease in the withdrawal latency to cold induced by dry ice application (B, ^#^ *P* < 0.05 Before vs After OXP, two‐way ANOVA). Vehicle‐treated mice retained significant cold sensitization after i.p. treatment (B, ^$,#^ *P* < 0.05, Before OXP vs 30 min after Vehicle, two‐way ANOVA), whereas this difference was not evident in **23**‐treated mice. However, **23** did not exhibit cold antinociceptive effects following systemic treatment. (C) Oxaliplatin increased the duration of the responses to acetone‐induced cold (C, ^##^ *P* < 0.01 Before vs After OXP, two‐way ANOVA) and **23** alleviated oxaliplatin‐induced cold sensitivity 30 and 90 min after its local subcutaneous administration in the paw (i.pl., *P < 0.05 After OXP vs. 30 min after **23**, **P < 0.01 After OXP vs. 90 min after **23**). Two‐way ANOVA followed by Tukey *post hoc* test when appropriate. Bars represent average values and error bars represent SEM. Dots are the individual values of each mouse (*N* = 6–7).

Given the inhibitory activity of compound **23** and the antinociceptive effects of TRPM8 antagonists described in models of oxaliplatin (OXP)‐induced neuropathy [47, 48, 49], we conducted a behavioral experiment in mice to assess the antinociceptive effects of compound **23** after repeated oxaliplatin administration. Oxaliplatin was intraperitoneally (i.p.) administered every other day for 5 days at 6 mg·kg^−1^, reaching a total accumulative dose of 18 mg·kg^−1^. The acetone test, based on the duration of the licking response after the application of acetone drops to the hind paws (Fig. 8A–C, Before OXP vs. After OXP), revealed a significant enhancement in the nociceptive response to cold after OXP treatment (Fig. 8A–C, *P* < 0.01 Before OXP vs. After OXP). The OXP‐induced sensitization to cold stimuli was also evident with the dry ice test, as evidenced by the significant reduction in the latency to cold stimulation through the application of a dry ice pellet against the paw on a glass surface (Fig. 8B, *P* < 0.05 Before OXP vs. After OXP).

We next investigated the antinociceptive effect of systemic compound **23** (5 mg·kg^−1^ i.p.) as compared to its vehicle in both the acetone and the dry ice tests. Measurements were conducted 30 and 90 min after compound **23** or vehicle i.p. administration. As shown in Fig. 8A,B, instillation of compound **23** did not significantly affect the licking response to acetone drops nor the withdrawal latencies to dry ice application as compared to vehicle. This unexpected lack of antinociceptive activity may be because of poor pharmacokinetic due to the metabolic instability of the ester bond that is susceptible to esterase hydrolysis.

To circumvent the potential systemic metabolic instability, we investigated if a local application in the animal paw produced antinociceptive activity. As illustrated in Fig. 8C, local subcutaneous application of compound **23** produced a significant antinociceptive effect in the acetone test, evident at 30 and 90 min after compound administration (*P* < 0.05 at 30 min, *P* < 0.01 at 90 min, After OXP vs. 30 and 90 min after compound **23**). Collectively, these results suggest that α‐acyloxy carboxamide **23** exhibits a significant antinociceptive effect following local application to the peripheral endings, substantiating that systemic treatment is limited by a poor pharmacokinetics of the antagonist.

### Compound 23 exhibits mild metabolic stability

Because of the lack of systemic activity of compound **23**, we evaluated the *in vitro* plasmatic and hepatic metabolic stability. To this aim, compound **23** was exposed to mouse plasma and mouse liver microsome (MLM) fractions, and the residual substrate was measured after 1 h. Under these conditions, the residual substrate was about 97% and 90% in plasma and microsomes, respectively, denoting that this molecule suffers from mild hydrolysis of the ester group. However, when the microsomal monooxygenase system was activated by the NADPH regenerating system, the residual substrate dropped about 42% suggesting a susceptibility towards oxidative metabolism.

**Fig. 9:**
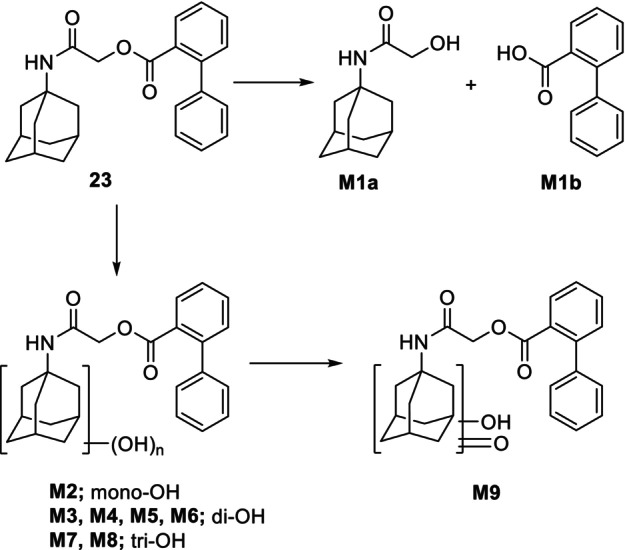
Proposed *in vitro* phase I metabolic scheme for the compound **23**.

Metabolic biotransformation of compound **23** was investigated by tandem high‐resolution mass spectrometry using the software compound discoverer 3.2™. Alongside enzymatic hydrolysis giving M1a and M1b metabolites, mono and multi‐aliphatic oxidation was the predominant biotransformation occurring on the adamantane ring M2–M9 (Table 2; Fig. 9) and (Tables S1 and S2, Figs S79 and S80 for full spectral data) also demonstrate by the relative abundancy of some of them estimated based on the total ion current (TIC) measurement. Notably, the susceptibility of the adamantane ring to undergo microsomal oxidation is consistent with previous published findings [50, 51]. Indeed, some synthetic cannabinoid mono‐, di‐, and even tri‐hydroxylate derivatives of the adamantane group were revealed after hepatic phase I biotransformation.

**Table 2:** Phase I metabolites of the compound **23** incubated in mouse liver microsomes and detected by LC‐HRMS analysis.

	Retention time (min)	Theoretical [M + H]^+^	Measured [M + H]^+^	Δ Da p.p.m.	Metabolite relative abundancy %
**23**	10.71	390.20637	390.20584	1.36	–
**M1a**	4.87	210.14886	210.14871	0.71	18.7
**M1b**	6.07	199.07536	199.07563	1.36	n.d.^a^
**M2**	6.94	406.20129	406.20118	0.27	11.5
**M3**	4.42	422.19620	422.19601	0.45	14.2
**M4**	4.82	422.19620	422.19602	0.43	1.5
**M5**	5.34	422.19620	422.19598	0.52	6.2
**M6**	5.67	422.19620	422.19577	1.02	12.8
**M7**	3.36	438.19111	438.19092	0.43	4.5
**M8**	4.20	438.19111	438.19077	0.78	29.0
**M9**	7.02	420.18055	420.18018	0.88	1.5

^a^ Not determined due to poor ionization in positive ion modality.

## Discussion

Cumulative evidence supports a role of TRPM8 in transducing both innocuous and harmful cold thermal signals under physiological and pathological conditions [52, 53, 54]. Accordingly, TRPM8 is considered a pivotal clinical target, and the development of therapeutically useful modulators is a significant pharmaceutical and medical unmet goal. Akin to TRPV1 antagonists, most of systemic TRPM8 modulators tested have failed clinical development due to safety concerns. However, topical formulations of TRPM8 modulators directed to attenuate receptor pathological overactivity in conditions such as dry eye syndrome [14] or pruritus [23] have reported promising results. In this context, the salient contribution of this study is the design of hTRPM8 ligands with potential therapeutic properties based on multicomponent reactions such as the Ugi and Passerini reactions that capitalize on the drug‐like properties of the adamantane group. These reactions enable the rapid assembly of focused chemical libraries using green chemistry criteria [40]. Although medicinal chemists have extensively utilized the Ugi reaction in drug discovery [32], the Passerini reaction has been less popular. This reluctance stems from the apprehension of medicinal chemists towards esters, which are considered enzymatically unstable and unsuitable for systemic drug administration. In contrast to this belief, we have demonstrated that depending on the nature of the carboxylic acid utilized, Passerini adducts can be obtained that are resistant to plasma and hepatic esterases [55]. Nonetheless, these ester‐containing soft compounds have been shown useful in improving the safety of topically acting vanilloid‐based soft antagonists [56].

Among all compounds tested, α‐acyloxy carboxamide **23** stems as a potent TRPM8 competitive antagonist, substantiated by Schild plot analysis. Furthermore, compound **23** exhibited high blockade potency (IC_50_ of 80 nm) and efficacy (100% response block). Structurally, α‐acyloxy carboxamide **23** displays a bulky moiety that readily fits into the menthol binding site of the receptor stabilizing the channel in the closed state, most likely by restricting the movement of the S4 and TRP domains necessary to drive the allosteric conformation that leads to the opening of the inner channel gate [57]. Computational docking studies show the potential binding interactions of compound **23** to hTRPM8 and mTRPM8 orthologs and provide evidence for the lower blockade potency on the murine channel. These differences appear to be mediated by a slightly different orientation of the compound within the receptor binding site that impacts the binding energy and consequently the blockade potency. Interestingly, the capacity of compound **23** to effectively bind to the channel closed state is compatible with a competitive blockade mechanism, as this site is readily accessible for agonists to bind and trigger the allosteric conformation to the open state. It is also tempting to hypothesize that occupancy of a binding site of the four available in the native tetrameric channel may suffice to prevent channel opening by the agonist, as suggested by the estimated hill coefficient from the dose–response curves. In this regard, it has been reported that efficient opening of TRPM8 channels requires occupancy of the four binding sites [58] thus interference with a single binding site by antagonists may suffice to lock the channel in the closed state. Nonetheless, additional experiments are needed to unveil the structural determinants of compound **23** binding, including site‐specific mutagenesis, along with biophysical analysis of its blockade activity.

Fast unblocking kinetics of α‐acyloxy carboxamide **23** unblock, that is, fast *k*_off_, ensures reversible activity, which is important to reduce a use‐dependent channel block. This property is associated with high‐affinity antagonists exhibiting a slow unblockage kinetics, which limits their therapeutic utility because of undesirable side effects, as exemplified by the high affinity of TRPV1 blockers [59] or the NMDA blocker MK‐801 [60]. In this regard, compounds endowed of fast on/off kinetics appear to be safer for therapeutic applications. As TRPM8 is a physiologically relevant channel transducing environmental temperature detection for proper body temperature homeostasis and prevention of tissue damage, preservation of this physiological activity is crucial to prevent alterations in body temperature unwanted effects described with previous antagonists including hot sensation, gut motility deficits, and cold burns [61, 62]. Absence of use‐dependent block minimizes the manifestation of unwanted side effects, increasing the safety profile of drug candidates.

A complementary property of α‐acyloxy carboxamide **23** is represented by its channel selectivity, as evidenced by its preferential action on TRPM8 channels, and lower interaction with structurally related thermosensory channels such as TRPV1 and TRPA1, which are also highly expressed in nociceptive neurons and contribute to the etiology of peripheral neuropathies. Interestingly, cross‐reactivity of agonists and antagonists of these thermosensory channels has been reported [46]. For instance, an interaction of capsaicin with TRPM8 and menthol with TRPV1 and TRPA1 channels has been described [46]. This cross‐recognition is due to conserved similarities between the receptor membrane domain structuring the ligand binding sites, which have evolved to preferentially recognize one ligand but conserve the essential features to be mildly recognized by others.

Drug‐off target effects may raise safety concerns in contrast to highly selective drugs. However, it should be noted that although drug selectivity has been considered a pivotal goal for therapeutic leads and long pursued by medicinal chemistry programs, it is being realized that some cross‐reactivity of drugs with structurally similar receptors may enhance their therapeutic value as peripheral neuropathies involve the participation of various thermosensory receptors.

Absence of use‐dependency also in off‐target interactions minimizes the appearance of side effects that could compromise the therapeutic index. Furthermore, a multitarget cross‐activity may be beneficial in cancer patients treated with chemotherapy that develop a distal peripheral neuropathy, where a contribution of TRPM8, TRPV1, and TRPA1 channels has been reported [63]. The possibility of differentially targeting all three channels with a single compound could be a promising therapeutic strategy to target this disease. In support of this tenet, local application of α‐acyloxy carboxamide **23** exhibited significant antinociceptive activity attenuating OIPN‐induced cold allodynia.

A systemic administration of compound **23**, however, failed to reduce the cold allodynia that accompanies OIPN. This lack of therapeutic effect is most likely due to a low *in vivo* metabolic stability of compound **23** that resulted in poor pharmacokinetics. In this regard, although the bulky aromatic groups present in the molecule slowed the hydrolysis when exposed to plasma and microsomal fractions, it did not fully prevent the breakdown of the ester under oxidative metabolism when the microsomal monooxygenase system was activated. Though this metabolic instability may be seen as a therapeutic limitation, metabolically sensitive soft drugs are ideal candidates for local applications whereby the therapeutic activity needs to be peripherally restricted to avoid potential interference with a pharmacological treatment, such as in cancer patients suffering OIPN. In addition, this lack of systemic distribution helps to reduce any potential off‐target effect on physiologically acting thermosensory channels in other tissues, which would raise safety concerns. Thus, drugs that act epidermally and are enzymatically hydrolyzed at the dermis appear relevant for therapeutic interventions of peripheral neuropathies. In this regard, the anti‐allodynic local activity of compound **23** paves the way to develop this compound for the topical treatment of this disabling sensory condition suffered by cancer patients.

In conclusion, our findings indicate that α‐acyloxy carboxamide **23** is a potential therapeutic TRPM8 antagonist that attenuates pathological cold allodynia arising from a common peripheral neuropathy in cancer patients. The *in vitro* pharmacological properties of compound **23**, that is, its potency and efficacy, reversible inhibition, receptor selectivity, lack of use‐dependent block, and relative peripheral stability, make it a potential drug candidate for local therapeutic intervention of peripheral neuropathies mediated by dysfunctional TRPM8 channels.

## Materials and methods

### Chemistry

#### General experimental methods

Commercially available reagents and solvents were purchased from Sigma‐Aldrich (Merck Life Science, Milan, Italy) or Alfa Aesar (Thermo Fisher Scientific, Monza, Italy) and were used without further purification. Melting points were determined in open glass capillary with a Stuart scientific SMP3 apparatus and are uncorrected. Infrared spectra were acquired with a FT‐IR Thermo‐Nicolet Avatar. ^1^H‐NMR and ^13^C‐NMR spectra were recorded on a JEOL ECP 300 MHz spectrometer (JEOL, Basiglio, Italy). Chemical shifts (δ) are reported in parts per million (p.p.m.) referenced to the residual solvent peak. The multiplicity of each signal is designated using the following abbreviations: s (singlet), d (doublet), t (triplet), q (quadruplet), quint (quintuplet), m (multiplet), br s (broad singlet), dd (doublet of doublets). Coupling constants (*J*) are reported in Hertz (Hz). High‐resolution ESI‐MS spectra were acquired on a Thermo Scientific Q‐Exactive™ Plus Hybrid Quadrupole‐Orbitrap™ mass spectrometer. The spectra were recorded by infusion into the ESI source using methanol as the solvent. Flash column chromatography was performed on silica gel (Merck Kieselgel 60, 230–400 mesh ASTM, Milan, Italy). Thin layer chromatography (TLC) was carried out on plates with a layer thickness of 0.25 mm (Merck Silica gel 60 F_254_); when necessary, they were developed with KMnO_4_ reagent or Dragendorff reagent. 1‐Isocyanoadamantane **3** was synthesized as described previously [43]. Compounds **1**, **4–14**, and **15–19** were commercially available. The purity of final compounds was ≥ 95% and was determined by high‐performance liquid chromatography coupled with an ultraviolet–visible detector using the instrumentation and methods reported in Figs S81–S92.

#### General procedure A for the synthesis of α‐acyloxy carboxamides **20–29**

To a solution of the corresponding carboxylic acid (0.62 mmol, 1 equiv) in dichloromethane (2 mL), 37% aqueous formaldehyde solution (2.48 mmol, 4 equiv) and 1‐isocyanoadamantane (**3**) (0.62 mmol, 1 equiv) were added. The reaction mixture was stirred at 25 °C for 18 h. After completion of the reaction, water was added, and the product was extracted with dichloromethane (x2). The combined organic layers were washed with aqueous saturated Na_2_CO_3_ solution (x1), brine (x1) and dried over sodium sulfate. After evaporation of the solvent under reduced pressure, the crude material was purified by flash column chromatography.

#### 2‐((3*S*,5*S*,7*S*)‐adamantan‐1‐ylamino)‐2‐oxoethyl benzoate (20)

The title compound was prepared from 1‐isocyanoadamantane (**3**), 37% aqueous formaldehyde solution, and benzoic acid (**4**) according to general procedure A. After extraction, the crude was purified by flash column chromatography using PE/EtOAc 9 : 1 as eluent to give a white solid; yield 83%; mp 155–157 °C; IR (KBr) $\tilde{\nu }$ 3275, 3090, 2916, 2853, 1727, 1657, 1271, 1129, 709 cm^−1^; ^1^H‐NMR (300 MHz, CDCl_3_) δ 8.05 (d, *J* = 8.3 Hz, 2H), 7.62 (t, *J* = 7.3 Hz, 1H), 7.49 (d, *J* = 7.5 Hz, 2H), 5.79 (br s, 1H), 4.70 (s, 2H), 2.09 (br s, 3H), 2.04 (br s, 6H), 1.68 (br s, 6H) p.p.m.; ^13^C‐NMR (75 MHz, CDCl_3_) δ 165.9, 165.2, 133.7, 129.8, 129.2, 128.8, 63.7, 52.2, 41.7, 36.3, 29.5 p.p.m.; HRMS (ESI^+^): *m/z* = 314.17464 [M + H]^+^; calcd. for C_19_H_23_O_3_N + H^+^: 314.17507.

#### 2‐((3*S*,5*S*,7*S*)‐adamantan‐1‐ylamino)‐2‐oxoethyl 2‐phenylacetate (21)

The title compound was prepared from 1‐isocyanoadamantane (**3**), 37% aqueous formaldehyde solution and phenylacetic acid (**5**) according to general procedure A. After extraction, the crude was purified by flash column chromatography using PE/EtOAc 8 : 2 as eluent to give a white solid; yield 84%; mp 78–80 °C; IR (KBr) $\tilde{\nu }$ 3292, 3065, 2911, 2851, 1750, 1671, 1544, 1234, 1134 cm^−1^; ^1^H‐NMR (300 MHz, CDCl_3_) δ 7.33–7.25 (m, 5H), 5.47 (br s, 1H), 4.39 (s, 2H), 3.66 (s, 2H), 1.99 (br s, 3H), 1.81 (br s, 6H), 1.60 (br s, 6H) p.p.m.; ^13^C‐NMR (75 MHz, CDCl_3_) δ 169.6, 165.6, 133.4, 129.1, 128.8, 127.4, 63.1, 51.8, 41.3, 41.2, 36.1, 29.3 p.p.m.; HRMS (ESI^+^): *m/z* = 328.19034 [M + H]^+^; calcd. for C_20_H_25_O_3_N + H^+^: 328.19072.

#### 2‐((3*S*,5*S*,7*S*)‐adamantan‐1‐ylamino)‐2‐oxoethyl acetate (22)

The title compound was prepared from 1‐isocyanoadamantane (**3**), 37% aqueous formaldehyde solution and acetic acid (**6**) according to general procedure A. After extraction, the product is obtained as a white solid; yield 91%; mp 102–104 °C; IR (KBr) $\tilde{\nu }$ 3292, 3077, 2909, 2849, 1745, 1662, 1558, 1225, 1095, 999, 605 cm^−1^; ^1^H‐NMR (300 MHz, CDCl_3_) δ 5.83 (br s, 1H), 4.31 (s, 2H), 2.03 (br s, 3H), 1.96 (br s, 3H), 1.90 (br s, 6H), 1.56 (br s, 6H) p.p.m.; ^13^C‐NMR (75 MHz, CDCl_3_) δ 169.3, 165.6, 63.0, 51.9, 41.3, 36.1, 29.2, 20.6 p.p.m.; HRMS (ESI^+^): *m/z* = 252.15916 [M + H]^+^; calcd. for C_14_H_21_O_3_N + H^+^: 252.15942.

#### 2‐((3*S*,5*S*,7*S*)‐adamantan‐1‐ylamino)‐2‐oxoethyl [1,1′‐biphenyl]‐2‐carboxylate (23)

The title compound was prepared from 1‐isocyanoadamantane (**3**), 37% aqueous formaldehyde solution and [1,1′‐biphenyl]‐2‐carboxylic acid (**7**) according to general procedure A. After extraction, the product is obtained as a colorless oil which taken up with methanol precipitated as a white solid, yield 89%; mp 53–55 °C; IR (KBr) $\tilde{\nu }$ 3059, 2908, 2850, 1734, 1685, 1533, 1273, 1132, 747 cm^−1^; ^1^H‐NMR (300 MHz, CDCl_3_) δ 7.83 (d, *J* = 7.7 Hz, 1H), 7.52 (t, *J* = 7.5 Hz, 1H), 7.42–7.27 (m, 7H), 5.27 (br s, 1H), 4.32 (s, 2H), 2.00 (br s, 3H), 1.81 (br s, 6H), 1.60 (br s, 6H) p.p.m.; ^13^C‐NMR (75 MHz, CDCl_3_) δ 167.3, 165.0, 141.9, 140.9, 131.7, 130.6, 130.1, 129.8, 128.4, 128.0, 127.5, 127.3, 63.6, 51.6, 41.0, 36.1, 29.2 p.p.m.; HRMS (ESI^+^): *m/z* = 390.20607 [M + H]^+^; calcd. for C_25_H_27_O_3_N + H^+^: 390.20637.

#### 2‐((3*S*,5*S*,7*S*)‐adamantan‐1‐ylamino)‐2‐oxoethyl butyrate (24)

The title compound was prepared from 1‐isocyanoadamantane (**3**), 37% aqueous formaldehyde solution and butanoic acid (**8**) according to general procedure A. After extraction, the product is obtained as a colorless oil; yield 95%; IR (KBr) $\tilde{\nu }$ 3297, 3073, 2908, 2849, 1749, 1664, 1551, 1421, 1170, 1109 cm^−1^; ^1^H‐NMR (300 MHz, CDCl_3_) δ 5.78 (br s, 1H), 4.32 (s, 2H), 2.27 (t, *J* = 7.3 Hz, 2H), 1.96 (br s, 3H), 1.90 (br s, 6H), 1.57 (br s, 8H), 0.86 (t, *J* = 7.5 Hz, 3H) p.p.m.; ^13^C‐NMR (75 MHz, CDCl_3_) δ 171.9, 165.8, 62.8, 51.8, 41.3, 36.1, 35.7, 29.2, 18.2, 13.5 p.p.m.; HRMS (ESI^+^): *m/z* = 280.19042 [M + H]^+^; calcd. for C_16_H_25_O_3_N + H^+^: 280.19072.

#### 2‐((3*S*,5*S*,7*S*)‐adamantan‐1‐ylamino)‐2‐oxoethyl cyclopentane carboxylate (25)

The title compound was prepared from 1‐isocyanoadamantane (**3**), 37% aqueous formaldehyde solution and cyclopentanecarboxylic acid (**9**) according to general procedure A. After extraction, the crude was purified by flash column chromatography using PE/EtOAc 9 : 1 as eluent to give a white solid; yield 83%; mp 104–106 °C; IR (KBr) $\tilde{\nu }$ 3280, 2910, 2851, 1743, 1673, 1558, 1153, 1096, 997, 814 cm^−1^; ^1^H‐NMR (300 MHz, CDCl_3_) δ 5.76 (br s, 1H), 4.34 (s, 2H), 2.73 (quint, *J* = 7.6 Hz, 1H), 1.98 (br s, 3H), 1.91 (br s, 6H), 1.88–1.49 (m, 14H) p.p.m.; ^13^C‐NMR (75 MHz, CDCl_3_) δ 175.0, 165.9, 62.8, 51.8, 43.4, 41.4, 36.1, 29.9, 29.3, 25.7 p.p.m.; HRMS (ESI^+^): *m/z* = 306.20594 [M + H]^+^; calcd. for C_18_H_27_O_3_N + H^+^: 306.20637.

#### 2‐((3*S*,5*S*,7*S*)‐adamantan‐1‐ylamino)‐2‐oxoethyl nicotinate (26)

The title compound was prepared from 1‐isocyanoadamantane (**3**), 37% aqueous formaldehyde solution and nicotinic acid (**10**) according to general procedure A. After extraction, the crude was purified by flash column chromatography, using PE/EtOAc 5 : 5 as eluent to give a white solid; yield 82%; mp 155–157 °C; IR (KBr) $\tilde{\nu }$ 3273, 3094, 2913, 2852, 1732, 1658, 1568, 1271, 1134, 741 cm^−1^; ^1^H‐NMR (300 MHz, CDCl_3_) δ 9.13 (s, 1H), 8.70 (d, *J* = 3.4 Hz, 1H), 8.22 (d, *J* = 8.0 Hz, 1H), 7.33 (dd, *J* = 8.0/4.9 Hz, 1H), 5.95 (br s, 1H), 4.63 (s, 2H) 1.97 (br s, 3H), 1.92 (d, *J* = 2.1 Hz, 6H), 1.56 (br s, 6H) p.p.m.; ^13^C‐NMR (75 MHz, CDCl_3_) δ 165.2, 164.0, 153.8, 150.7, 137.1, 125.2, 123.4, 63.6, 52.1, 41.4, 36.1, 29.2 p.p.m.; HRMS (ESI^+^): *m/z* = 315.16979 [M + H]^+^; calcd. for C_18_H_22_O_3_N_2_ + H^+^: 315.17032.

#### 2‐((3*S*,5*S*,7*S*)‐adamantan‐1‐ylamino)‐2‐oxoethyl 4‐acetamidobutanoate (27)

The title compound was prepared from 1‐isocyanoadamantane (**3**), 37% aqueous formaldehyde solution and 4‐acetamidobutanoic acid (**11**) according to general procedure A. After extraction, the product is obtained as a white solid; yield 94%; mp 102–104 °C; IR (KBr) $\tilde{\nu }$ 3560, 3415, 3074, 2908, 2850, 1742, 1697, 1547, 1361, 1175 cm^−1^; ^1^H‐NMR (300 MHz, CDCl_3_) δ 7.03 (br s, 1H), 6.25 (br s, 1H), 4.32 (s, 2H), 3.14 (q, *J* = 6.1 Hz, 2H), 2.31 (t, *J* = 6.9 Hz, 2H), 1.93 (br s, 3H), 1.88 (br s, 6H), 1.83 (s, 3H), 1.73 (quint, *J* = 6.7 Hz, 2H), 1.53 (br s, 6H) p.p.m.; ^13^C‐NMR (75 MHz, CDCl_3_) δ 171.9, 170.7, 166.0, 62.7, 52.0, 41.2, 38.4, 36.0, 31.3, 29.2, 24.6, 22.7 p.p.m.; HRMS (ESI^+^): *m/z* = 337.21169 [M + H]^+^; calcd. for C_18_H_28_O_4_N_2_ + H^+^: 337.21218.

#### 2‐((3*S*,5*S*,7*S*)‐adamantan‐1‐ylamino)‐2‐oxoethyl 4‐chlorobenzoate (28)

The title compound was prepared from 1‐isocyanoadamantane (**3**), 37% aqueous formaldehyde solution and 4‐chlorobenzoic acid (**12**) according to general procedure A. After extraction, the crude was purified by flash column chromatography, using PE/EtOAc 7 : 3 as eluent to give a white solid; yield 91%; mp 165–167 °C; IR (KBr) $\tilde{\nu }$ 3307, 3074, 2906, 2849, 1720, 1658, 1552, 1117, 855, 764, 686 cm^−1^; ^1^H‐NMR (300 MHz, CDCl_3_) δ 7.94 (d, *J* = 8.3 Hz, 2H), 7.40 (d, *J* = 8.6 Hz, 2H), 5.81 (br s, 1H), 4.64 (s, 2H), 2.03 (br s, 3H), 1.98 (br s, 6H), 1.63 (br s, 6H) p.p.m.; ^13^C‐NMR (75 MHz, CDCl_3_) δ 165.6, 164.4, 140.1, 131.1, 129.0, 127.6, 63.7, 52.2, 41.6, 36.2, 29.4 p.p.m.; HRMS (ESI^+^): *m/z* = 348.13573 [M + H]^+^; calcd. for C_19_H_22_O_3_ClN + H^+^: 348.13610.

#### 2‐((3*S*,5*S*,7*S*)‐adamantan‐1‐ylamino)‐2‐oxoethyl 3‐nitrobenzoate (29)

The title compound was prepared from 1‐isocyanoadamantane (**3**), 37% aqueous formaldehyde solution and 3‐nitrobenzoic acid (**13**) according to general procedure A. After extraction, the crude was purified by flash column chromatography, using PE/EtOAc 9 : 1 and PE/EtOAc 7 : 3 as eluents to give a white solid; yield 76%; mp 122–124 °C; IR (KBr) $\tilde{\nu }$ 3282, 3083, 2905, 2848, 1729, 1533, 1352, 1263, 1143, 715 cm^−1^; ^1^H‐NMR (300 MHz, CDCl_3_) δ 8.78 (s, 1H), 8.39–8.32 (m, 2H), 7.64 (t, *J* = 8.0 Hz, 1H), 5.89 (br s, 1H), 4.70 (s, 2H), 2.01 (br s, 3H), 1.98 (br s, 6H), 1.61 (br s, 6H) p.p.m.; ^13^C‐NMR (75 MHz, CDCl_3_) δ 165.1, 163.4, 148.2, 135.4, 131.0, 129.9, 127.8, 124.6, 64.0, 52.3, 41.5, 36.2, 29.3 p.p.m.; HRMS (ESI^+^): *m/z* = 359.15987 [M + H]^+^; calcd. for C_19_H_22_O_5_N_2_ + H^+^: 359.16015.

#### General procedure B for the synthesis of α‐aminoacyl amides 30–41

To a solution of 1‐isocyanoadamantane (**3**) (0.070 g, 0.40 mmol, 1 equiv) in methanol (3 mL), paraformaldehyde (0.026 g, 0.60 mmol, 1.5 equiv), the corresponding amine (0.40 mmol, 1 equiv) and the corresponding carboxylic acid (0.40 mmol, 1 equiv) were added. The reaction mixture was stirred at 60 °C for 4 h. After completion of the reaction, water was added and the product was extracted with dichloromethane (x2). The combined organic layers were washed with water (x1), NaOH 2 m (x1), brine (x1) and dried over sodium sulfate. After evaporation of the solvent under reduced pressure, the crude material was purified by flash column chromatography.

#### *N*‐(2‐(((3*S*,5*S*,7*S*)‐adamantan‐1‐yl)amino)‐2‐oxoethyl)‐*N*‐cyclopropylbenzamide (30)

The title compound was prepared from 1‐isocyanoadamantane (**3**), paraformaldehyde; cyclopropylamine (**15**) and benzoic acid (**4**) according to general procedure B. After extraction, the crude was purified by flash column chromatography, using PE/EtOAc 8 : 2 as eluent to give a white solid; yield 73%; mp 71–72 °C; IR (KBr) $\tilde{\nu }$ 3445, 3316, 3066, 2906, 2849, 1645, 1626, 1548, 1426, 728, 702 cm^−1^; ^1^H‐NMR (300 MHz, CDCl_3_) δ 7.55–7.34 (m, 5H), 6.05 (br s, 1H), 4.06 (s, 2H), 2.96–2.88 (m, 1H), 2.05 (br s, 3H), 1.98 (br s, 6H), 1.65 (br s, 6H), 0.60–0.50 (m, 4H) p.p.m.; ^13^C‐NMR (75 MHz, CDCl_3_) δ 173.4, 168.5, 136.7, 130.0, 128.2, 127.4, 53.5, 52.0, 41.6, 36.4, 33.6, 29.5, 9.8 p.p.m.; HRMS (ESI^+^): *m/z* = 353.22214 [M + H]^+^; calcd. for C_22_H_28_O_2_N_2_ + H^+^: 353.22235.

#### *N*‐(2‐(((3*S*,5*S*,7*S*)‐adamantan‐1‐yl)amino)‐2‐oxoethyl)‐*N*‐cyclopropylbutyramide (31)

The title compound was prepared from 1‐isocyanoadamantane (**3**), paraformaldehyde; cyclopropylamine (**15**) and butanoic acid (**8**) according to general procedure B. After extraction, the crude was purified by flash column chromatography, using PE/EtOAc 7 : 3 and PE/EtOAc 5 : 5 as eluents to give an off‐white solid; yield 72%; mp 126–127 °C; IR (KBr) $\tilde{\nu }$ 3312, 3073, 2906, 2849, 1679, 16 345, 1554, 1428, 1362, 1290, 1275, 744 cm^−1^; ^1^H‐NMR (300 MHz, CDCl_3_) δ 5.87 (br s, 1H), 3.85 (s, 2H), 2.81–2.73 (m, 1H), 2.52 (t, *J* = 7.3 Hz, 2H), 2.01 (br s, 3H), 1.91 (br s, 6H), 1.73–1.61 (m, 8H), 0.94 (t, *J* = 7.3 Hz, 3H), 0.91–0.81 (m, 4H) p.p.m.; ^13^C‐NMR (75 MHz, CDCl_3_) δ 176.8, 169.0, 53.2, 51.8, 41.6, 36.4, 35.9, 31.7, 29.5, 18.5, 14.1, 9.3 p.p.m.; HRMS (ESI^+^): *m/z* = 319.23778 [M + H]^+^; calcd. for C_19_H_30_O_2_N_2_ + H^+^: 319.23800.

#### *N*‐(2‐(((3*S*,5*S*,7*S*)‐adamantan‐1‐yl)amino)‐2‐oxoethyl)‐*N*‐cyclopropylcyclopentanecarboxamide (32)

The title compound was prepared from 1‐isocyanoadamantane (**3**), paraformaldehyde; cyclopropylamine (**15**) and cyclopentanecarboxylic acid (**9**) according to general procedure B. After extraction, the crude was purified by flash column chromatography, using PE/EtOAc 9 : 1 and PE/EtOAc 8 : 2 as eluents to give a white solid; yield 52%; mp 152–153 °C; IR (KBr) $\tilde{\nu }$ 3407, 3321, 2906, 2849, 1681, 1630, 1546, 1429, 1361, 1273 cm^−1^; ^1^H‐NMR (300 MHz, CDCl_3_) δ 5.91 (br s, 1H), 3.85 (s, 2H), 3.42–3.32 (m, 1H), 2.83–2.76 (m, 1H), 1.99–1.54 (m, 23H), 0.86–0.80 (m, 4H) p.p.m.; ^13^C‐NMR (75 MHz, CDCl_3_) δ 180.5, 169.0, 53.3, 51.6, 41.5, 41.4, 36.3, 31.5, 30.6, 29.4, 26.3, 9.1 p.p.m. HRMS (ESI^+^): *m/z* = 345.25329 [M + H]^+^; calcd. for C_21_H_32_O_2_N_2_ + H^+^: 345.25365.

#### *N*‐(2‐(((3*S*,5*S*,7*S*)‐adamantan‐1‐yl)amino)‐2‐oxoethyl)‐*N*‐cyclopropyl‐[1,1′‐biphenyl]‐2‐carboxamide (33)

The title compound was prepared from 1‐isocyanoadamantane (**3**), paraformaldehyde; cyclopropylamine (**15**) and [1,1′‐biphenyl]‐2‐carboxylic acid (**7**) according to general procedure B. After extraction, the crude was purified by flash column chromatography, using PE/EtOAc 8 : 2 and PE/EtOAc 7 : 3 as eluents to give a white solid; yield 49%; mp 83–84 °C; IR (KBr) $\tilde{\nu }$ 3313, 3060, 2906, 2850, 1683, 1629, 1542, 1453, 745, 701 cm^−1^; ^1^H‐NMR (300 MHz, DMSO‐*d*_6_ 75 °C) δ 7.48–7.35 (m, 9H), 6.94 (br s, 1H), 3.76 (br s, 2H), 3.30 (br s, 1H), 2.01–1.84 (m, 9H), 1.64 (br s, 6H), 0.56–0.24 (m, 4H) p.p.m.; ^13^C‐NMR (75 MHz, DMSO‐*d*_6_ 75 °C) δ 172.0, 167.0, 139.7, 137.6, 136.5, 129.0, 128.6, 128.1, 128.0, 127.2, 127.1, 126.7, 51.9, 50.3, 40.9, 35.8, 31.4, 28.6, 7.5 p.p.m.; HRMS (ESI^+^): *m/z* = 429.25359 [M + H]^+^; calcd. for C_28_H_32_O_2_N_2_ + H^+^: 429.25365.

#### *N*‐((3*S*,5*S*,7*S*)‐adamantan‐1‐yl)‐2‐(*N*‐cyclopropylacetamido)acetamide (34)

The title compound was prepared from 1‐isocyanoadamantane (**3**), paraformaldehyde; cyclopropylamine (**15**) and acetic acid (**6**) according to general procedure B. After extraction, the crude was purified by flash column chromatography, using PE/EtOAc 7 : 3 and PE/EtOAc 5 : 5 as eluents to give a white solid; yield 70%; mp 73–74 °C; IR (KBr) $\tilde{\nu }$ 3330, 3053, 2906, 2849, 1660, 1544, 1422, 1360, 1291, 1030 cm^−1^; ^1^H‐NMR (300 MHz, CDCl_3_) δ 5.86 (br s, 1H), 3.82 (s, 2H), 2.81–2.74 (m, 1H), 2.19 (s, 3H), 1.97 (br s, 3H), 1.90 (br s, 6H), 1.59 (br s, 6H), 0.83–0.76 (m, 4H) p.p.m.; ^13^C‐NMR (75 MHz, CDCl_3_) δ 174.3, 168.6, 52.7, 51.8, 41.4, 36.3, 32.3, 29.4, 22.4, 9.1 p.p.m.; HRMS (ESI^+^): *m/z* = 291.20628 [M + H]^+^; calcd. for C_17_H_26_O_2_N_2_ + H^+^: 291.20670.

#### *N*‐(2‐(((3*S*,5*S*,7*S*)‐adamantan‐1‐yl)amino)‐2‐oxoethyl)‐*N*‐cyclopropylbenzo[b]thiophene‐2‐carboxamide (35)

The title compound was prepared from 1‐isocyanoadamantane (**3**), paraformaldehyde; cyclopropylamine (**15**) and benzo[b]thiophene‐2‐carboxylic acid (**14**) according to general procedure B. After extraction, the crude was purified by flash column chromatography, using PE/EtOAc 7 : 3 and PE/EtOAc 5 : 5 as eluents to give a white solid; yield 23%; mp 193–194 °C; IR (KBr) $\tilde{\nu }$ 3411, 3309, 3142, 2907, 2851, 1666, 1600, 1515, 1390, 1288, 961, 760 cm^−1^; ^1^H‐NMR (300 MHz, CDCl_3_) signals are referred to the main rotamer, δ 7.89–7.79 (m, 3H), 7.44–7.33 (m, 2H), 6.04 (br s, 1H), 4.10 (s, 2H), 3.22–3.14 (m, 1H), 2.14–1.97 (m, 9H), 1,71–1.63 (m, 6H), 0.89–0.82 (m, 4H) p.p.m.; ^13^C‐NMR (75 MHz, CDCl_3_) signals are referred to the main rotamer, δ 168.3, 166.5, 141.0, 138.8, 137.7, 127.9, 126.3, 125.2, 124.8, 122.4, 54.6, 52.1, 41.6, 36.4, 33.4, 29.5, 10.7 p.p.m.; HRMS (ESI^+^): *m/z* = 409.19433 [M + H]^+^; calcd. for C_24_H_28_O_2_N_2_ + H^+^: 409.19443.

#### *N*‐(2‐(((3*S*,5*S*,7*S*)‐adamantan‐1‐yl)amino)‐2‐oxoethyl)‐*N*‐cyclopropyl‐2‐phenylacetamide (36)

The title compound was prepared from 1‐isocyanoadamantane (**3**), paraformaldehyde; cyclopropylamine (**15**) and phenylacetic acid (**5**) according to general procedure B. After extraction, the crude was purified by flash column chromatography, using PE/EtOAc 7 : 3 as eluent to give an off‐white solid; yield 53%; mp 119–120 °C; IR (KBr) $\tilde{\nu }$ 3329, 3025, 2905, 2851, 1723, 1689, 1639, 1454, 1300, 1042, 742, 696 cm^−1^; ^1^H‐NMR (300 MHz, CDCl_3_) δ 7.29–7.20 (m, 5H), 5.84 (br s, 1H), 3.92 (s, 2H), 3.88 (s, 2H), 2.77–2.69 (m, 1H), 2.00 (br s, 3H), 1.85 (br s, 6H), 1.61 (br s, 6H), 0.89–0.81 (m, 4H) p.p.m.; ^13^C‐NMR (75 MHz, CDCl_3_) δ 174.6, 168.5, 135.0, 129.1, 128.7, 126.9, 53.1, 51.7, 41.4, 41.1, 36.3, 32.0, 29.4, 9.5 p.p.m.; HRMS (ESI^+^): *m/z* = 367.23767 [M + H]^+^; calcd. for C_23_H_30_O_2_N_2_ + H^+^: 367.23800.

#### *N*‐((3*S*,5*S*,7*S*)‐adamantan‐1‐yl)‐2‐(*N*‐cyclohexylacetamido)acetamide (37)

The title compound was prepared from 1‐isocyanoadamantane (**3**), paraformaldehyde; cyclohexylamine (**16**) and acetic acid (**6**) according to general procedure B. After extraction, the crude was purified by flash column chromatography, using PE/EtOAc 7 : 3 and PE/EtOAc 5 : 5 as eluents to give a white solid; yield 78%; mp 77–78 °C; IR (KBr) $\tilde{\nu }$ 3309, 2907, 2851, 1669, 1627, 1538, 1453, 1416, 1359, 1241, 976 cm^−1^; ^1^H‐NMR (300 MHz, CDCl_3_) signals are referred to the main rotamer, δ 6.36 (br s, 1H), 3.74 (s, 2H), 3.50–3.41 (m, 1H), 2.10 (s, 3H), 1.99–1.87 (m, 9H), 1.80–1.46 (m, 12H), 1.33–1.16 (m, 4H) p.p.m.; ^13^C‐NMR (75 MHz, CDCl_3_) signals are referred to the main rotamer, δ 171.3, 169.6, 58.7, 51.4, 48.4, 47.4, 41.4, 36.3, 31.1, 30.4, 29.3, 25.8, 25.0, 21.7 p.p.m.; HRMS (ESI^+^): *m/z* = 333.25320 [M + H]^+^; calcd. for C_20_H_32_O_2_N_2_ + H^+^: 333.25365.

#### *N*‐((3*S*,5*S*,7*S*)‐adamantan‐1‐yl)‐2‐(*N*‐((3*S*,5*S*,7*S*)‐adamantan‐1‐yl)acetamido)acetamide (38)

The title compound was prepared from 1‐isocyanoadamantane (**3**), paraformaldehyde, (3S,5S,7S)‐adamantan‐1‐amine (**1**), and acetic acid (**6**) according to general procedure B. After extraction, the crude was purified by flash column chromatography, using PE/EtOAc 7 : 3 and PE/EtOAc 5 : 5 as eluents to give a white solid; yield 87%; mp 239–240 °C; IR (KBr) $\tilde{\nu }$ 3302, 3064, 2908, 2849, 1691, 1623, 1549, 1414, 1359, 988, 902 cm^−1^; ^1^H‐NMR (300 MHz, CDCl_3_) δ 5.80 (br s, 1H), 3.78 (s, 2H), 2.11 (br s, 6H), 2.02–1.92 (m, 15H), 1.61 (br s, 12H) p.p.m.; ^13^C‐NMR (75 MHz, CDCl_3_) δ 172.1, 168.8, 59.2, 52.1, 49.6, 41.6, 40.0, 36.2, 36.1, 30.1, 29.3, 25.8 p.p.m.; HRMS (ESI^+^): *m/z* = 385.28461 [M + H]^+^; calcd. for C_24_H_36_O_2_N_2_ + H^+^: 385.28495.

#### *N*‐((3*S*,5*S*,7*S*)‐adamantan‐1‐yl)‐2‐(*N*‐butylacetamido)acetamide (39)

The title compound was prepared from 1‐isocyanoadamantane (**3**), paraformaldehyde, butan‐1‐amine (**17**), and acetic acid (**6**) according to general procedure B. After extraction, the crude was purified by flash column chromatography, using PE/EtOAc 7 : 3 and PE/EtOAc 5 : 5 as eluents to give a white solid; yield 70%; mp 124–125 °C; IR (KBr) $\tilde{\nu }$ 3296, 2907, 2853, 1644, 1537, 1414, 1305, 1245, 985, 708 cm^−1^; ^1^H‐NMR (300 MHz, CDCl_3_) signals are referred to the main rotamer, δ 6.20 (br s, 1H), 3.75 (s, 2H), 3.26 (t, *J* = 7.4 Hz, 2H), 2.05–1.87 (m, 12H), 1.60–1.46 (m, 8H), 1.24–1.20 (m, 2H), 0.85 (t, *J* = 7.0 Hz, 3H) p.p.m.; ^13^C‐NMR (75 MHz, CDCl_3_) signals are referred to the main rotamer, δ 171.2, 168.5, 52.0, 50.4, 47.1, 41.4, 36.2, 30.6, 29.3, 21.1, 19.9, 13.7 p.p.m.; HRMS (ESI^+^): *m/z* = 307.23759 [M + H]^+^; calcd. for C_18_H_30_O_2_N_2_ + H^+^: 307.23800.

#### *N*‐((3*S*,5*S*,7*S*)‐adamantan‐1‐yl)‐2‐(*N*‐benzylacetamido)acetamide (40)

The title compound was prepared from 1‐isocyanoadamantane (**3**), paraformaldehyde, benzylamine (**18**), and acetic acid (**6**) according to general procedure B. After extraction, the product was obtained as a white solid; yield 78%; mp 195–196 °C; IR (KBr) $\tilde{\nu }$ 3312, 3030, 2920, 2854, 1642, 1533, 1409, 1344, 1004, 722, 693 cm^−1^; ^1^H‐NMR (300 MHz, CDCl_3_) signals are referred to the main rotamer, δ 7.35–7.26 (m, 5H), 5.90 (br s, 1H), 4.62 (s, 2H), 3.83 (s, 2H), 2.16–1.61 (m, 18H) p.p.m.; ^13^C‐NMR (75 MHz, CDCl_3_) signals are referred to the main rotamer, δ 171.7, 167.9, 136.2, 129.1, 127.9, 126.8, 53.5, 52.0, 51.3, 41.7, 36.5, 29.6, 21.5 p.p.m.; HRMS (ESI^+^): *m/z* = 341.22196 [M + H]^+^; calcd. for C_21_H_28_O_2_N_2_ + H^+^: 341.22235.

#### *N*‐(2‐(((3*S*,5*S*,7*S*)‐adamantan‐1‐yl)amino)‐2‐oxoethyl)‐*N*‐benzyl‐2‐phenylacetamide (41)

The title compound was prepared from 1‐isocyanoadamantane (**3**), paraformaldehyde, benzylamine (**18**), and phenylacetic acid (**5**) according to general procedure B. After extraction, the crude was purified by flash column chromatography, using PE/EtOAc 7 : 3 as eluent to give a white solid; yield 63%; mp 60–61 °C; IR (KBr) $\tilde{\nu }$ 3300, 3063, 3029, 2906, 2849, 1681, 1632, 1545, 1453, 1359, 724, 697 cm^−1^; ^1^H‐NMR (300 MHz, CDCl_3_) signals are referred to the main rotamer, δ 7.31–7.26 (m, 10H), 5.98 (br s, 1H), 4.60 (s, 2H), 3.84 (s, 2H), 3.77 (s, 2H), 2.02–1.60 (m, 15H) p.p.m.; ^13^C‐NMR (75 MHz, CDCl_3_) signals are referred to the main rotamer, δ 172.2, 167.6, 135.8, 134.6, 129.0, 128.9, 128.6, 127.9, 127.1, 126.7, 52.5, 51.8, 51.2, 41.4, 40.7, 36.3, 29.4 p.p.m.; HRMS (ESI^+^): *m/z* = 417.25337 [M + H]^+^; calcd. for C_27_H_32_O_2_N_2_ + H^+^: 417.25365.

#### General procedure C for the synthesis of α‐acylpiperazino amides 42–45

To a solution of piperazine (**19**) (0.037 g, 0.40 mmol, 1 equiv) in methanol (3 mL) were added paraformaldehyde (0.031 g, 0.60 mmol, 1.5 equiv), the corresponding carboxylic acid (0.40 mmol, 1 equiv) and 1‐isocyanoadamantane (**3**) (0.070 g, 0.40 mmol, 1 equiv) sequentially at room temperature. The reaction mixture was heated at 60 °C for 3 h, and the solvent was evaporated. The crude material was purified by flash column chromatography.

#### *N*‐((3*S*,5*S*,7*S*)‐adamantan‐1‐yl)‐2‐(4‐benzoylpiperazin‐1‐yl)acetamide (42)

The title compound was prepared from 1‐isocyanoadamantane (**3**), paraformaldehyde, piperazine (**19**), and benzoic acid (**4**) according to general procedure C. The crude was purified by flash column chromatography using EtOAc as eluent to give a white solid; yield 62%; mp 141–142 °C; IR (KBr) $\tilde{\nu }$ 3346, 3019, 2909, 2848, 1668, 1638, 1530, 1458, 1428, 1138, 1017, 715 cm^−1^; ^1^H‐NMR (300 MHz, CDCl_3_) δ 7.35 (br s, 5H), 6.75 (br s, 1H), 3.72–3.44 (m, 4H), 2.88 (s, 2H), 2.50 (br s, 4H), 2.03–1.94 (m, 9H), 1.64 (br s, 6H) p.p.m.; ^13^C‐NMR (75 MHz, CDCl_3_) δ 170.4, 168.3, 135.6, 129.8, 128.6, 127.0, 62.2, 53.3 (4C), 51.2, 41.7, 36.3, 29.4 p.p.m.; HRMS (ESI^+^): *m/z* = 382.24859 [M + H]^+^; calcd. for C_23_H_31_O_2_N_3_ + H^+^: 382.24890.

#### *N*‐((3*S*,5*S*,7*S*)‐adamantan‐1‐yl)‐2‐(4‐(cyclopentanecarbonyl)piperazin‐1‐yl)acetamide (43)

The title compound was prepared from 1‐isocyanoadamantane (**3**), paraformaldehyde, piperazine (**19**), and cyclopentanecarboxylic acid (**9**) according to general procedure C. The crude was purified by flash column chromatography using EtOAc as eluent to give a white solid; yield 75%; mp 118–119 °C; IR (KBr) $\tilde{\nu }$ 3270, 3062, 2907, 2852, 2811, 1672, 1644, 1552, 1514, 1452, 1239, 814, 715 cm^−1^; ^1^H‐NMR (300 MHz, CDCl_3_) δ 6.80 (br s, 1H), 3.56 (br s, 2H), 3.47 (br s, 2H), 2.90–2.74 (m, 3H), 2.46–2.40 (m, 4H), 2.01–1.91 (m, 9H), 1.76–1.48 (m, 14H) p.p.m.; ^13^C‐NMR (75 MHz, CDCl_3_) δ 174.6, 168.5, 62.0, 53.4, 53.0, 51.2, 45.4, 41.8, 41.6, 40.9, 36.2, 30.0, 29.3, 25.9 p.p.m.; HRMS (ESI^+^): *m/z* = 374.27984 [M + H]^+^; calcd. for C_22_H_35_O_2_N_3_ + H^+^: 374.28020.

#### *N*‐((3*S*,5*S*,7*S*)‐adamantan‐1‐yl)‐2‐(4‐(2‐phenylacetyl)piperazin‐1‐yl)acetamide (**44**)

The title compound was prepared from 1‐isocyanoadamantane (**3**), paraformaldehyde, piperazine (**19**), and phenylacetic acid (**5**) according to general procedure C. The crude was purified by flash column chromatography, using EtOAc as eluent to give a white solid; yield 83%; mp 145–146 °C; IR (KBr) $\tilde{\nu }$ 3321, 3019, 2908, 2848, 1664, 1630, 1511, 1456, 1241, 1143, 736, 723 cm^−1^; ^1^H‐NMR (300 MHz, CDCl_3_) δ 7.28–7.15 (m, 5H), 6.70 (br s, 1H), 3.67 (s, 2H), 3.59 (br s, 2H), 3.40–3.37 (m, 2H), 2.78 (s, 2H), 2.41–2.38 (m, 2H), 2.26–2.23 (m, 2H), 2.02–1.91 (m, 9H), 1.61 (br s, 6H) p.p.m.; ^13^C‐NMR (75 MHz, CDCl_3_) δ 169.4, 168.2, 134.8, 128.7, 128.4, 126.8, 62.0, 53.0, 52.8, 51.1, 46.0, 41.7, 41.6, 40.9, 36.2, 29.3 p.p.m.; HRMS (ESI^+^): *m/z* = 396.26419 [M + H]^+^; calcd. for C_24_H_33_O_2_N_3_ + H^+^: 396.26455.

#### 2‐(4‐acetylpiperazin‐1‐yl)‐*N*‐((3*S*,5*S*,7*S*)‐adamantan‐1‐yl)acetamide (**45**)

The title compound was prepared from 1‐isocyanoadamantane (**3**), paraformaldehyde, piperazine (**19**), and acetic acid (**6**) according to general procedure C. The crude was purified by flash column chromatography, using EtOAc/MeOH 95 : 5 as eluent to give a white solid; yield 76%; mp 135–136 °C; IR (KBr) $\tilde{\nu }$ 3322, 3008, 2915, 2848, 2901, 1667, 1654, 1512, 1427, 1273, 1245, 1006, 990 cm^−1^; ^1^H‐NMR (300 MHz, CDCl_3_) δ 6.73 (br s, 1H), 3.55 (br s, 2H), 3.41 (br s, 2H), 2.84 (s, 2H), 2.47–2.39 (m, 4H), 2.02–1.92 (m, 12H), 1.61 (br s, 6H) p.p.m.; ^13^C‐NMR (75 MHz, CDCl_3_) δ 168.9, 168.2, 62.1, 53.2, 52.9, 51.2, 46.2, 41.6, 41.4, 36.2, 29.3, 21.3 p.p.m.; HRMS (ESI^+^): *m/z* = 320.23295 [M + H]^+^; calcd. for C_18_H_29_O_2_N_3_ + H^+^: 320.23325.

### Biology

#### Animals

C57BL/6JRccHSd female mice (15‐23‐week‐old, 24–35 g; Harlan, the Netherlands) bred at the animal facility at Universidad Miguel Hernández de Elche (UMH, Elche, Spain) were used to assess the antinociceptive effects of compound **23** on the model of chemotherapy‐induced neuropathic pain. Housing conditions were maintained at 21 ± 1 °C and 55 ± 20% relative humidity in a controlled light/dark cycle (light on between 8:00 a.m. and 8:00 p.m). Care was taken to minimize the number of animals used and the pain and stress they experienced. Animal experimentation procedures were conducted under the approval of the Institutional Animal and Ethical Committee at UMH, following the guidelines of the European Community (2010/63/EU), and the Committee for Research and Ethical Issues of the International Association for the Study of Pain [64]. The study protocol received approval from the Ethical Committee of Universidad Miguel Hernández de Elche (UMH, Elche, Spain) and the regional government (approval code: 2022 VSC PEA 0078‐2). For the *in vivo* behavioral experiments evaluating the effects of the TRPM8 antagonist on mice with chemotherapy‐induced neuropathy, male C57BL/6JRccHSd mice (15–23 weeks old; Harlan, the Netherlands) were utilized. The animals were bred and housed at the UMH Animal Facility (Servicio de Experimentación Animal, UMH, Elche, Spain). The experimental protocol was reviewed and approved by the Institutional Animal and Ethical Committee of UMH.

#### Primary cultures of dorsal root ganglia neurons

Primary cultures of neonatal dorsal root ganglia were used for multielectrode array (MEA) and patch‐clamp experiments following established methodologies previously described [65]. Neonatal dorsal root ganglia (DRGs) were obtained from Wistar rats (3–5 days old) and were isolated and digested with 0.25% (w/v) collagenase (type IA) in DMEM GlutaMax with 1% (v/v) penicillin/streptomycin (P/S) solution for 1 h at 37 °C in a 5% CO_2_ Thermo Scientific incubator. Following digestion, DRGs were mechanically dissociated and passed through a 100 μm cell strainer to obtain single‐cell suspensions. Suspensions were washed with DMEM GlutaMax with 10% (v/v) fetal bovine serum (FBS) and 1% (v/v) penicillin/streptomycin (P/S). Cells were then seeded in microelectrode array chambers coated with poly‐l‐lysine (8.3 μg·mL^−1^) and laminin (5 μg·mL^−1^). After 1 h, the medium was replaced with DMEM GlutaMax, 10% (v/v) FBS, and 1% (v/v) P/S, supplemented with mouse 2.5S NGF 50 ng·mL^−1^ and 1.25 μg·mL^−1^ cytosine arabinoside. Isolated mouse cells were incubated with 0.67% (w/v) collagenase type XI and 3% (w/v) dispase (Gibco) in INC mix medium (in mm): 155 NaCl, 1.5 K_2_HPO_4_, 5.6 HEPES, 4.8 NaHEPES, and 5 glucose for 1 h (37 °C, 5% 3 CO_2_). Primary cultures of mouse DRGs were used for patch‐clamp experiments. Mouse DRG were mechanically dissociated using a glass Pasteur pipette. Single‐cell suspensions were passed through a 100 μm cell strainer and washed with DMEM GlutaMax plus 10% FBS (Invitrogen (Thermo Fisher Scientific), Barcelona, Spain) and 1% P/S. For each experiment, cells were seeded at the required density on 12 mm coverglass slides in a 24‐well plate or microelectrode array chambers previously coated with poly‐l‐lysine (8.33 μg·mL^−1^) and laminin (5 μg·mL^−1^). After 2 h, the medium was replaced with DMEM GlutaMax, 10% FBS, and 1% P/S, supplemented with mouse 2.5 s NGF 50 ng·mL^−1^ (Promega). All experiments in patch‐clamp were conducted 48 h after cell seeding.

#### Calcium microfluorimetric assay

Assays were conducted to assess the channel activity of the recombinant hTRPM8 stably expressed in HEK‐293 cells. HEK‐293‐hTRPM8 cells were cultured in a monolayer using a DMEM GlutaMax with 10% FBS and 1% P/S and maintained at 37 °C in 5% CO_2_. For experiments, cells were prepared and seeded at the indicated densities. To evaluate compound effectiveness against hTRPM8 activity, microfluorometry‐based calcium flux assays were performed using Fluo‐4 NW Ca^2+^ dye and fluorescence detection. HEK cell lines expressing hTRPM8 were seeded in 96‐well plates and incubated with the dye‐loading solution. Ion channel activity was measured using a plate reader, and fluorescence intensity changes were recorded following the addition of vehicle, compound at varying concentrations, and antagonist (10 μm AMTB). Data analysis included calculating the Z‐factor for each assay and normalizing compound effects to capsaicin‐induced fluorescence. Data were analyzed to determine the concentration exerting half‐maximal inhibition (IC_50_) or activation (EC_50_) of agonist‐induced calcium elevation, using graphpad prism8® software (Boston, MA, USA). All experiments were conducted in triplicate, and results are presented as mean ± standard deviation.

#### Patch‐clamp recordings from recombinant cells

Recording through patch‐clamp techniques was performed on HEK293 cells cultured in DMEM GlutaMax with 10% FBS and 1% P/S. The cell lines were authenticated following the standardization of STR Profiling guidelines (ANSI/ATCC ASN‐0002‐2022) using the CLA Identifier Plus PCR Amplification Kit (Thermo Fisher‐A44660) to analyze 16 highly variant human STRs. The cells were confirmed to be mycoplasma‐free. These cells were transiently transfected with plasmids encoding hTRPV1, hTRPM8, or hTRPA1 using Lipofectamine 3000. Transfected cells were seeded on 12 mm Ø glass coverslips treated with poly‐l‐lysine solution and recorded 2 days post‐transfection. The intracellular pipette solution included (in mm) 150 NaCl, 3 MgCl_2_, 5 EGTA, and 10 HEPES, pH 7.2 with CsOH, while the extracellular solution contained (in mm) 150 NaCl, 6 CsCl, 1 MgCl_2_, 1.5 CaCl_2_, 10 glucose, and 10 HEPES, pH 7.4 with NaOH. An EPC‐10 amplifier with Patchmaster software was used in whole‐cell experiments. Patch pipettes, created from thin‐wall borosilicate capillary glass tubing, were pulled with a Micropipette puller to a final resistance of 2–8 MΩ when filled with the internal solution. Recordings were acquired at 10 kHz and low‐pass filtered at 3 kHz, discarding recordings with leak currents > 200pA or series resistance > 20 MΩ. In voltage‐clamp recordings, cells were held at a constant potential, and the application of modulators was carried out using a gravity‐driven perfusion system. Total currents were normalized to the first current peak evoked by the activating stimuli. To study the effect of compound **23** on TRPM8 voltage dependence, a voltage step protocol from −120 to 120 mV was used with 100 ms steps of 20 mV from a holding potential at 0 mV. Leak currents were not subtracted, and conductance was calculated using the equation: *G* = *I*/(*V* − *V*_r_), where *I* is the measured ionic current, *V* is the applied voltage, and *V*_r_ is the reversal potential that for the ionic conditions used was set to 0 mV.

Dose–response relationships for WS‐12 channel activation were normalized to the response of the channel to a saturating concentration of the agonist. Similarly, dose–response curves for blockade activity were normalized with respect to the response in the absence of the blocker. The experimental data were fitted to the Hill equation: $Y=\text{Bottom}\;+\left(\mathrm{Top}‐\text{Bottom}\right)/(1+10^{\left(\left[\text{ligand}\right]-{\text{LogIC}}_{50}\left(\text{or}\;\mathrm{Log}\left({\mathrm{EC}}_{50}\right)\right)\right)})$ using prism 9 software. Top was fixed to 100, as the unblocked response or the maximal response for WS‐12.

#### Patch‐clamp recordings from DRG nociceptors

Two days after being seeded on 12 mm Ø glass coverslips treated with poly‐l‐lysine solution and Laminin (Sigma‐Aldrich), whole‐cell patch‐clamp recordings were conducted on sensory DRG neurons from adult mice. The intracellular pipette solution consisted of (in mm): 4 NaCl, 110 K gluconate, 1 CaCl_2_, 30 KCl, 2 MgCl_2_, 10 HEPES, 4 ATP, 0.4 GTP, and 10 EGTA, with a pH of 7.2 adjusted with KOH. The extracellular solution comprised (in mm): 140 NaCl, 4 KCl, 2 CaCl_2_, 2 MgCl_2_, 10 HEPES, 5 glucose, and 20 mannitol, with a pH of 7.4 adjusted with NaOH.

#### Microelectrode array (MEA)

Microelectrode array measurements were conducted using 60‐electrode thin MEA chips, featuring 30 μm diameter electrodes and 200 μm inter‐electrode spacing, including an integrated reference electrode (Multi Channel Systems GmbH, Reutlingen, Germany). The MEA1060 System (Multi Channel Systems GmbH) and mc rack software version 4.3.0 were employed to record the electrical activity of primary sensory neurons. Short 30 s applications (termed P1 and P2) of WS‐12 were applied using a continuous perfusion system (2 mL·min^−1^). Between each stimulus, cells underwent 4 min and 30 s washes with external solution. Treated cells were perfused with compound **23**, 1 min before and together with P2. The protocol concluded with the application of 40 mm KCl to confirm neuronal excitability and viability. All measurements were conducted at approximately 34.5 °C using the Multichannel Systems Temperature Controller. For microelectrode array analysis, data were processed using MC_RACK spike sorter with a sample rate of 25 kHz, applying a Butterworth high‐pass 2nd order filter with a 200 Hz cutoff. For AITC analysis, a cutoff of 500 Hz was applied. An evoked spike was defined when the amplitude of neuronal electrical activity reached a threshold established by automatic estimation at −4.7 μV Std. For AITC analysis, an evoked spike was defined when the amplitude of neuronal electrical activity reached a threshold established by automatic estimation at −5 μV Std. Spiking activity was measured for 60 s immediately following the instillation of activating stimuli. Electrodes lacking electrical activity in the first agonist pulse were excluded. The recorded signals were then analyzed to extract the mean spike frequency for each pulse (P1–P2). The ratio P2/P1 of mean spike frequency was calculated and normalized to the vehicle for comparison across different conditions.

#### Model of chemotherapy‐induced neuropathic pain

Chemotherapy‐induced sensitization was induced by repeated administration of oxaliplatin (ref#2623; Tocris, Bristol, UK). Oxaliplatin was freshly prepared every day by dissolution in 5% dextrose in warmed distilled water at 37 °C. The chemotherapeutic was administered intraperitoneally every other day for 5 days at a 6 mg·kg^−1^ and in a volume of 10 mL·kg^−1^, reaching an accumulated dose of 18 mg·kg^−1^ after the three injections.

#### Compound 23 treatment

The systemic effect of compound **23** was assessed after intraperitoneal administration at 5 mg·kg^−1^, dissolved in a solution of 2% Cremophor and 5% DMSO in saline, with a volume of 10 mL·kg^−1^. This dose was chosen based on the relative potency of the compound in the *in vitro* studies when compared to the canonical antagonist AMTB. Nociceptive sensitivity was evaluated for 30‐ and 90‐min postinjection using the acetone drop test (7 days after the first oxaliplatin dose) and the dry ice test (9 days after the last oxaliplatin dose).

The local effect of compound **23** or its vehicle was assessed 11 days after beginning the oxaliplatin treatment through subcutaneous intraplantar injection of 1 μg compound **23** or vehicle (2% Cremophor and 5% DMSO in saline) in the ventral side of the right hind paw, administered in a volume of 10 μL [66]. This dose was chosen based on previous works [48] and considering the relative potency of the compound in the *in vitro* studies. Nociceptive sensitivity was evaluated 30 and 90 min after the intraplantar injection with the acetone test [66].

#### Behavioral assessment of cold sensitivity

Prior to conducting behavioral experiments, mice underwent a 2‐day acclimatization period to the experimental conditions, during which they were handled and habituated to the male experimenter for a minimum of 2 min per day and mouse [66]. Additionally, the animals were familiarized with each testing environment 2 h per day, placed individually in Plexiglas® chambers (10 × 10 × 14 cm). Every day of evaluation, the mice spent an additional hour of habituation in the testing environment before the measurement of nociceptive sensitivity.

#### Acetone drop test

Mice were placed over a metal grid and allowed to habituate for approximately 1 h. Acetone (179124; Sigma‐Aldrich) was applied in 20 μL drops onto the mid‐plantar surface of the right hind paw by using a 200 μL pipette with a plastic tip manually curved [66]. Responses were recorded using an iPhone SE camera (Apple, Cupertino, CA, USA), and quantification of paw‐licking responses was conducted afterwards by a blinded observer. The responses were measured for 1 min after acetone application, with a digital stopwatch (Xnote Stopwatch Version 1.63 2011 Dmitry Nikitin) and were averaged for both hindpaws after the systemic treatment with compound **23** or conducted only in the right hind paw after the intraplantar treatment. For each measurement, the paws were sampled three times, and the mean was calculated. The interval between each application of acetone was at least 3 min [66].

#### Dry ice test

The dry ice test was conducted as previously described [67] with some modifications. Mice underwent a 1‐h habituation period on a 6 mm thick glass surface (Flores Valles, Madrid, Spain). Subsequently, a hand‐made probe was prepared by cutting the top of a 3 mL syringe (DicoNEX; ZARYS International Group, Zabrze, Poland) and drilling the syringe with a 25 G needle (BD Microlance 3; Beckton Dickinson & Co Ltd, Louth, Ireland) to prevent the accumulation of CO_2_ gas. Powdered dry ice was used to fill the probe. The ice was compacted by pressing the plunger against the bench until a dense pellet at least 1 cm long was obtained. The flattened pellet was then applied to the glass surface using the probe, targeting the right and left hindpaws. A minimum interval of 5 min was maintained between applications. To determine the threshold value for each mouse, the latency to paw withdrawal was recorded for each hindpaw and subsequently averaged. The glass surface was always kept dry.

#### Statistical analysis

For the analysis of *in vitro* studies, all data are expressed as mean ± SEM. The number of replicates is indicated in the figure legends. *In vitro* data were statistically analyzed using one‐way ANOVA followed by the Bonferroni *post hoc* test of multiple comparisons as indicated, or unpaired, two‐tailed Student *t*‐test for some experiments are also indicated. For one‐way ANOVA, we report the *F* (DFn, DFd) and the *P* value, along with the *P* values derived from the Bonferroni *post hoc* test. The *P* value for all the analyses was set at 0.05, and the value obtained is reported.

For the analysis of behavioral experiments, graphpad prism 9 was used (GraphPad Software Inc., San Diego, CA, USA). An ANOVA with two factors (Within Factor ‘OXP treatment’, between factor ‘**23** vs Vehicle group’) and their interaction was first used to assess the effect of the oxaliplatin treatment and to assess possible baseline differences between treatment groups. A subsequent two‐way ANOVA was used to study the effects of compound **23** treatment (Within Factor ‘Time Point’, between factor ‘**23** vs Vehicle group’ and their interaction). *Post hoc* Tukey's multiple comparisons tests were run whenever the interaction was significant and differences were considered statistically significant when *P* value was below 0.05.

### Metabolic stability

#### Incubation in mouse plasma

The standard incubation mixture (100 μL final volume) was carried out by dissolving the tested substrate (100 μm) in DMSO (5% final volume) in preincubated plasma at 37 °C. The mixture was shaken for 60 min at 37 °C. Control incubations were carried out without substrate. Each incubation was stopped by addition of 200 μL of ice‐cold acetonitrile, vortexed, and centrifuged at 13 000 r.p.m. for 10 min. The supernatants were analyzed by LC‐UV [68].

#### Incubation in mouse liver microsomes

The standard incubation mixture (250 μL final volume) was carried out in a 50 mm Tris (tris[hydroxymethyl]aminomethane) buffer (pH 7.4) containing 150 mm KCl, 1.5 mm, 3.3 mm MgCl_2_, 1.3 mm NADPNa_2_, 3.3 mm glucose 6‐phosphate, 0.4 units·mL^−1^ glucose 6‐phosphate dehydrogenase, acetonitrile as cosolvent (1% of total volume), and the substrate (50 μm). After pre‐equilibration of the mixture, an appropriate volume of MLM suspension was added to give a final protein concentration of 1.0 mg·mL^−1^. The mixture was shaken for 60 min at 37 °C using a horizontal shaking thermostatic bath while protecting the samples from light. Control incubations were carried out without the presence of substrate, or NADPH regenerating system, or microsomes. Each incubation was stopped by the addition of 250 μL of ice‐cold acetonitrile, vortexed, and centrifuged at 13 000 r.p.m. for 5 min [68]. The supernatants were analyzed by LC‐UV and LC‐HRMS equipment (see Tables S1 and S2, Fig. S79 for full parameters setting in the Supporting Information).

#### Data processing

The metabolic stability of the compound **23** was determined *in vitro* by measuring the residual peak area after incubation by LC‐UV analysis. Samples were further processed by LC‐HRMS equipment for metabolite profiling. Raw data files were processed using both xcalibur® and compound discoverer 3.2® software (Thermo Scientific) using a customized workflow for the detection and identification of the expected and unknown metabolites (Fig. S80).

#### Molecular modeling and docking

Human and mouse TRPM8 structures (PDB ID 8BDC, 8E4N) [4, 69] were used to explore the binding of compound **23**. The docking procedure was performed with the AutoDock4 algorithm [70] implemented in Yasara [71, 72]. Briefly, a local docking procedure was accomplished using either human or mouse TRPM8 structures, and compound **23**. The search space was limited to a simulation box built around the well‐known agonist or antagonist ligands (WS‐12, AMTB). A total of 500 flexible docking runs were set and clustered around the selected binding sites. The program performs a simulated annealing minimization of the complexes, which moves the structure to a stable energy minimum, by using the implemented AMBER 99 (Assisted Model Building with Energy Refinement) force field [73]. The Yasara pH command was set to 7.0, to ensure that molecules preserved their pH dependency of bond orders and protonation patterns. The best binding energy complex in each cluster was stored, analyzed, and used to select the best orientation of the interacting partners. The theoretical affinities of ligands at its binding site were determined by calculating the binding energy of the ligand‐receptor complex. The binding energy was obtained by measuring the energy at infinite distance (the unbound state) and subtracting from that value the energy of the complex at the bound state. Figures were drawn using the open‐source pymol v2.6 (The PyMOL Molecular Graphics System, at http://www.pymol.org/). Interactions were determined in PLIP (https://plip‐tool.biotec.tu‐dresden.de/plip‐web/plip/index), a fully automated web server [74] to identify noncovalent interactions between macromolecules and ligands. The same molecular modeling methodology was used by Lamberti *et al*. [75].

## Conflict of interest

The authors declare no conflict of interest.

## Author contributions

UG, AF‐M, GCT, and AF‐C wrote and revised the manuscript with input from all the authors, drafting the work or reviewing it critically for important intellectual content. UG and FT synthesized the compounds. SA performed metabolic stability, HRMS analysis, and purity of final compounds. AL performed the biological evaluation, patch‐clamp, and MEA experiments and wrote the manuscript. DC performed *in vivo* experiments and wrote and revised the manuscript. LB performed MEA experiments and revised the manuscript. GFB performed docking simulations. All the authors have approved the final version of the manuscript.

## Peer review

The peer review history for this article is available at https://www.webofscience.com/api/gateway/wos/peer‐review/10.1111/febs.70065.

## Supporting information


**Figs S1–S78.** ^1^H‐NMR, ^13^C‐NMR and HRMS spectra of final compounds **20–45**.
**Figs S79–S80.** LC‐UV methods for purity and metabolic stability evaluation and LC‐HRMS metabolic profile of compound **23** in mouse liver microsomes.
**Figs S81–S92.** Purity of selected active compounds.
**Tables S1–S2.** LC‐UV methods for purity and metabolic stability evaluation and LC‐HRMS metabolic profile of compound **23** in mouse liver microsomes.

## Data Availability

The authors confirm that the data supporting the findings of this study are available within the main text and the Supporting Information. The data that support the findings of this study are available from the corresponding authors upon reasonable request.

## References

[1] Kashio M & Tominaga M (2022) TRP channels in thermosensation. Curr Opin Neurobiol 75, 102591. 35728275 10.1016/j.conb.2022.102591

[2] Pertusa M , Solorza J & Madrid R (2023) Molecular determinants of TRPM8 function: key clues for a cool modulation. Front Pharmacol 14, 1213337. 37388453 10.3389/fphar.2023.1213337 PMC10301734

[3] Lolignier S , Gkika D , Andersson D , Leipold E , Vetter I , Viana F , Noël J & Busserolles J (2016) New insight in cold pain: role of ion channels, modulation, and clinical perspectives. J Neurosci 36, 1435–11439. 10.1523/JNEUROSCI.2327-16.2016 PMC6601718 27911746

[4] Palchevskyi S , Czarnocki‐Cieciura M , Vistoli G , Gervasoni S , Nowak E , Beccari AR , Nowotny M & Talarico C (2023) Structure of human TRPM8 channel. Commun Biol 6, 1065. 37857704 10.1038/s42003-023-05425-6 PMC10587237

[5] Peier AM , Moqrich A , Hergarden AC , Reeve AJ , Andersson DA , Story GM , Earley TJ , Dragoni I , Mcintyre P , Bevan S *et al*. (2002) A TRP channel that senses cold stimuli and menthol. Cell 108, 705–715. 11893340 10.1016/s0092-8674(02)00652-9

[6] Plaza‐Cayón A , González‐Muñiz R & Martín‐Martínez M (2022) Mutations of TRPM8 channels: unraveling the molecular basis of activation by cold and ligands. Med Res Rev 42, 2168–2203. 35976012 10.1002/med.21920 PMC9805079

[7] Khalil M , Alliger K , Weidinger C , Yerinde C , Wirtz S , Becker C & Engel MA (2018) Functional role of transient receptor potential channels in immune cells and epithelia. Front Immunol 9, 174. 29467763 10.3389/fimmu.2018.00174 PMC5808302

[8] Silverman HA , Chen A , Kravatz NL , Chavan SS & Chang EH (2020) Involvement of neural transient receptor potential channels in peripheral inflammation. Front Immunol 11, 590261. 33193423 10.3389/fimmu.2020.590261 PMC7645044

[9] Liu H , Liu Q , Hua L & Pan J (2018) Inhibition of transient receptor potential melastatin 8 alleviates airway inflammation and remodeling in a murine model of asthma with cold air stimulus. Acta Biochim Biophys Sin 50, 499–506. 29635321 10.1093/abbs/gmy033

[10] Bidaux G , Borowiec A‐S , Dubois C , Delcourt P , Schulz C , Vanden Abeele F , Lepage G , Desruelles E , Bokhobza A , Dewailly E *et al*. (2016) Targeting of short TRPM8 isoforms induces 4TM‐TRPM8‐dependent apoptosis in prostate cancer cells. Oncotarget 7, 29063–29080. 27074561 10.18632/oncotarget.8666 PMC5045378

[11] Ochoa SV , Casas Z , Albarracín SL , Sutachan JJ & Torres YP (2023) Therapeutic potential of TRPM8 channels in cancer treatment. Front Pharmacol 14, 1098448. 37033630 10.3389/fphar.2023.1098448 PMC10073478

[12] Pérez De Vega MJ , Gómez‐Monterrey I , Ferrer‐Montiel A & González‐Muñiz R (2016) Transient receptor potential melastatin 8 channel (TRPM8) modulation: cool entryway for treating pain and cancer. J Med Chem 59, 10006–10029. 27437828 10.1021/acs.jmedchem.6b00305

[13] Mahmoud O , Soares GB & Yosipovitch G (2023) Transient receptor potential channels and itch. Int J Mol Sci 24, 420. 10.3390/ijms24010420 PMC9820407 36613861

[14] Fakih D , Baudouin C , Réaux‐Le Goazigo A & Mélik Parsadaniantz S (2020) TRPM8: a therapeutic target for neuroinflammatory symptoms induced by severe dry eye disease. Int J Mol Sci 21, 8756. 33228217 10.3390/ijms21228756 PMC7699525

[15] Parra A , Madrid R , Echevarria D , Del Olmo S , Morenilla‐Palao C , Acosta MC , Gallar J , Dhaka A , Viana F & Belmonte C (2010) Ocular surface wetness is regulated by TRPM8‐dependent cold thermoreceptors of the cornea. Nat Med 16, 1396–1399. 21076394 10.1038/nm.2264

[16] Wirta DL , Senchyna M , Lewis AE , Evans DG , McLaurin EB , Ousler GW & Hollander DAA (2022) Randomized, vehicle‐controlled, phase 2b study of two concentrations of the TRPM8 receptor agonist AR‐15512 in the treatment of dry eye disease (COMET‐1). Ocul Surf 26, 166–173. 35970431 10.1016/j.jtos.2022.08.003

[17] Dussor G & Cao YQ (2016) TRPM8 and migraine. Headache 56, 1406–1417. 27634619 10.1111/head.12948 PMC5335856

[18] Alarcón‐Alarcón D , Cabañero D , de Andrés‐López J , Nikolaeva‐Koleva M , Giorgi S , Fernández‐Ballester G , Fernández‐Carvajal A & Ferrer‐Montiel A (2022) TRPM8 contributes to sex dimorphism by promoting recovery of normal sensitivity in a mouse model of chronic migraine. Nat Commun 13, 6304. 36272975 10.1038/s41467-022-33835-3 PMC9588003

[19] Wu B , Su X , Zhang W , Zhang YH , Feng X , Ji YH & Tan ZY (2021) Oxaliplatin depolarizes the IB4 – dorsal root ganglion neurons to drive the development of neuropathic pain through TRPM8 in mice. Front Mol Neurosci 14, 690858. 34149356 10.3389/fnmol.2021.690858 PMC8211750

[20] Aierken A , Xie YK , Dong W , Apaer A , Lin JJ , Zhao Z , Yang S , Xu ZZ & Yang F (2021) Rational design of a modality‐specific inhibitor of TRPM8 channel against oxaliplatin‐induced cold allodynia. Adv Sci 8, 2101717. 10.1002/advs.202101717 PMC8596132 34658162

[21] Weyer‐Menkhoff I & Lötsch J (2018) Human pharmacological approaches to TRP‐ion‐channel‐based analgesic drug development. Drug Discov Today 23, 2003–2012. 29969684 10.1016/j.drudis.2018.06.020

[22] Beccari AR , Gemei M , Monte ML , Menegatti N , Fanton M , Pedretti A , Bovolenta S , Nucci C , Molteni A , Rossignoli A *et al*. (2017) Novel selective, potent naphthyl TRPM8 antagonists identified through a combined ligand‐ and structure‐based virtual screening approach. Sci Rep 7, 10999. 28887460 10.1038/s41598-017-11194-0 PMC5591244

[23] Fernández‐Carvajal A , González‐Muñiz R , Fernández‐Ballester G & Ferrer‐Montiel A (2020) Investigational drugs in early phase clinical trials targeting thermotransient receptor potential (ThermoTRP) channels. Expert Opin Investig Drugs 29, 1209–1222. 10.1080/13543784.2020.1825680 32941080

[24] Horne DB , Biswas K , Brown J , Bartberger MD , Clarine J , Davis CD , Gore VK , Harried S , Horner M , Kaller MR *et al*. (2018) Discovery of TRPM8 antagonist (S)‐6‐(((3‐Fluoro‐4‐(trifluoromethoxy)phenyl)(3‐fluoropyridin‐2‐yl)methyl)carbamoyl)nicotinic acid (AMG 333), a clinical candidate for the treatment of migraine. J Med Chem 61, 8186–8201. 30148953 10.1021/acs.jmedchem.8b00518

[25] Andrews MD , Forselles KA , Beaumont K , Galan SRG , Glossop PA , Grenie M , Jessiman A , Kenyon AS , Lunn G , Maw G *et al*. (2015) Discovery of a selective TRPM8 antagonist with clinical efficacy in cold‐related pain. ACS Med Chem Lett 6, 419–424. 25893043 10.1021/ml500479v PMC4394344

[26] Wu S , Huang J , Gazzarrini S , He S , Chen L , Li J , Xing L , Li C , Chen L , Neochoritis CG *et al*. (2015) Isocyanides as influenza a virus subtype H5N1 wild‐type M2 channel inhibitors. ChemMedChem 10, 1837–1845. 26506405 10.1002/cmdc.201500318

[27] Liu J , Obando D , Liao V , Lifa T & Codd R (2011) The many faces of the adamantyl group in drug design. Eur J Med Chem 46, 1949–1963. 21354674 10.1016/j.ejmech.2011.01.047

[28] Wanka L , Iqbal K & Schreiner PR (2013) The lipophilic bullet hits the targets: medicinal chemistry of adamantane derivatives. Chem Rev 113, 3516–3604. 23432396 10.1021/cr100264t PMC3650105

[29] Grillaud M & Bianco A (2015) Multifunctional adamantane derivatives as new scaffolds for the multipresentation of bioactive peptides. J Pept Sci 21, 330–345. 25448731 10.1002/psc.2719

[30] Dömling A , Wang W & Wang K (2012) Chemistry and biology of multicomponent reactions. Chem Rev 112, 3083–3135. 22435608 10.1021/cr100233r PMC3712876

[31] Zhu J , Wang Q & Wang MX (2014) Multicomponent Reactions in Organic Synthesis. Wiley, Weinheim.

[32] Hulme C & Gore V (2003) Multi‐component reactions: emerging chemistry in drug discovery ‘from xylocain to crixivan’. Curr Med Chem 10, 51–80. 12570721 10.2174/0929867033368600

[33] Ruijter E & Orru RVA (2013) Multicomponent reactions – opportunities for the pharmaceutical industry. Drug Discov Today Technol 10, e15–e20. 24050225 10.1016/j.ddtec.2012.10.012

[34] Ugi I , Meyr U , Fetzer U & Steinbrückner C (1959) Versuche mit isonitrilen. Angew Chem 71, 386–388.

[35] Dömling A & Ugi I (2000) Multicomponent reactions with isocyanides. Angew Chem 39, 3168–3210. 11028061 10.1002/1521-3773(20000915)39:18<3168::aid-anie3168>3.0.co;2-u

[36] Ugi I & Werner B (2007) Methods and Reagents for Green Chemistry: An Introduction ( Tundo P , Perosa A & Zecchini F , eds), pp. 1–22. Wiley, Hoboken, NJ. doi: 10.1002/9780470124086

[37] Flores‐Reyes JC , Islas‐Jácome A & González‐Zamora E (2021) The Ugi three‐component reaction and its variants. Org Chem Front 8, 5460–5515.

[38] Passerini M (1921) Isonitrili, I. Composti del p‐isonitrileazobenzene con acetone ed acido acetico. Gazz Chim Ital 51, 126–129.

[39] Banfi L , Basso A , Lambruschini C , Moni L & Riva R (2021) The 100 facets of the Passerini reaction. Chem Sci 12, 15445–15472. 35003575 10.1039/d1sc03810a PMC8654045

[40] Pirali T , Galli U , Serafini M , Griglio A , Genazzani AA & Tron GC (2019) Drug discovery for soft drugs on TRPV1 and TRPM8 channels using the Passerini reaction. In TRP Channels: Methods and Protocols, *Methods in Molecular Biology* ( Ferrer‐Montiel A & Hucho T , eds), 1987, pp. 207–221. Springer, New York, NY. 10.1007/978-1-4939-9446-5_13 31028682

[41] Banfi L & Riva R (2005) The Passerini reaction. In Organic Reactions, pp. 1–140. Wiley, Hoboken, NJ. doi: 10.1002/0471264180.or065.01

[42] Giovenzana GB , Tron GC , Di Paola S , Menegotto IG & Pirali T (2006) A mimicry of primary amines by bis‐secondary diamines as components in the Ugi four‐component reaction. Angew Chem Int Ed 45, 1099–1102. 10.1002/anie.200503095 16389629

[43] Galli U , Tron GC , Purghè B , Grosa G & Aprile S (2020) Metabolic fate of the isocyanide moiety: are isocyanides pharmacophore groups neglected by medicinal chemists? Chem Res Toxicol 33, 955–966. 32212628 10.1021/acs.chemrestox.9b00504

[44] Voets T , Talavera K , Owsianik G & Nilius B (2005) Sensing with TRP channels. Nat Chem Biol 1, 85–92. 16408004 10.1038/nchembio0705-85

[45] Eid SR , Crown ED , Moore EL , Liang HA , Choong KC , Dima S , Henze DA , Kane SA & Urban MO (2008) HC‐030031, a TRPA1 selective antagonist, attenuates inflammatory‐ and neuropathy‐induced mechanical hypersensitivity. Mol Pain 4, 48. 18954467 10.1186/1744-8069-4-48 PMC2584039

[46] Takaishi M , Uchida K , Suzuki Y , Matsui H , Shimada T , Fujita F & Tominaga M (2016) Reciprocal effects of capsaicin and menthol on thermosensation through regulated activities of TRPV1 and TRPM8. J Physiol Sci 66, 143–155. 26645885 10.1007/s12576-015-0427-y PMC4752590

[47] Bertamino A , Ostacolo C , Medina A , Di Sarno V , Lauro G , Ciaglia T , Vestuto V , Pepe G , Basilicata MG , Musella S *et al*. (2020) Exploration of TRPM8 binding sites by β‐carboline‐based antagonists and their in vitro characterization and in vivo analgesic activities. J Med Chem 63, 9672–9694. 32787109 10.1021/acs.jmedchem.0c00816 PMC8009520

[48] Iraci N , Ostacolo C , Medina‐Peris A , Ciaglia T , Novoselov AM , Altieri A , Cabañero D , Fernández‐Carvajal A , Campiglia P , Gómez‐Monterrey I *et al*. (2022) In vitro and in vivo pharmacological characterization of a novel TRPM8 inhibitor chemotype identified by small‐scale preclinical screening. Int J Mol Sci 23, 2070. 35216186 10.3390/ijms23042070 PMC8877448

[49] Martín‐Escura C , Bonache MÁ , Medina JA , Medina‐Peris A , De Andrés‐López J , González‐Rodríguez S , Kerselaers S , Fernández‐Ballester G , Voets T , Ferrer‐Montiel A *et al*. (2023) β‐Lactam TRPM8 antagonists derived from phe‐phenylalaninol conjugates: structure–activity relationships and antiallodynic activity. Int J Mol Sci 24, 14894. 37834342 10.3390/ijms241914894 PMC10573892

[50] Gandhi AS , Wohlfarth A , Zhu M , Pang S , Castaneto M , Scheidweiler KB & Huestis MA (2015) High‐resolution mass spectrometric metabolite profiling of a novel synthetic designer drug, N‐(adamantan‐1‐yl)‐1‐(5‐fluoropentyl)‐1H‐indole‐3‐carboxamide (STS‐135), using cryopreserved human hepatocytes and assessment of metabolic stability with human liver microsomes. Drug Test Anal 7, 187–198. 24827428 10.1002/dta.1662 PMC4232487

[51] Gandhi AS , Zhu M , Pang S , Wohlfarth A , Scheidweiler KB , Liu H & Huestis MA (2013) First characterization of AKB‐48 metabolism, a novel synthetic cannabinoid, using human hepatocytes and high‐resolution mass spectrometry. AAPS J 15, 1091–1098. 23913126 10.1208/s12248-013-9516-0 PMC3787239

[52] Lelis Carvalho A , Treyball A , Brooks DJ , Costa S , Neilson RJ , Reagan MR , Bouxsein ML & Motyl KJ (2021) TRPM8 modulates temperature regulation in a sex‐dependent manner without affecting cold‐induced bone loss. PLoS One 16, e0231060. 34086678 10.1371/journal.pone.0231060 PMC8177490

[53] Asuthkar S , Velpula KK , Elustondo PA , Demirkhanyan L & Zakharian E (2015) TRPM8 channel as a novel molecular target in androgen‐regulated prostate cancer cells. Oncotarget 6, 17221–17236. 25980497 10.18632/oncotarget.3948 PMC4627303

[54] Zhang L & Barritt GJ (2004) Evidence that TRPM8 is an androgen‐dependent Ca^2+^ channel required for the survival of prostate cancer cells. Cancer Res 64, 8365–8373. 15548706 10.1158/0008-5472.CAN-04-2146

[55] Brunelli F , Ceresa C , Fracchia L , Tron GC & Aprile S (2022) Expanding the chemical space of drug‐like Passerini compounds: can α‐acyloxy carboxamides be considered hard drugs? ACS Med Chem Lett 13, 1898–1904. 36518692 10.1021/acsmedchemlett.2c00420 PMC9743426

[56] Nikolaeva‐Koleva M , Butron L , González‐Rodríguez S , Devesa I , Valente P , Serafini M , Genazzani AA , Pirali T , Fernández‐Ballester G , Fernández‐Carvajal A *et al*. (2021) A capsaicinoid‐based soft drug, AG1529, for attenuating TRPV1‐mediated histaminergic and inflammatory sensory neuron excitability. Sci Rep 11, 246. 33420359 10.1038/s41598-020-80725-z PMC7794549

[57] Diaz‐Franulic I , Poblete H , Miño‐Galaz G , González C & Latorre R (2016) Allosterism and structure in thermally activated transient receptor potential channels. Annu Rev Biophys 45, 371–398. 27297398 10.1146/annurev-biophys-062215-011034

[58] Janssens A & Voets T (2011) Ligand stoichiometry of the cold‐ and menthol‐activated channel TRPM8. J Physiol 589(Pt 20), 4827–4835. 21878524 10.1113/jphysiol.2011.216523 PMC3224877

[59] Garami A , Shimansky YP , Pakai E , Oliveira DL , Gavva NR & Romanovsky AA (2010) Contributions of different modes of TRPV1 activation to TRPV1 antagonist‐induced hyperthermia. J Neurosci 30, 1435–1440. 20107070 10.1523/JNEUROSCI.5150-09.2010 PMC2824913

[60] Williamson JM & Lothman EW (1989) The effect of MK‐801 on kindled seizures: implications for use and limitations as an antiepileptic drug. Ann Neurol 26, 85–90. 2549848 10.1002/ana.410260113

[61] Winchester WJ , Gore K , Glatt S , Petit W , Gardiner JC , Conlon K , Postlethwaite SPP , Roberts S , Gosset JR , Matsuura T *et al*. (2014) Inhibition of TRPM8 channels reduces pain in the cold pressor test in humans. J Pharm Exp Ther 351, 259–269. 10.1124/jpet.114.216010 25125580

[62] Shulman RJ , Chumpitazi BP , Abdel‐Rahman SM , Garg U , Musaad S & Kearns GL (2022) Randomised trial: peppermint oil (menthol) pharmacokinetics in children and effects on gut motility in children with functional abdominal pain. Br J Clin Pharmacol 88, 1321–1333. 34528282 10.1111/bcp.15076 PMC8863319

[63] Singh R , Adhya P & Sharma SS (2021) Redox‐sensitive TRP channels: a promising pharmacological target in chemotherapy‐induced peripheral neuropathy. Expert Opin Ther Targets 25, 529–545. 34289785 10.1080/14728222.2021.1956464

[64] Zimmermann M (1983) Ethical guidelines for investigations of experimental pain in conscious animals. Pain 16, 109–110. 6877845 10.1016/0304-3959(83)90201-4

[65] Bonache MÁ , Martín‐Escura C , de la Torre Martínez R , Medina A , González‐Rodríguez S , Francesch A , Cuevas C , Roa AM , Fernández‐Ballester G , Ferrer‐Montiel A *et al*. (2020) Highly functionalized β‐lactams and 2‐ketopiperazines as TRPM8 antagonists with antiallodynic activity. Sci Rep 10, 14154. 32843690 10.1038/s41598-020-70691-x PMC7447632

[66] Martín‐Escura C , Bonache MA , Medina‐Peris A , Voets T , Ferrer‐Montiel A , Fernández‐Carvajal A & González‐Muñiz R (2025) Phenylalanine‐derived β‐lactam TRPM8 antagonists: revisiting configuration and new benzoyl derivatives. Explor Drug Sci 3, 100882.

[67] Brenner DS , Golden JP & Gereau RW (2012) A novel Behavioral assay for measuring cold sensation in mice. PLoS One 7, e39765. 22745825 10.1371/journal.pone.0039765 PMC3382130

[68] Brunelli F , Ceresa C , Aprile S , Coppo L , Castiglioni B , Bosetti M , Fracchia L & Tron GC (2023) Isocyanides in med chem: a scaffold hopping approach for the identification of novel 4‐isocyanophenylamides as potent antibacterial agents against methicillin‐resistant *Staphylococcus aureus* . Eur J Med Chem 246, 114950. 36462437 10.1016/j.ejmech.2022.114950

[69] Yin Y , Zhang F , Feng S , Butay KJ , Borgnia MJ , Im W & Lee SY (2022) Activation mechanism of the mouse cold‐sensing TRPM8 channel by cooling agonist and PIP2. Science 378, eadd1268. 36227998 10.1126/science.add1268 PMC9795508

[70] Morris GM , Huey R , Lindstrom W , Sanner MF , Belew RK , Goodsell DS & Olson AJ (2009) AutoDock4 and AutoDockTools4: automated docking with selective receptor flexibility. J Comput Chem 30, 2785–2791. 19399780 10.1002/jcc.21256 PMC2760638

[71] Krieger E & Vriend G (2014) YASARA view—molecular graphics for all devices—from smartphones to workstations. Bioinformatics 30, 2981–2982. 24996895 10.1093/bioinformatics/btu426 PMC4184264

[72] Ozvoldik K , Stockner T & Krieger E (2023) YASARA model–interactive molecular modeling from two dimensions to virtual realities. J Chem Inf Model 63, 6177–6182. 37782001 10.1021/acs.jcim.3c01136 PMC10598798

[73] Duan Y , Wu C , Chowdhury S , Lee MC , Xiong G , Zhang W , Yang R , Cieplak P , Luo R , Lee T *et al*. (2003) A point‐charge force field for molecular mechanics simulations of proteins based on condensed‐phase quantum mechanical calculations. J Comput Chem 24, 1999–2012. 14531054 10.1002/jcc.10349

[74] Adasme MF , Linnemann KL , Bolz SN , Kaiser F , Salentin S , Haupt VJ & Schroeder M (2021) Expanding the scope of the protein–ligand interaction profiler to DNA and RNA. Nucleic Acids Res 49, W530–W534. 33950214 10.1093/nar/gkab294 PMC8262720

[75] Lamberti A , Serafini M , Aprile S , Bhela IP , Goutsiou G , Pessolano E , Fernández‐Ballester G , Ferrer‐Montiel A , Di Martino RMC , Fernández‐Carvajal A *et al*. (2024) The multicomponent Passerini reaction as a means of accessing diversity in structure, activity and properties: soft and hard Vanilloid/cannabinoid modulators. Eur J Med Chem 279, 116845. 39265249 10.1016/j.ejmech.2024.116845

