# Supplementary material for: An adamantane‐based ligand as a novel chemical tool for thermosensory TRPM8 channel therapeutic modulation
Source: FEBS J. 2025 Mar 23;292(13):3449–76. doi: 10.1111/febs.70065 (PMC12220852; doi:10.1111/febs.70065)
Supplement: Supplementary file 1 — Figs S1–S78. 1H‐NMR, 13C‐NMR and HRMS spectra of final compounds 20–45. Figs S79–S80. LC‐UV methods for purity and metabolic stability evaluation and LC‐HRMS metabolic profile of compound 23 in mouse liver microsomes. Figs S81–S92. Purity of selected active compounds. Tables S1–S2. LC‐UV methods for purity and metabolic stability evaluation and LC‐HRMS metabolic profile of compound 23 in mouse liver microsomes. [file FEBS-292-3449-s001.pdf]

# SUPPORTING INFORMATION

## An adamantane-based ligand as a novel chemical tool for thermosensory TRPM8 channel therapeutic modulation

Angela Lamberti[1, §], Silvio Aprile [2, §], David Cabañero[1], Fabio Travagin[2], Laura Butron[1], Gregorio Fernández-Ballester[1], Gian Cesare Tron[2], Asia Fernández-Carvajal[1], Antonio Ferrer-Montiel[1]*, Ubaldina Galli[2]*

[1] *Instituto de Investigación, Desarrollo e Innovación en Biotecnología Sanitaria de Elche (IDiBE), Universitad Miguel Hernández, Elche, Spain.*

[2] *Department of Pharmaceutical Sciences, Università degli Studi del Piemonte Orientale, Largo Donegani 2, 28100 Novara, Italy.*

## Contents

$^1$H-NMR, $^{13}$C-NMR and HRMS spectra of final compounds **20-45**. p. S2

LC-UV method for purity and metabolic stability evaluation. p. S54

LC-HRMS metabolic profile of compound **23** in mouse liver microsomes. p. S55

Purity evaluation of selected compounds by HPLC-UV analysis. p. S69

## $^{1}$H-NMR, $^{13}$C-NMR and HRMS spectra of compounds 20-45

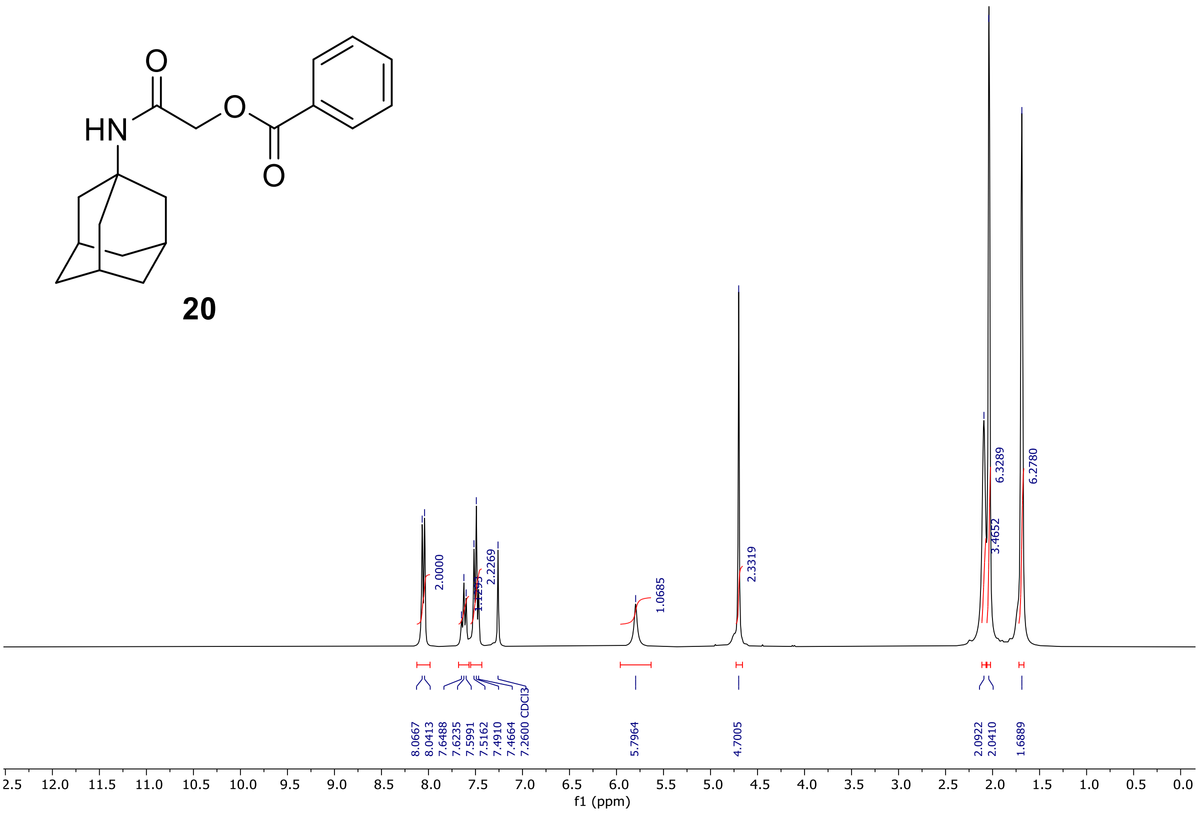


**Figure S1**. $^{1}$H NMR spectrum of compound **20**.

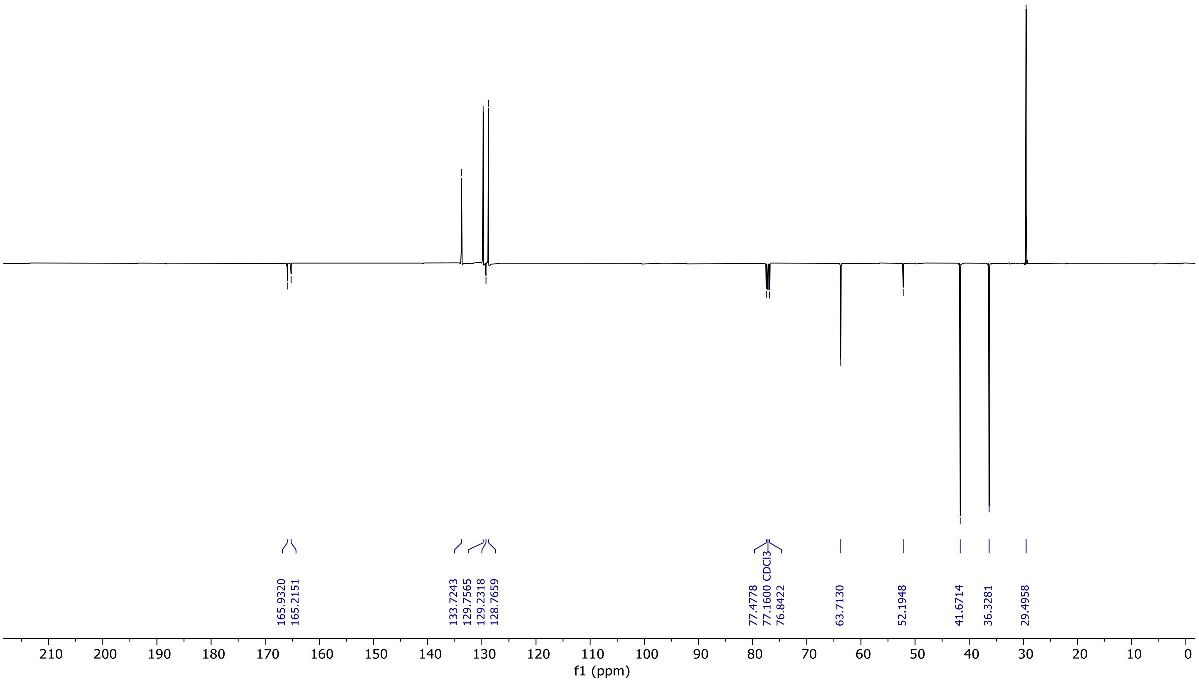


**Figure S2**. $^{13}$C APT NMR spectrum of compound **20**.

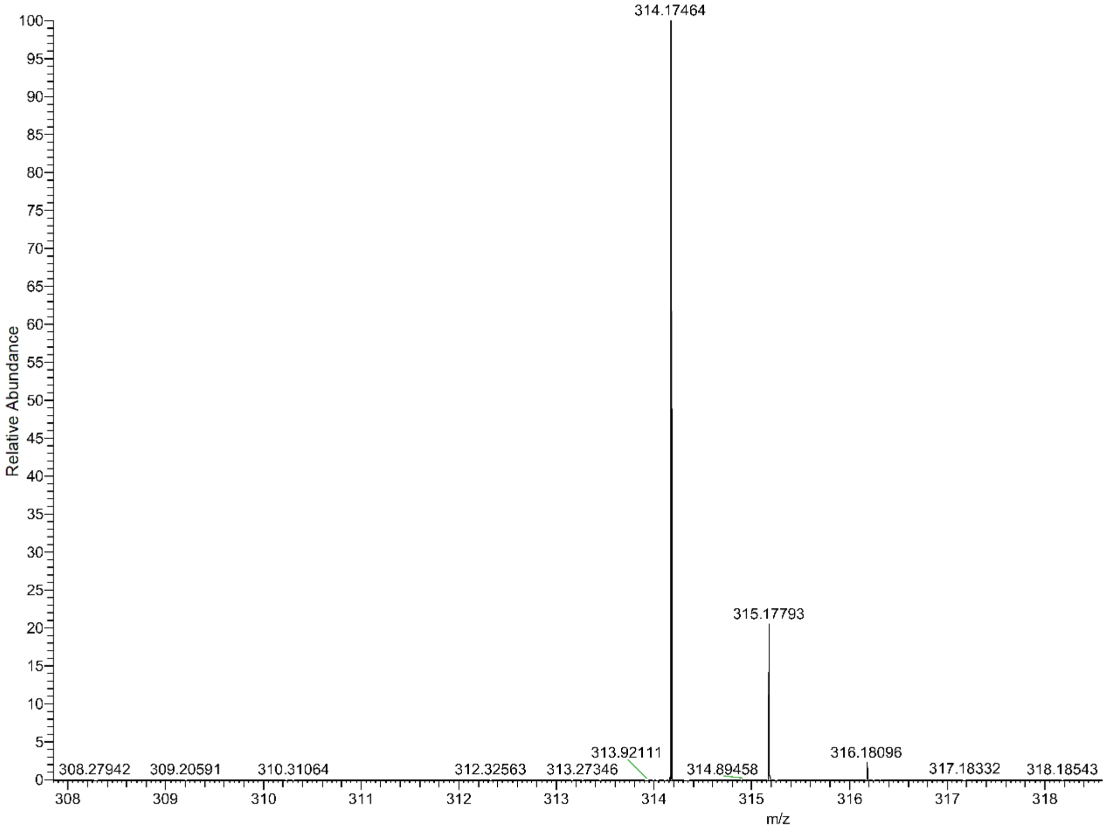


**Figure S3**. HRMS spectrum of compound **20**.

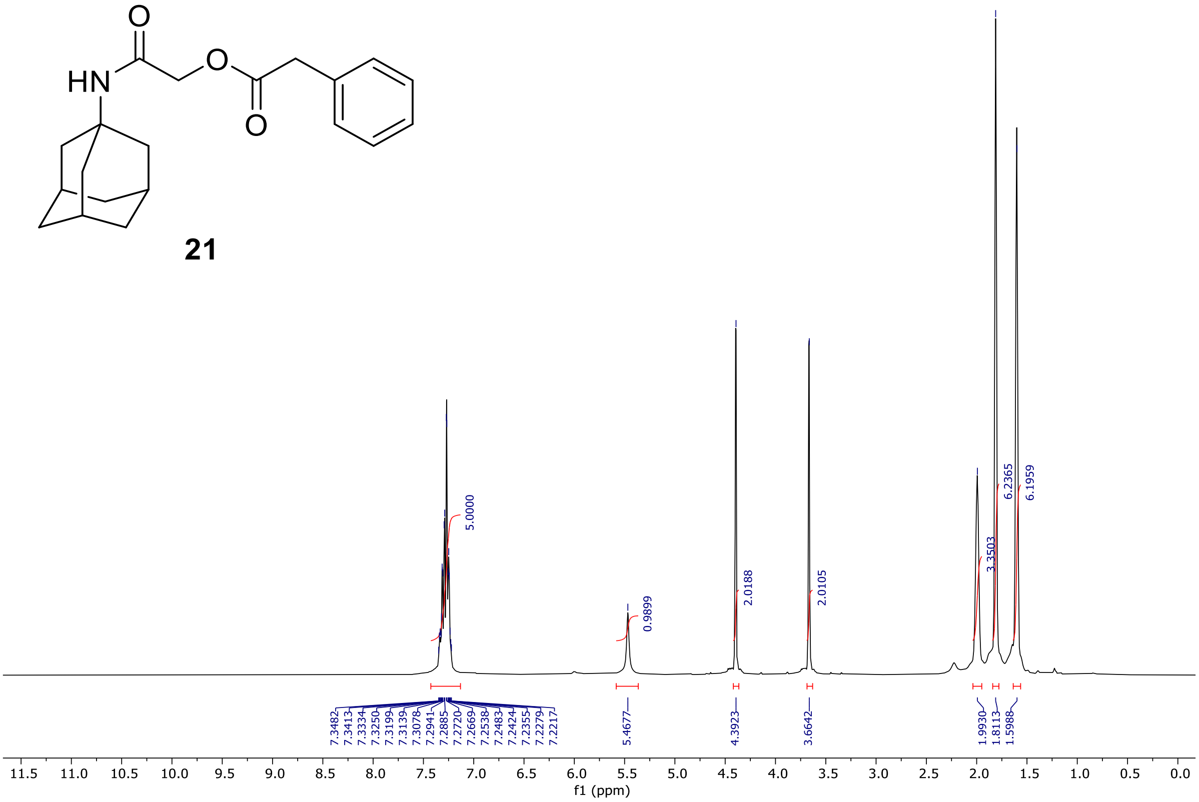


**Figure S4**. $^{1}H$ NMR spectrum of compound **21**.

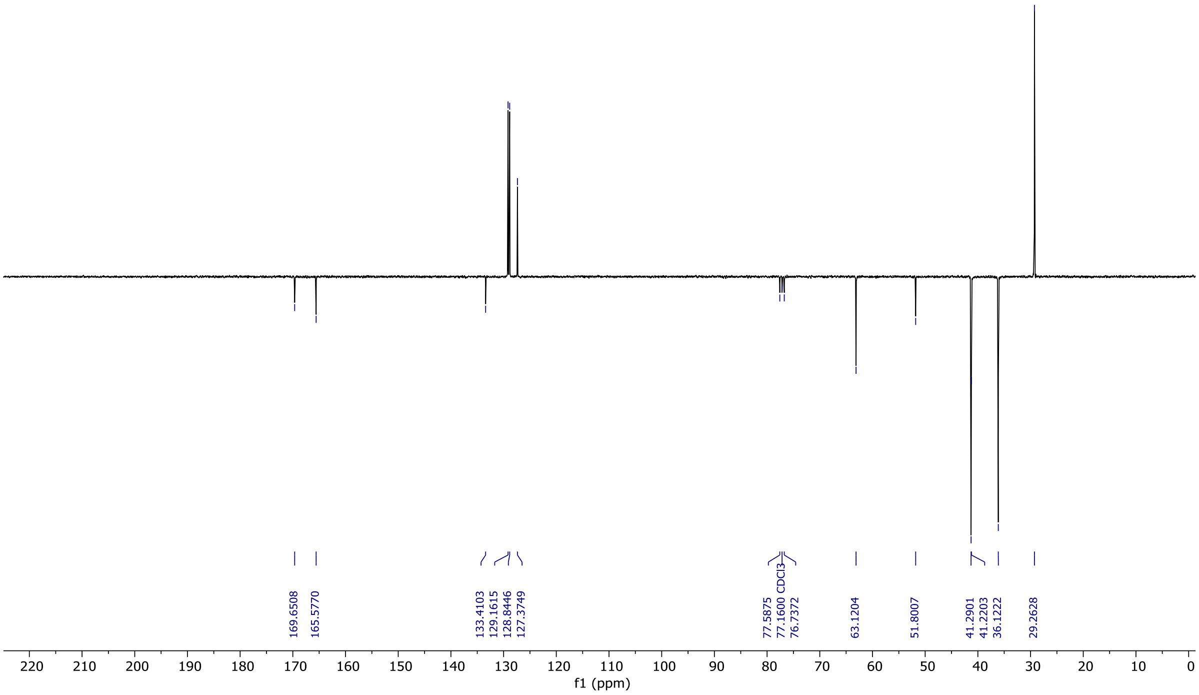


**Figure S5**. $^{13}C$ APT NMR spectrum of compound **21**.

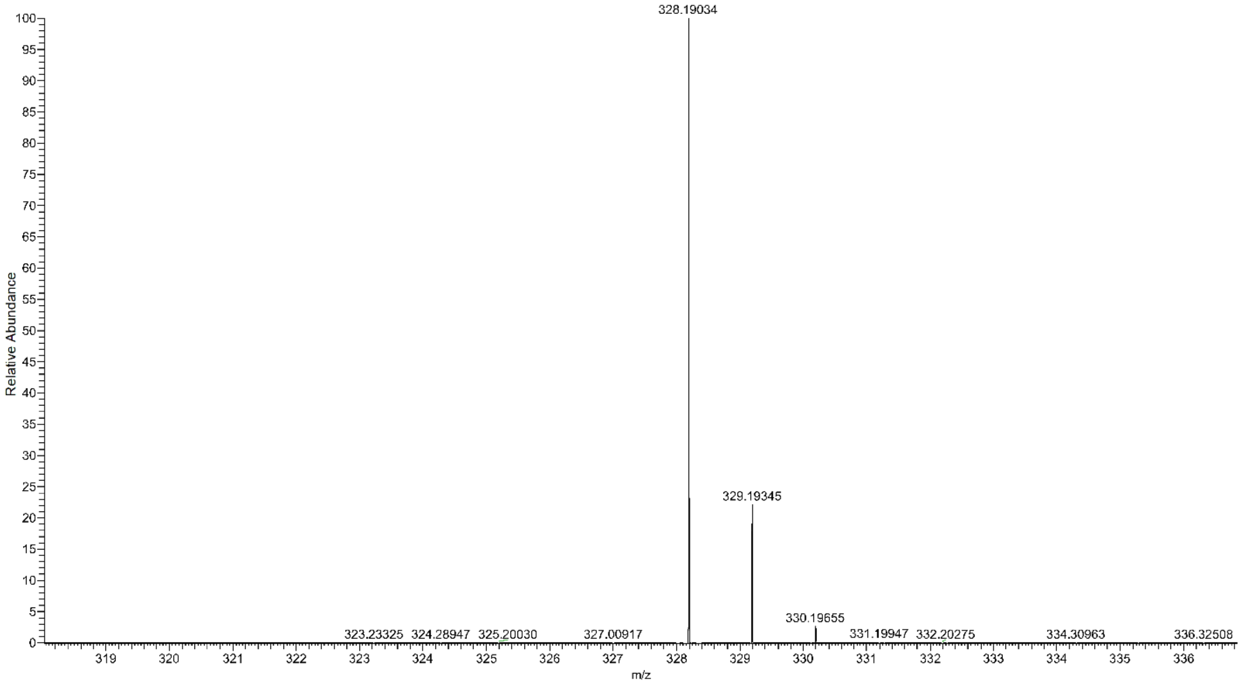

**Figure S6**. HRMS spectrum of compound **21**.

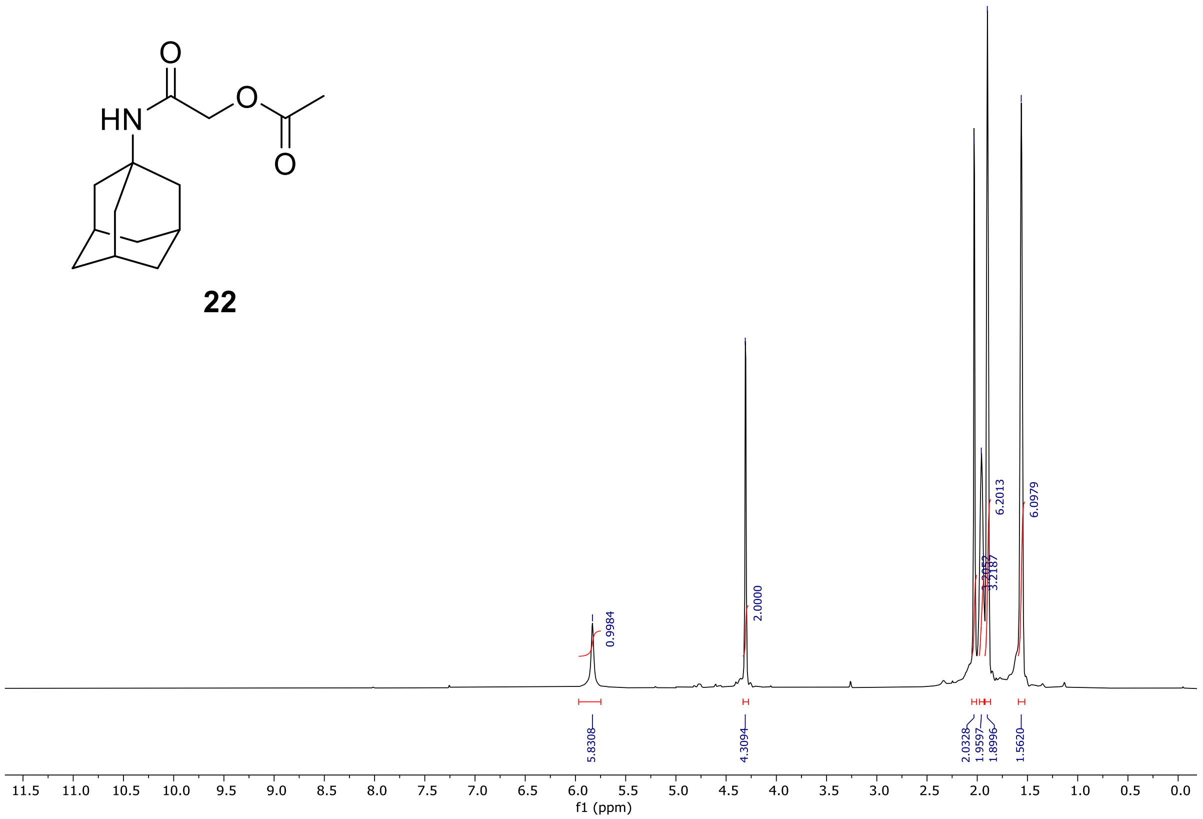


**Figure S7**. $^{1}$H NMR spectrum of compound **22**.

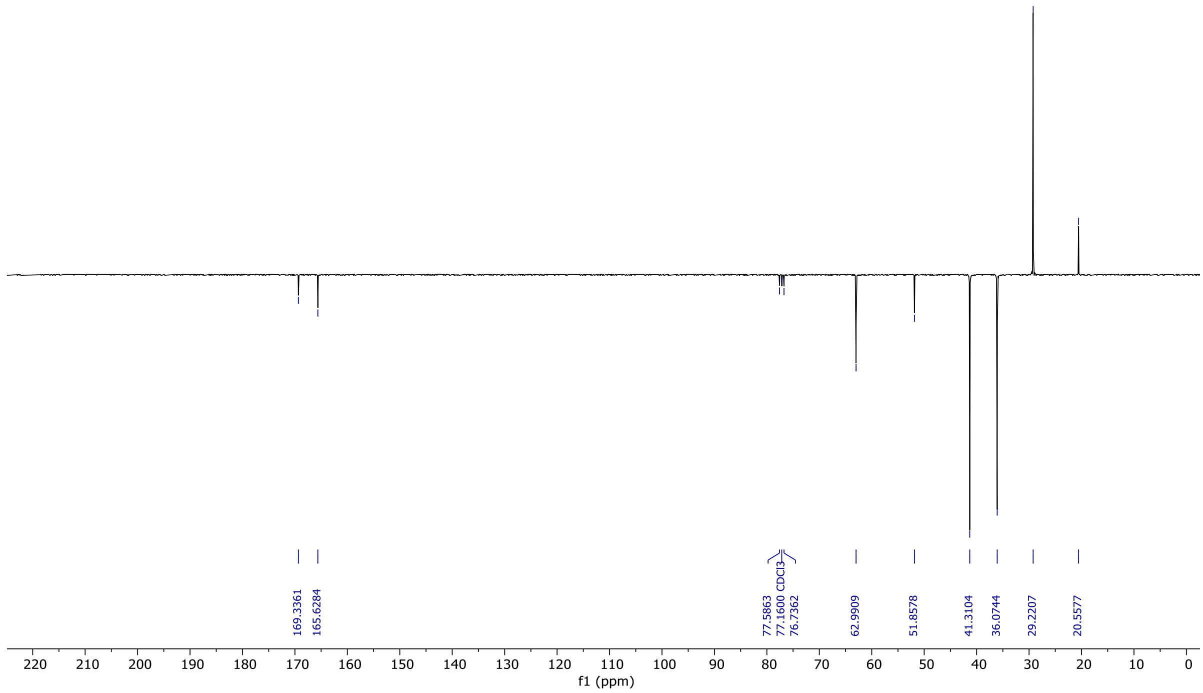


**Figure S8**. $^{13}$C APT NMR spectrum of compound **22**.

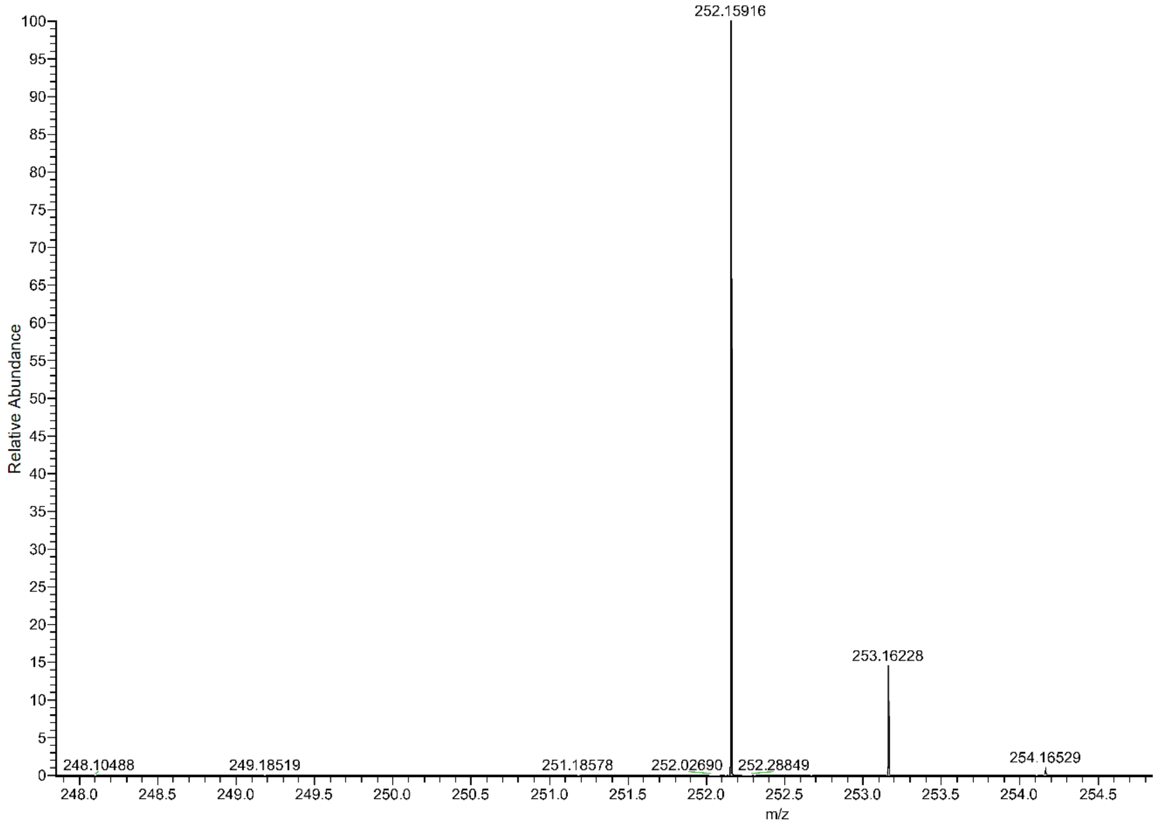


**Figure S9**. HRMS spectrum of compound **22**.

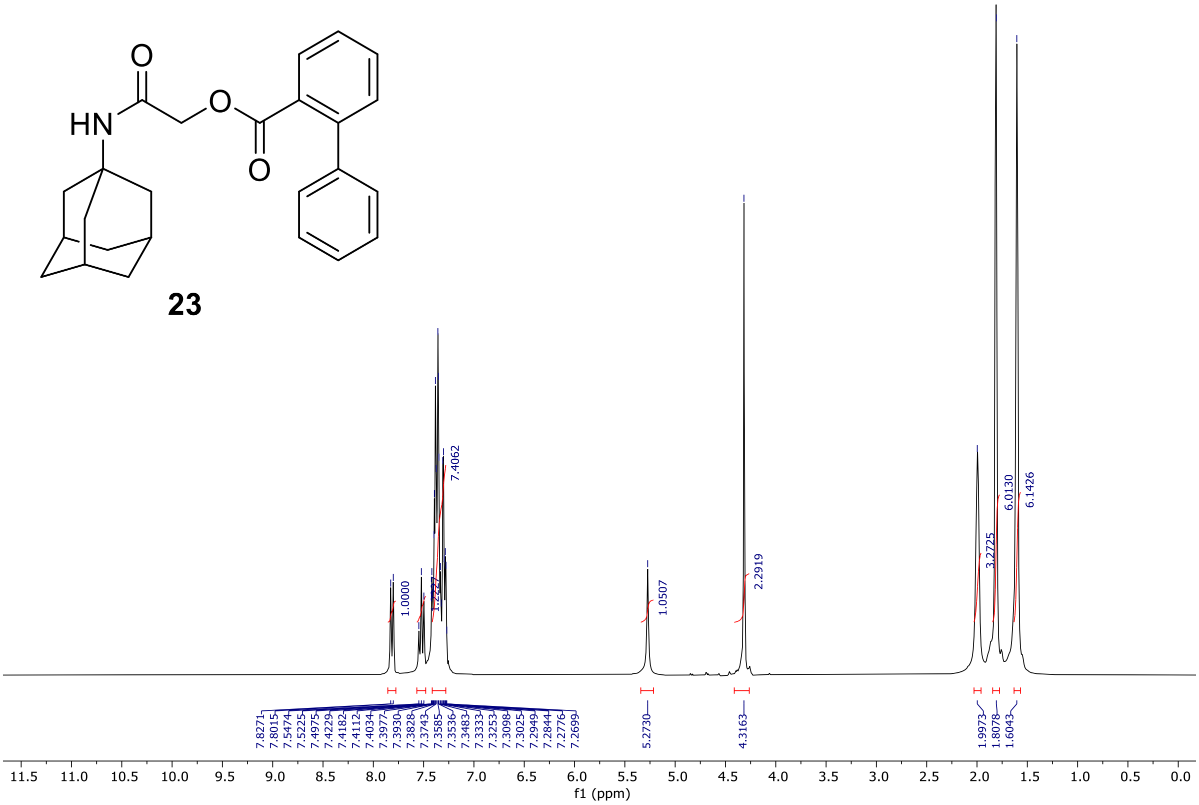


**Figure S10**. $^{1}$H NMR spectrum of compound **23**.

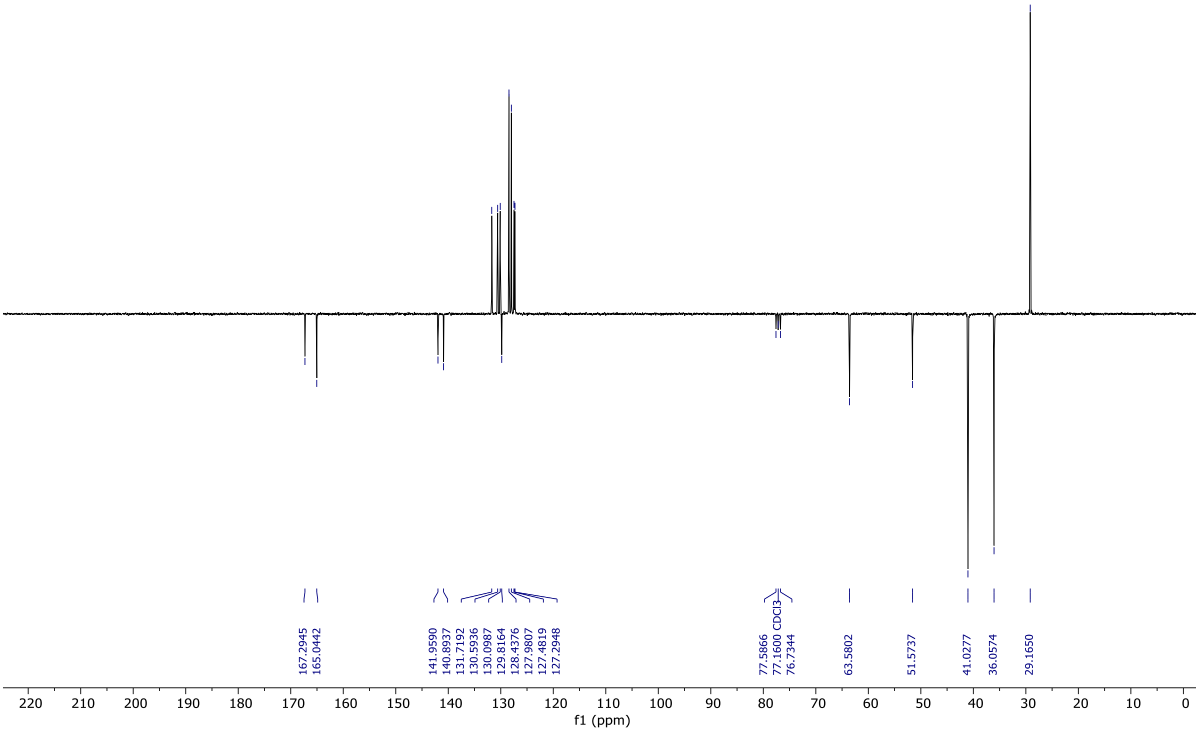


**Figure S11**. $^{13}$C APT NMR spectrum of compound **23**.

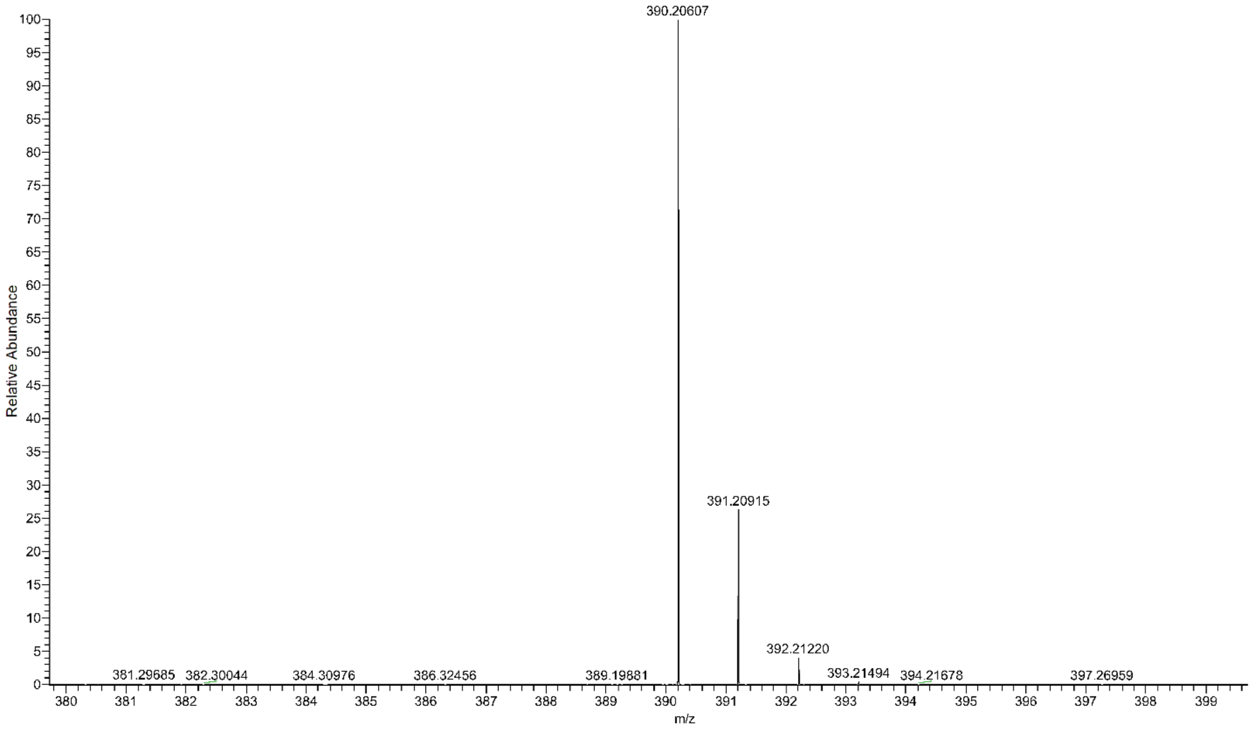


**Figure S12**. HRMS spectrum of compound **23**.

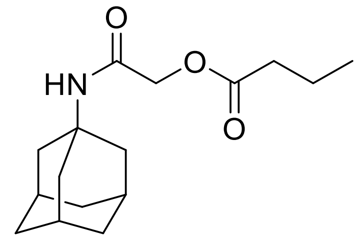

**24**

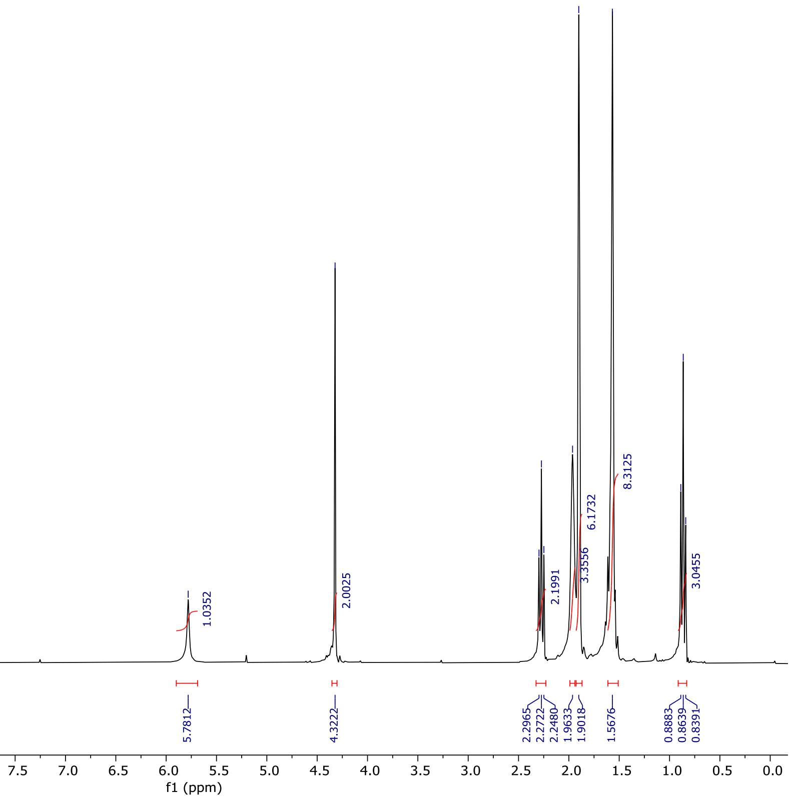


**Figure S13**. $^{1}$H NMR spectrum of compound **24**.

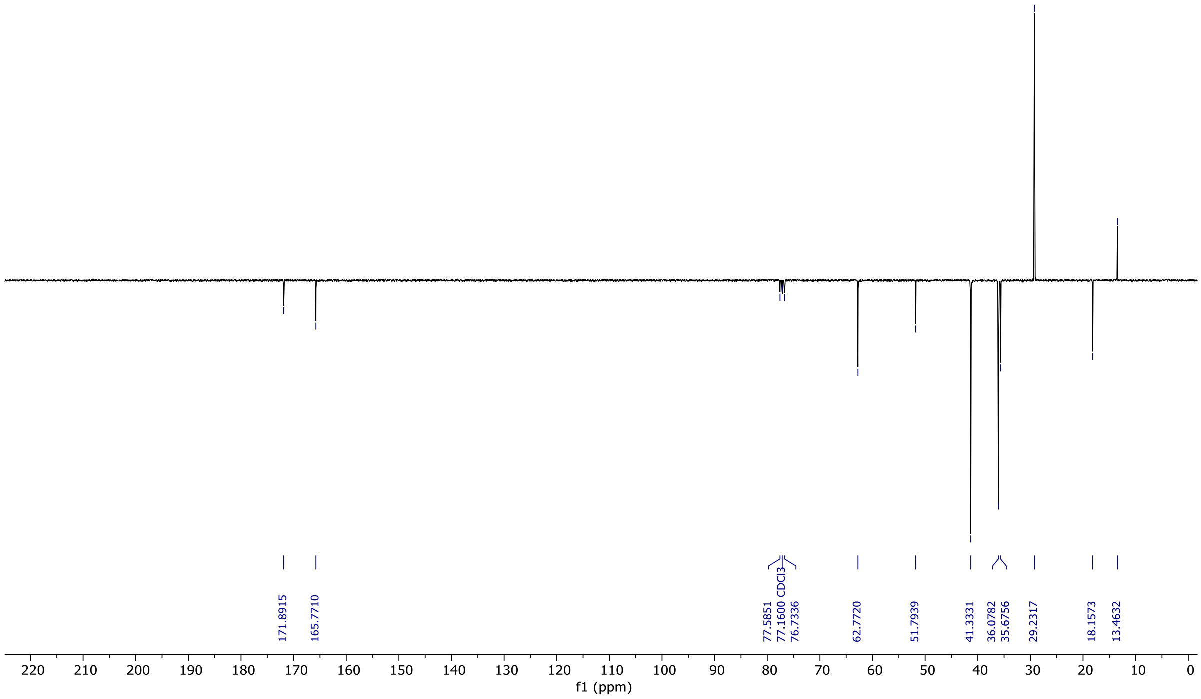


**Figure S14**. $^{13}$C APT NMR spectrum of compound **24**.

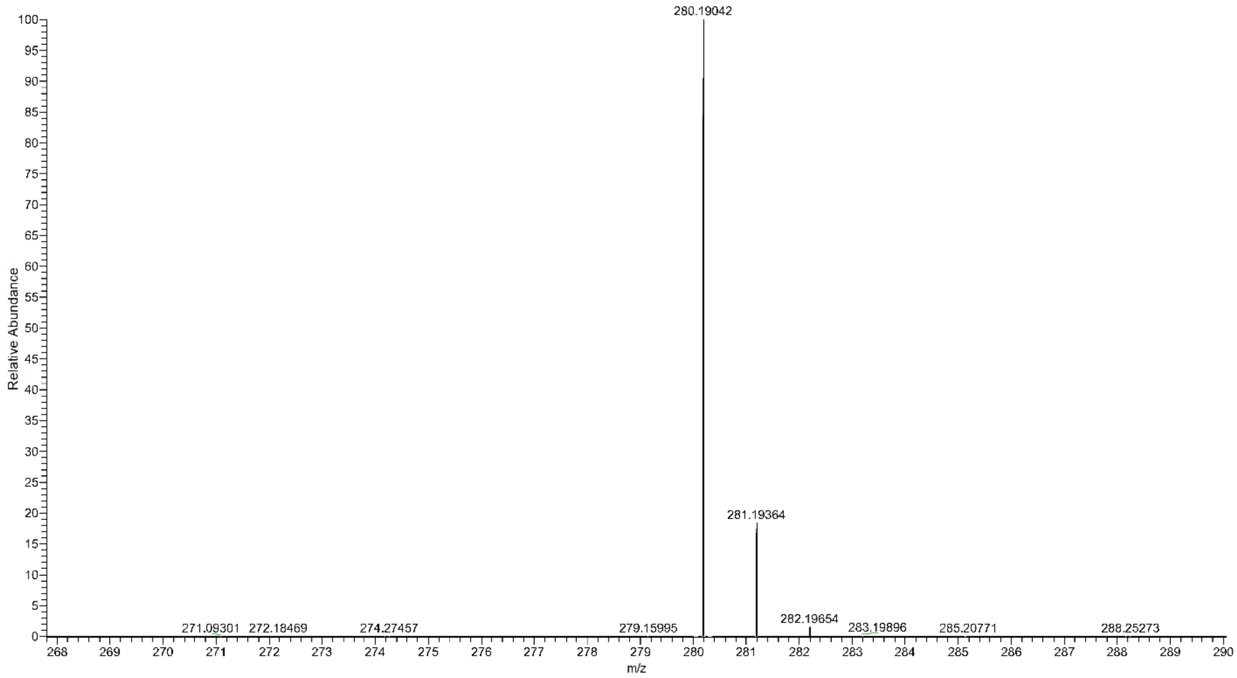


**Figure S15**. HRMS spectrum of compound **24**.

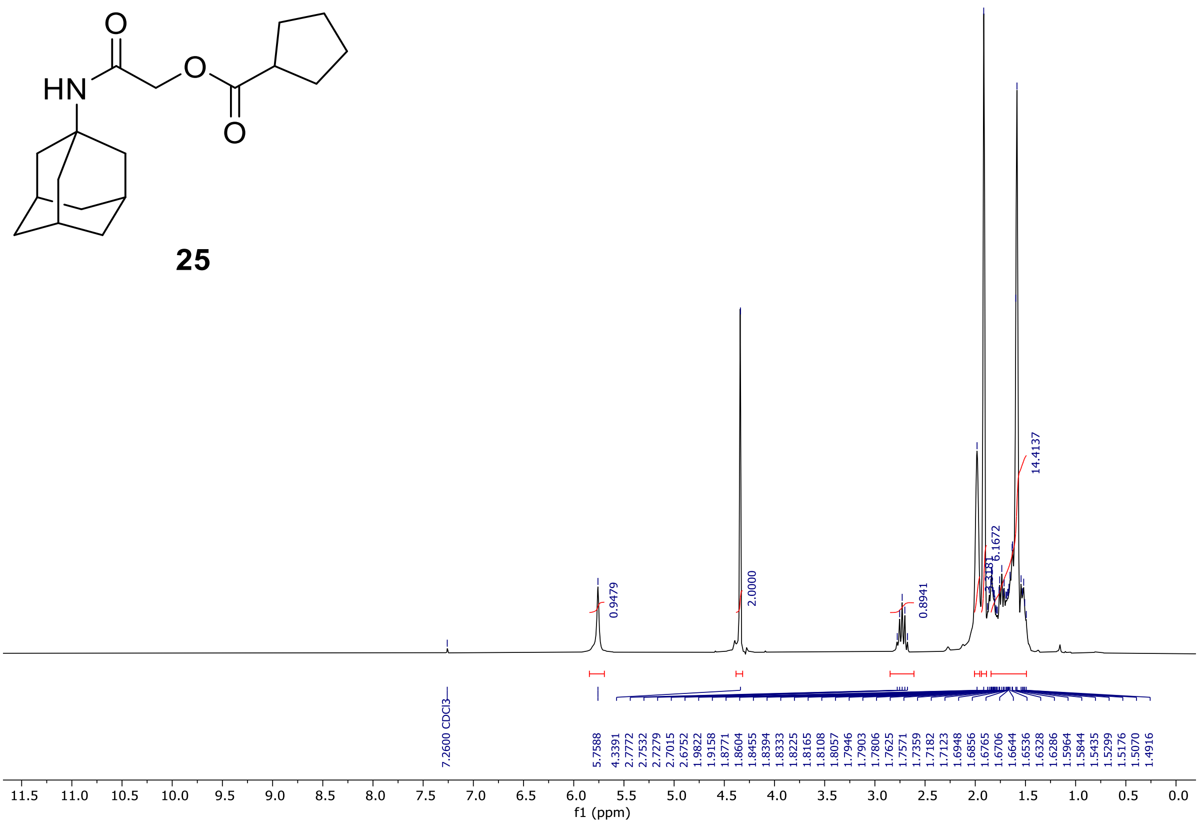


**Figure S16**. $^{1}$H NMR spectrum of compound **25**.

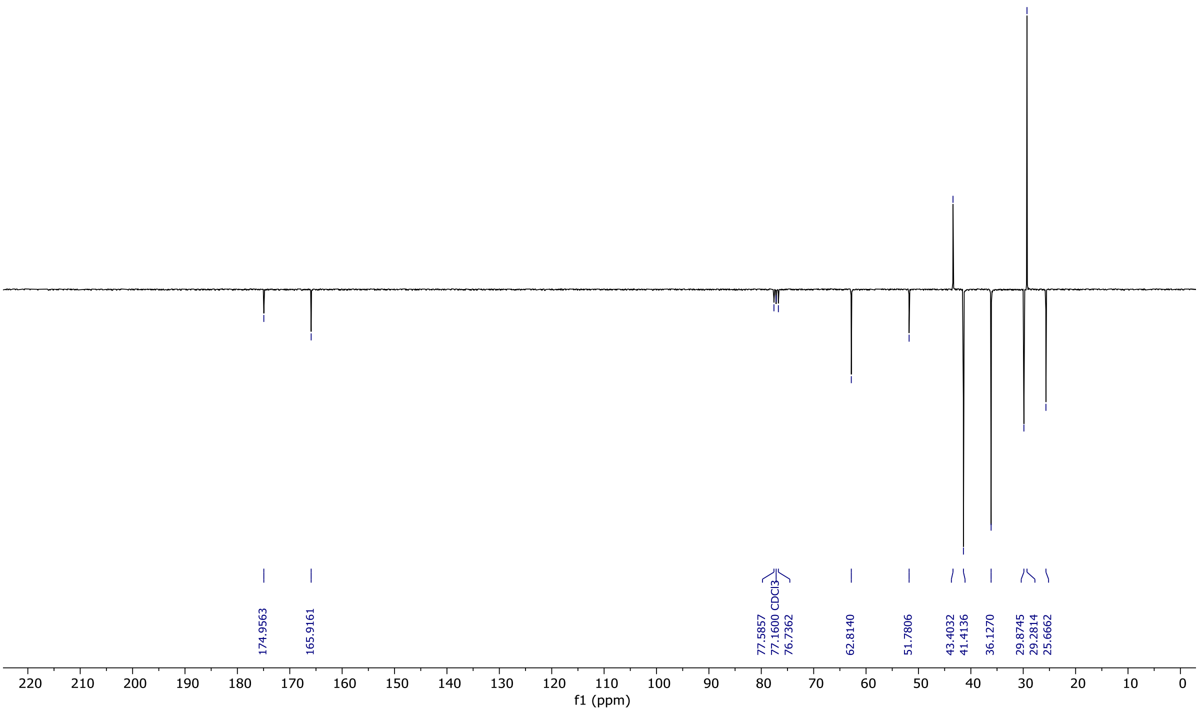


**Figure S17**. $^{13}$C APT NMR spectrum of compound **25**.

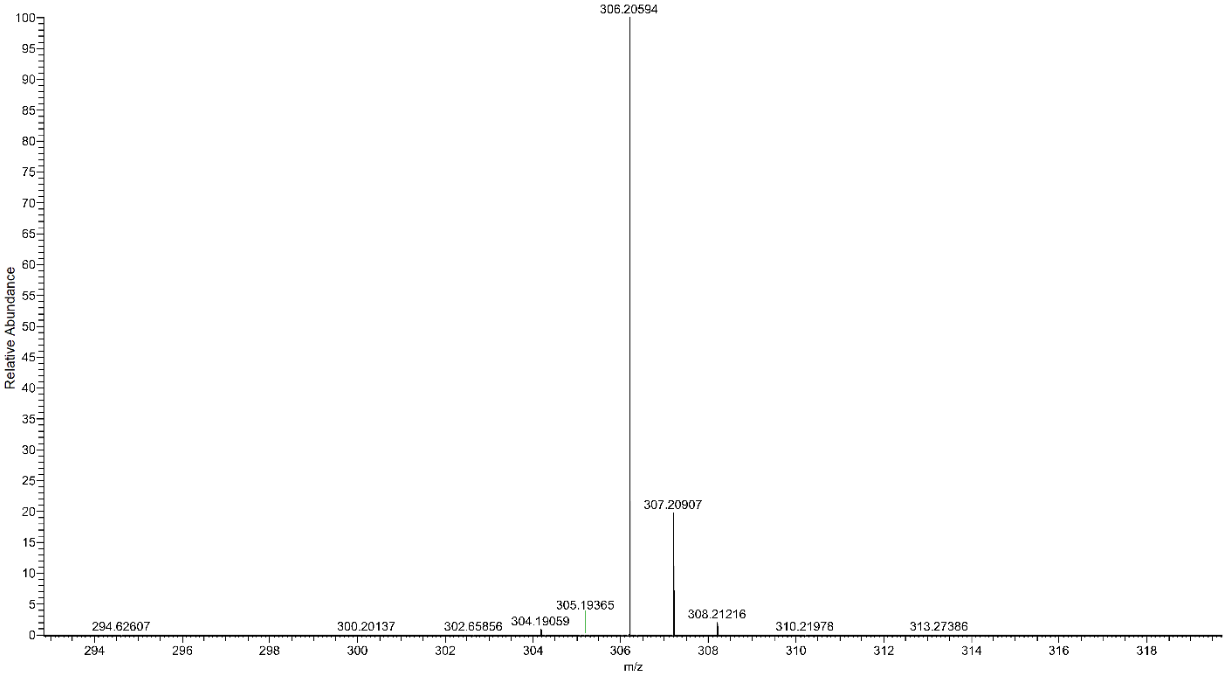


**Figure S18**. HRMS spectrum of compound **25**.

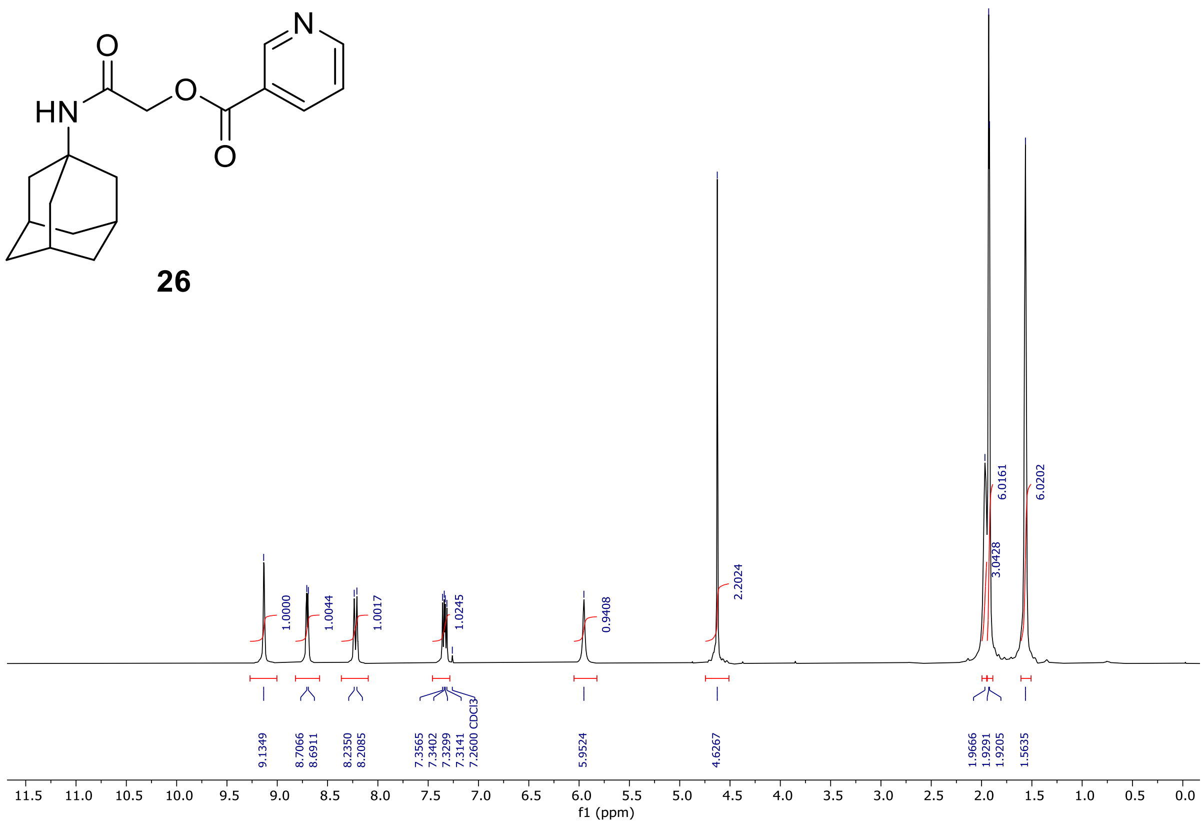


**Figure S19**. $^{1}$H NMR spectrum of compound **26**.

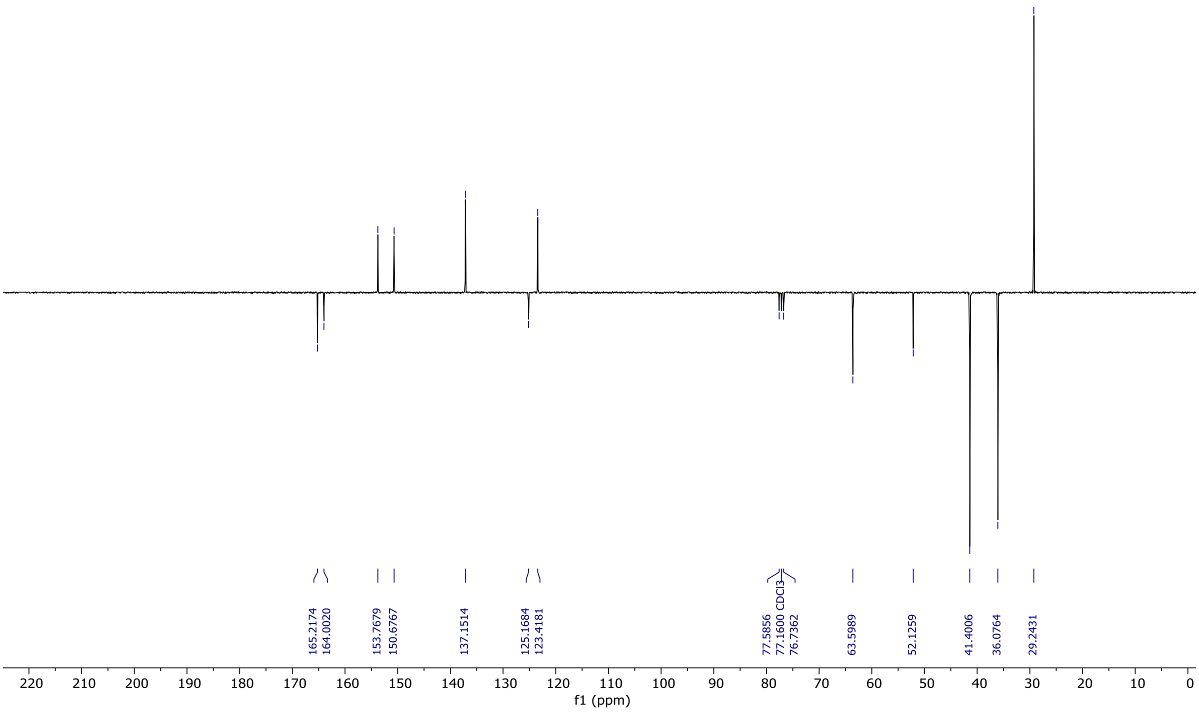


**Figure S20**. $^{13}$C APT NMR spectrum of compound **26**.

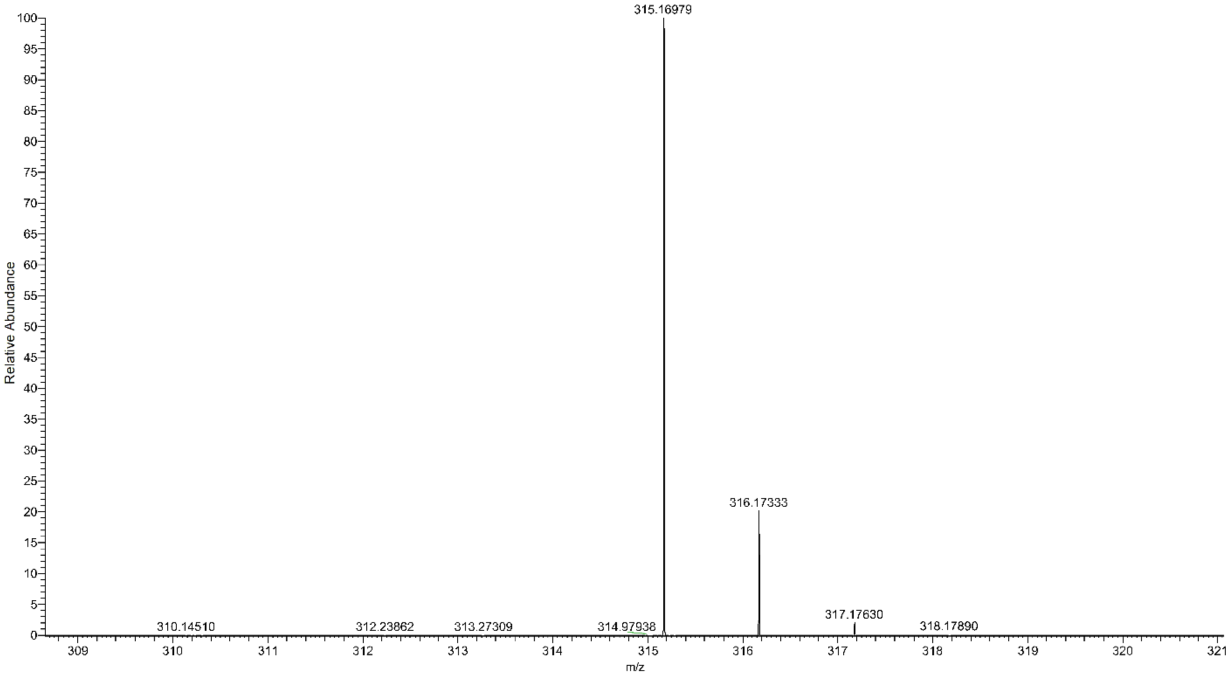

**Figure S21**. HRMS spectrum of compound **26**.

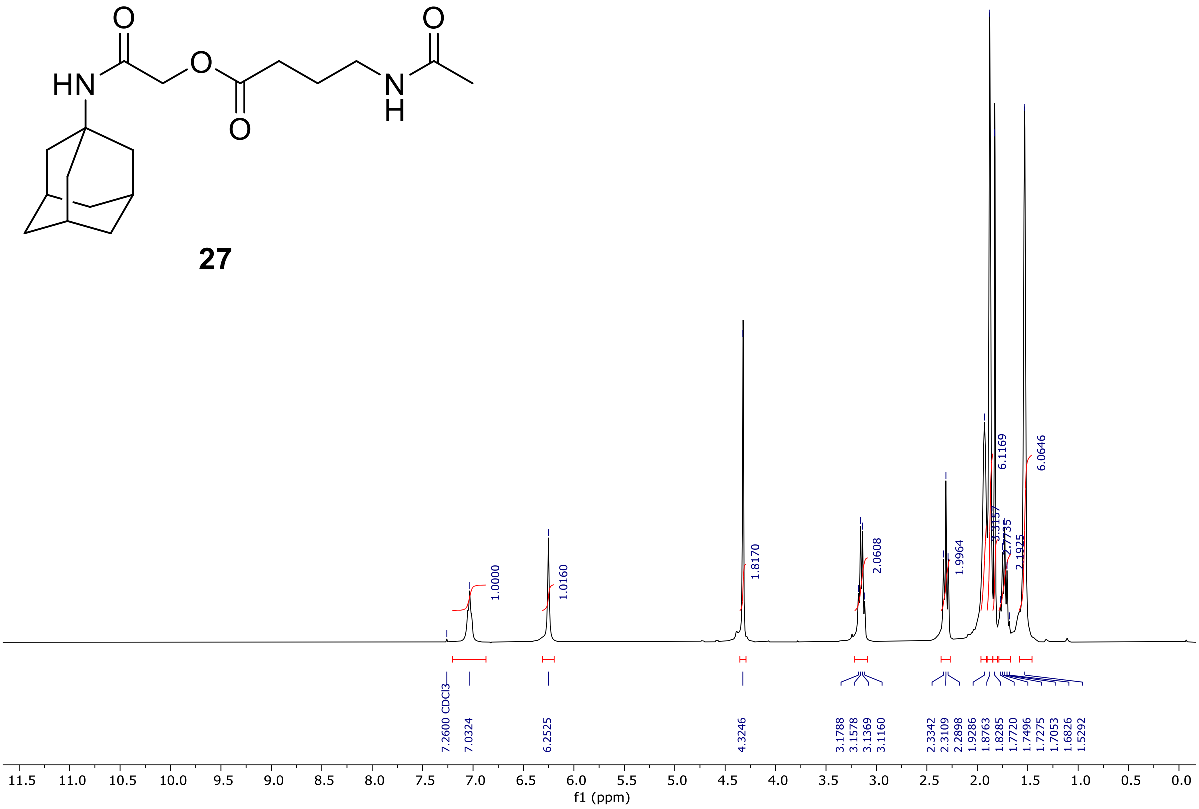


**Figure S22**. $^{1}$H NMR spectrum of compound **27**.

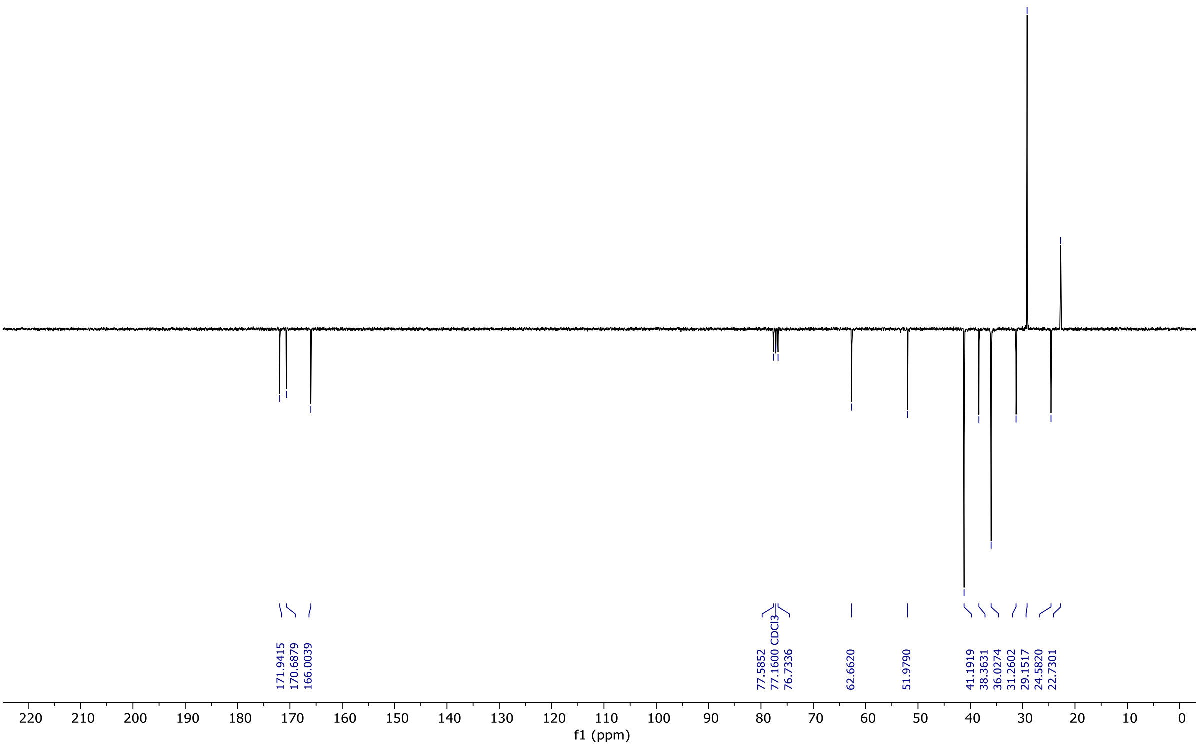


**Figure S23**. $^{13}$C APT NMR spectrum of compound **27**.

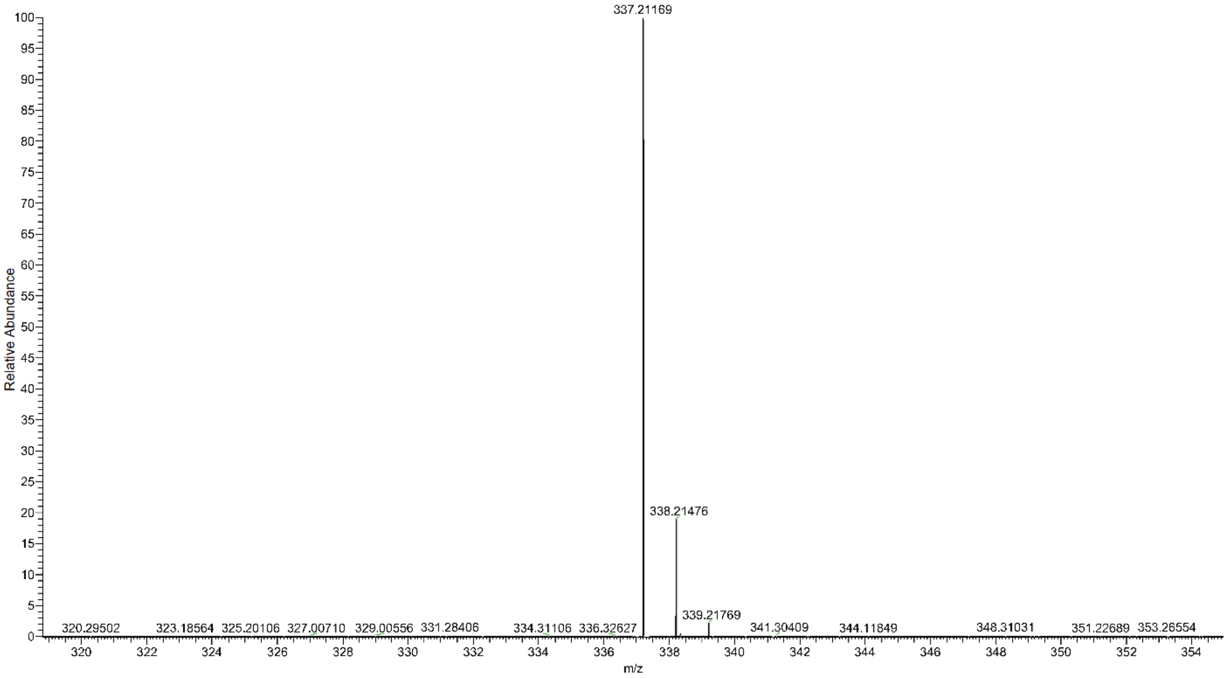


**Figure S24**. HRMS spectrum of compound **27**.

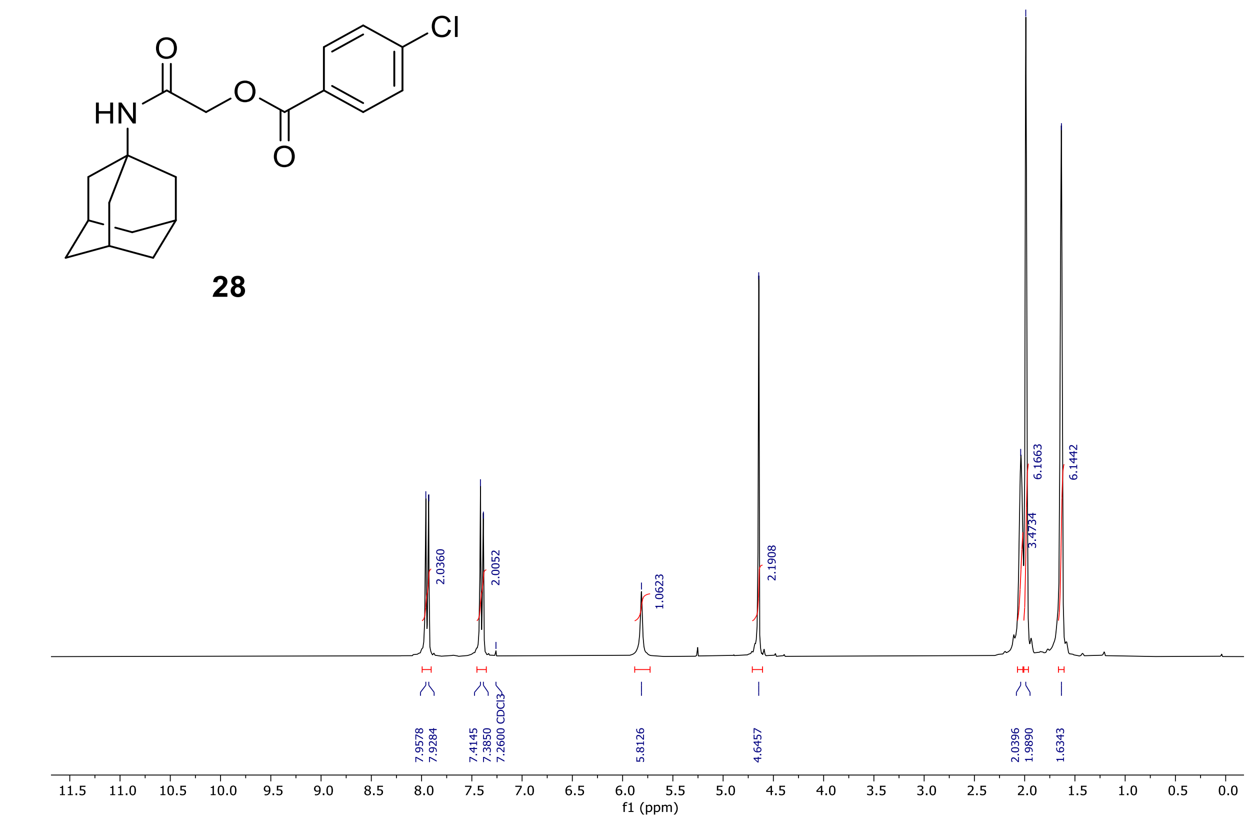


**Figure S25**. $^{1}$H NMR spectrum of compound **28**.

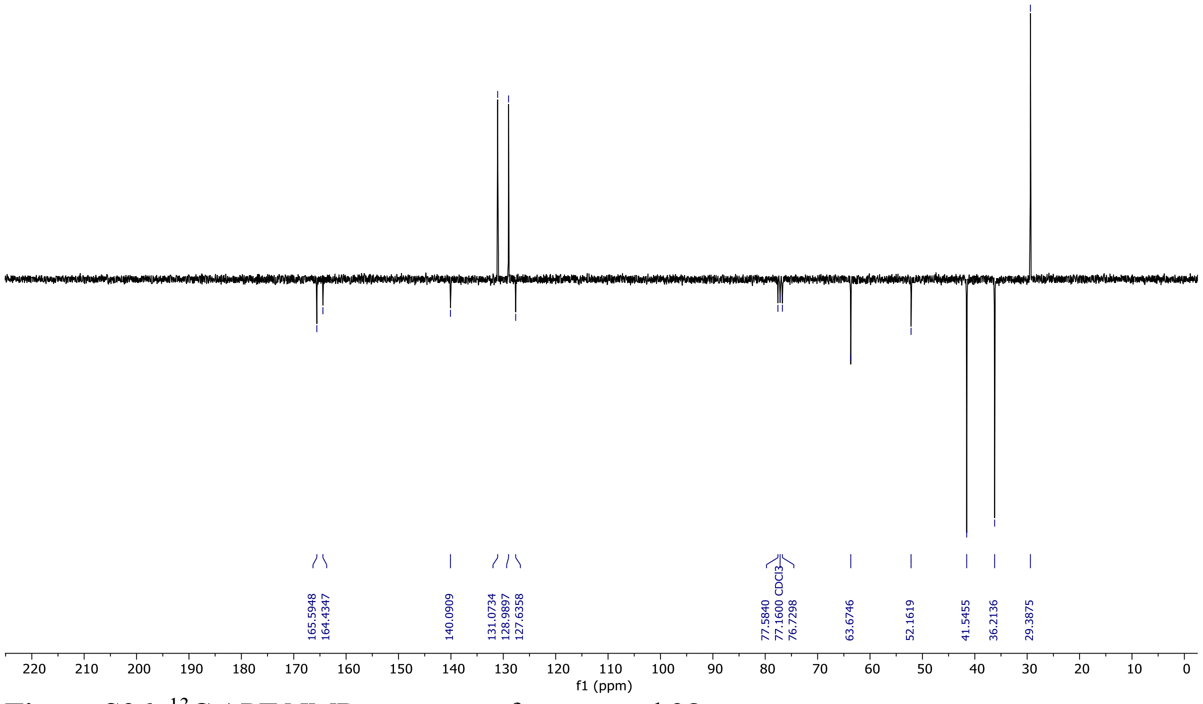


**Figure S26**. $^{13}$C APT NMR spectrum of compound **28**.

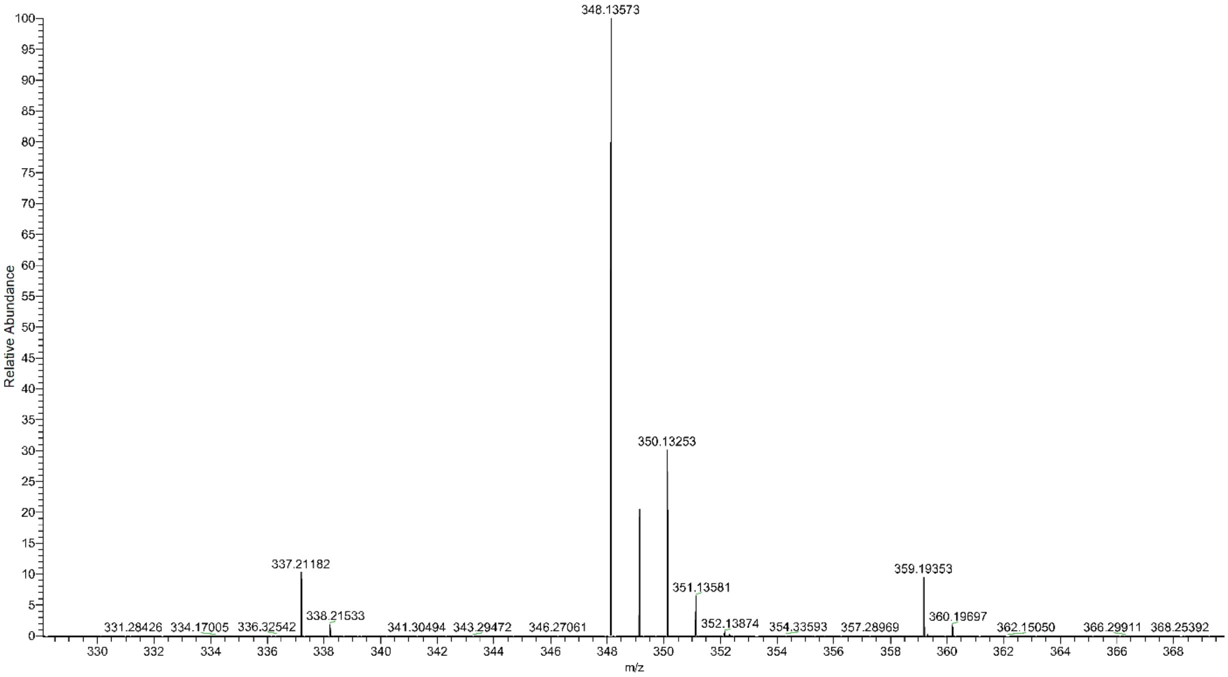


**Figure S27**. HRMS spectrum of compound **28**.

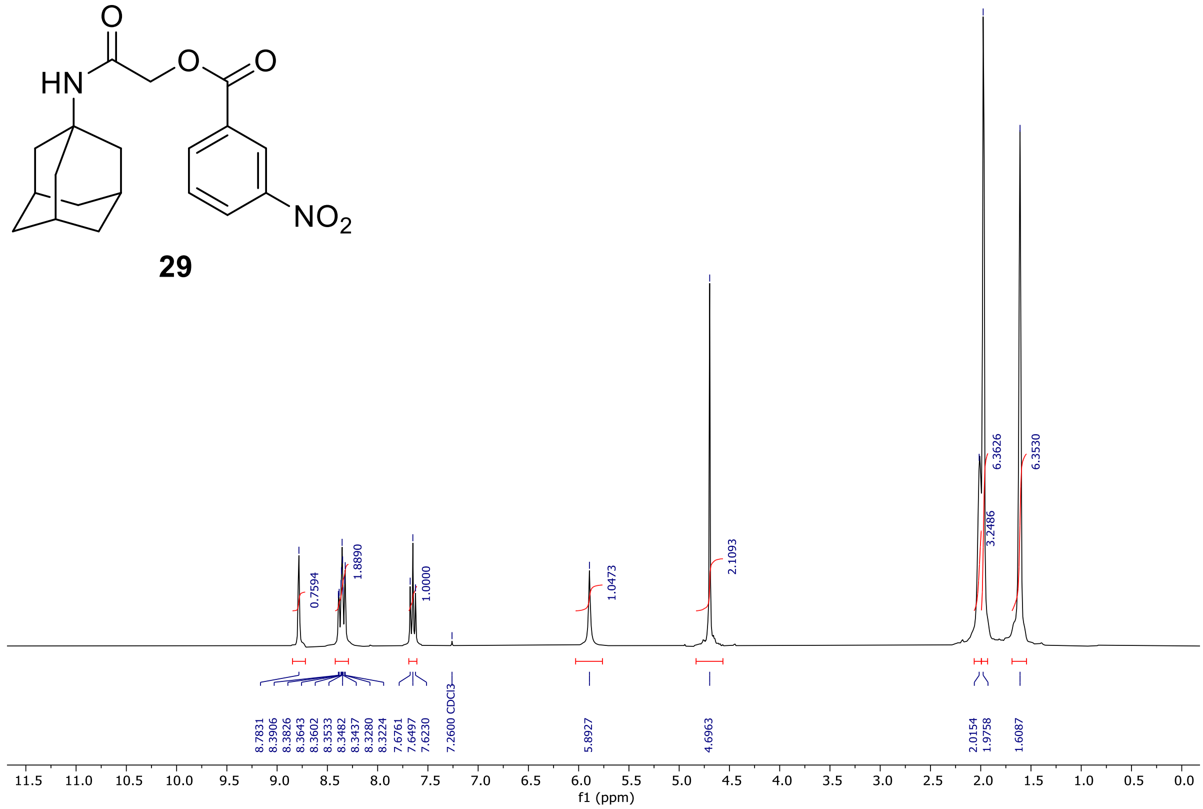


**Figure S28**. $^{1}$H NMR spectrum of compound **29**.

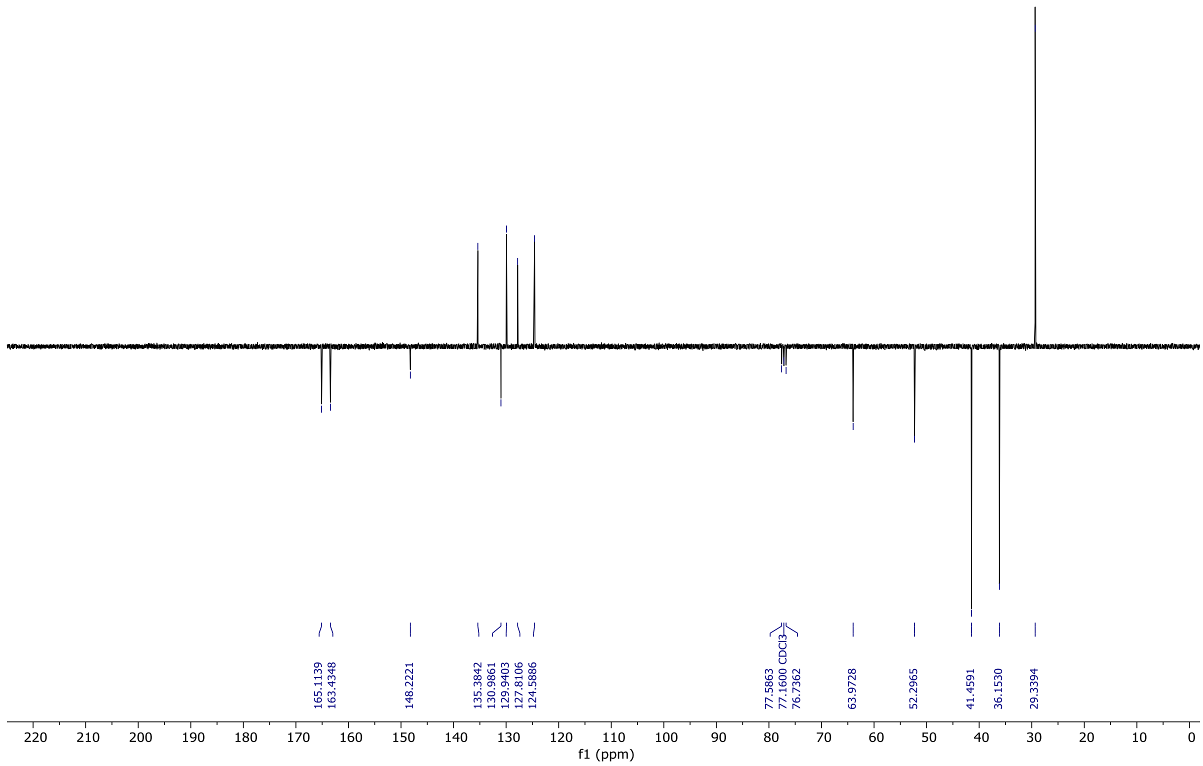


**Figure S29**. $^{13}$C APT NMR spectrum of compound **29**.

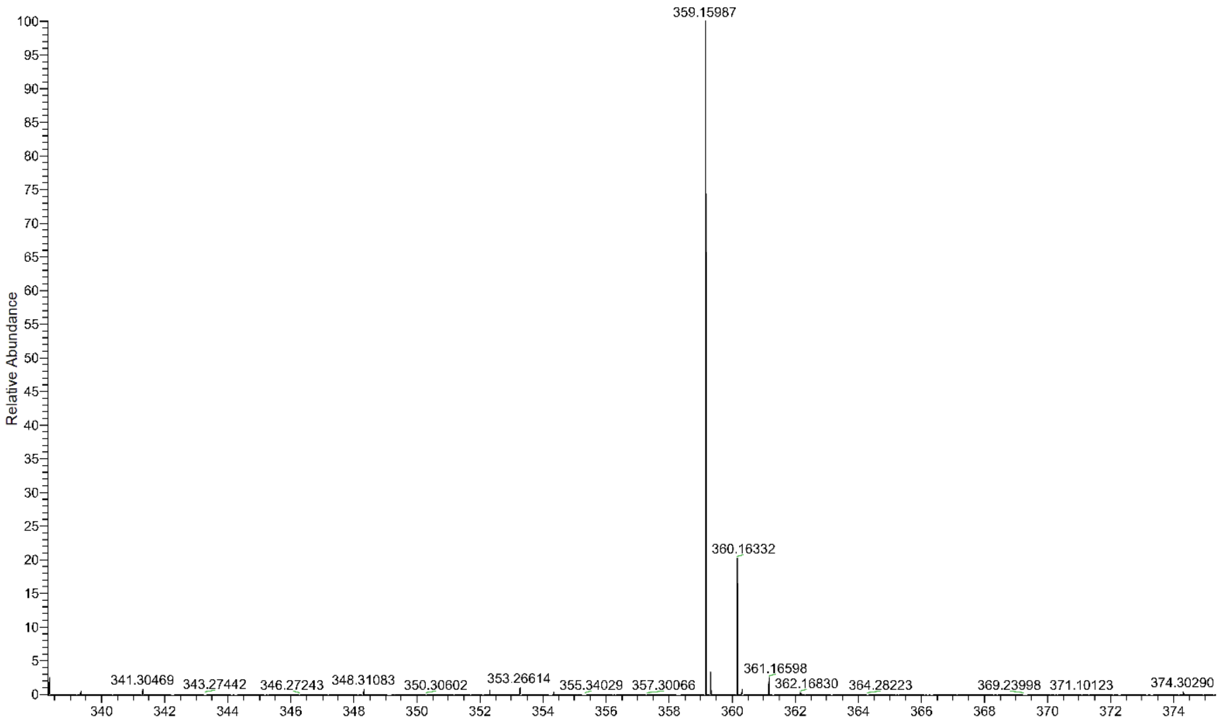


**Figure S30**. HRMS spectrum of compound **29**.

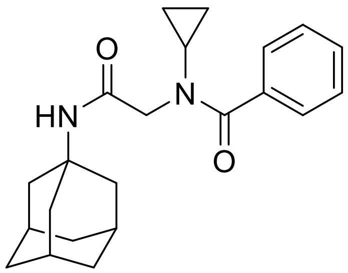


**30**

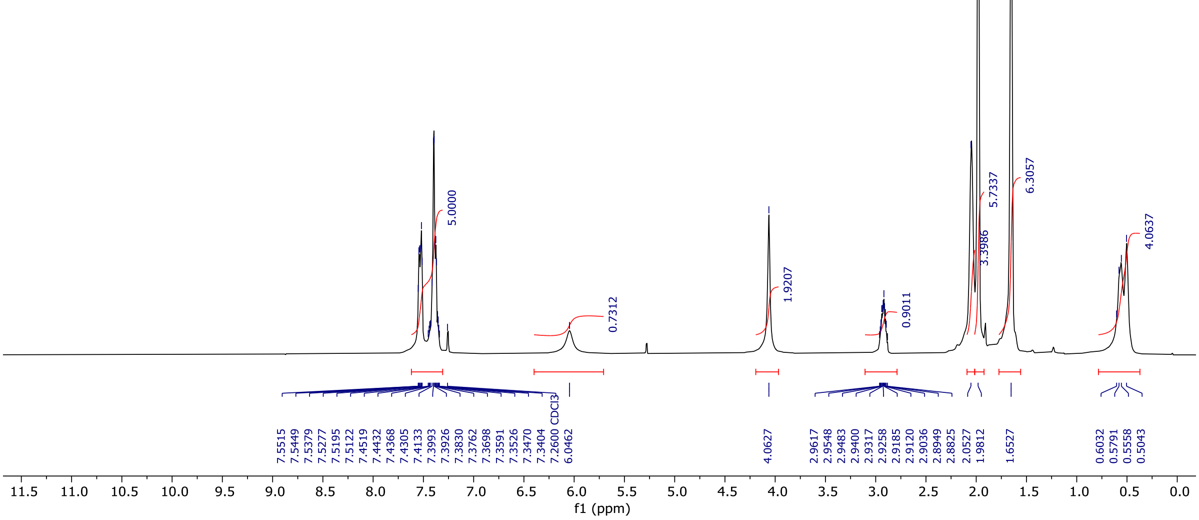


**Figure S31**. $^{1}$H NMR spectrum of compound **30**.

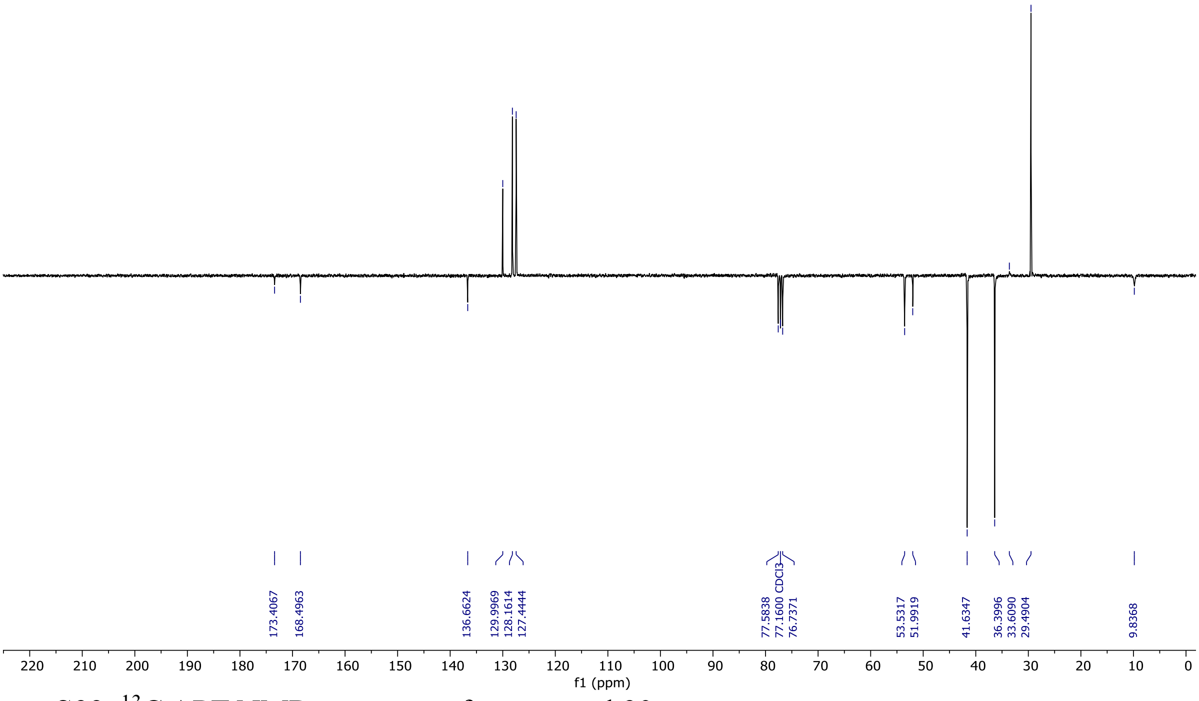


**Figure S32**. $^{13}$C APT NMR spectrum of compound **30**.

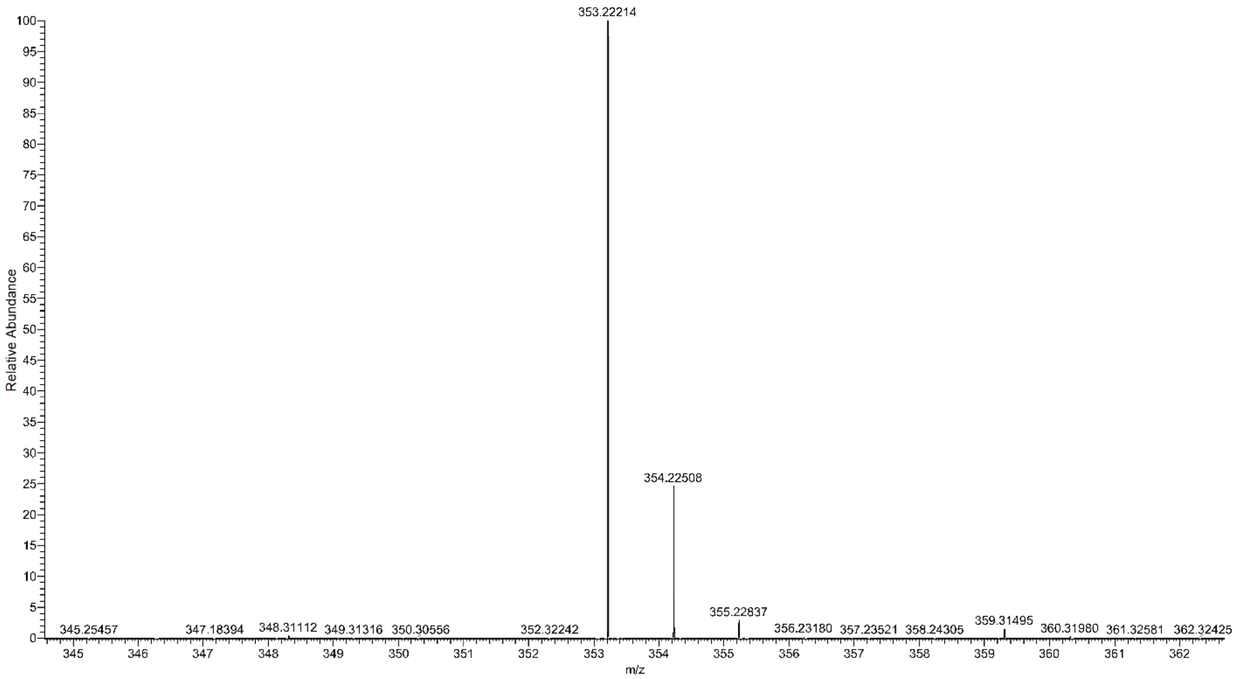


**Figure S33**. HRMS spectrum of compound **30**.

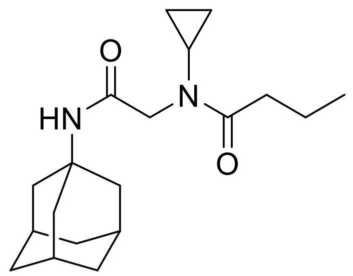


**31**

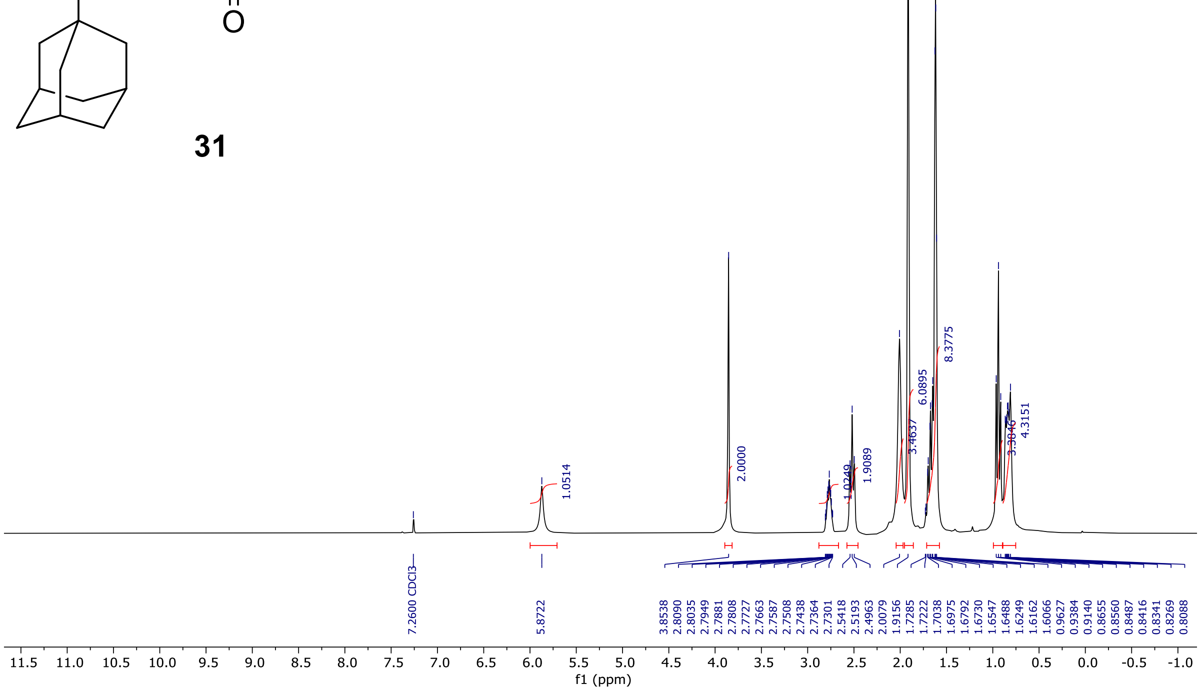


**Figure S34**. ¹H NMR spectrum of compound **31**.

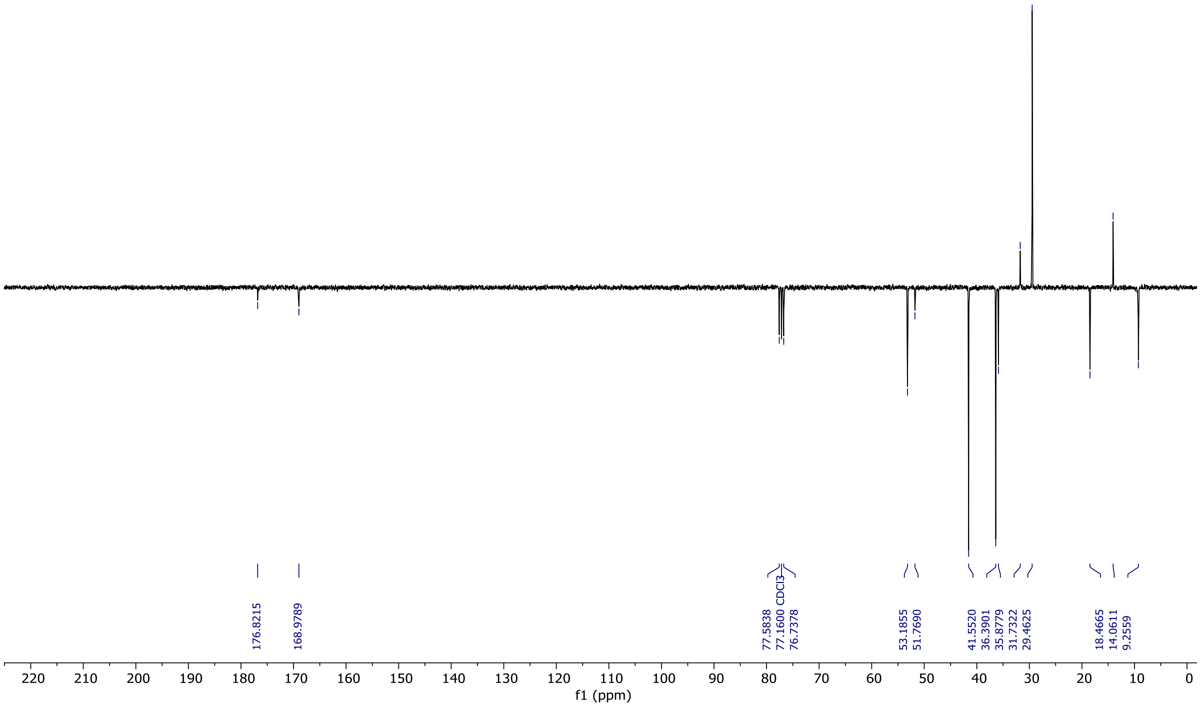


**Figure S35**. ¹³C APT NMR spectrum of compound **31**.

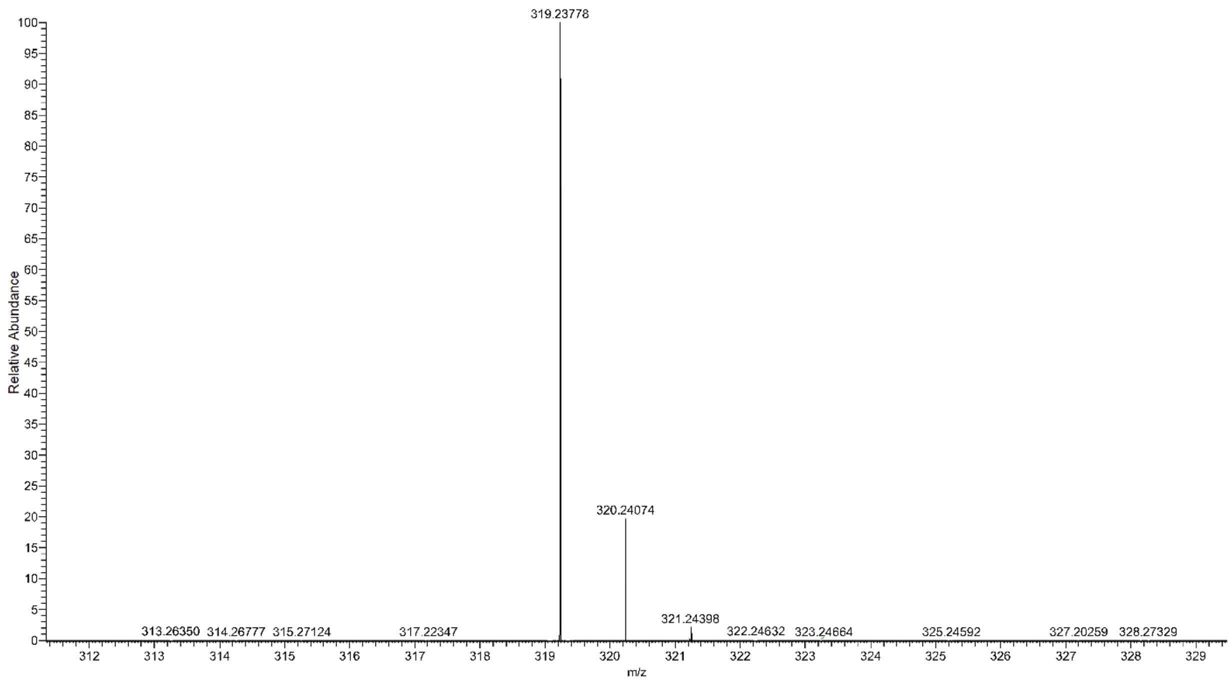


**Figure S36**. HRMS spectrum of compound **31**.

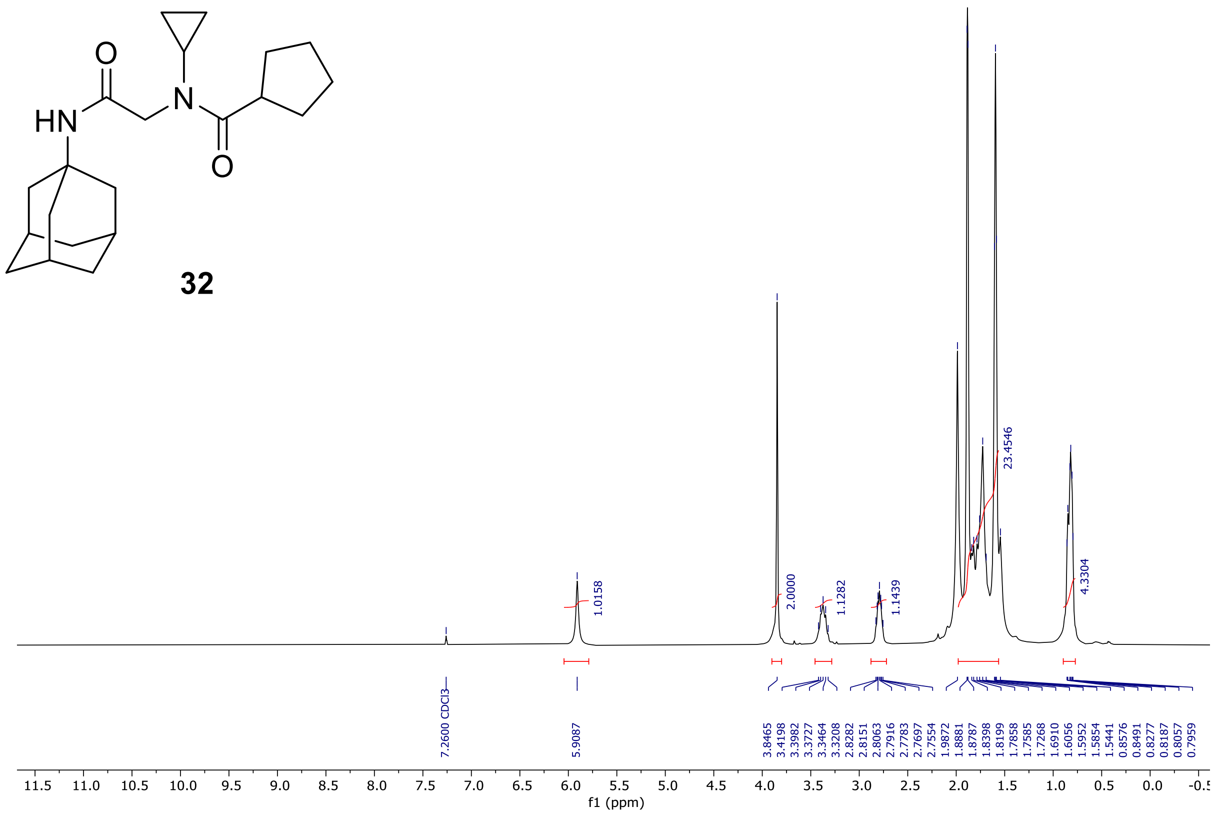


**32**



**Figure S37**. $^{1}$H NMR spectrum of compound **32**.

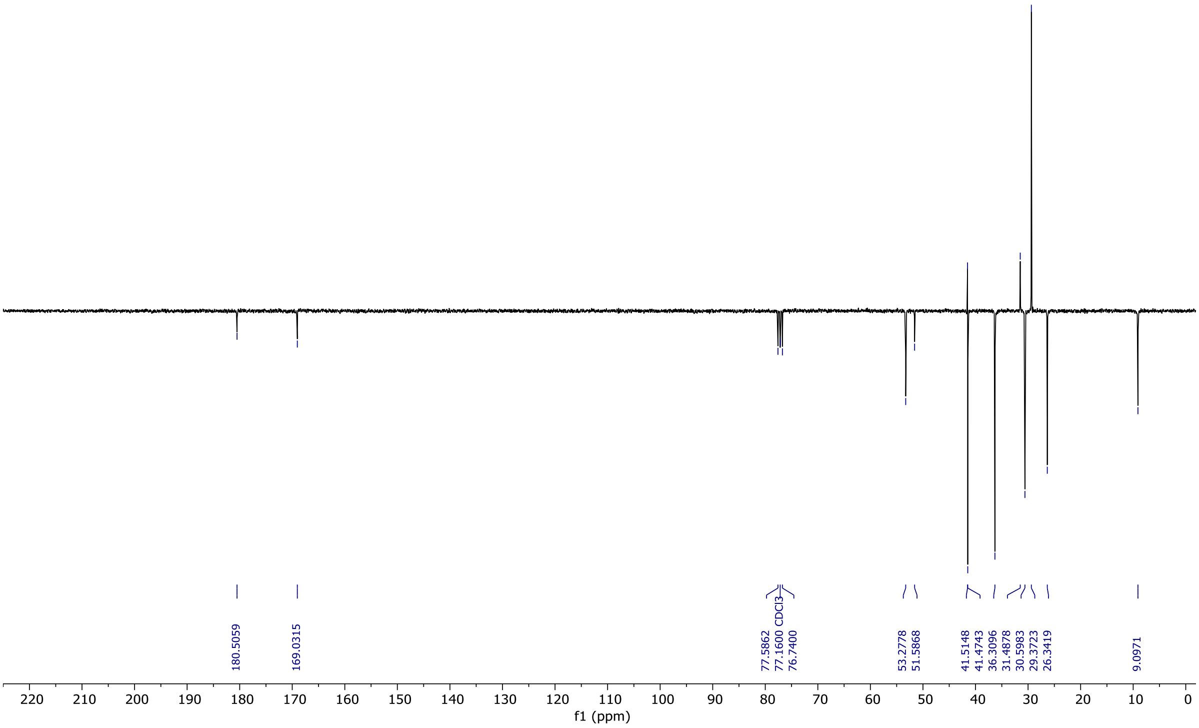


**Figure S38**. $^{13}$C APT NMR spectrum of compound **32**.

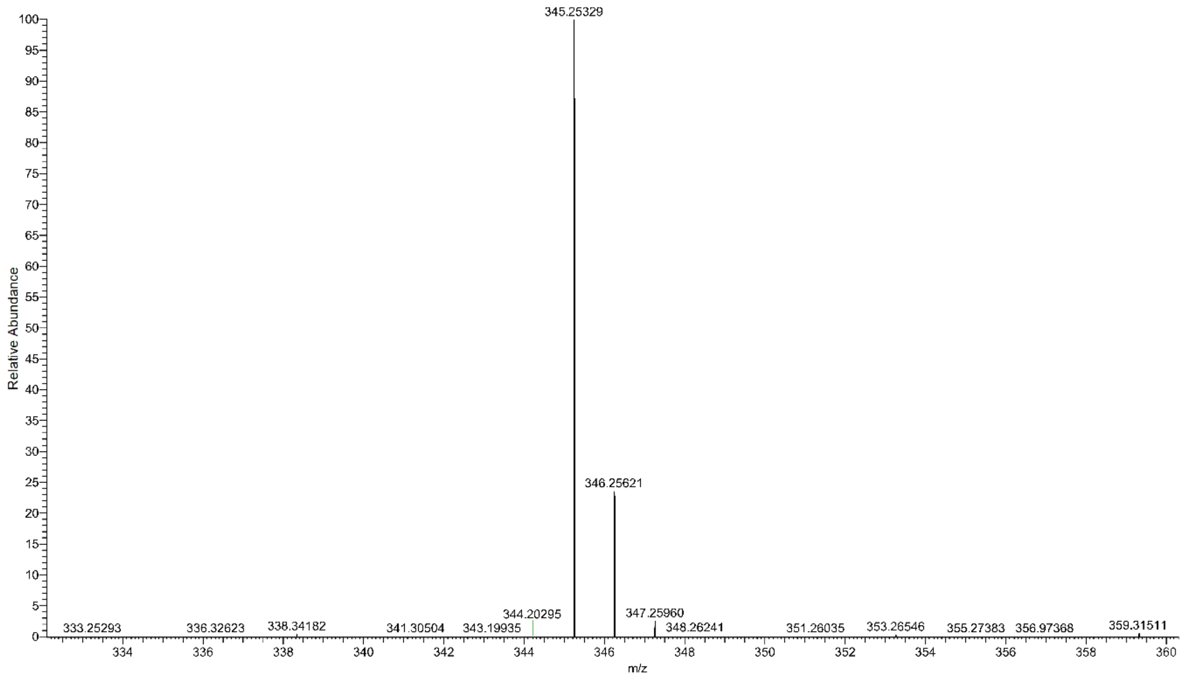


**Figure S39**. HRMS spectrum of compound **32**.

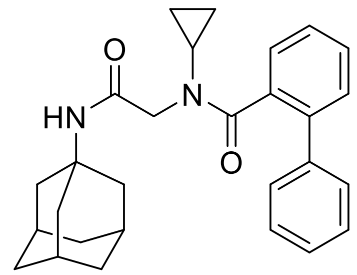


**33**

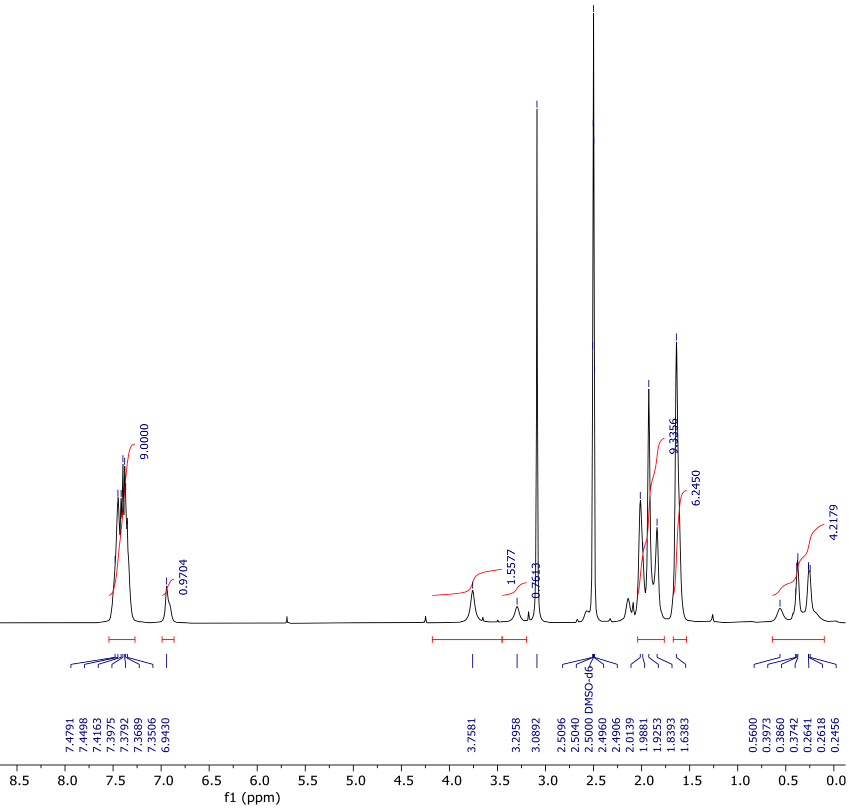


**Figure S40**. $^{1}$H NMR spectrum of compound **33**.

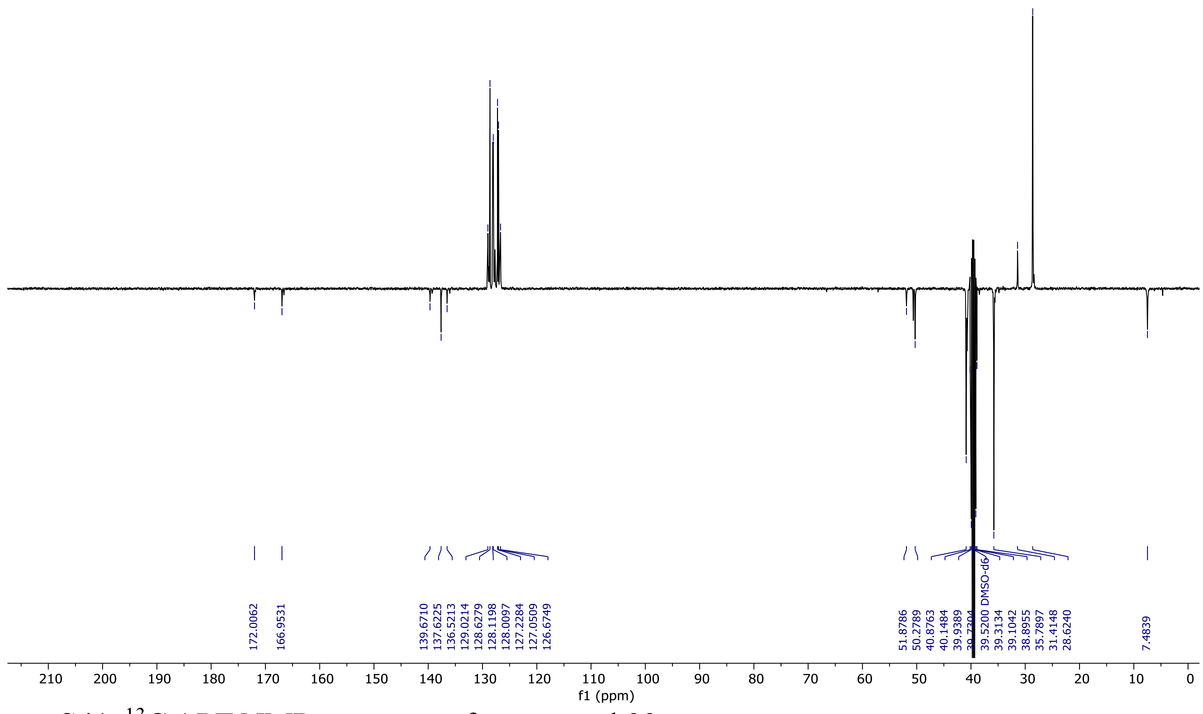


**Figure S41**. $^{13}$C APT NMR spectrum of compound **33**.

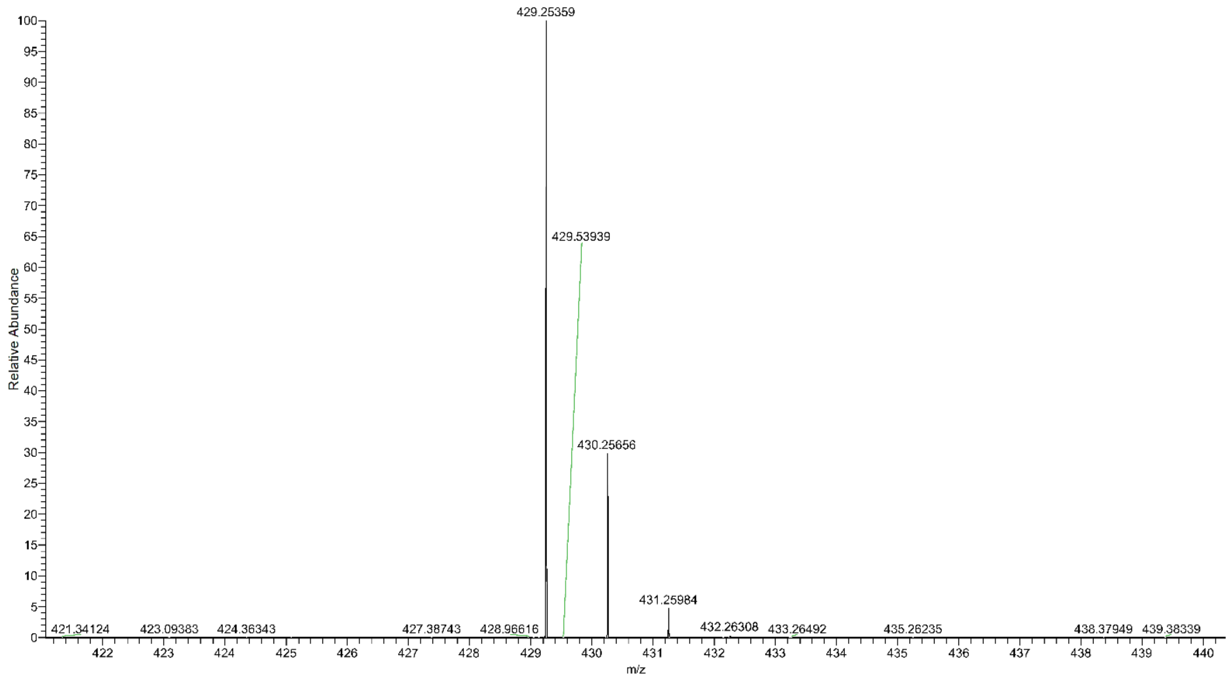

**Figure S42**. HRMS spectrum of compound **33**.

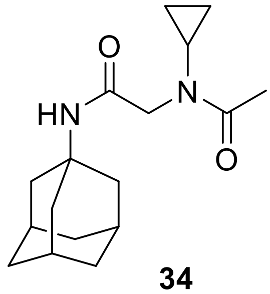

**34**

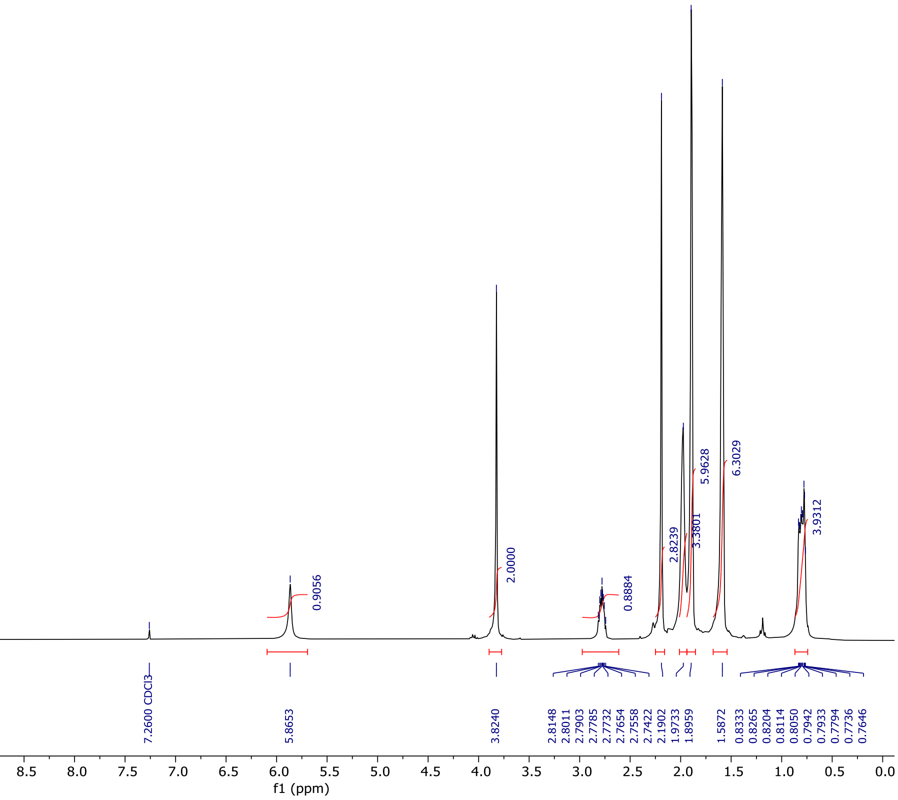


**Figure S43**. $^{1}$H NMR spectrum of compound **34**.

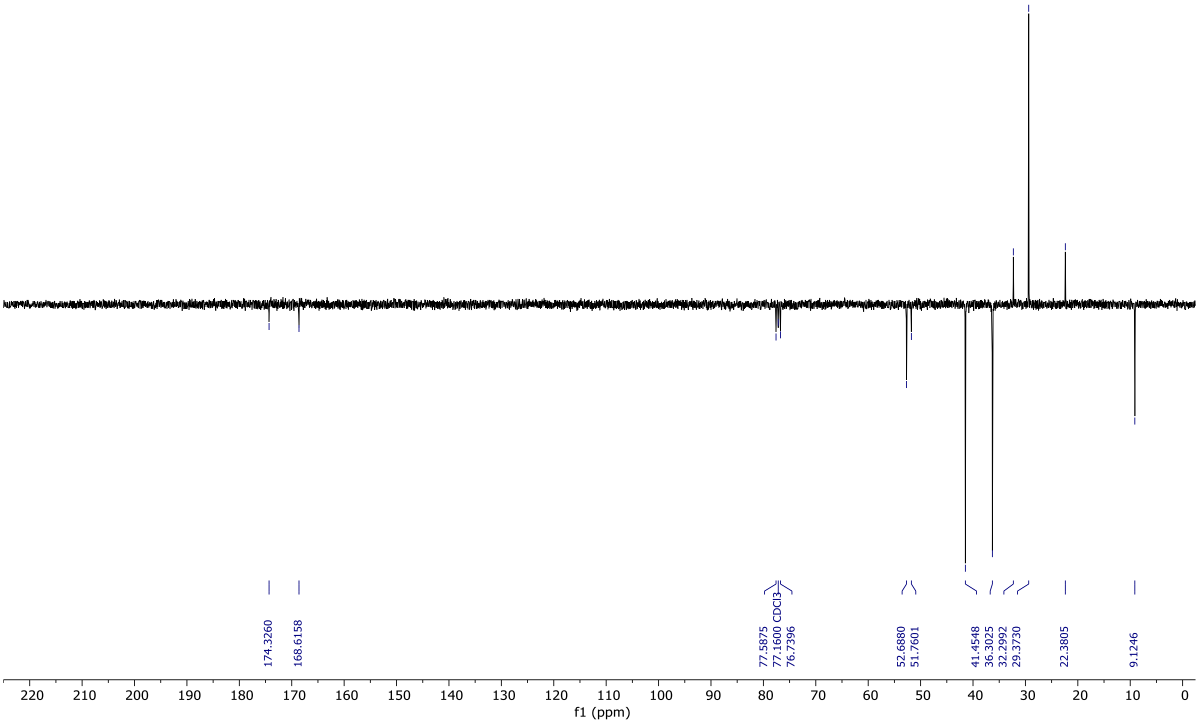


**Figure S44**. $^{13}$C APT NMR spectrum of compound **34**.

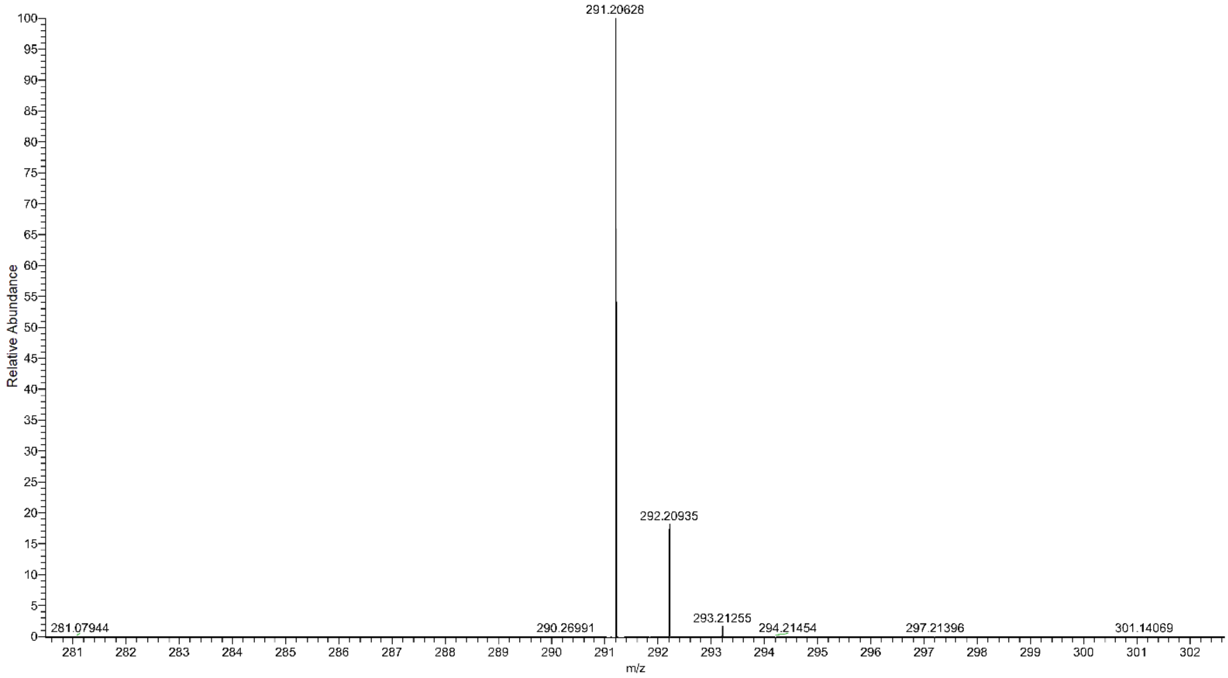


**Figure S45**. HRMS spectrum of compound **34**.

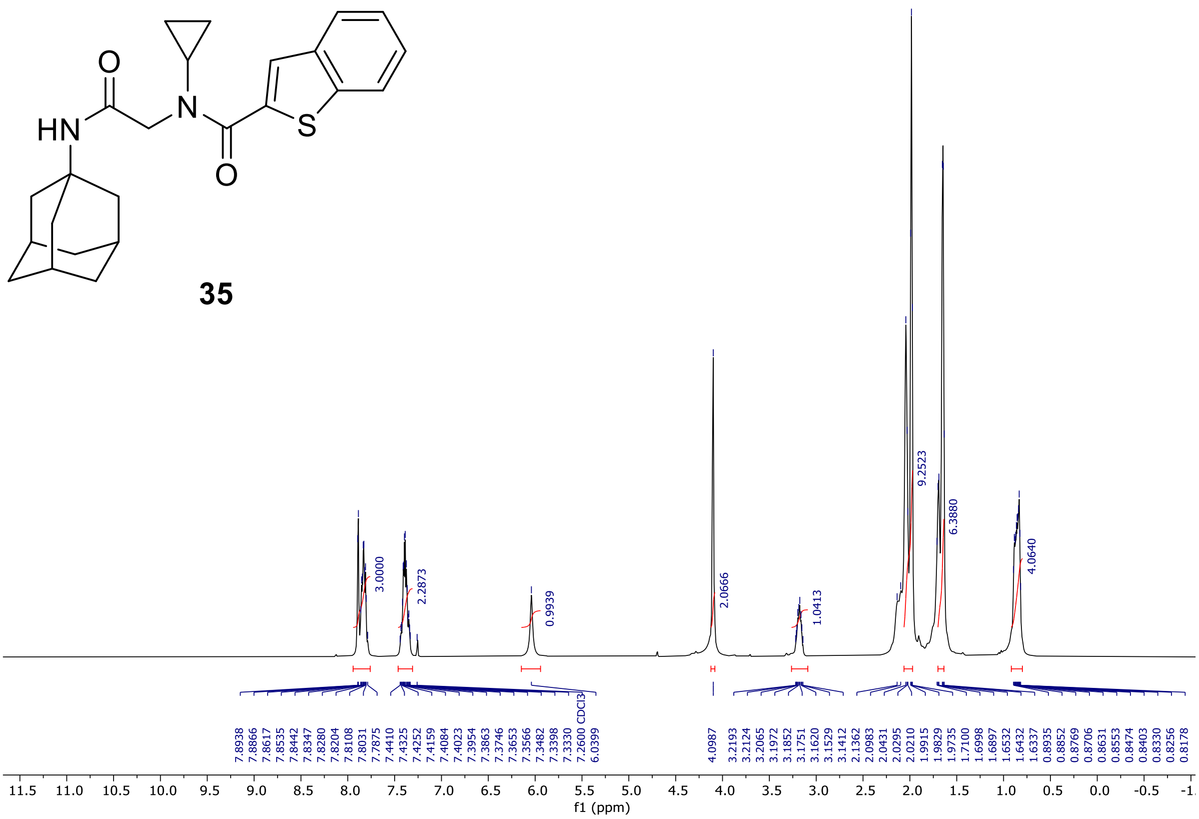


**Figure S46**. $^{1}$H NMR spectrum of compound **35**.

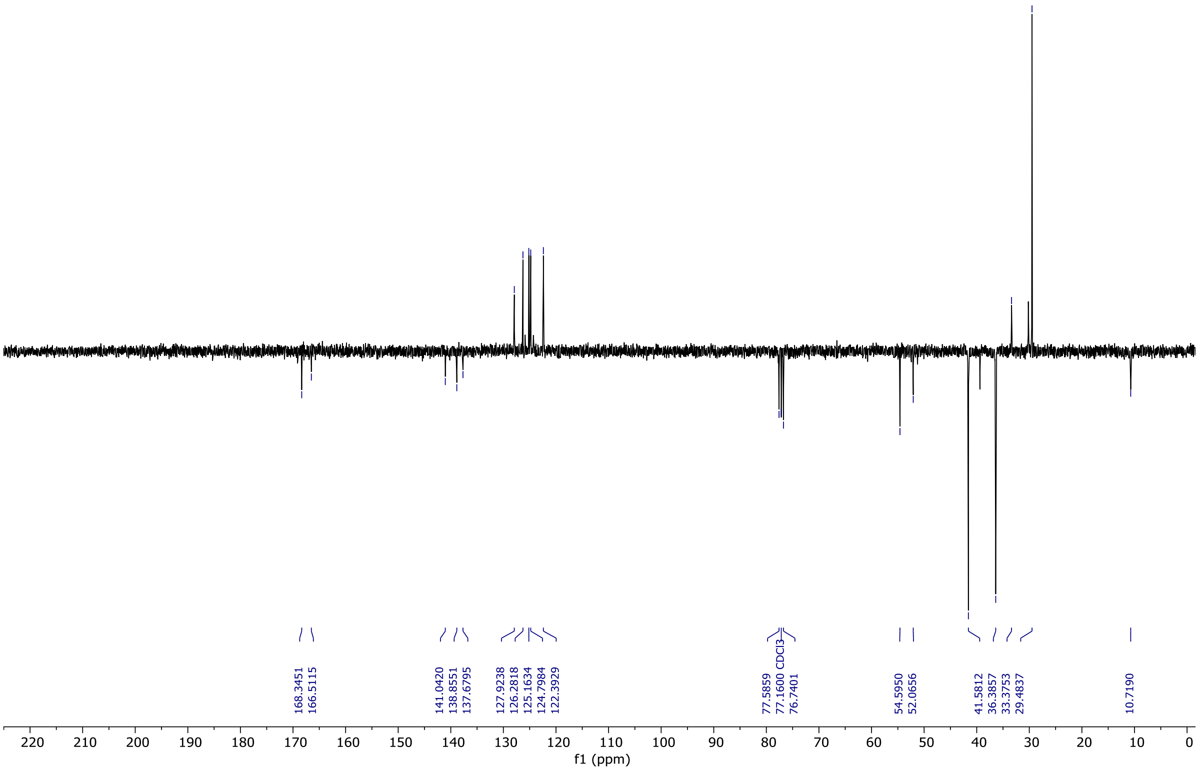


**Figure S47**. $^{13}$C APT NMR spectrum of compound **35**.

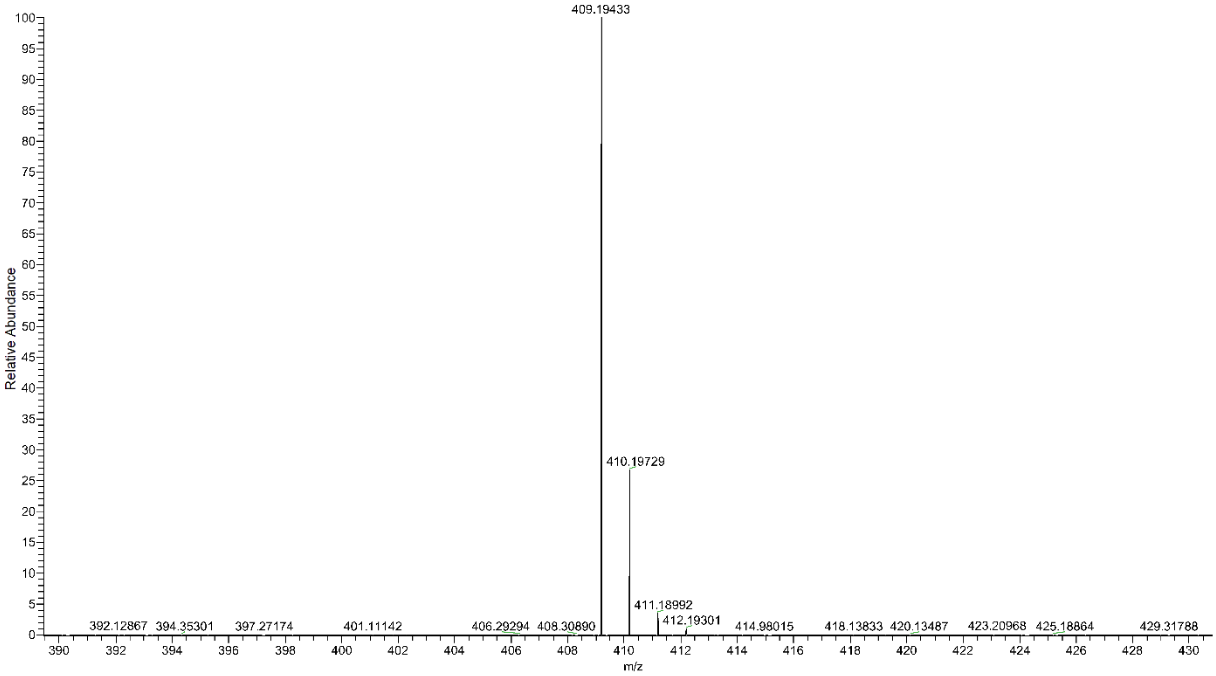


**Figure S48**. HRMS spectrum of compound **35**.

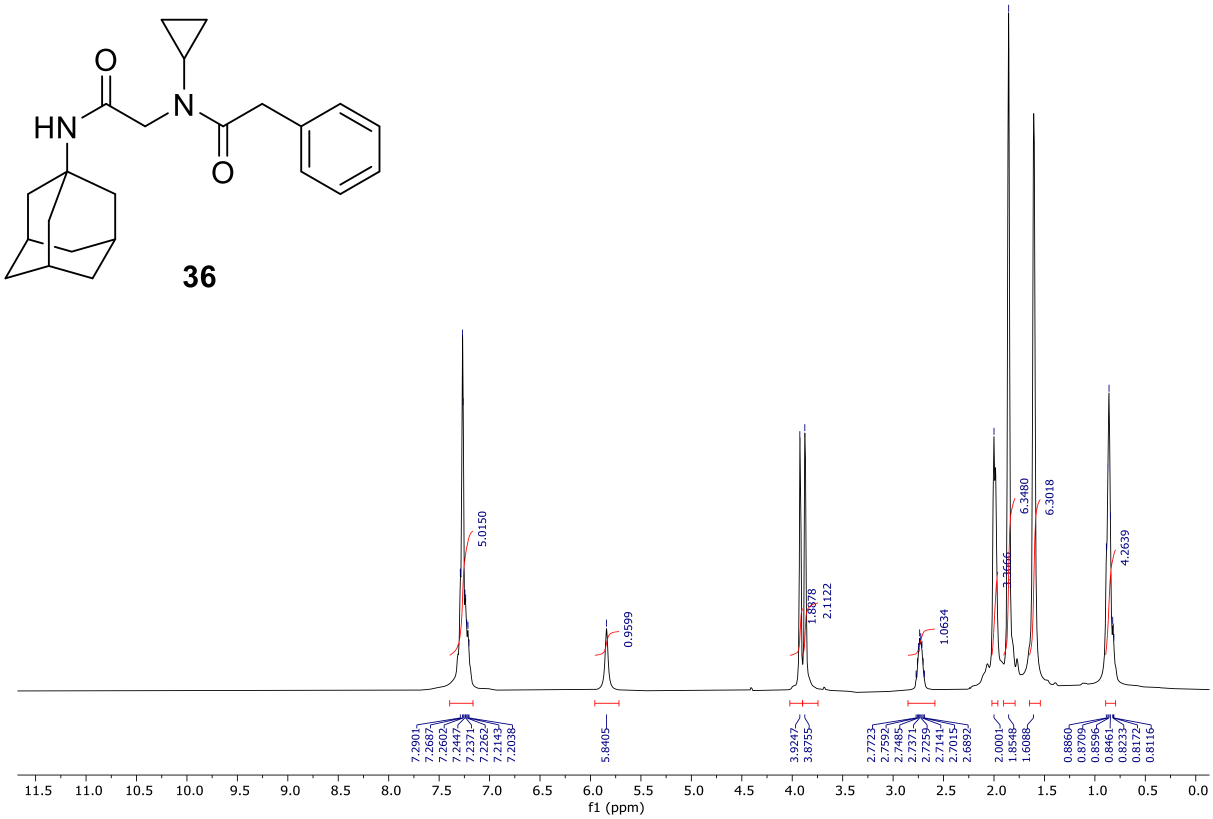


**Figure S49**. $^{1}$H NMR spectrum of compound **36**.

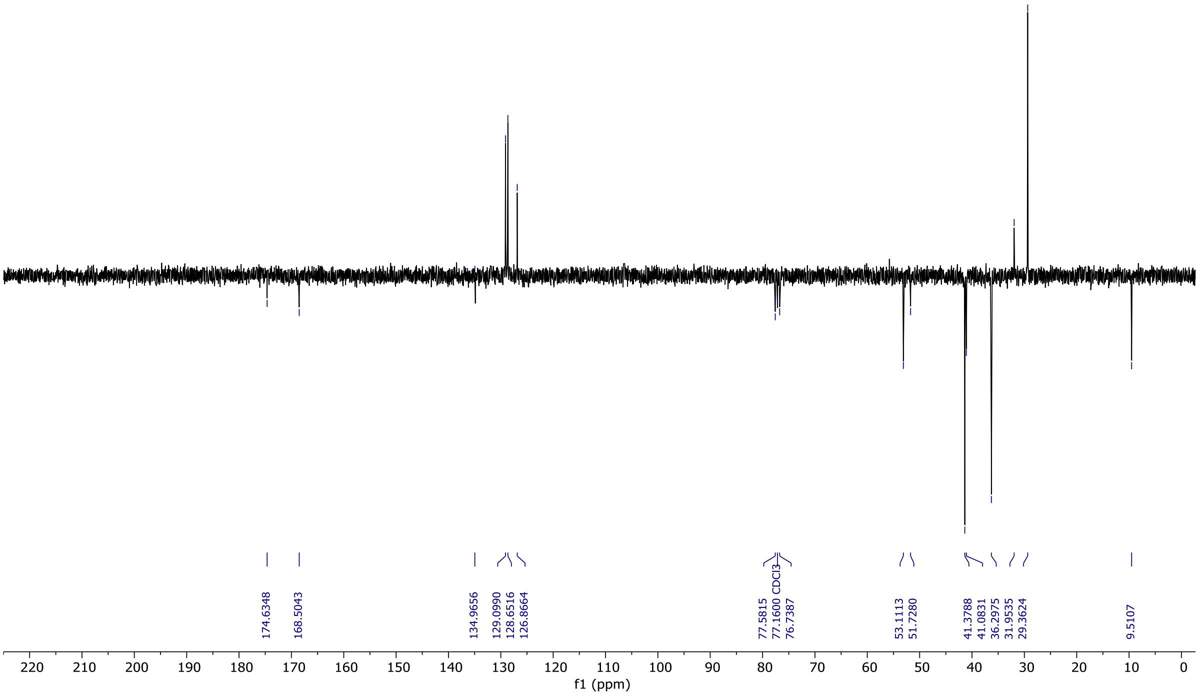


**Figure S50**. $^{13}$C APT NMR spectrum of compound **36**.

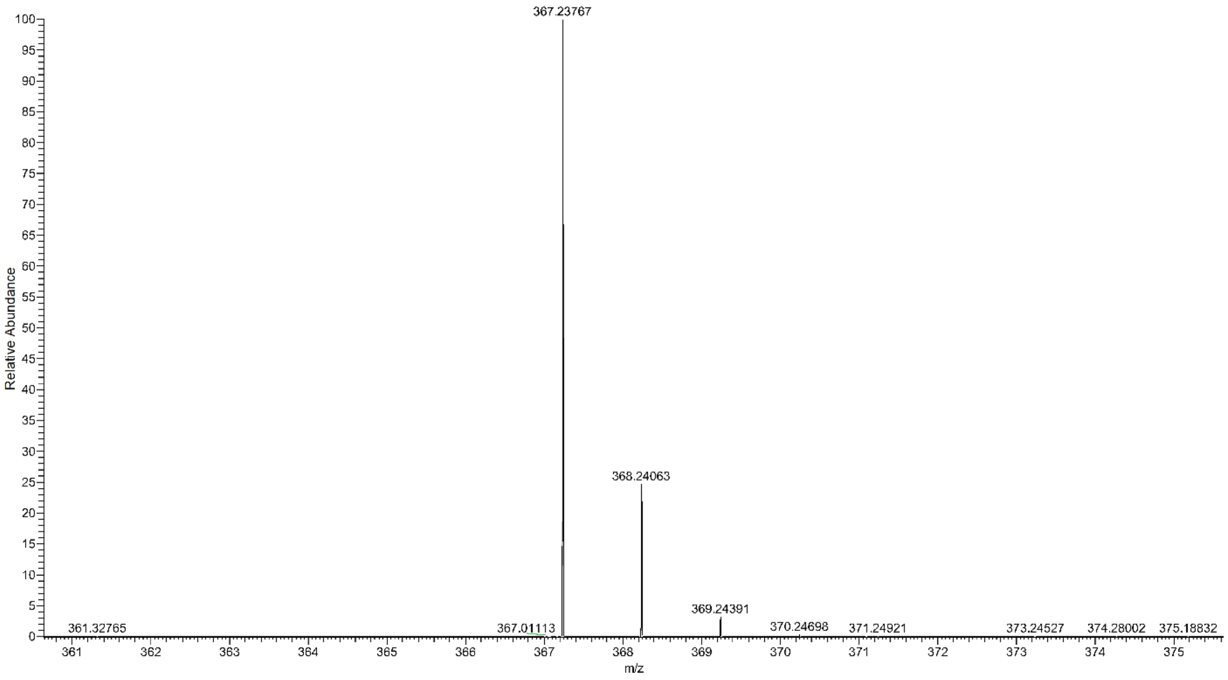

**Figure S51**. HRMS spectrum of compound **36**.

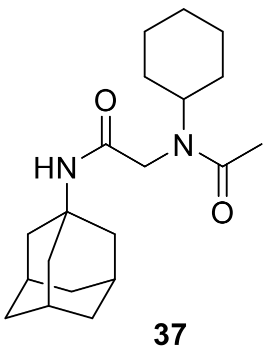

**37**

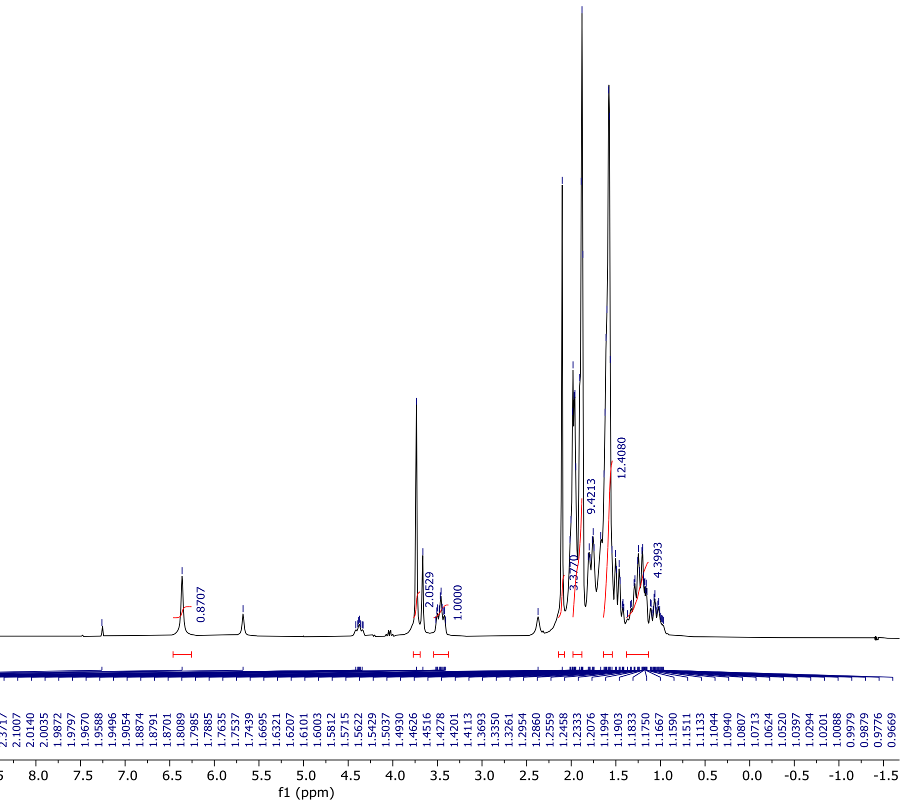


**Figure S52**. $^{1}$H NMR spectrum of compound **37**.

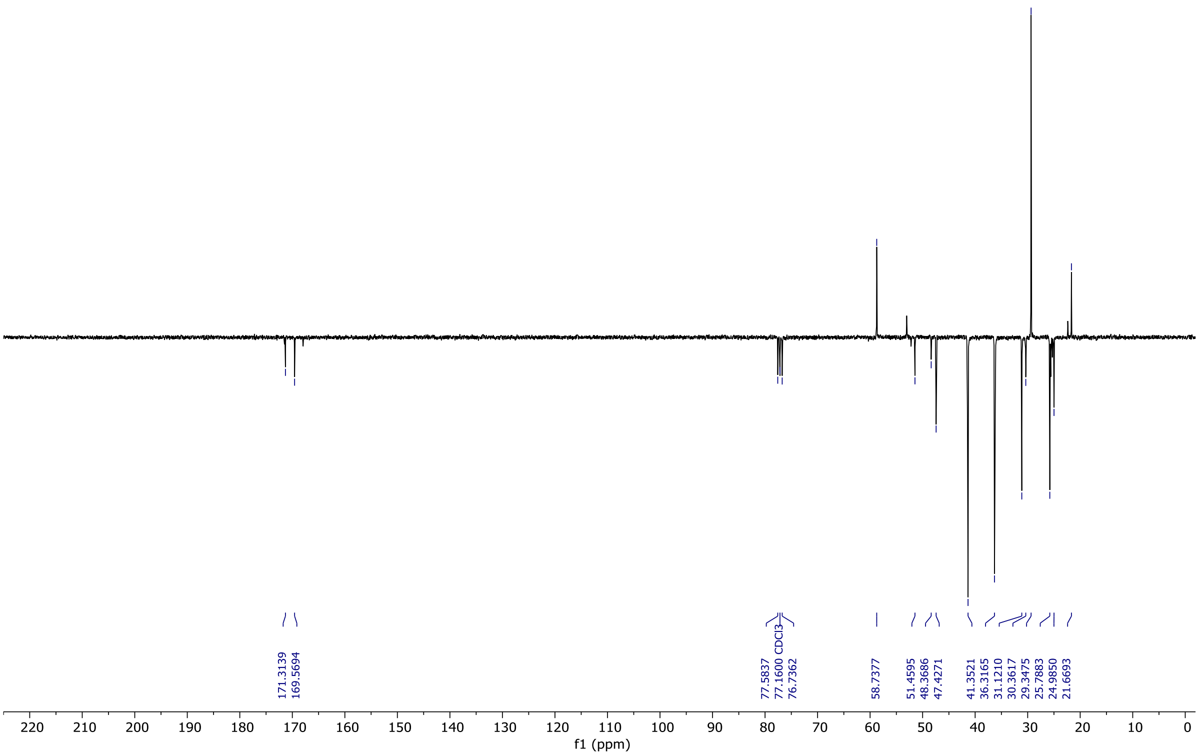


**Figure S53**. $^{13}$C APT NMR spectrum of compound **37**.

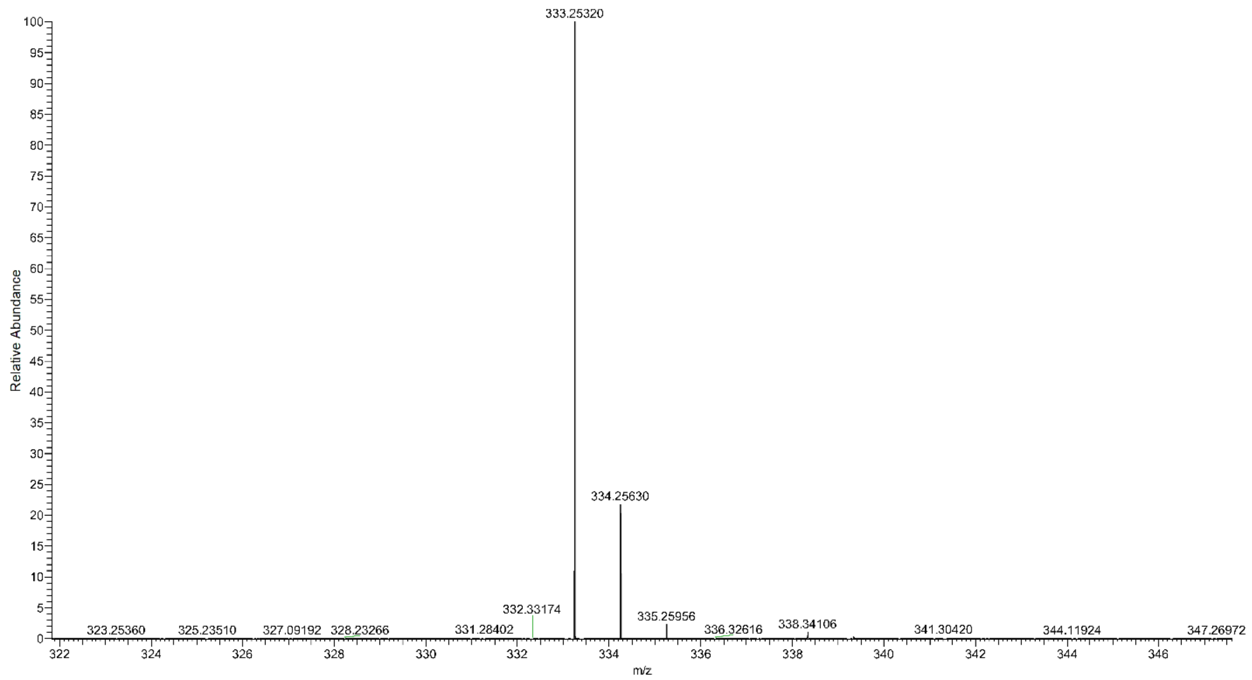


**Figure S54**. HRMS spectrum of compound **37**.

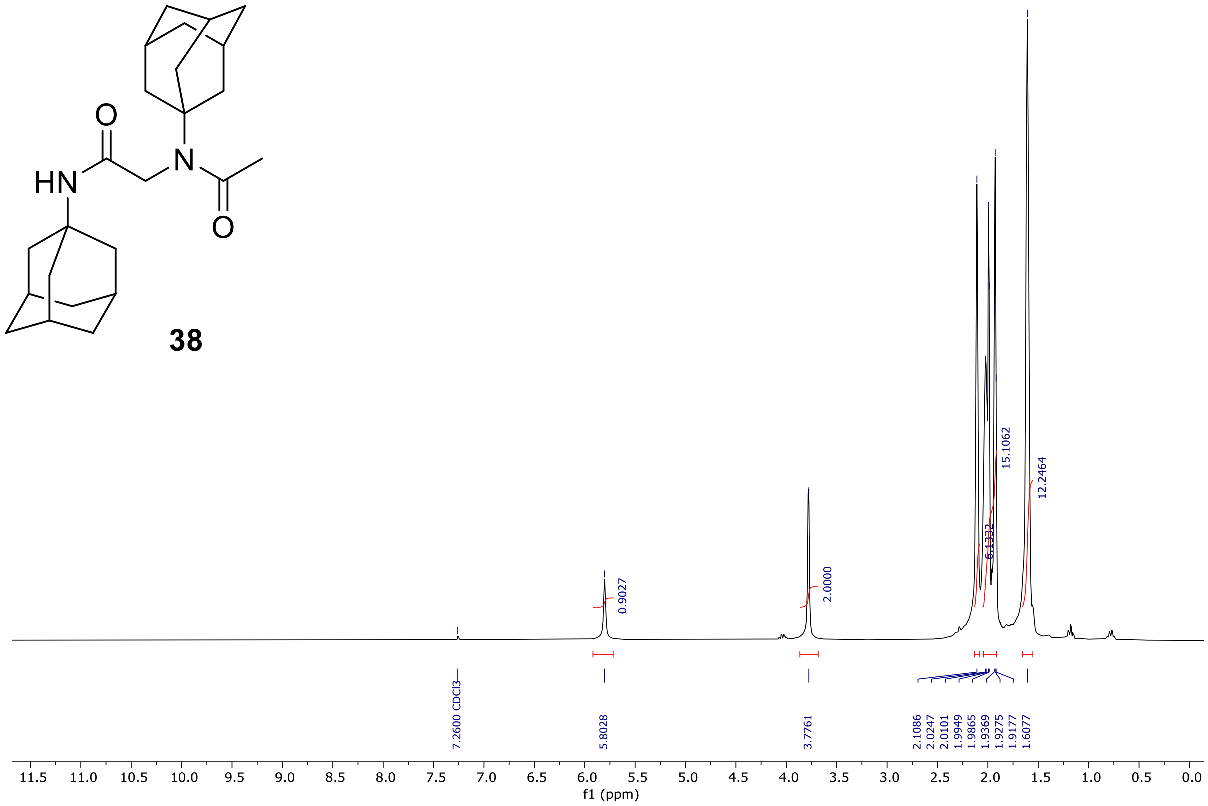


**Figure S55**. $^{1}$H NMR spectrum of compound **38**.

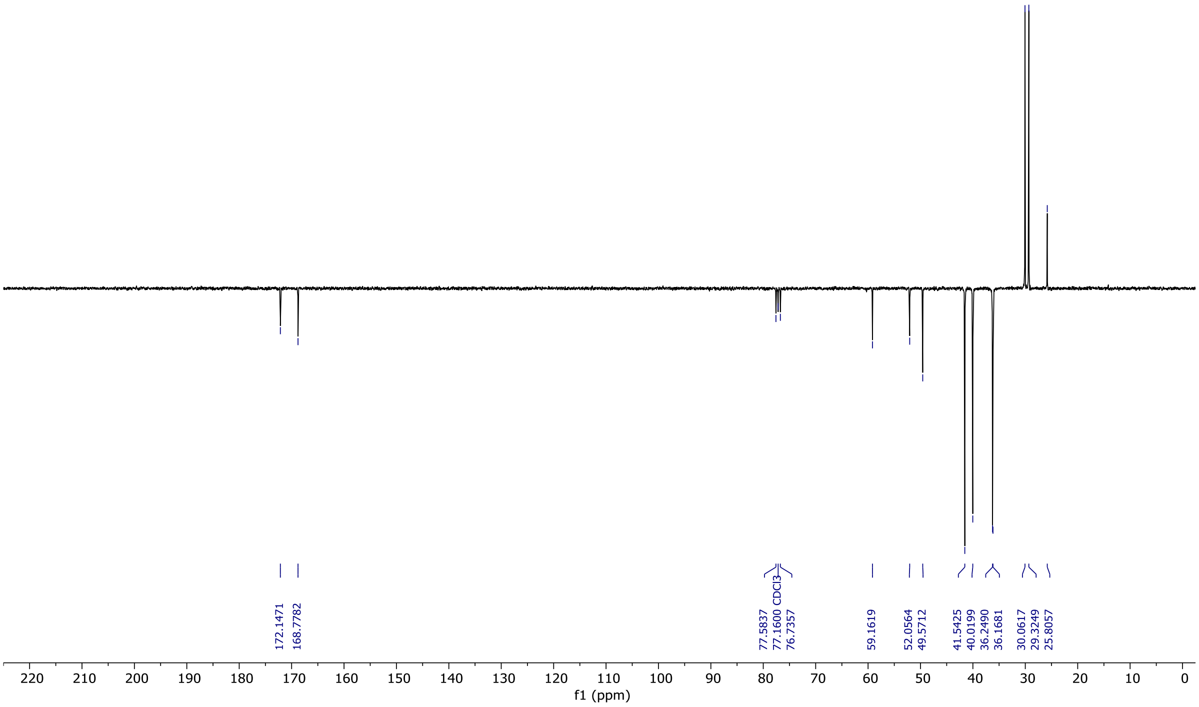


**Figure S56**. $^{13}$C APT NMR spectrum of compound **38**.

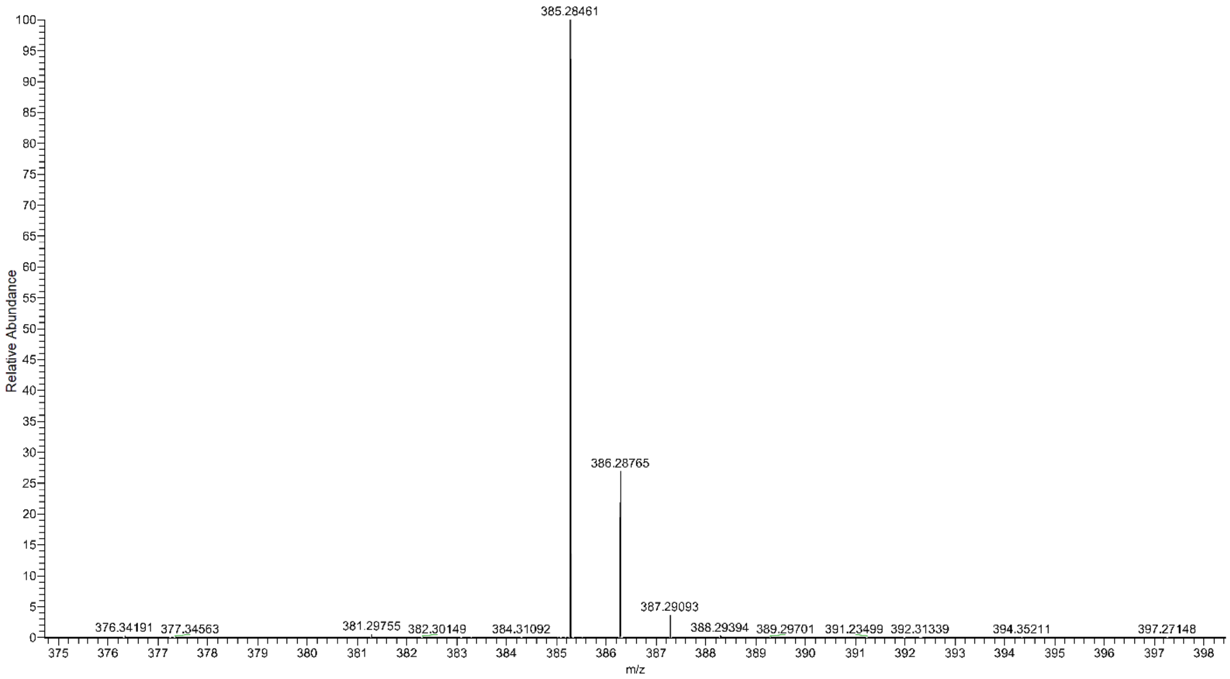


**Figure S57**. HRMS spectrum of compound **38**.

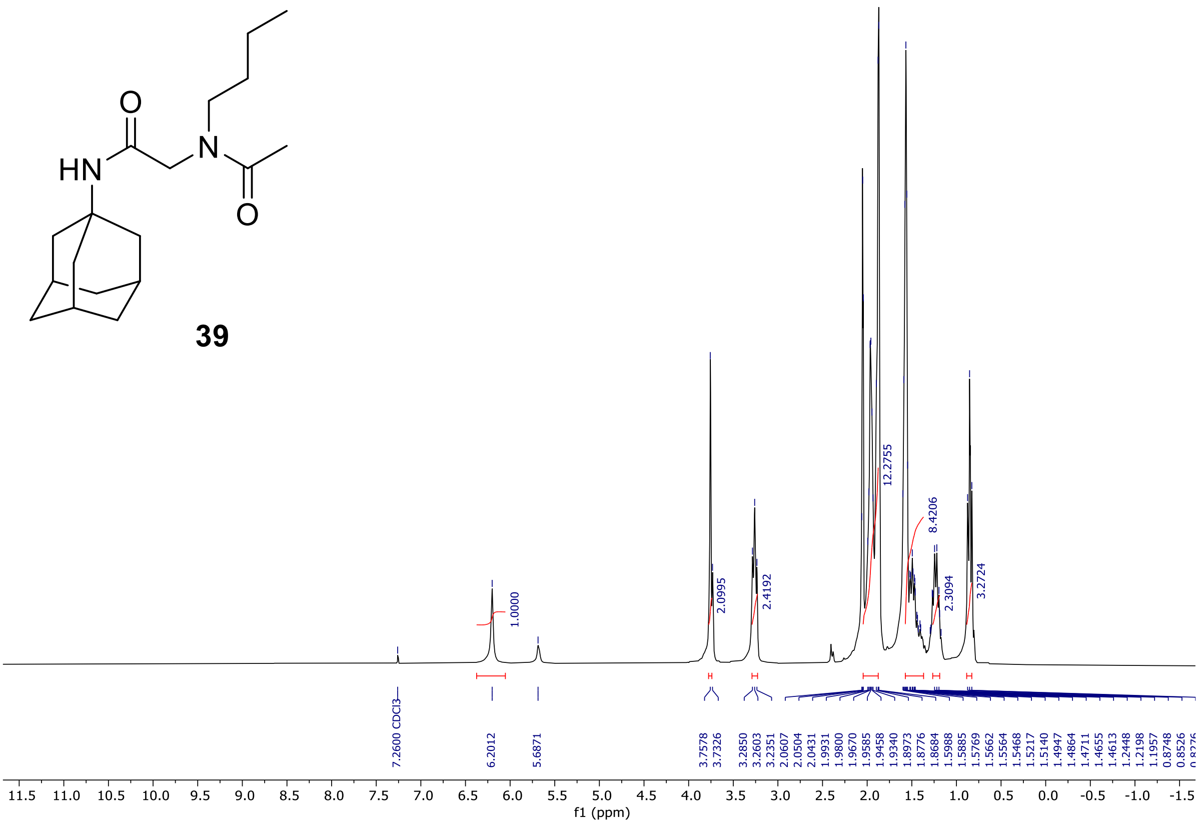


**Figure S58**. $^{1}$H NMR spectrum of compound **39**.

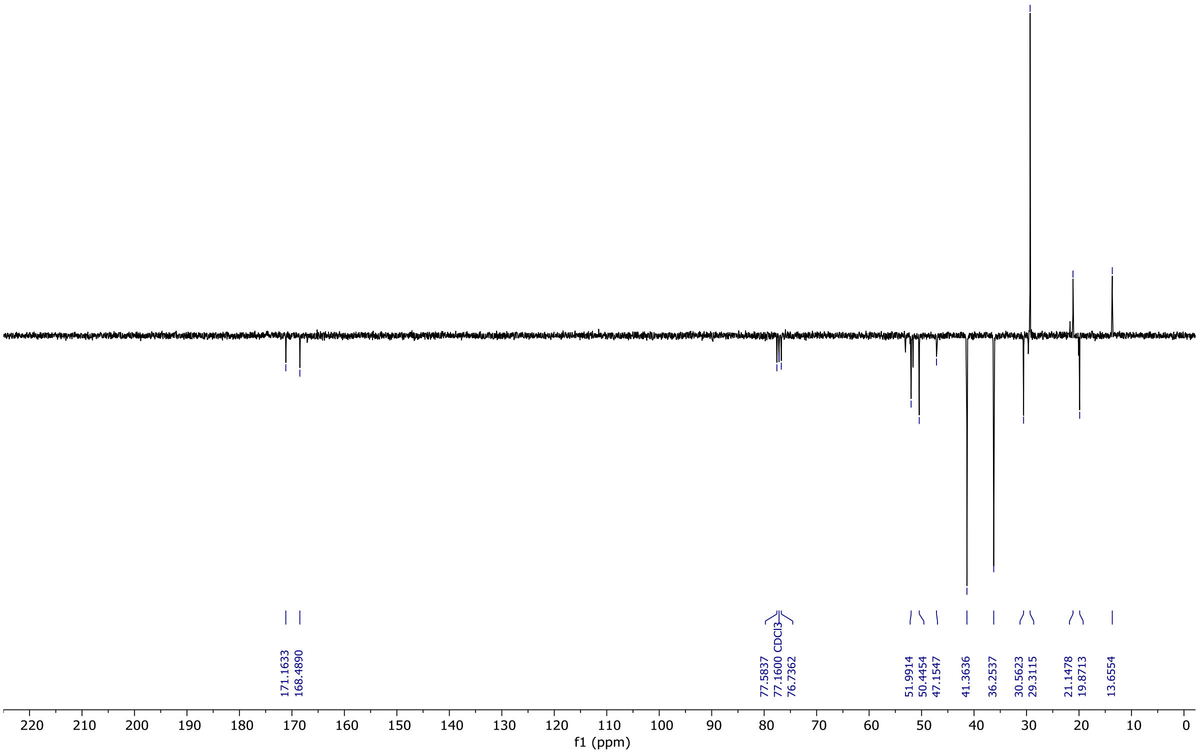


**Figure S59**. $^{13}$C APT NMR spectrum of compound **39**.

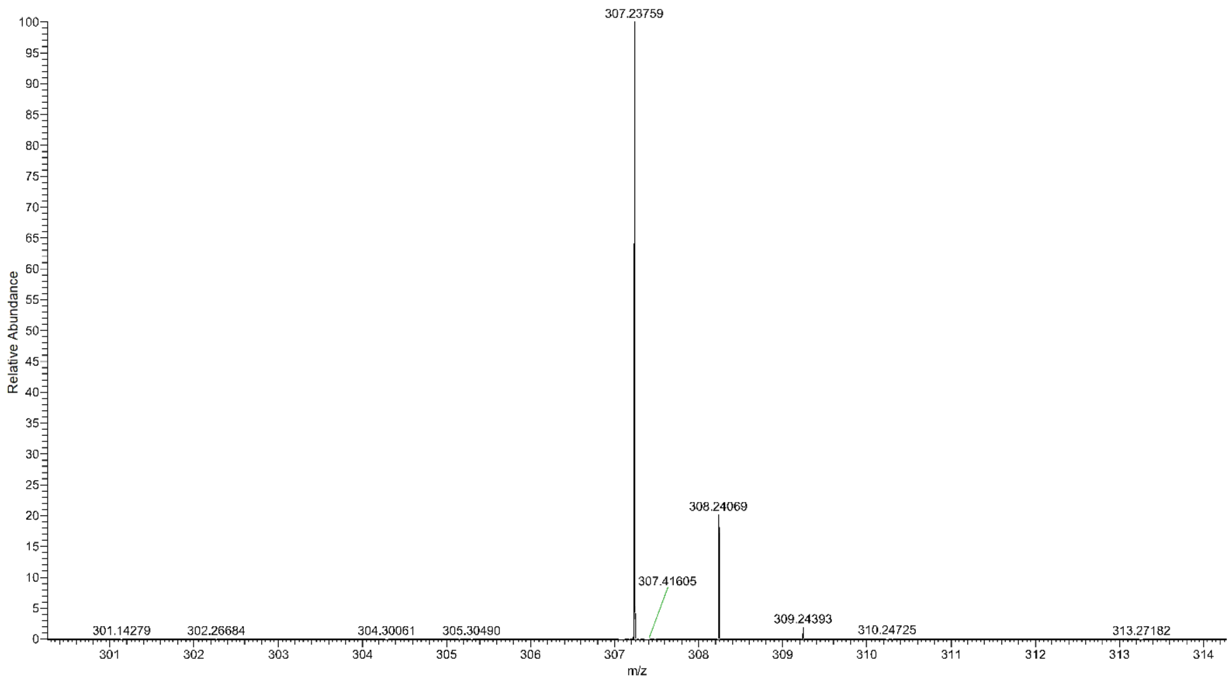


**Figure S60**. HRMS spectrum of compound **39**.

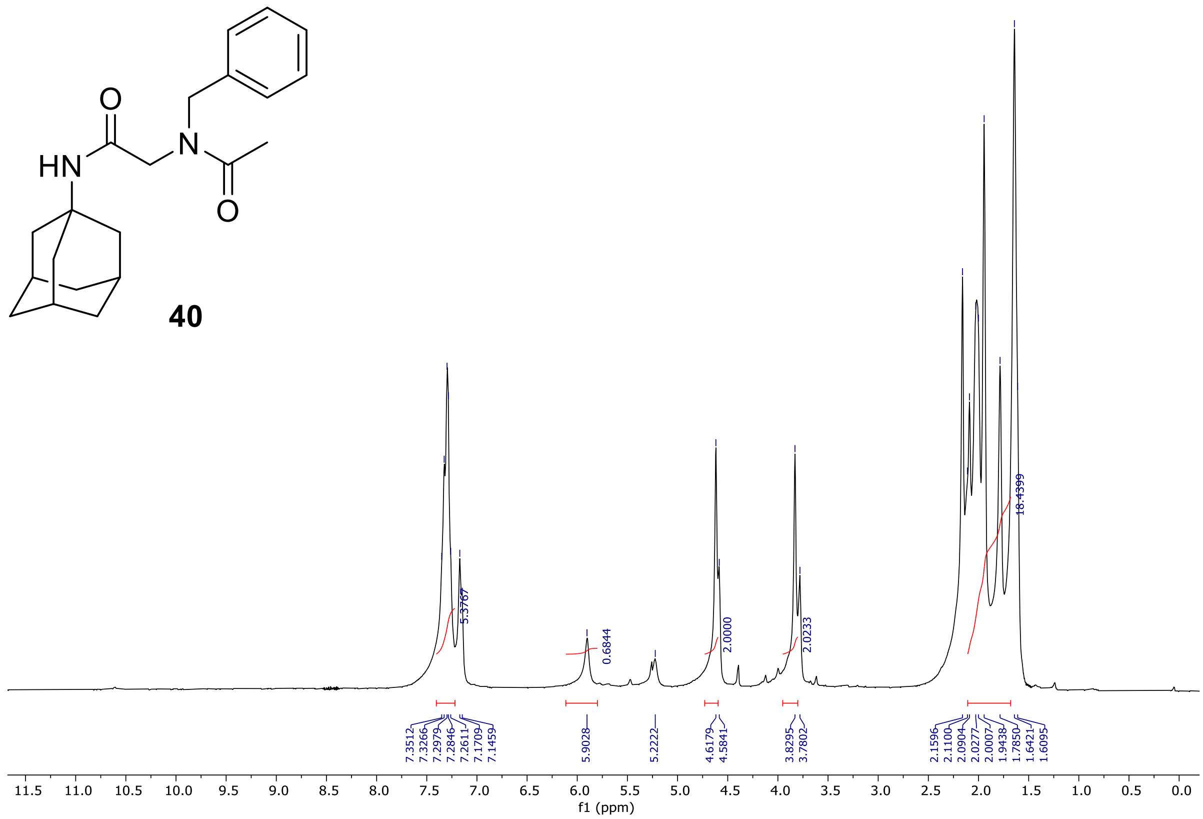


**Figure S61**. $^{1}$H NMR spectrum of compound **40**.

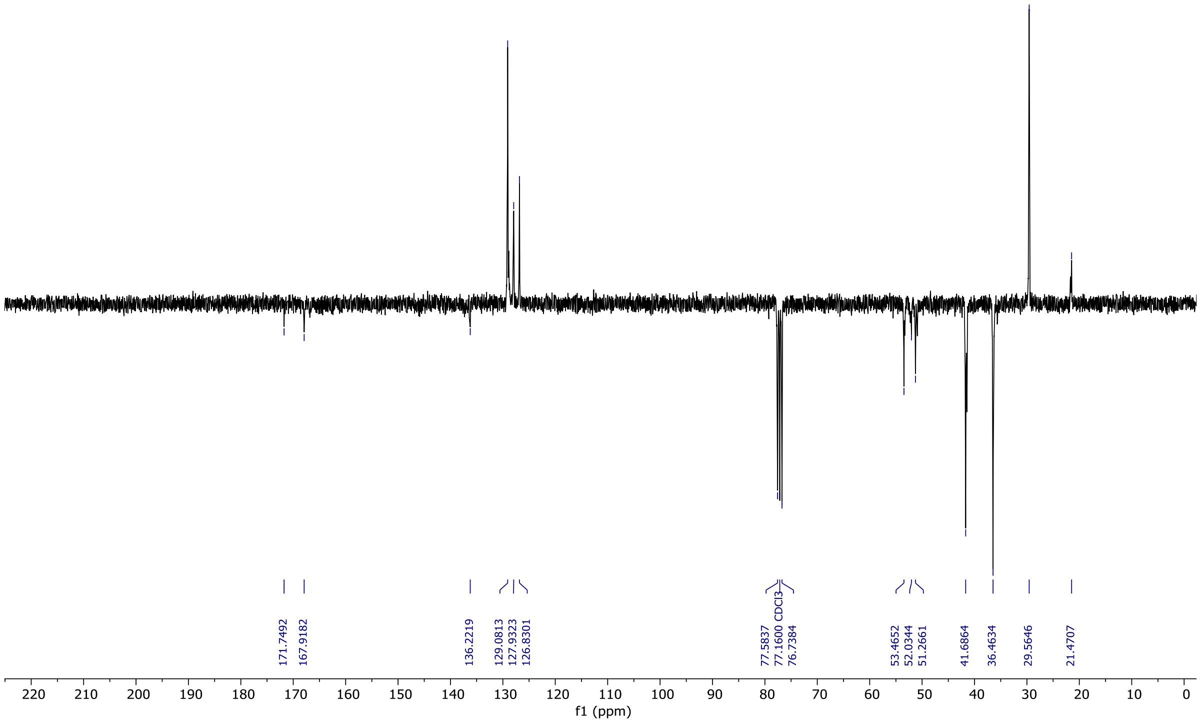


**Figure S62**. $^{13}$C APT NMR spectrum of compound **40**.

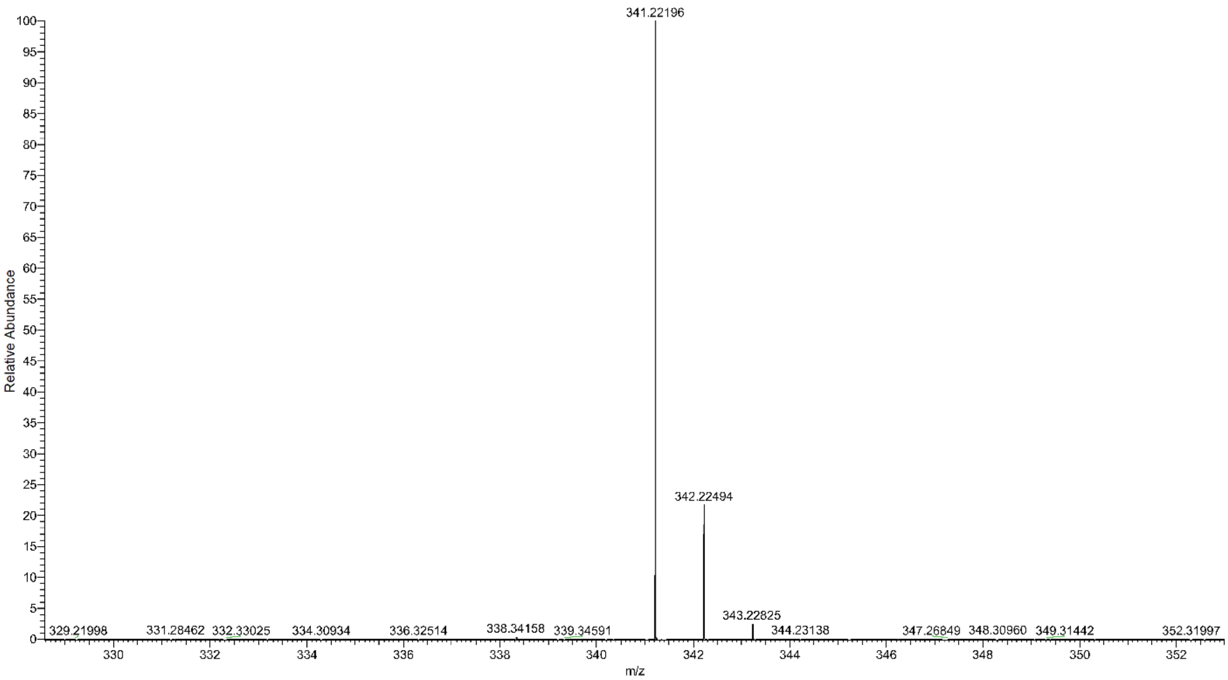


**Figure S63**. HRMS spectrum of compound **40**.

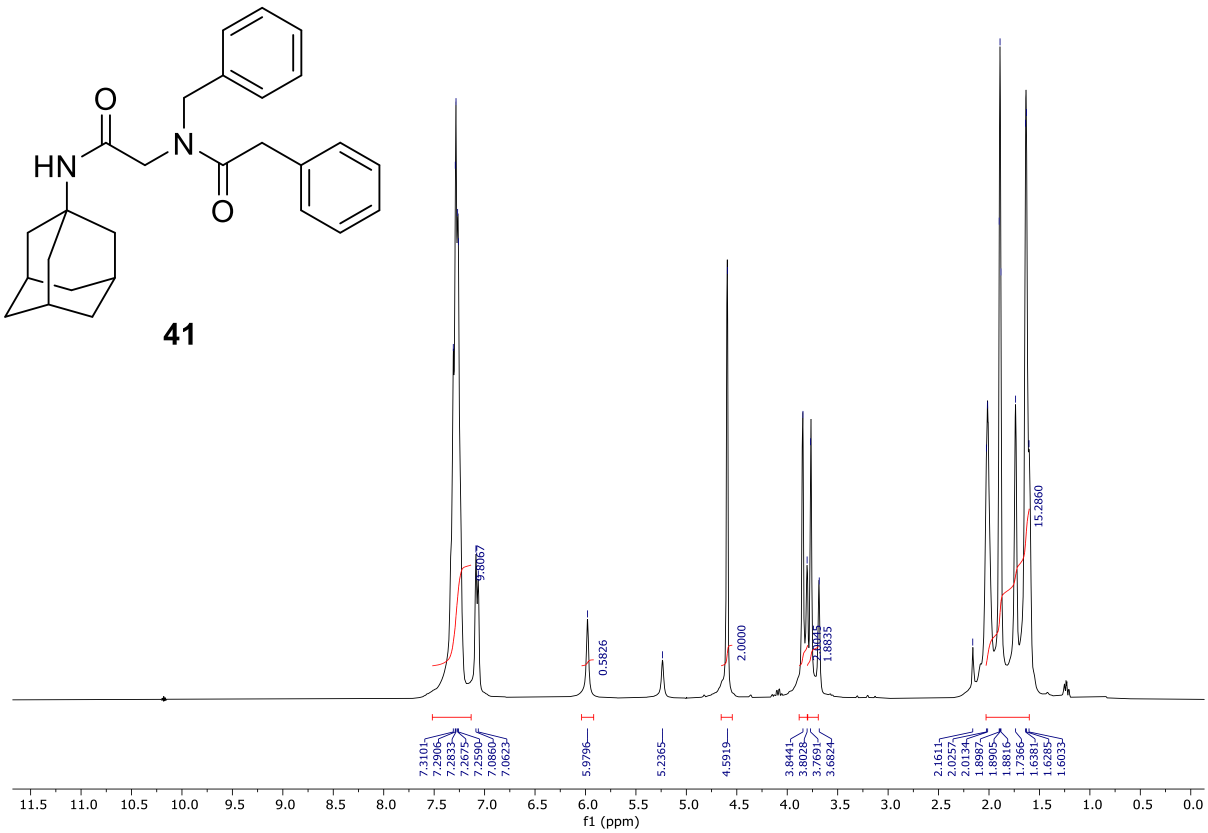


**Figure S64**. $^{1}$H NMR spectrum of compound **41**.

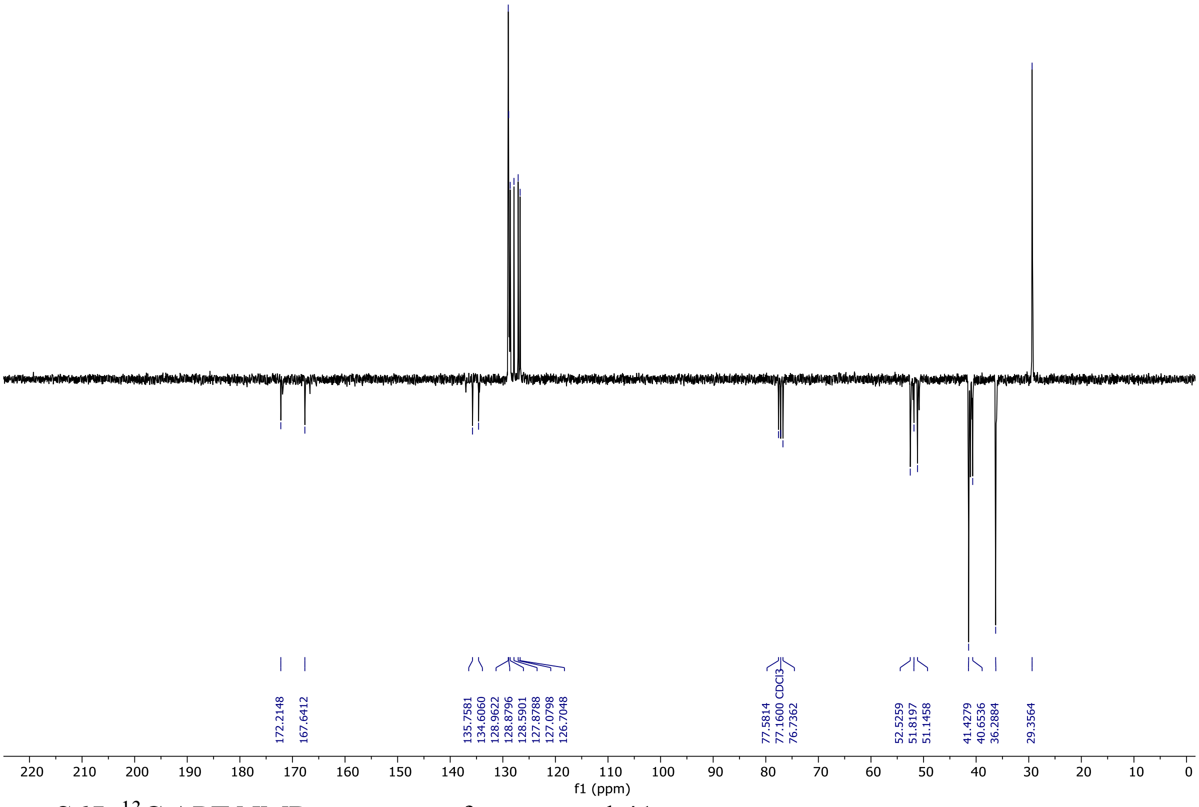


**Figure S65**. $^{13}$C APT NMR spectrum of compound **41**.

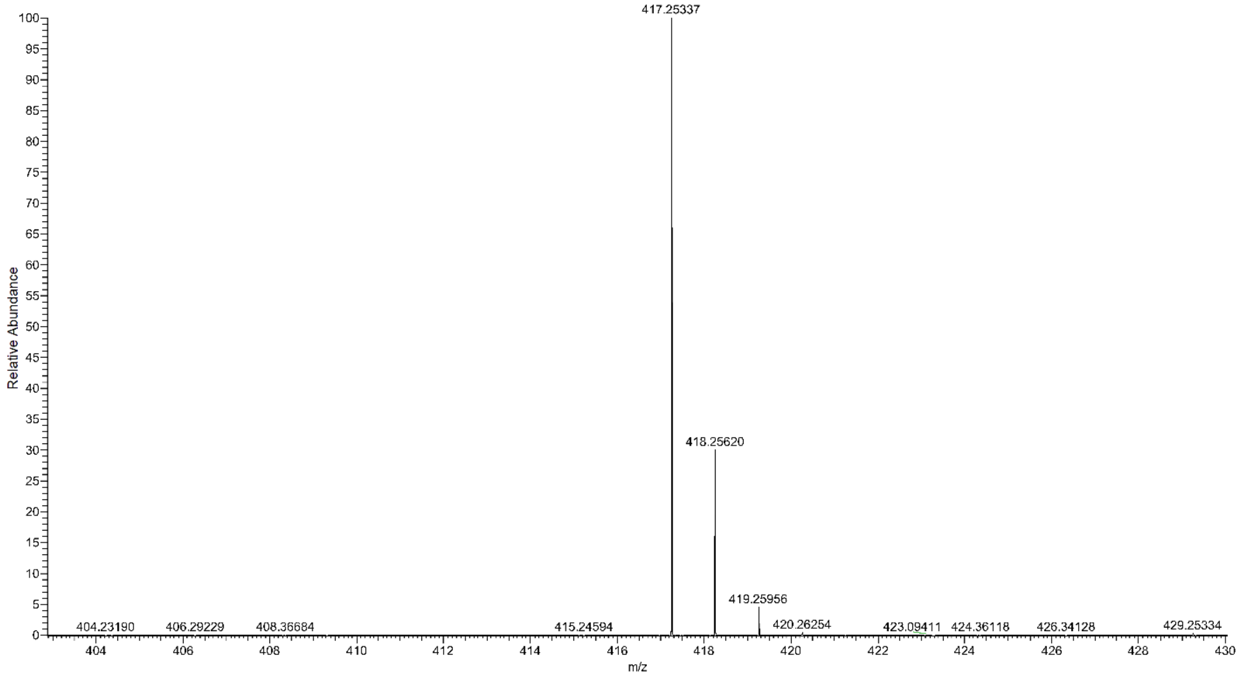


**Figure S66**. HRMS spectrum of compound **41**.

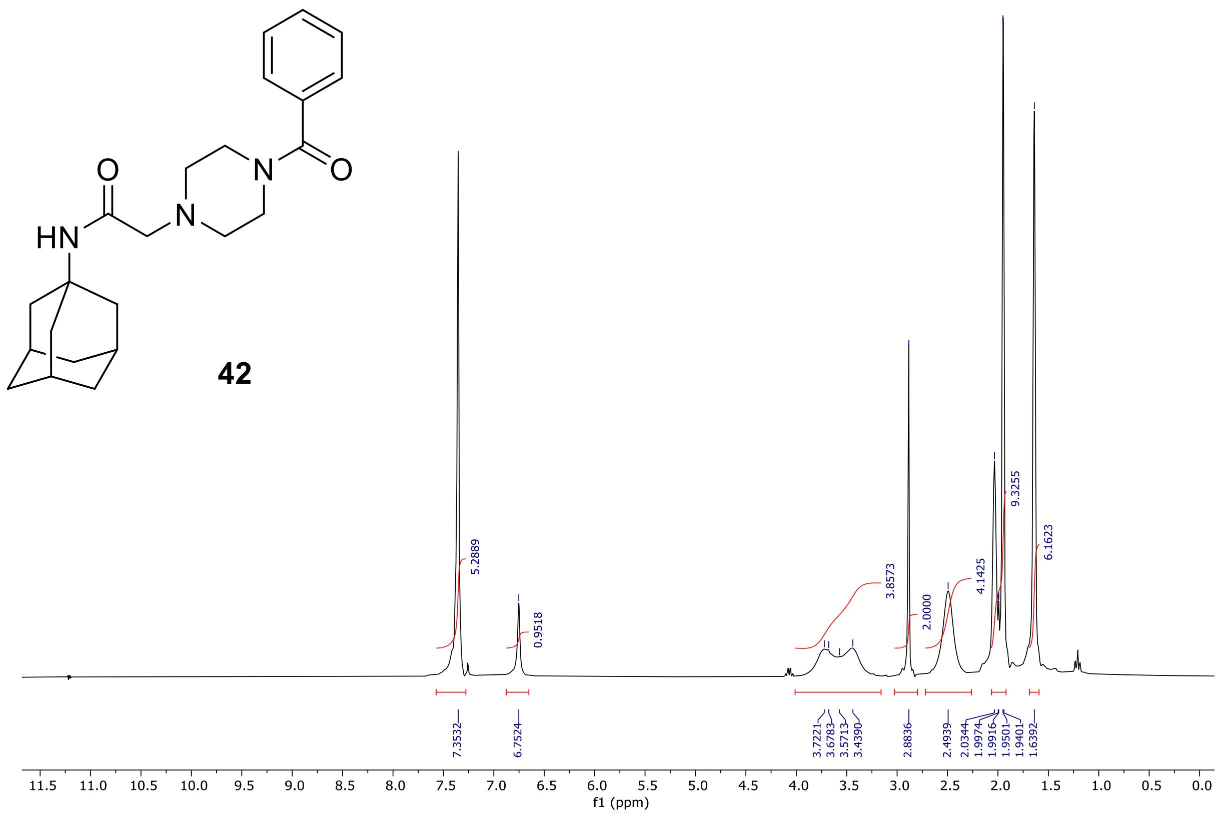


**Figure S67**. $^{1}$H NMR spectrum of compound **42**.

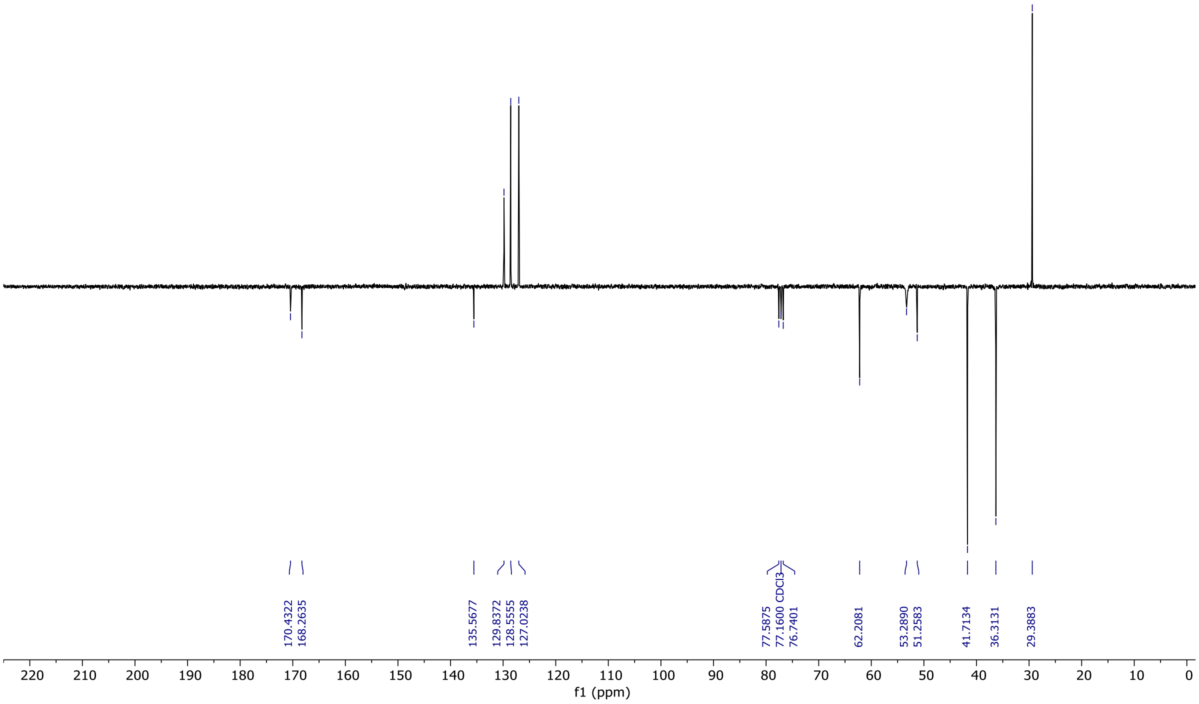


**Figure S68**. $^{13}$C APT NMR spectrum of compound **42**.

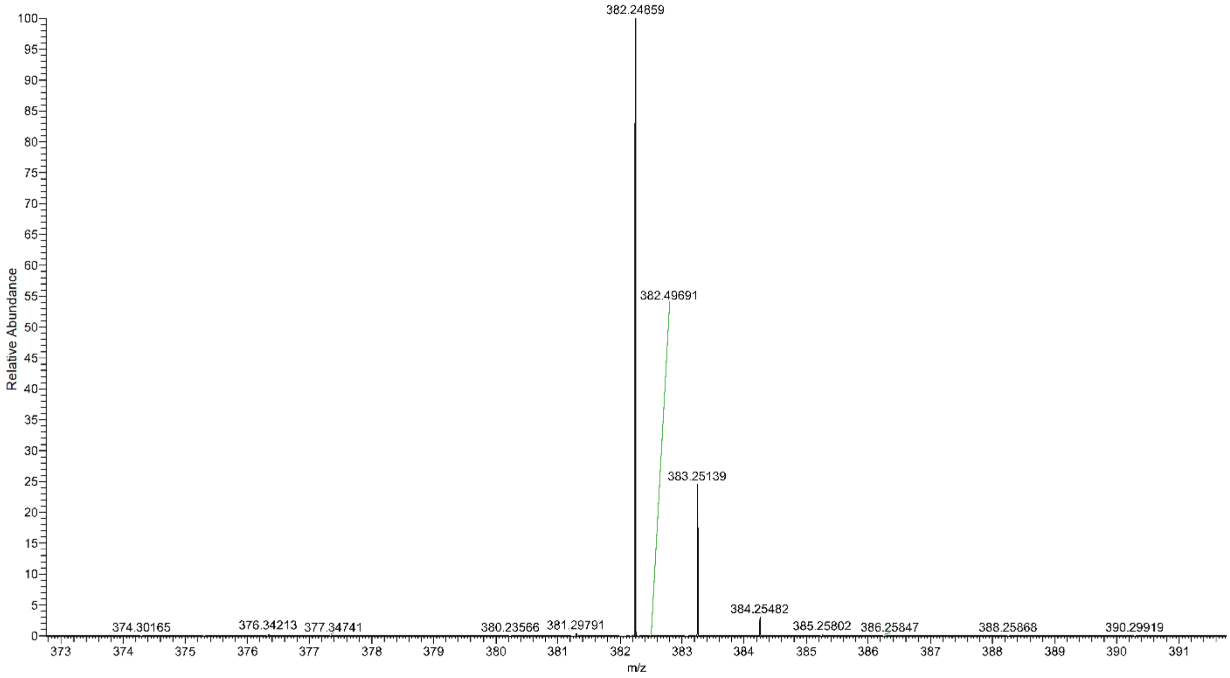


**Figure S69**. HRMS spectrum of compound **42**.

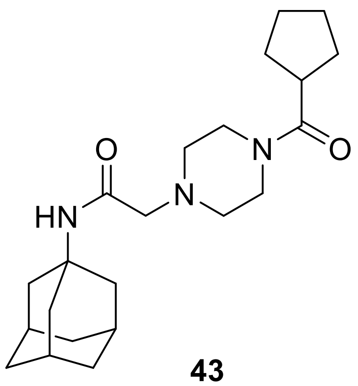

**43**

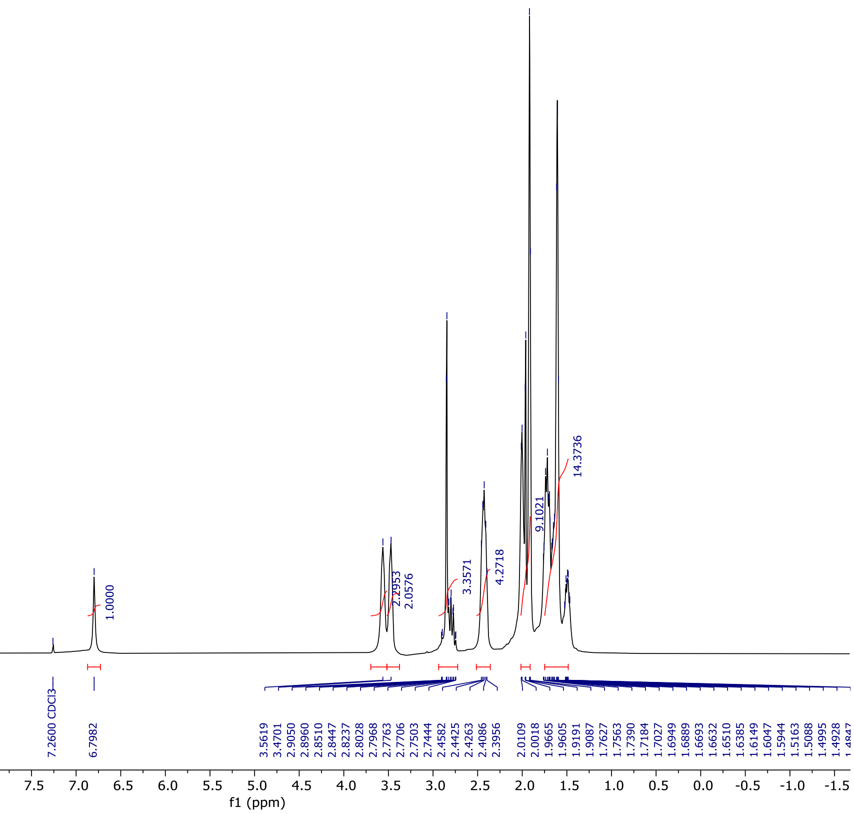


**Figure S70**. $^{1}$H NMR spectrum of compound **43**.

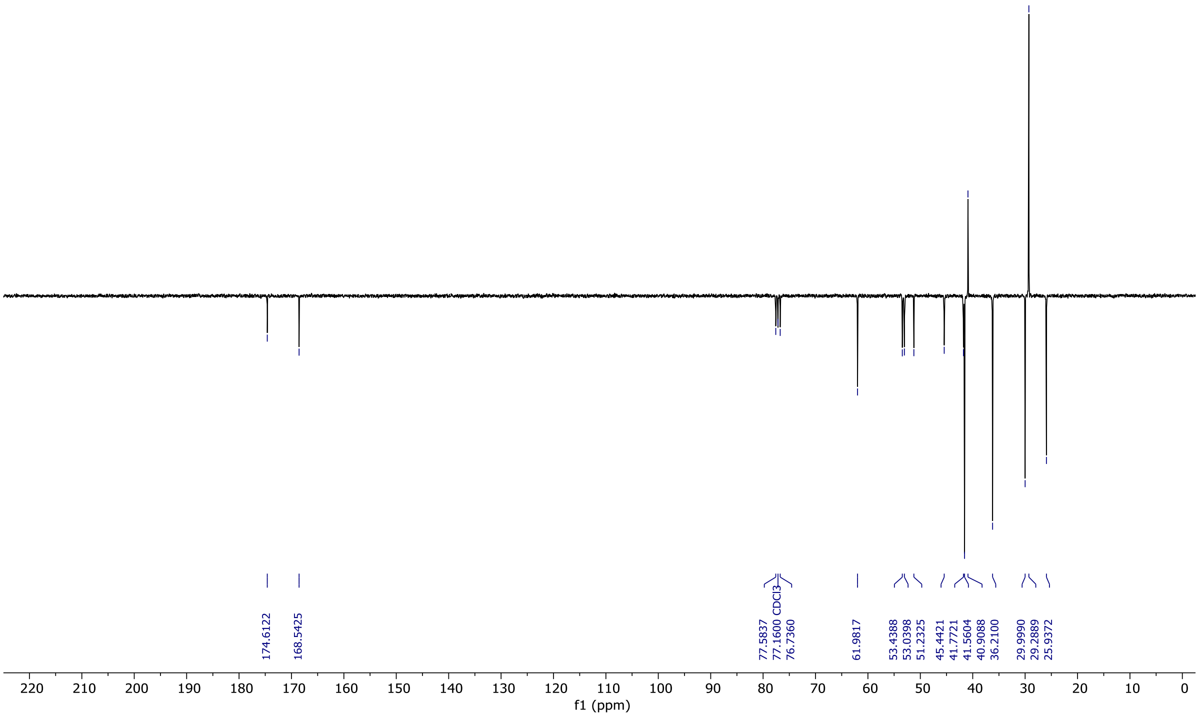


**Figure S71**. $^{13}$C APT NMR spectrum of compound **SH 43**.

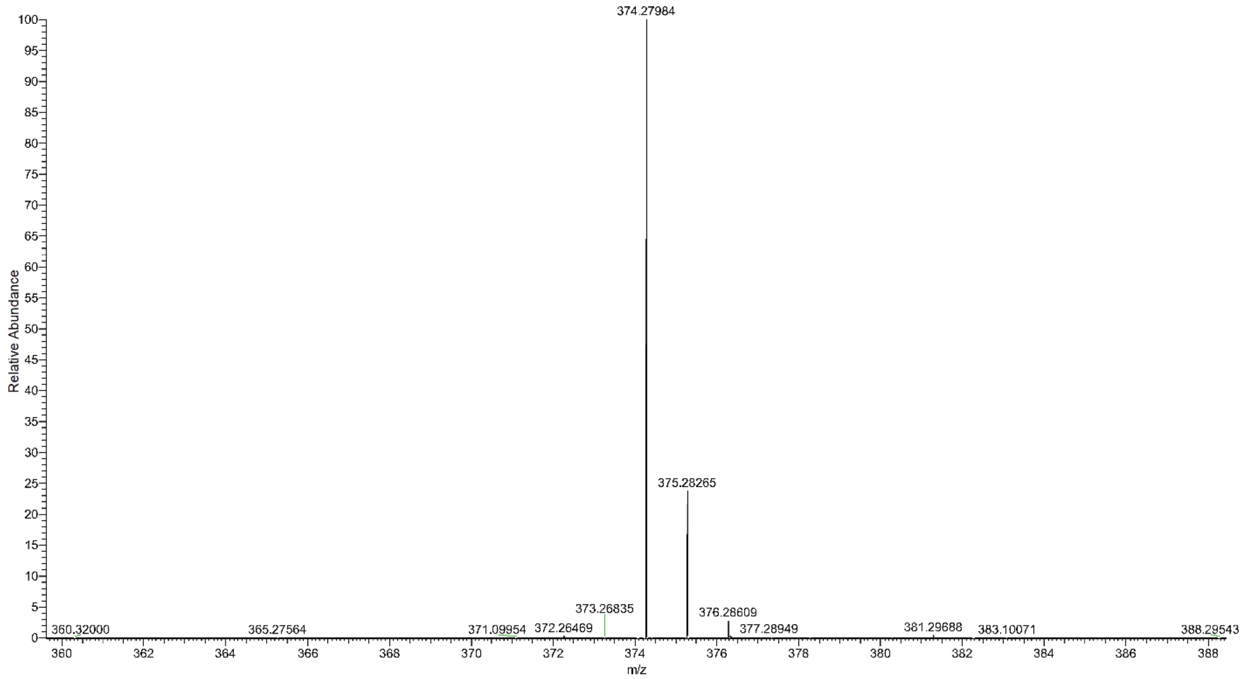


**Figure S72**. HRMS spectrum of compound **43**.

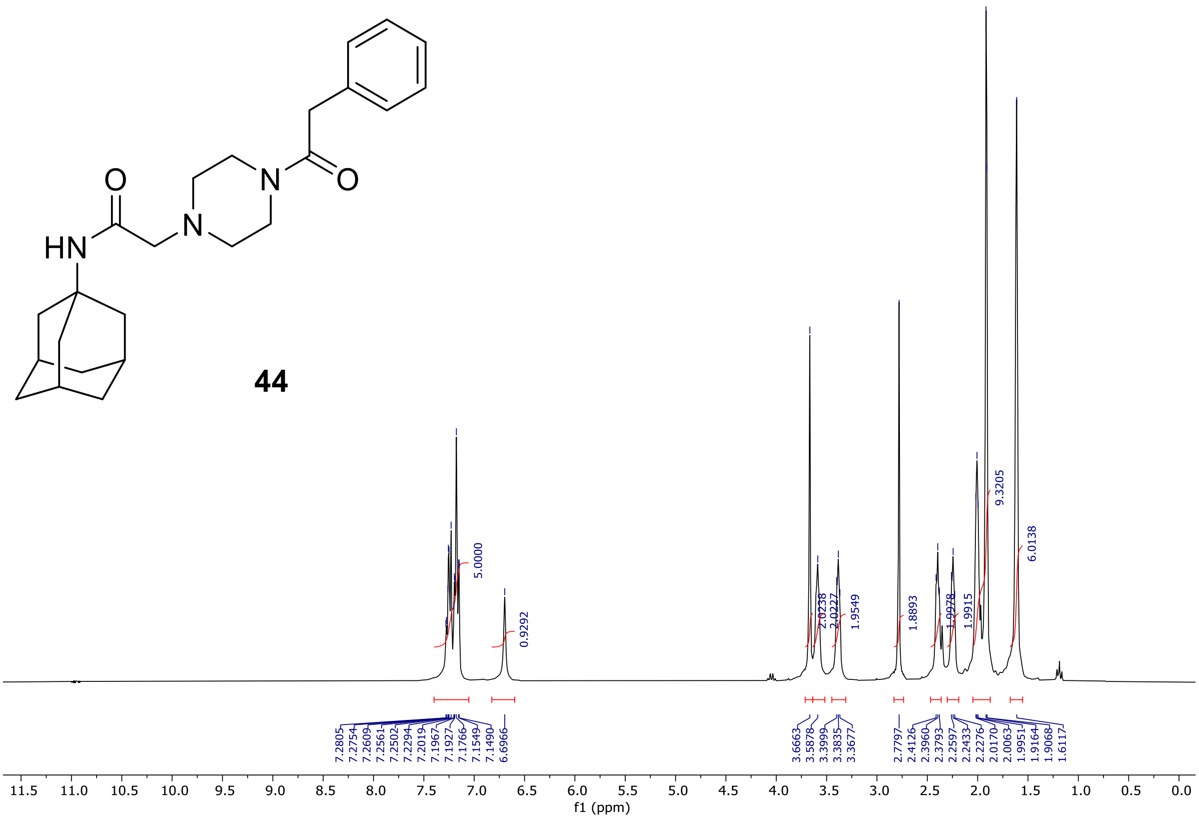


**Figure S73**. $^{1}$H NMR spectrum of compound **44**.

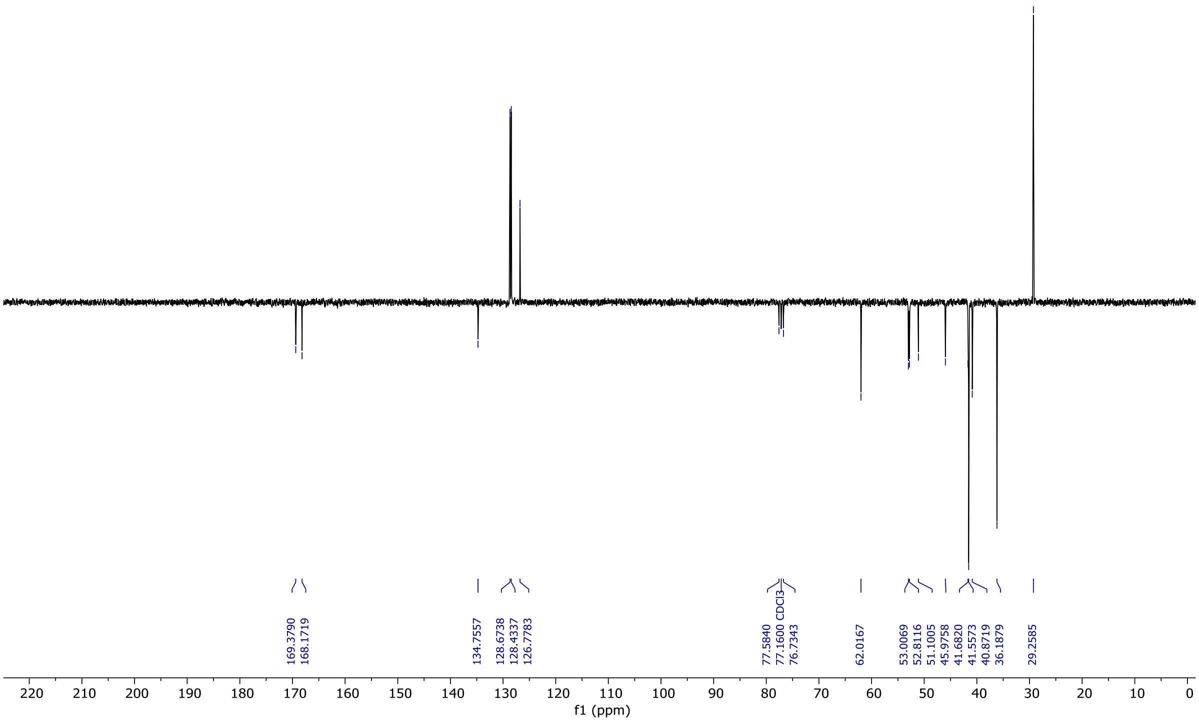


**Figure S74**. $^{13}$C APT NMR spectrum of compound **44**.

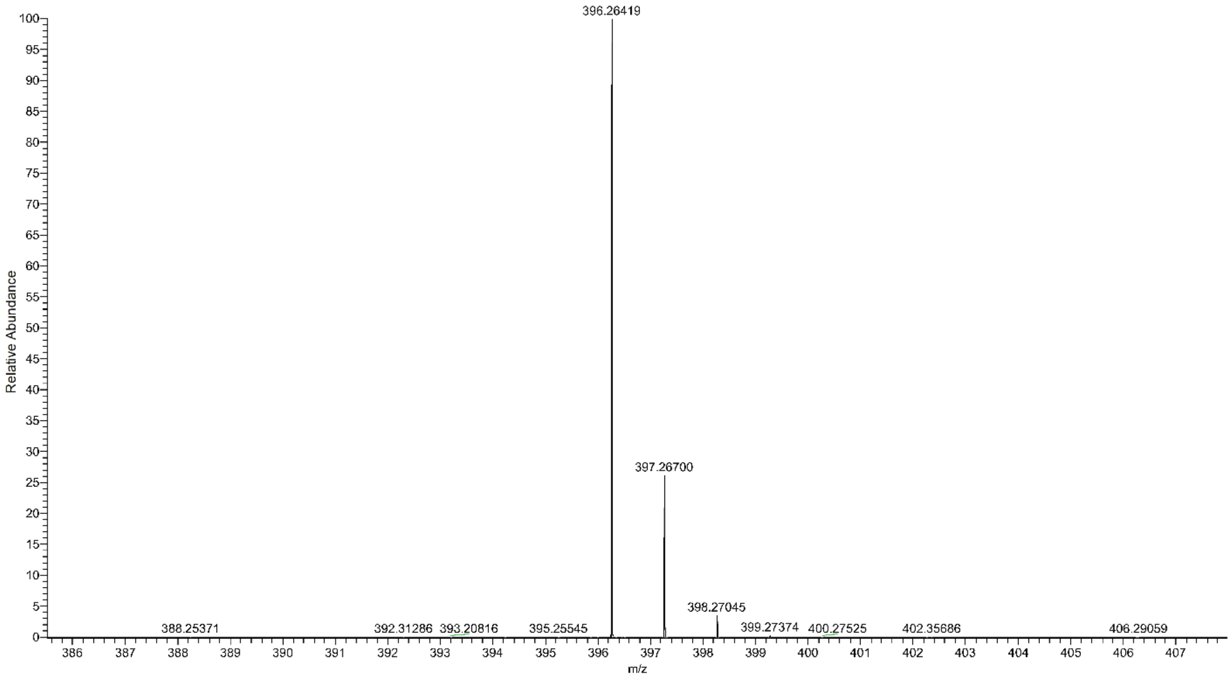


**Figure S75**. HRMS spectrum of compound **44**.

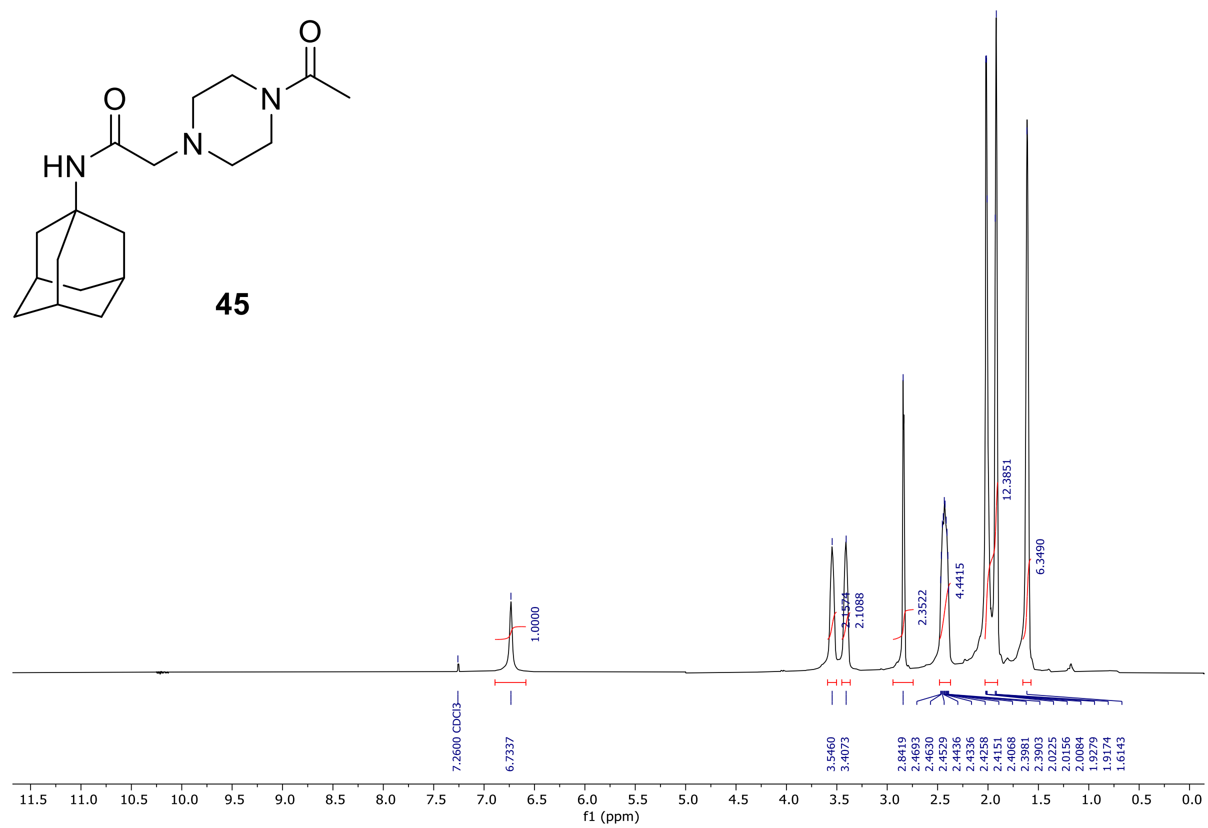


**Figure S76**. $^{1}$H NMR spectrum of compound **45**.

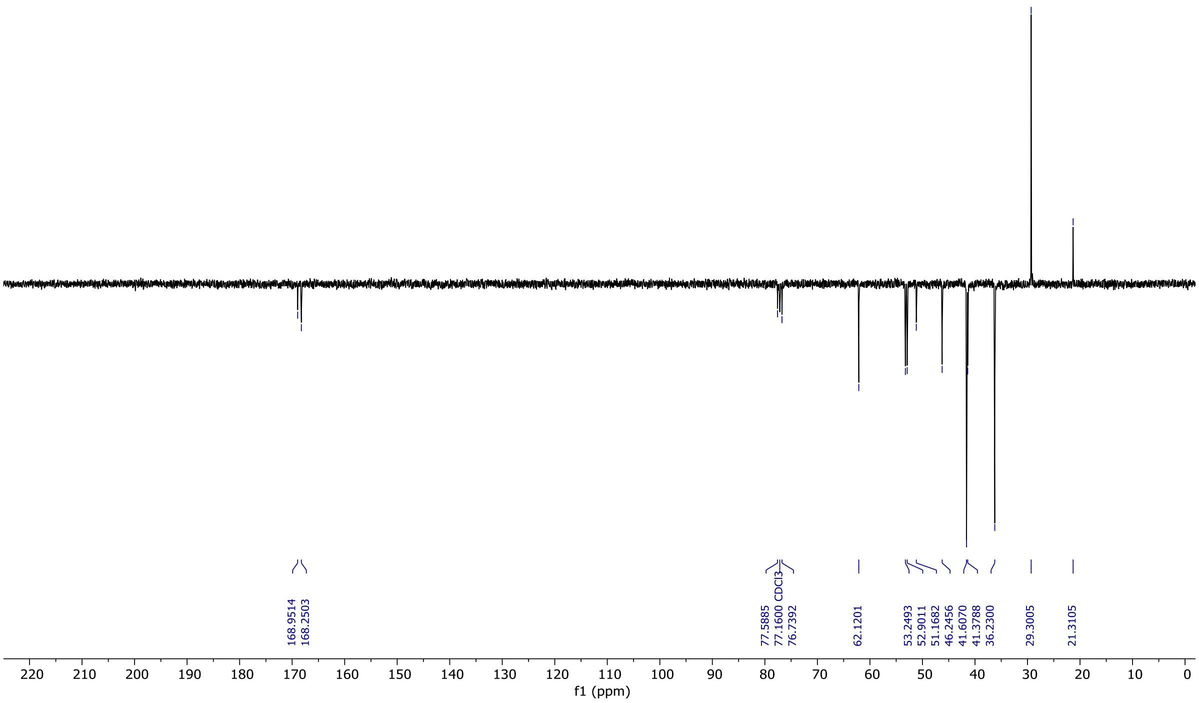


**Figure S77**. $^{13}$C APT NMR spectrum of compound **45**.

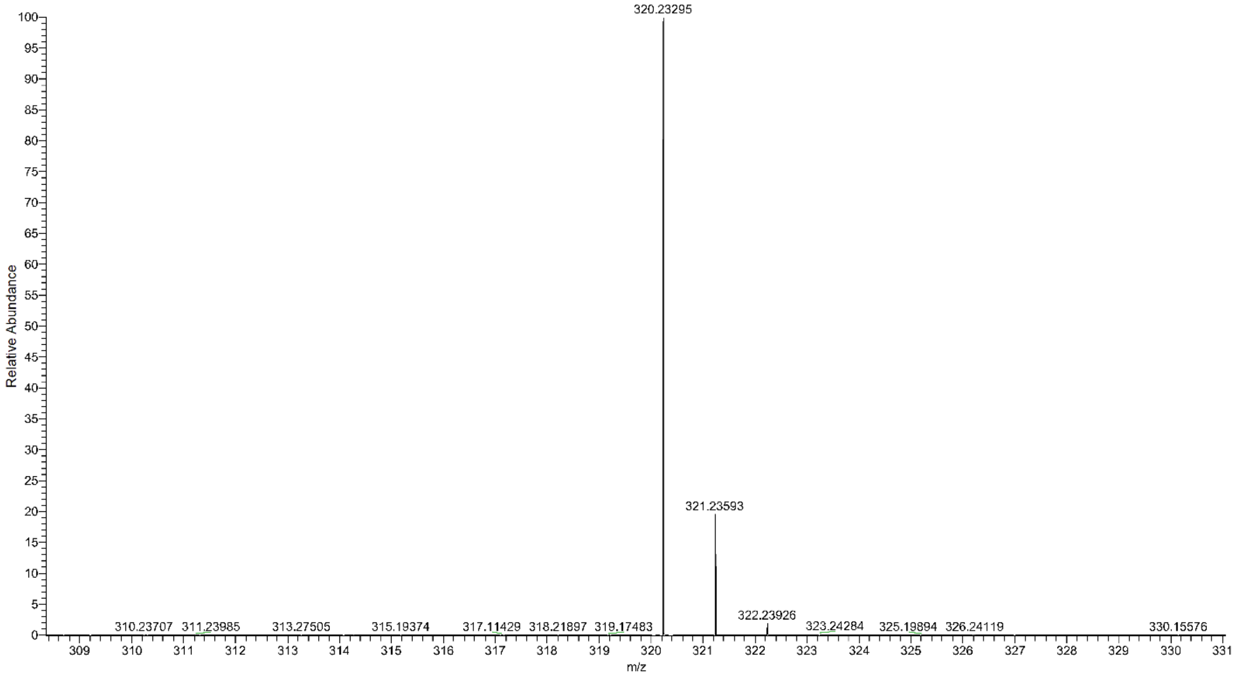


**Figure S78**. HRMS spectrum of compound **45**.

## LC-UV method for purity and metabolic stability evaluation.

| | |
|---|---|
| Instrumentation: | Shimadzu HPLC system LC-10AD series |
| Column: | Luna C8(2) (150 × 4.6 mm, 5 μm $d_p$) (Phenomenex). |
| Mobile Phase: | Phase A: 0.2% formic acid in water HPLC grade<br>Phase B: 0.2% formic acid in acetonitrile HPLC grade |
| Analysis mode: | Gradient of concentration: 0.00 min (%B=10), 1.00 min (%B=10), 5.00 min (%B=50), 11.00 min (%B=50), 11.50 min (%B=10), 16.00 min (%B=10). |
| Detection: | λ= 282 nm (for **23** metabolic stability); 220, 254 or 282 nm (for purity) |
| Flow rate: | 1 mL/min |
| Injected volume: | 20 µL |

## LC-HRMS metabolic profile of compound 23 in mouse liver microsomes.

Instrumentation: Hybrid quadrupole-orbitrap, Thermo Scientific Q-exactive *Plus*, equipped with a Vanquish UHPLC system.

Column: Luna C8(2) (150 × 3 mm, 5 µm $d_p$) (Phenomenex).

Mobile Phase: Phase A: 0.1% formic acid in water UHPLC grade.
Phase B: 0.1% formic acid in acetonitrile UHPLC grade.

Analysis mode: Gradient of concentration: 0.00 min (%B=40), 1.00 min (%B=40), 9.00 min (%B=90), 14.50 min (%B=90), 15.00 min (%B=40), 20.00 min (%B=40)

Flow rate: 0.400 mL/min

Column temperature: 40 °C

Sample temperature: 15 °C

Injected volume: 5 µL

**Table S1.** Operating conditions of the HESI.

| Parameter | Value |
|---|---|
| sheath gas flow rate ($N_2$) | 45 Auxiliary Units |
| auxiliary gas flow rate | 10 Auxiliary Units |
| sweep gas flow rate | 1 Auxiliary Units |
| spray voltage | 3.50 kV |
| capillary temperature | 300 °C |
| auxiliary gas heater temperature | 350 °C |

**Table S2.** Detector acquisition modes.

| + Full scan | Value |
|---|---|
| Microscan | 1 |
| Resolution | 70,000 |
| AGC target | 3e6 |

| +PRM ($MS^2$) | Value |
|---|---|
| Microscan | 1 |
| Resolution | 35,000 |
| AGC target | 1e5 |
| Maximum IT | 120 ms |
| Loop count | 1 |
| MSX count | 1 |
| Isolation wind. | 1.3 *m/z* |
| Isolation offset | 0.5 *m/z* |
| NCE | 20, 40, 80 |

| + dd$MS^2$ | Value |
|---|---|
| Microscan | 1 |
| Resolution | 17,500 |
| AGC target | 1e5 |
| Maximum IT | 60 ms |
| Loop count | 4 |
| MSX count | 1 |
| Isolation wind. | 2.0 *m/z* |
| Isolation offset | 0.0 *m/z* |
| NCE | 20, 40, 80 |

**Figure S79.** Proposed structures, mass spectra ($MS^2$) data and fragment ions interpretation of the detected metabolites **M1-M9** of compound **23** in mouse liver microsomes.

- **23**

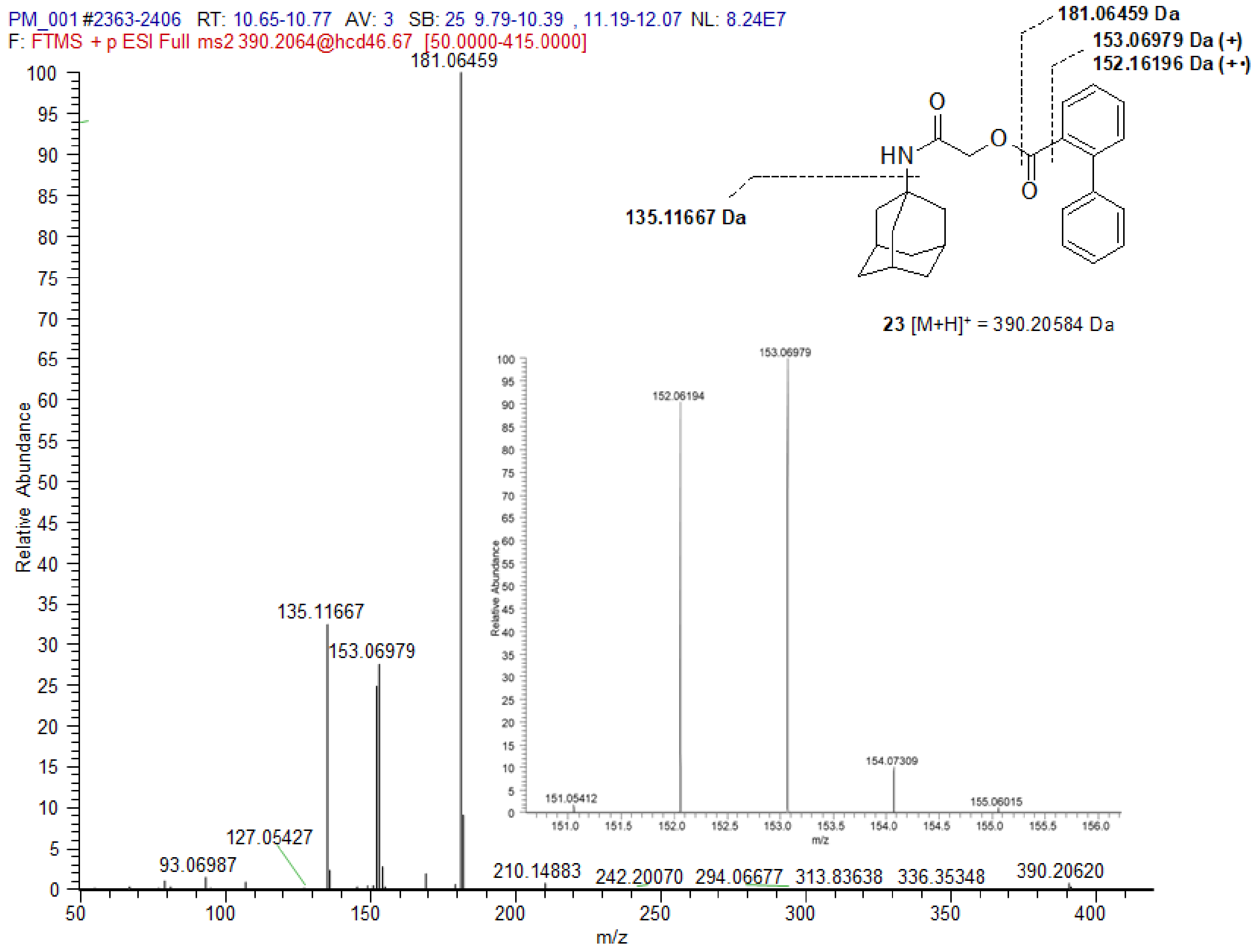

- **M1a**

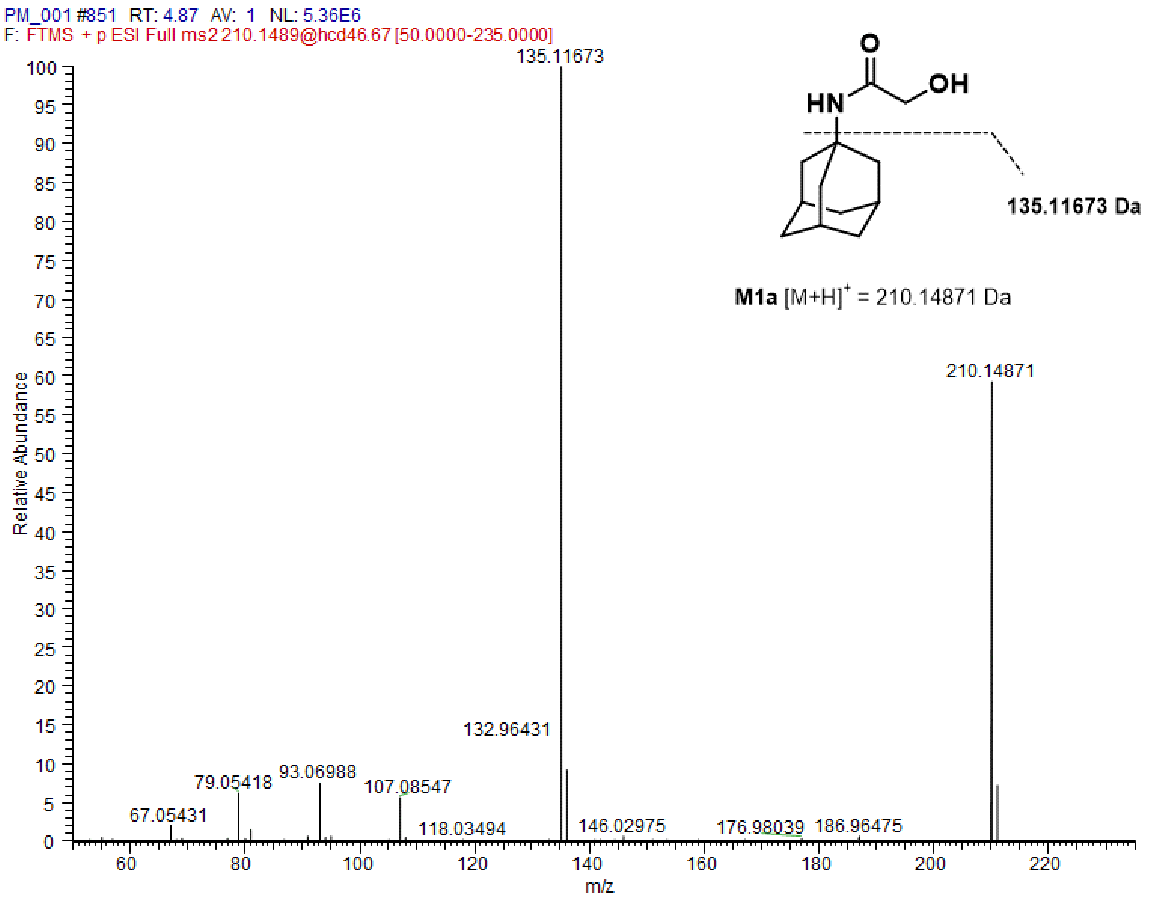
PM_001 #851 RT: 4.87 AV: 1 NL: 5.36E6
F: FTMS + p ESI Full ms2 210.1489@hcd46.67 [50.0000-235.0000]
Relative Abundance
m/z
135.11673
210.14871
132.96431
79.05418
93.06988
107.08547
67.05431
118.03494
146.02975
176.98039
186.96475
HN
O
OH
135.11673 Da
M1a [M+H]+ = 210.14871 Da

- **M1b**

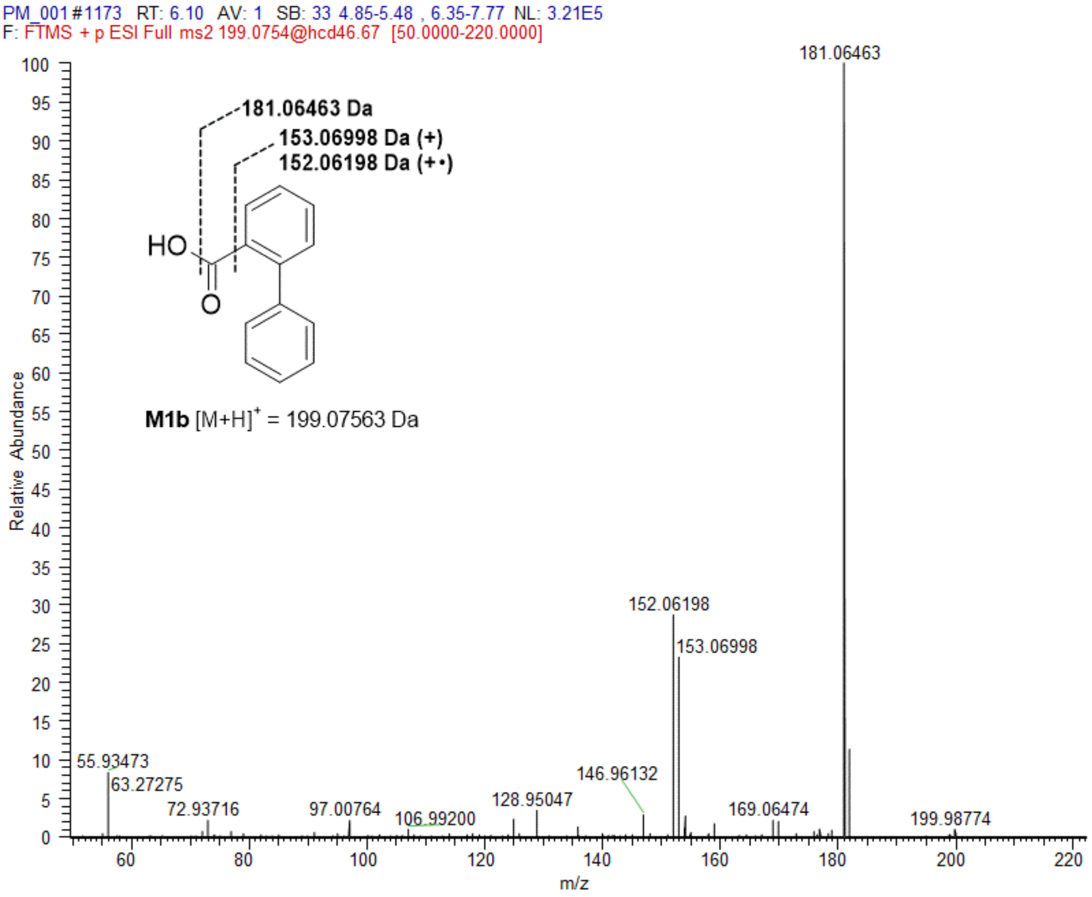

PM_001#1173 RT: 6.10 AV: 1 SB: 33 4.85-5.48 , 6.35-7.77 NL: 3.21E5
F: FTMS + p ESI Full ms2 199.0754@hcd46.67 [50.0000-220.0000]
181.06463 Da
153.06998 Da (+)
152.06198 Da (+•)
HO
O
M1b [M+H]+ = 199.07563 Da
Relative Abundance
m/z
181.06463
152.06198
153.06998
55.93473
63.27275
72.93716
97.00764
106.99200
128.95047
146.96132
169.06474
199.98774

- **M2**

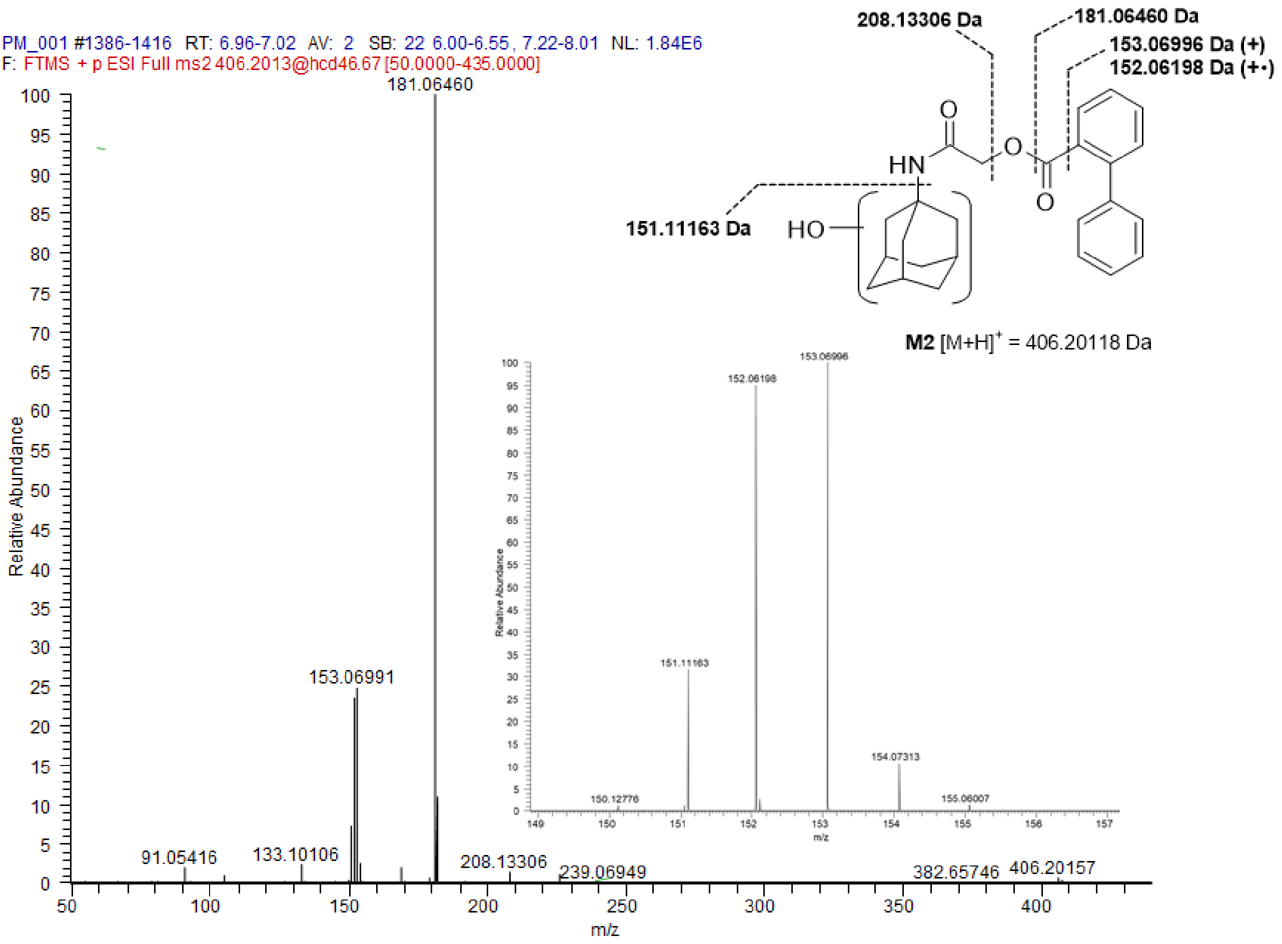

PM_001 #1386-1416 RT: 6.96-7.02 AV: 2 SB: 22 6.00-6.55 , 7.22-8.01 NL: 1.84E6
F: FTMS + p ESI Full ms2 406.2013@hcd46.67 [50.0000-435.0000]
Relative Abundance
m/z
181.06460
153.06991
91.05416
133.10106
208.13306
239.06949
382.65746
406.20157
208.13306 Da
181.06460 Da
153.06996 Da (+)
152.06198 Da (+•)
151.11163 Da
HO
HN
O
M2 [M+H]+ = 406.20118 Da
153.06996
152.06198
151.11163
154.07313
150.12776
155.06007

- **M3**

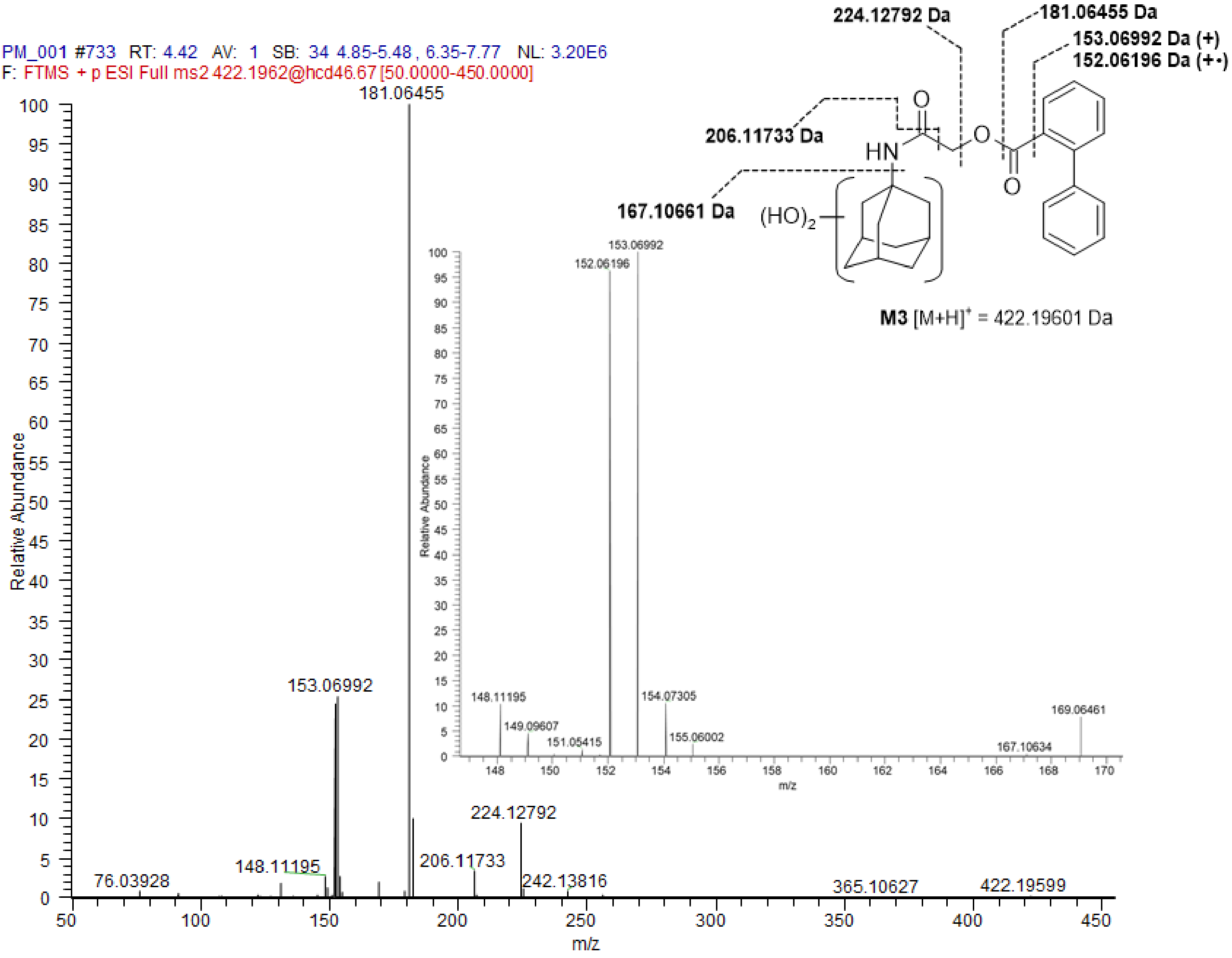

224.12792 Da
181.06455 Da
153.06992 Da (+)
152.06196 Da (+•)
206.11733 Da
167.10661 Da
(HO)2
HN
O
M3 [M+H]+ = 422.19601 Da
PM_001 #733 RT: 4.42 AV: 1 SB: 34 4.85-5.48 , 6.35-7.77 NL: 3.20E6
F: FTMS + p ESI Full ms2 422.1962@hcd46.67 [50.0000-450.0000]
Relative Abundance
m/z
181.06455
153.06992
224.12792
206.11733
242.13816
148.11195
76.03928
365.10627
422.19599
152.06196
154.07305
149.09607
151.05415
155.06002
169.06461
167.10634

- **M4**

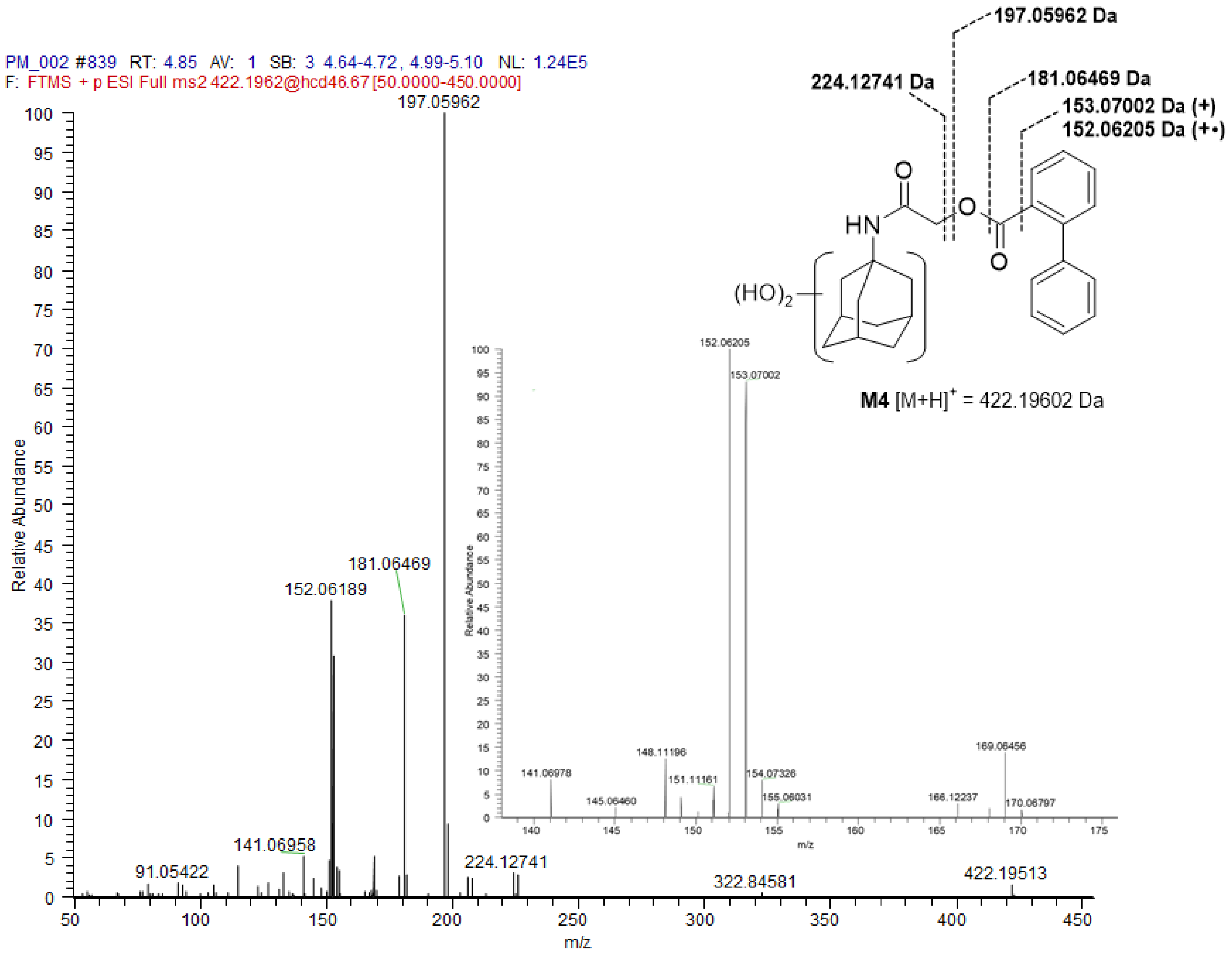
PM_002 #839 RT: 4.85 AV: 1 SB: 3 4.64-4.72 , 4.99-5.10 NL: 1.24E5
F: FTMS + p ESI Full ms2 422.1962@hcd46.67 [50.0000-450.0000]
197.05962
181.06469
152.06189
141.06958
91.05422
224.12741
322.84581
422.19513
Relative Abundance
m/z
152.06205
153.07002
148.11196
151.11161
141.06978
145.06460
154.07326
155.06031
169.06456
166.12237
170.06797
197.05962 Da
224.12741 Da
181.06469 Da
153.07002 Da (+)
152.06205 Da (+•)
HN
O
(HO)2
M4 [M+H]+ = 422.19602 Da

- **M5**

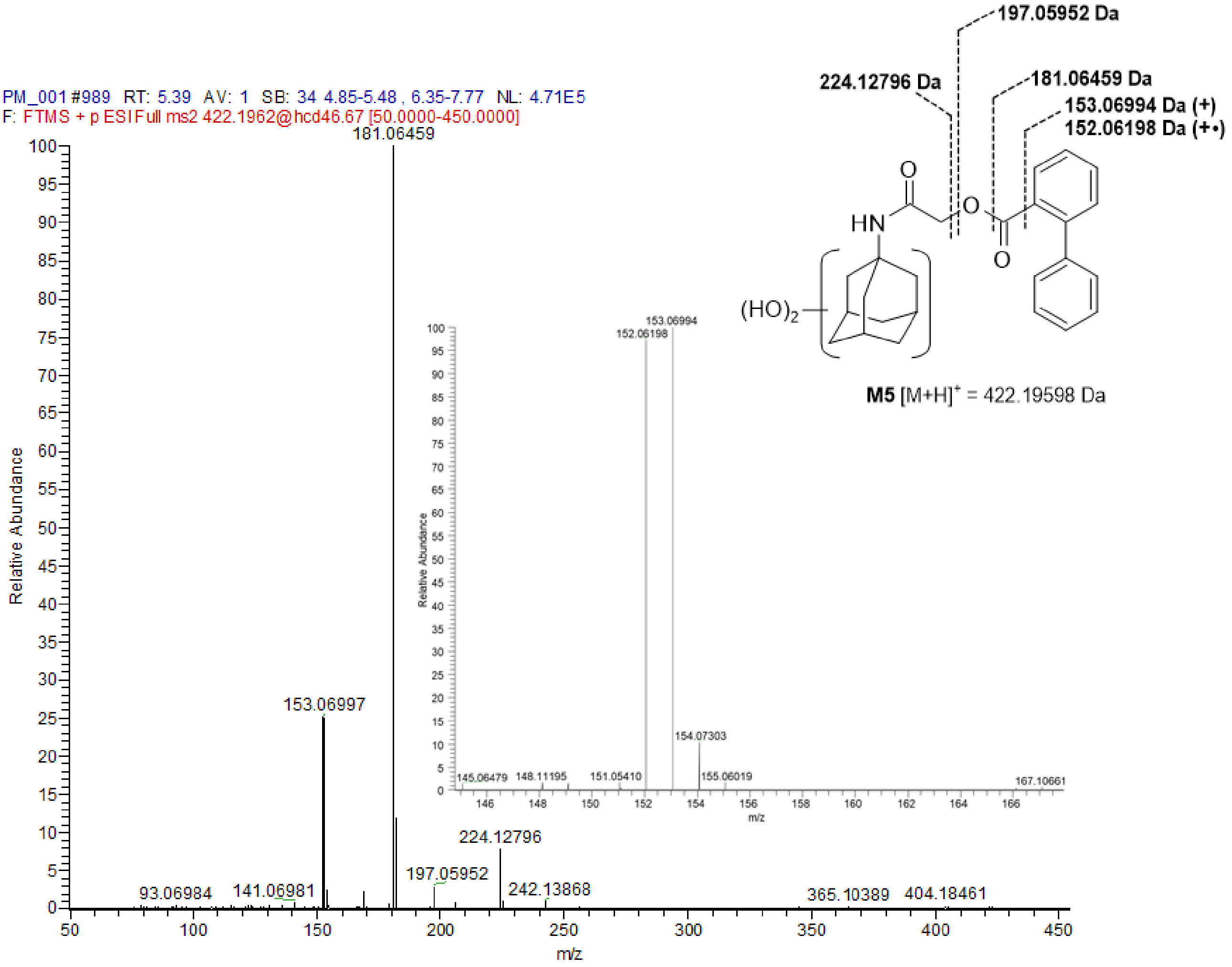
PM_001 #989 RT: 5.39 AV: 1 SB: 34 4.85-5.48 , 6.35-7.77 NL: 4.71E5
F: FTMS + p ESI Full ms2 422.1962@hcd46.67 [50.0000-450.0000]
181.06459
153.06997
224.12796
197.05952
242.13868
93.06984
141.06981
365.10389
404.18461
Relative Abundance
m/z
153.06994
152.06198
154.07303
145.06479
148.11195
151.05410
155.06019
167.10661
197.05952 Da
224.12796 Da
181.06459 Da
153.06994 Da (+)
152.06198 Da (+•)
HN
O
(HO)2
M5 [M+H]+ = 422.19598 Da

- **M6**

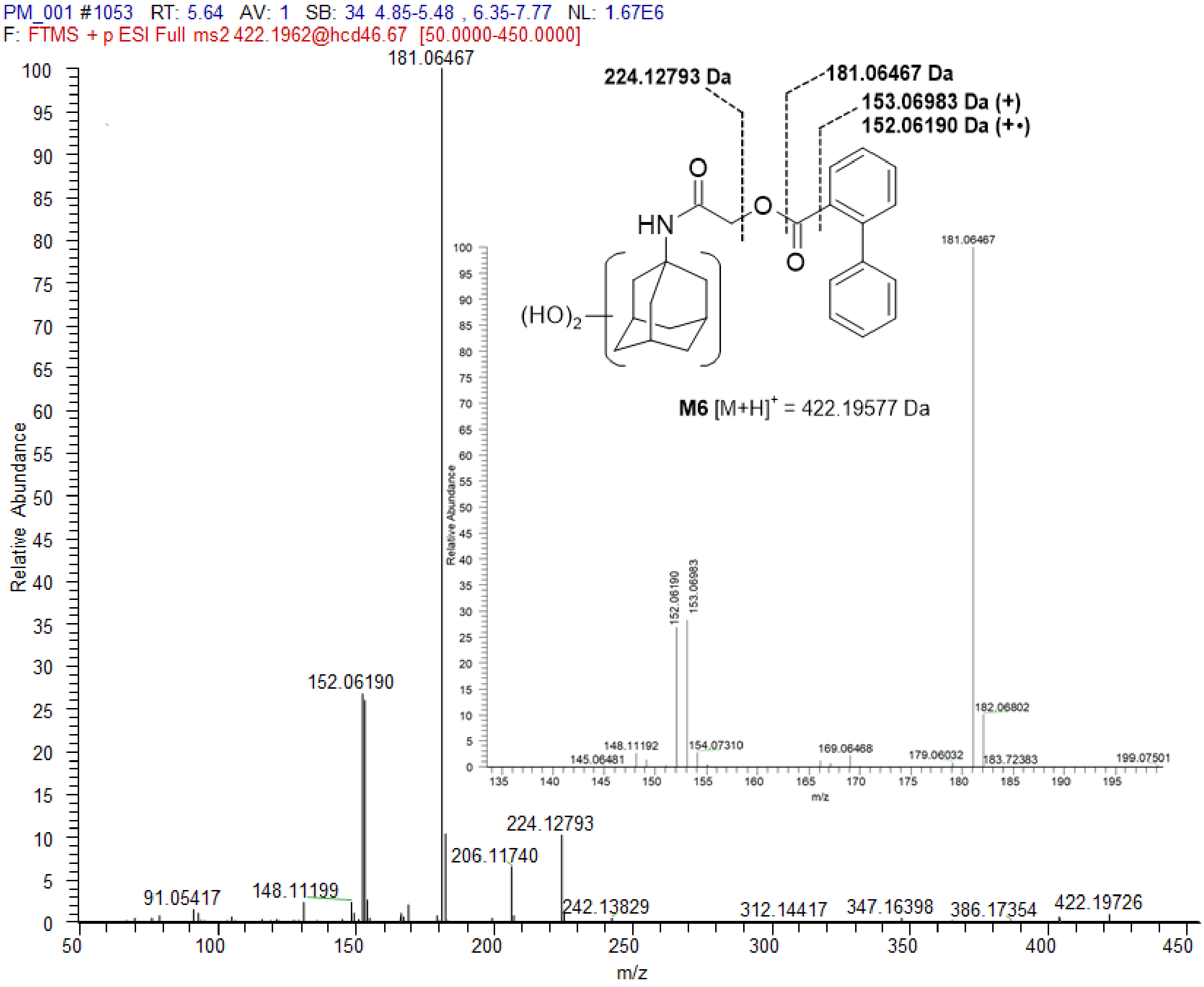
PM_001 #1053 RT: 5.64 AV: 1 SB: 34 4.85-5.48 , 6.35-7.77 NL: 1.67E6
F: FTMS + p ESI Full ms2 422.1962@hcd46.67 [50.0000-450.0000]
Relative Abundance
m/z
181.06467
152.06190
148.11199
91.05417
206.11740
224.12793
242.13829
312.14417
347.16398
386.17354
422.19726
224.12793 Da
181.06467 Da
153.06983 Da (+)
152.06190 Da (+•)
(HO)2
HN
O
M6 [M+H]+ = 422.19577 Da
182.06802
148.11192
145.06481
154.07310
169.06468
179.06032
183.72383
199.07501
152.06190
153.06983

- **M7**

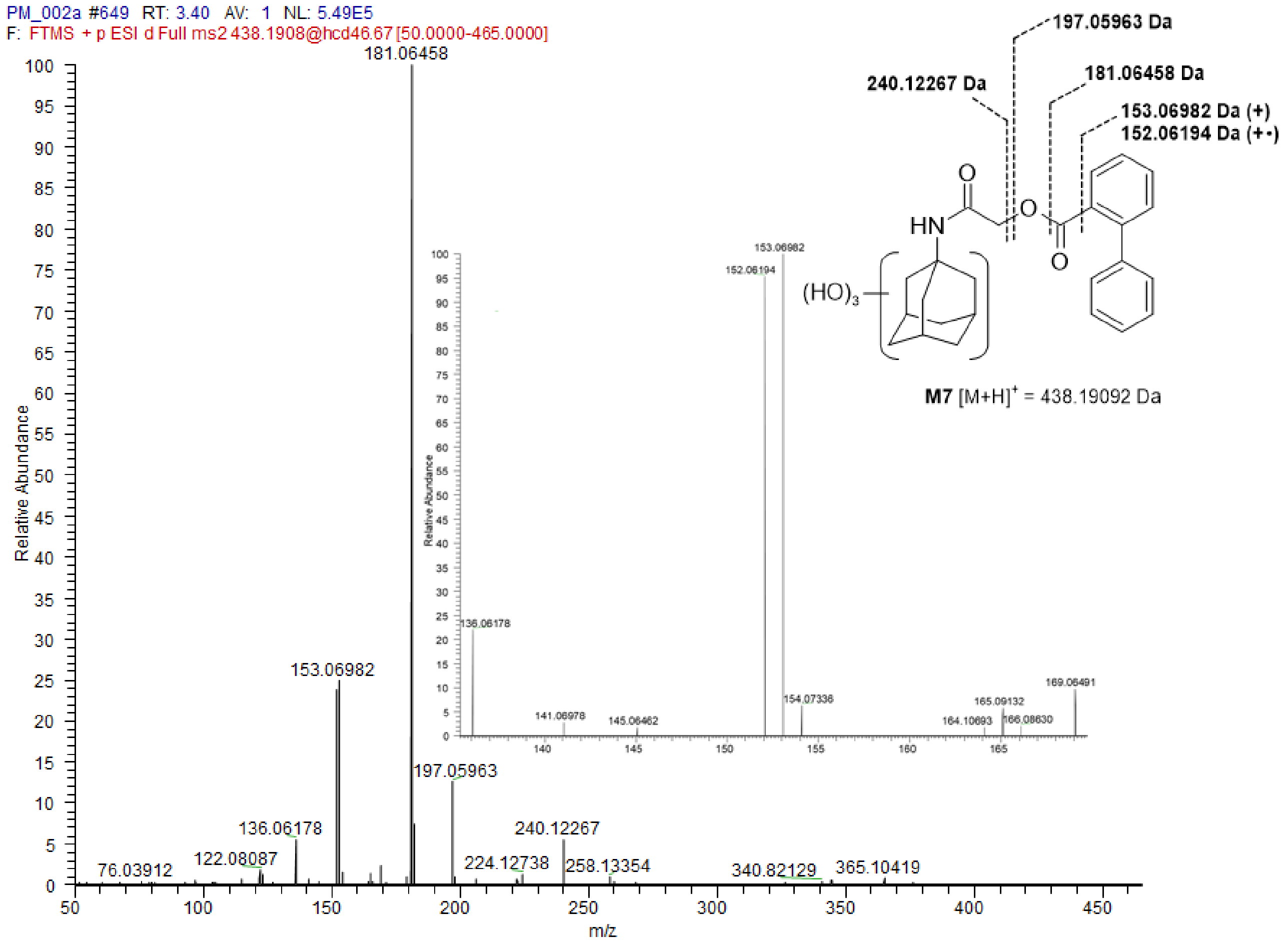

PM_002a #649 RT: 3.40 AV: 1 NL: 5.49E5
F: FTMS + p ESI d Full ms2 438.1908@hcd46.67 [50.0000-465.0000]
181.06458
153.06982
197.05963
136.06178
240.12267
122.08087
76.03912
224.12738
258.13354
340.82129
365.10419
Relative Abundance
m/z
152.06194
154.07336
141.06978
145.06462
165.09132
164.10693
166.08630
169.06491
197.05963 Da
240.12267 Da
181.06458 Da
153.06982 Da (+)
152.06194 Da (+•)
HN
O
(HO)3
M7 [M+H]+ = 438.19092 Da

- **M8**

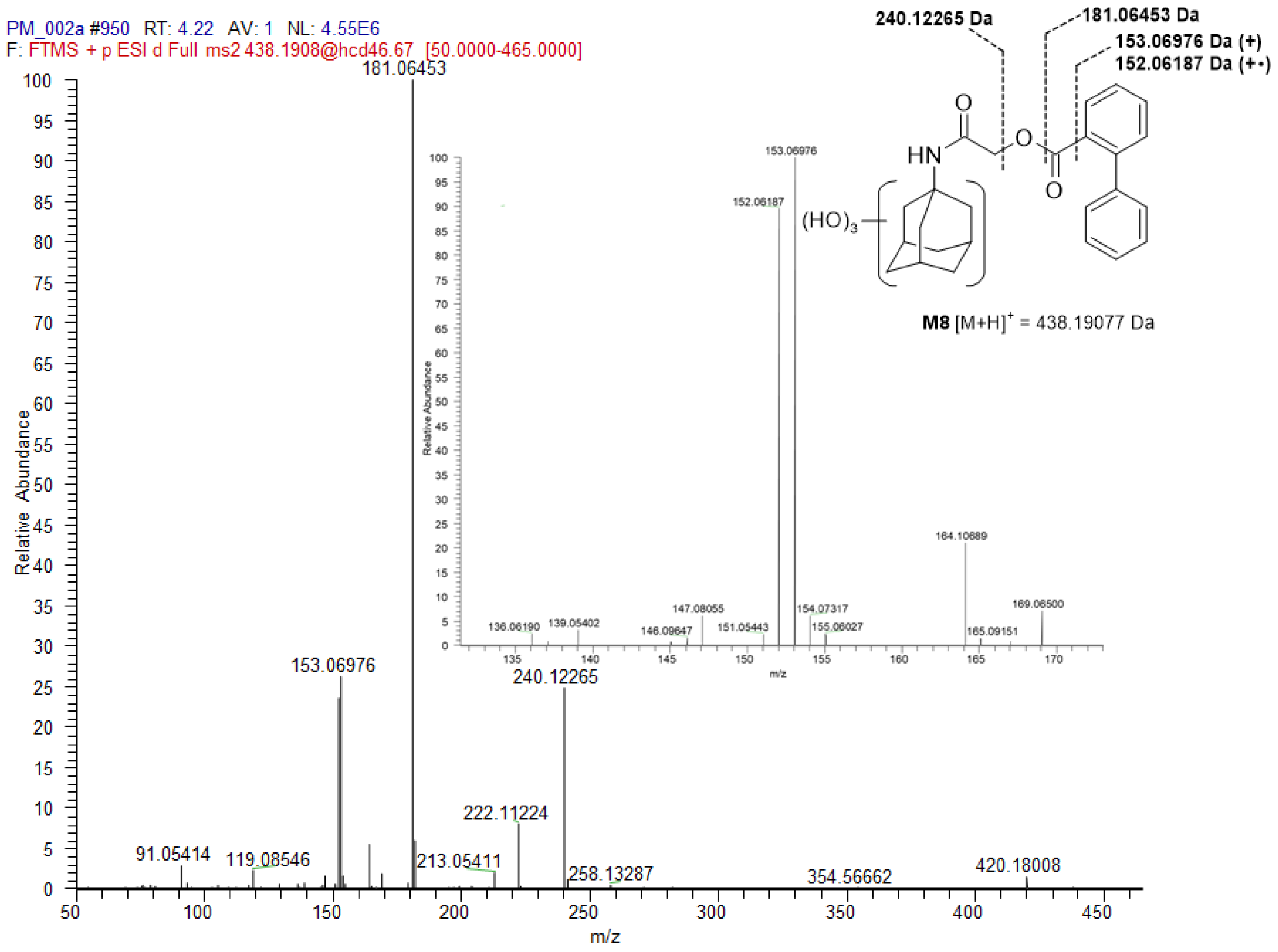

PM_002a #950 RT: 4.22 AV: 1 NL: 4.55E6
F: FTMS + p ESI d Full ms2 438.1908@hcd46.67 [50.0000-465.0000]
Relative Abundance
m/z
181.06453
153.06976
240.12265
222.11224
213.05411
258.13287
354.56662
420.18008
91.05414
119.08546
152.06187
164.10689
169.06500
147.08055
154.07317
155.06027
136.06190
139.05402
146.09647
151.05443
165.09151
240.12265 Da
181.06453 Da
153.06976 Da (+)
152.06187 Da (+•)
HN
O
(HO)3
M8 [M+H]+ = 438.19077 Da

- **M9**

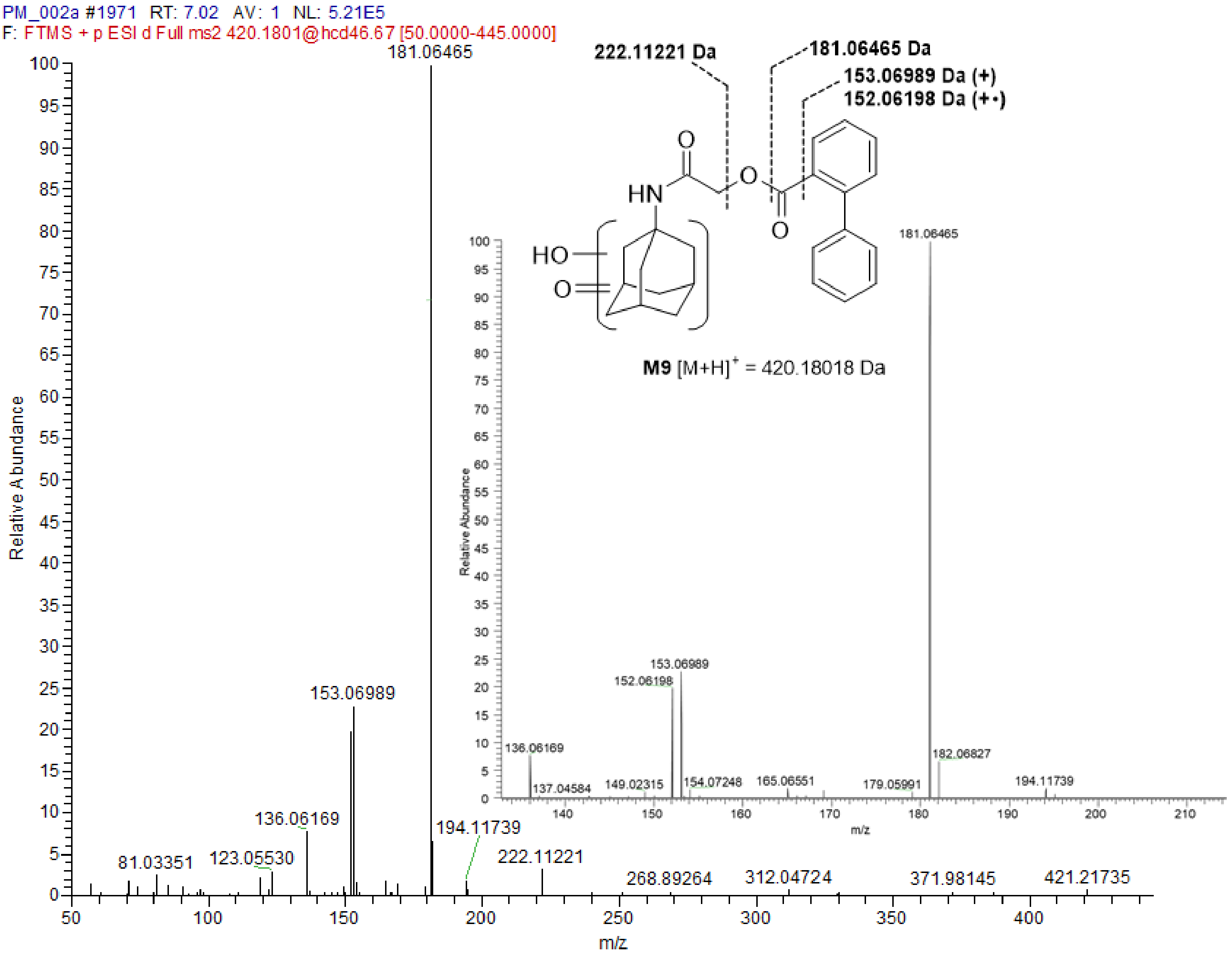

PM_002a #1971 RT: 7.02 AV: 1 NL: 5.21E5
F: FTMS + p ESI d Full ms2 420.1801@hcd46.67 [50.0000-445.0000]
222.11221 Da
181.06465 Da
153.06989 Da (+)
152.06198 Da (+•)
M9 [M+H]+ = 420.18018 Da
Relative Abundance
m/z
181.06465
153.06989
136.06169
123.05530
81.03351
194.11739
222.11221
268.89264
312.04724
371.98145
421.21735
152.06198
137.04584
149.02315
154.07248
165.06551
179.05991
182.06827

**Figure S80.** Compound Discoverer® (ver. 3.2) workflow used for compound **23** metabolite identification.

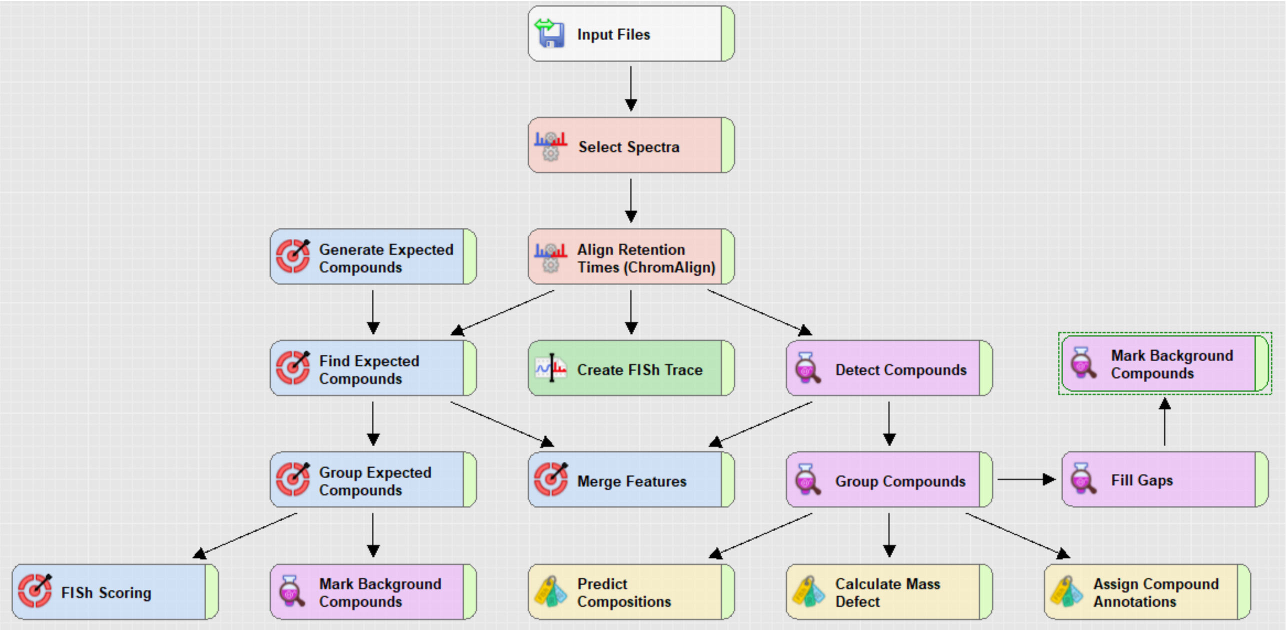

# Purity evaluation of selected compounds by HPLC-UV analysis.

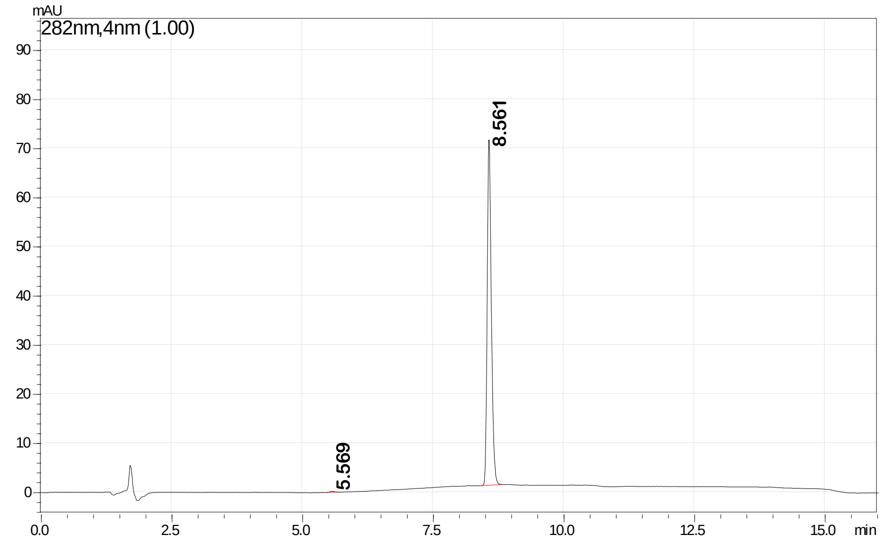


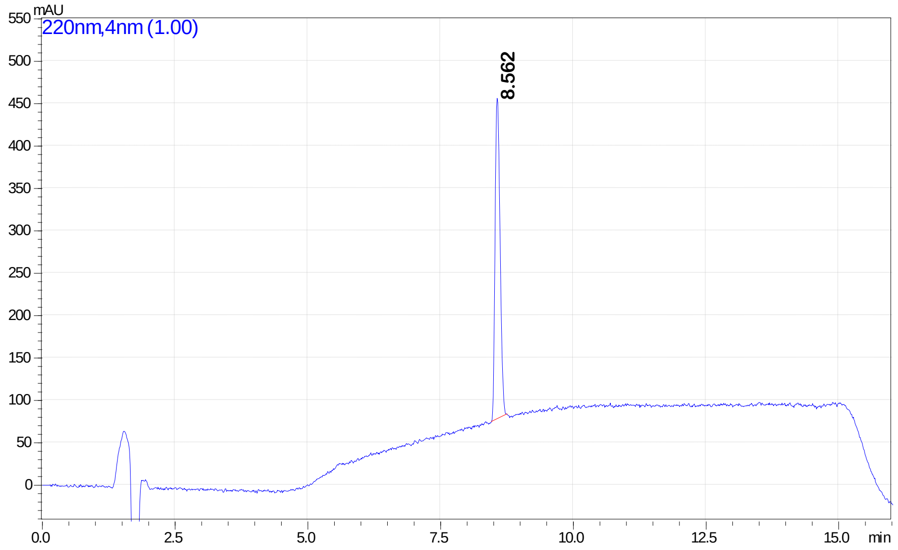


**Figure S81.** Purity $\lambda_{282nm}$>99%; purity $\lambda_{220nm}$>99% (method A) of compound **20**.

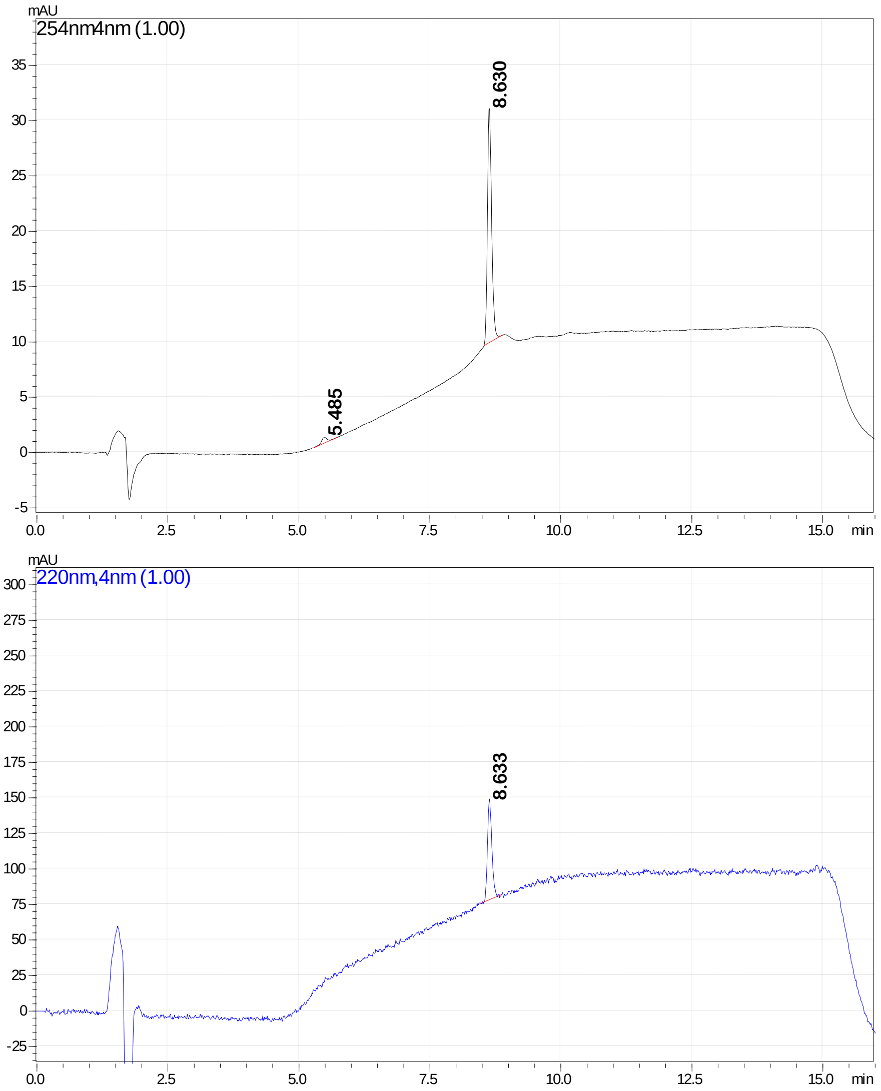


**Figure S82.** Purity $\lambda_{254nm}$=97%; purity $\lambda_{220nm}$>99% (method A) of compound **21**.

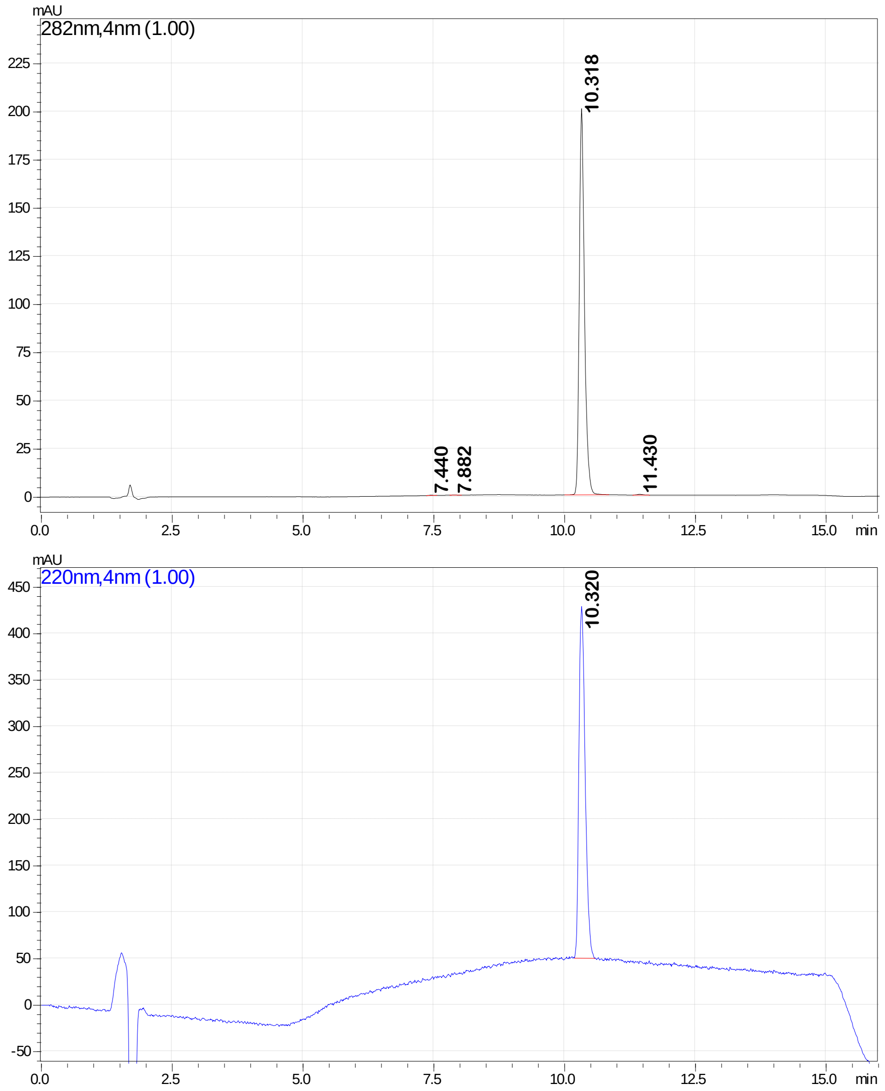


**Figure S83.** Purity $\lambda_{282nm}$>99%; purity $\lambda_{220nm}$>99% (method A) of compound **23**.

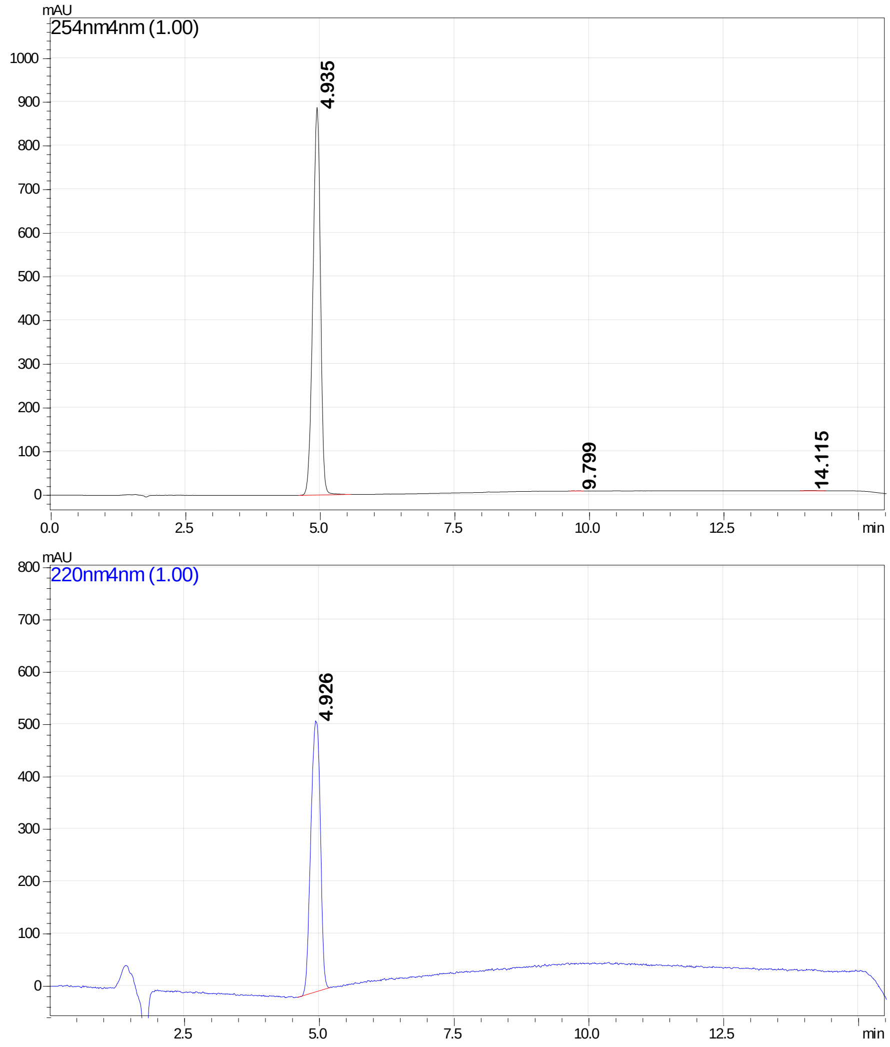


**Figure S84.** Purity $\lambda_{254nm}$>99%; purity $\lambda_{220nm}$>99% (method A) of compound **26**.

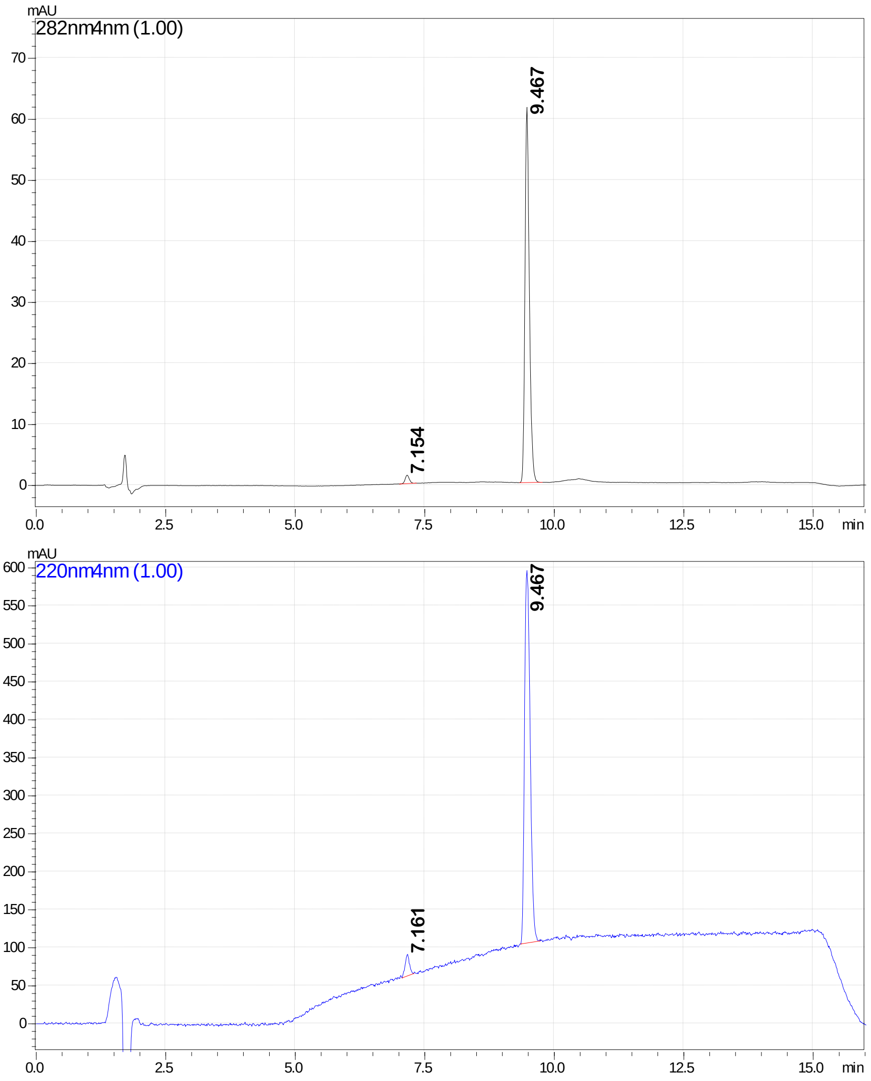


**Figure S85.** Purity $\lambda_{282nm}$=98%; purity $\lambda_{220nm}$=96% (method A) of compound **28**.

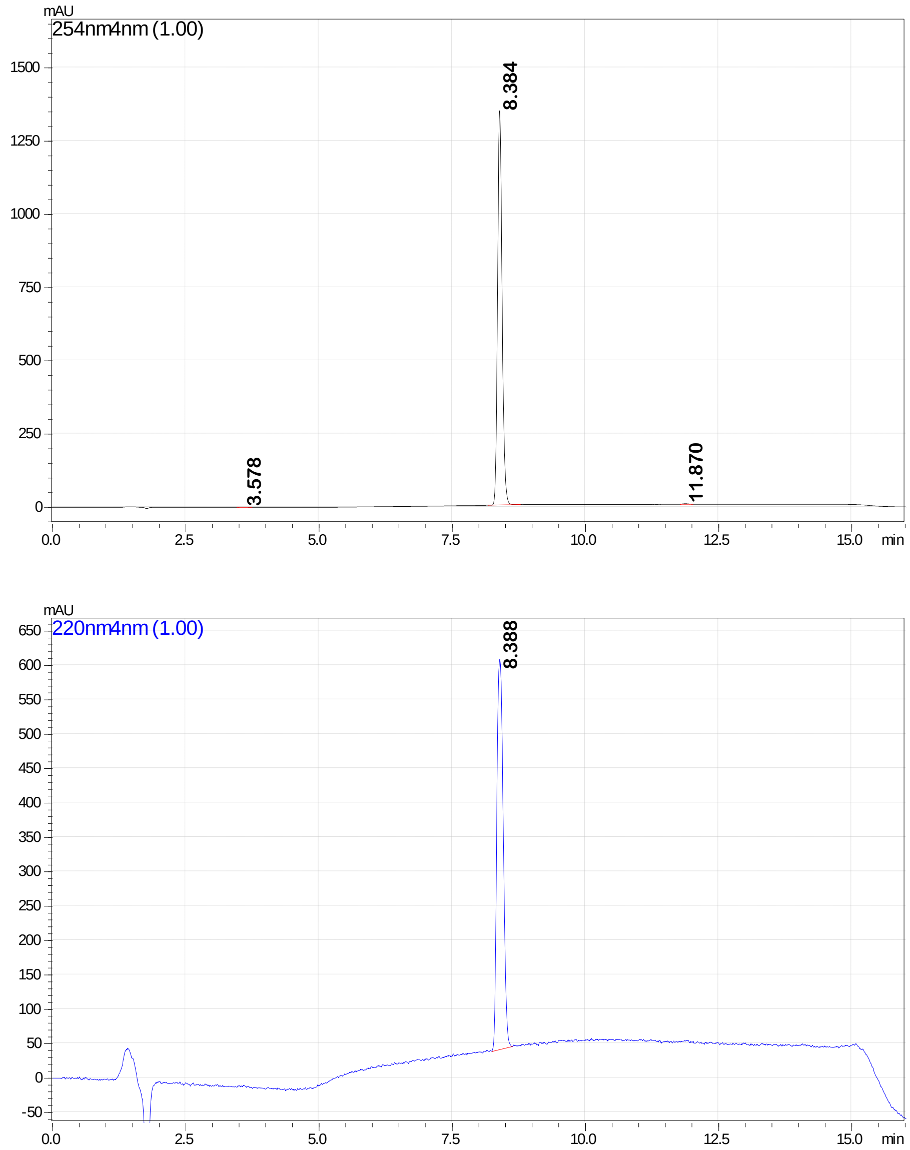


**Figure S86.** Purity $\lambda_{254nm}$>99%; purity $\lambda_{220nm}$>99% (method A) of compound **29**.

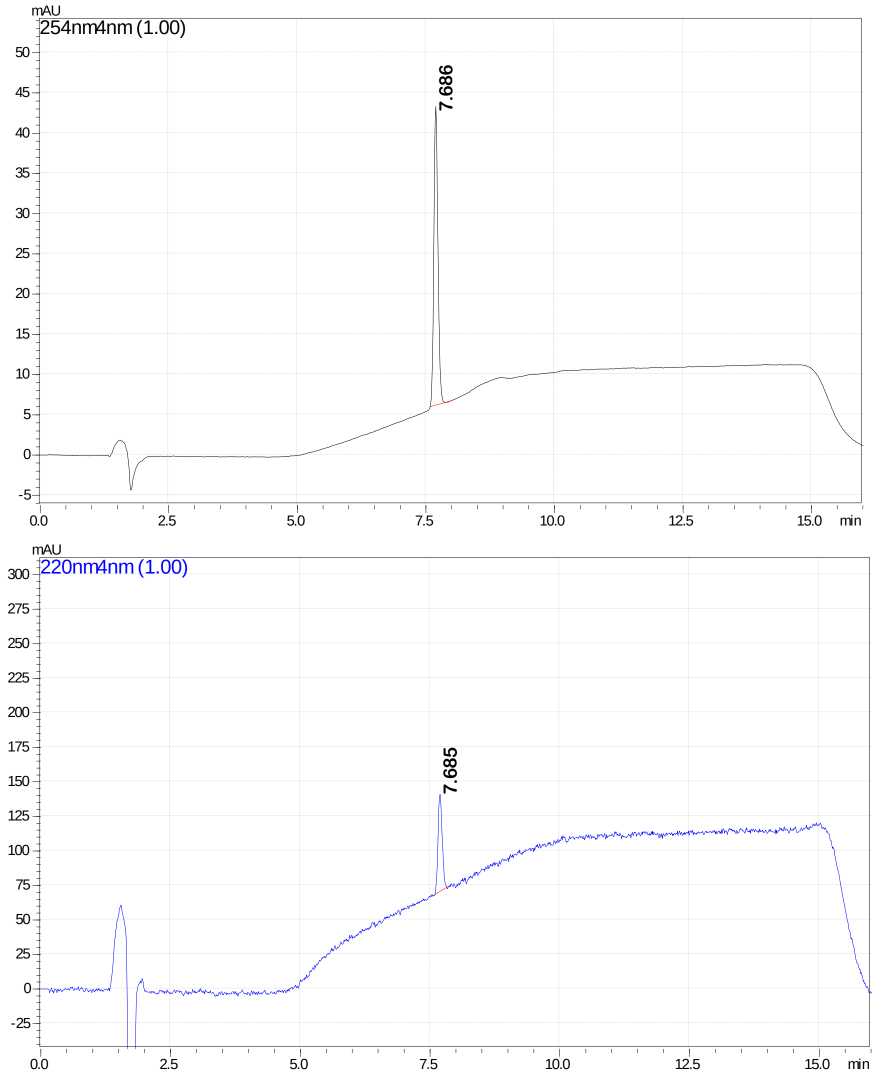


**Figure S87.** Purity $\lambda_{254nm}$>99%; purity $\lambda_{220nm}$>99% (method A) of compound **30**.

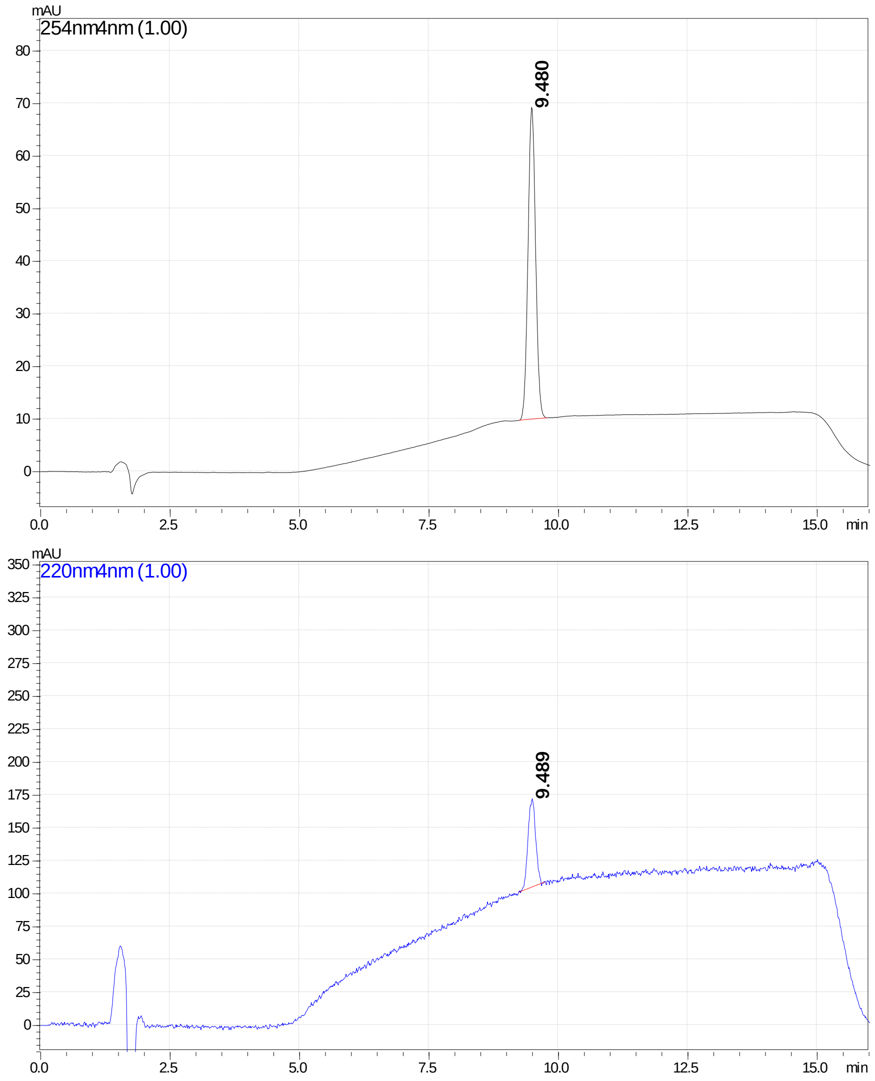


**Figure S88.** Purity $\lambda_{254nm}$>99%; purity $\lambda_{220nm}$>99% (method A) of compound **33**.

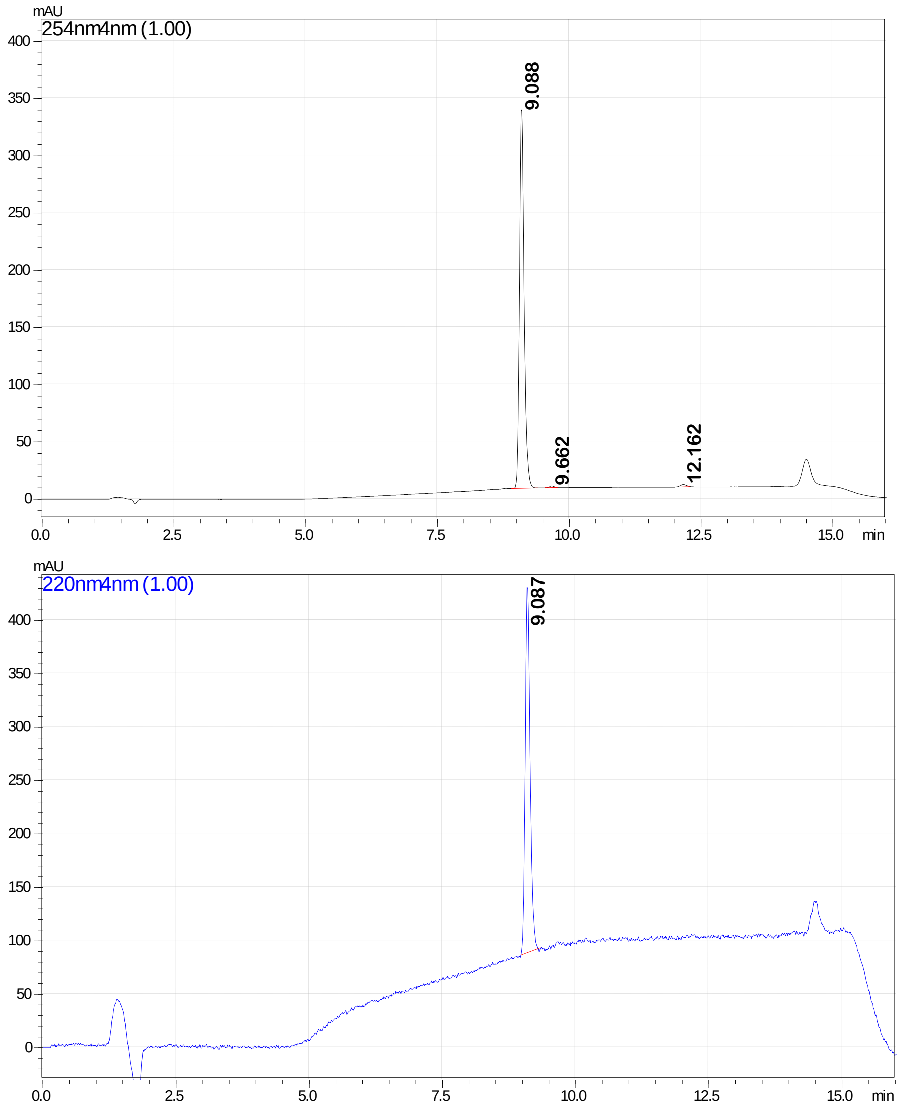


**Figure S89.** Purity $\lambda_{254nm}$=99%; purity $\lambda_{220nm}$>99% (method A) compound **35**.

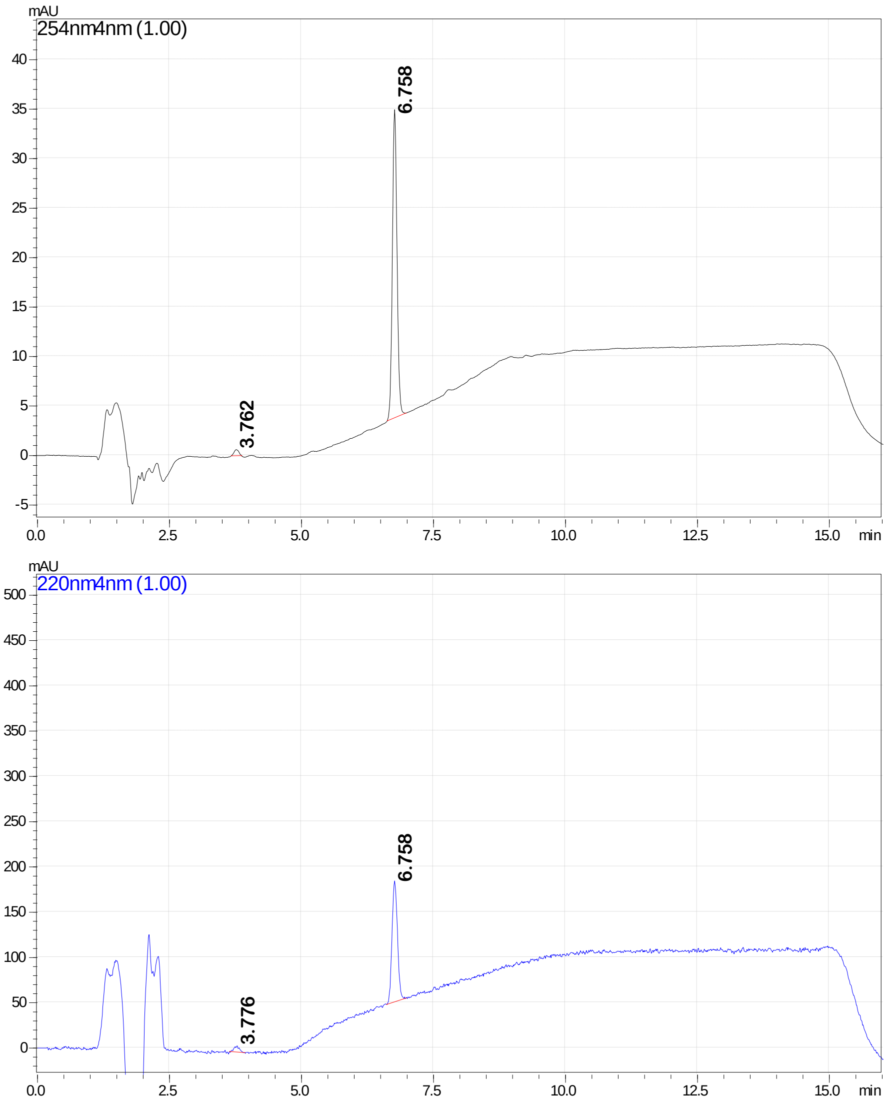


**Figure S90.** Purity $\lambda_{254nm}$=98%; purity $\lambda_{220nm}$=96% (method A) of compound **40**.

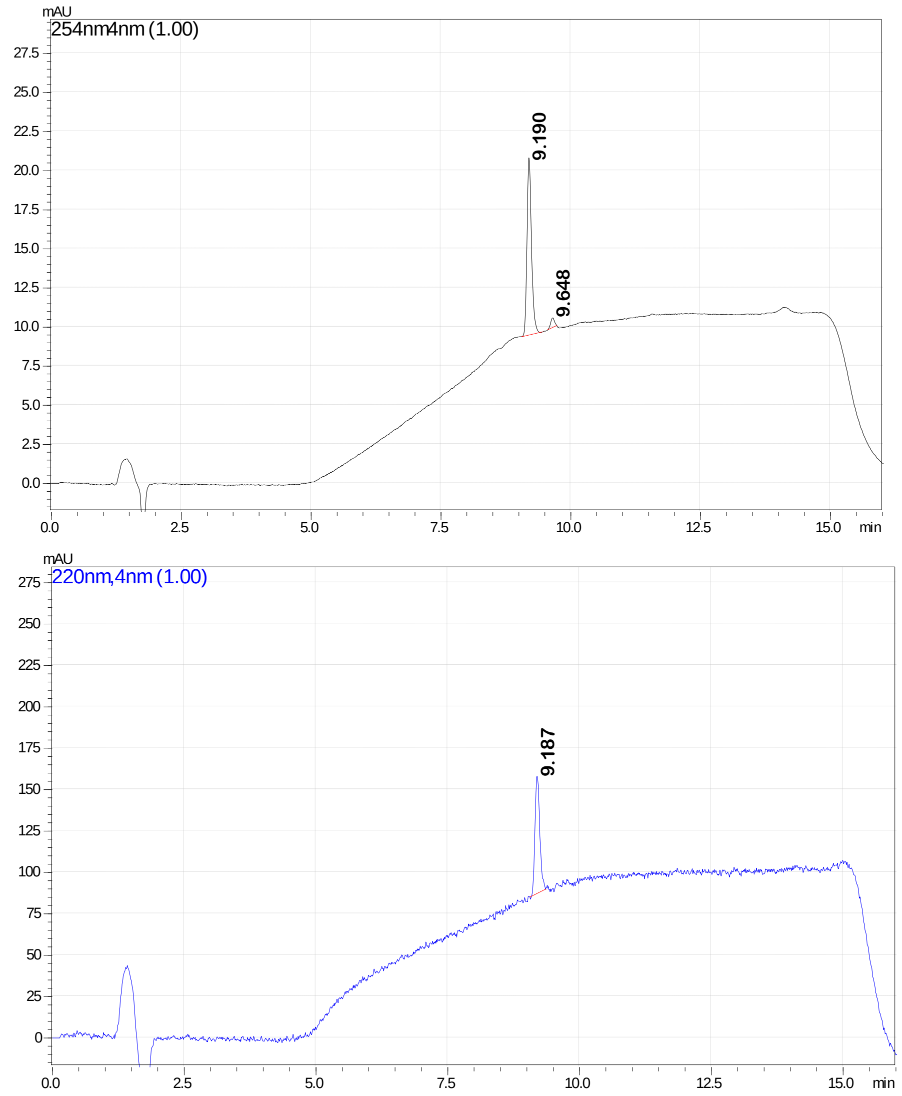


**Figure S91.** Purity $\lambda_{254nm}$=95%; purity $\lambda_{220nm}$>99% (method A) of compound **41**.

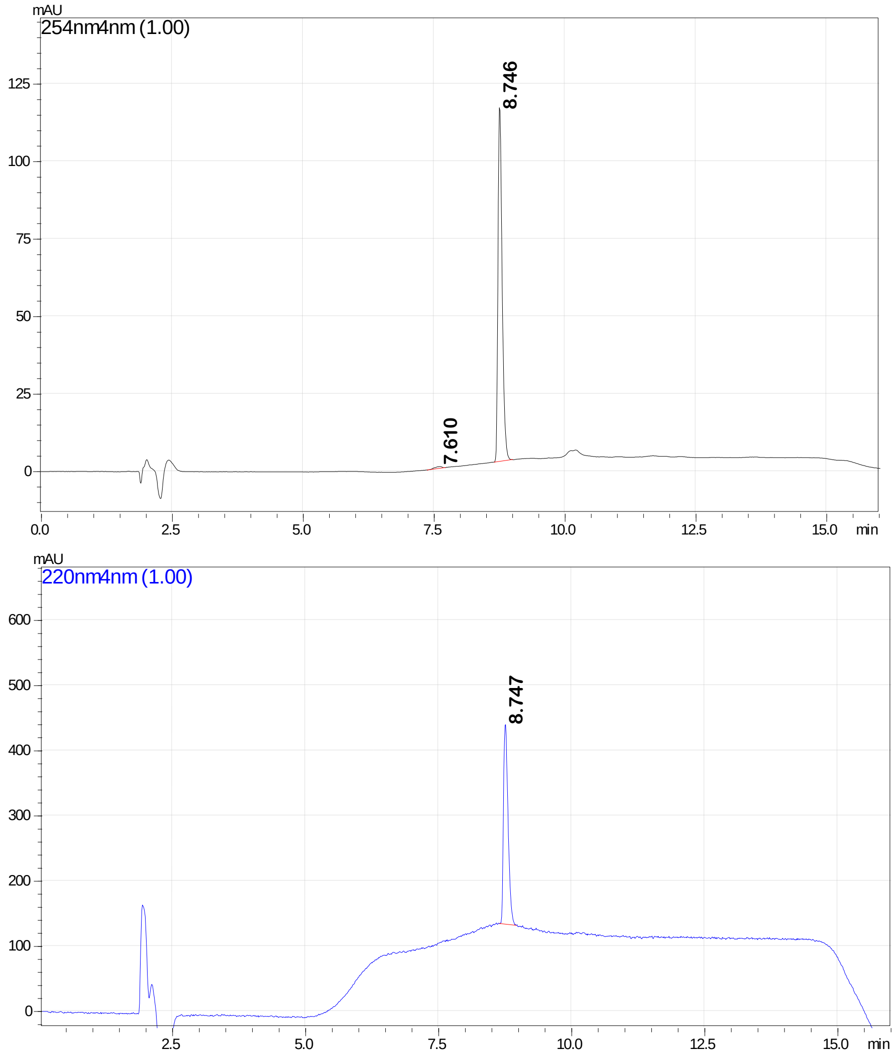


**Figure S92.** Purity $\lambda_{254nm}$= 99%; purity $\lambda_{220nm}$>99% (method B) of compound **42**.
